# Palladium-Catalyzed [5 + 2] Rollover Annulation of 1-Benzylpyrazoles with Alkynes: A Direct Entry to Tricyclic 2-Benzazepines

**DOI:** 10.1021/acs.orglett.2c04300

**Published:** 2023-01-31

**Authors:** Alejandro Suárez-Lustres, Nuria Martínez-Yáñez, Álvaro Velasco-Rubio, Jesús A. Varela, Carlos Saá

**Affiliations:** Centro Singular de Investigación en Química Biolóxica e Materiais Moleculares (CiQUS), Departamento de Química Orgánica, Universidade de Santiago de Compostela, 15782 Santiago de Compostela, Spain

## Abstract

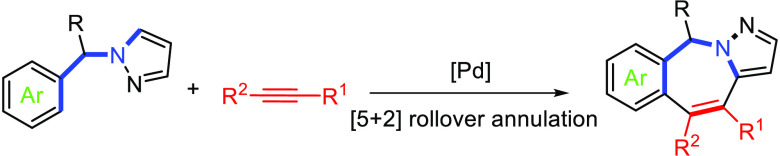

The first Pd-catalyzed [5 + 2] rollover annulation of 1-benzylpyrazoles with alkynes to assemble 10*H*-benzo[*e*]pyrazolo[1,5-*a*]azepines (tricyclic 2-benzazepines) has been developed. The rollover annulation implies a twofold C–H activation of aryl and heteroaryl C_sp^2^_–H bonds (C–H/C–H) of 1-benzylpyrazoles (five-atom partners) and alkynes to give the [5 + 2] annulated compounds.

2-Benzazepines, in particular their hetero-fused tricyclic derivatives, are privileged structures present in a wide number of compounds with a diverse range of relevant biological activities, including Aurora kinase A,^1^ bromodomain,^2^ and acetylcholinesterase^3^ inhibitory properties as well as antihepatitis C drugs^4^ (Figure 1).

**Figure 1:**
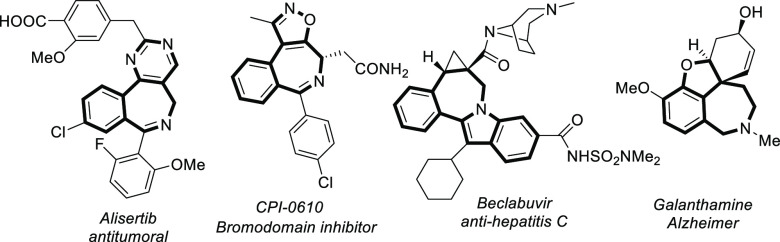
Biologically active tricyclic 2-benzazepines.

The remarkable biological activity of the 2-benzazepine scaffolds^5^ and the synthetic appeal of assembling benzo-fused seven-membered N-heterocyclic rings has stimulated rich synthetic creativity throughout the years. These synthetic approaches range from classical condensations,^6^ cyclizations,^7^ and metal-catalyzed cycloadditions with imines^8^ and nitriles^9^ to the promising Pd-catalyzed intramolecular C–H heteroarylations^10^ and intermolecular carbopalladations^11^ that allow rapid assembly of hetero-fused tricyclic derivatives. In recent years, more sustainable approaches based on intermolecular metal-catalyzed cycloadditions involving the direct activation of C–H bonds (oxidative annulations) have strongly emerged to build up medium-sized heterocycles.^12^ Thus, for 2-benzazepin(on)es, Glorius, Matsunaga/Yoshino, and Cui independently developed a convergent Rh-catalyzed [4 + 3] cycloaddition between benzamides and α,β-unsaturated carbonyls^13^ or vinylcarbenoids^14^ (Scheme 1a). Besides, Kim developed a Rh-catalyzed [4 + 3] cycloaddition between *N*-allyl benzylamines and allyl derivatives (Scheme 1a).^15^ On the other hand, Carretero exploited a Pd-catalyzed [6 + 1] cycloaddition of γ-arylpropylamine derivatives with CO (Scheme 1b).^16^ These annulations involve initial C_sp^2^_–H activation followed by condensation or amidation reactions or, alternatively, CH/NH functionalizations. More recently, Mascareñas and Gulías described the first assembly of 2-benzazepines in an interesting formal [5 + 2] annulation process involving the activation of C_sp^3^_–H bonds (Scheme 1c).^17^ Being aware of the capacity of pyrazoles to participate in metal-catalyzed C–H functionalizations^18^ via rollover processes,^19^ we herein report the first examples of efficient Pd-catalyzed [5 + 2] rollover annulations involving 1-benzylpyrazoles (five-atom partners) **1** with alkynes (two-carbon partners) **2** to afford tricyclic pyrazolo-2-benzazepines **3** in good to excellent yields (Scheme 1d). This rollover annulation implies an unusual twofold C–H activation of aryl and heteroaryl C_sp^2^_–H bonds (C–H/C–H), compared to the more typical annulation involving C–H/N–H activations (Scheme 1).

**Scheme 1:**
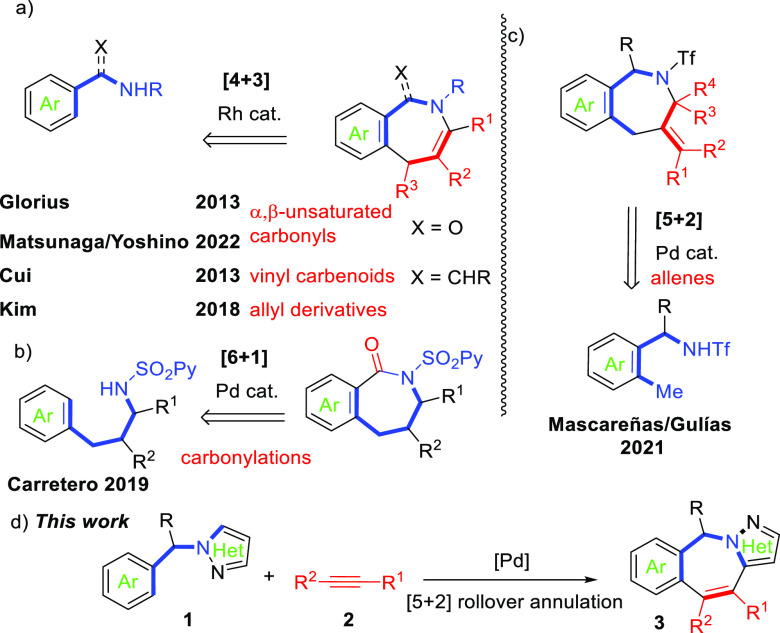
Metal-Catalyzed Oxidative Annulations to Form 2-Benzazepines

We started our investigation by testing the reactivity between 1-benzylpyrazole (**1a**) and diphenylacetylene (**2a**) as model partners under the known Miura’s rollover conditions^18^ for 1-phenylpyrazole (Table 1, entries 1–3). Unfortunately, the reaction did not proceed with either Cu(OAc)_2_ or AgOAc as the oxidant or xylene (150 °C) or toluene (100 °C) as the solvent. As the structure of **1a** contains a more flexible tetrahedrical C_sp^3^_ carbon compared to 1-phenylpyrazole, we thought that the formation of square-planar complexes might be more appropriate for catalytic C–H activation. Indeed, the reaction with Pd(OAc)_2_ as the catalyst and Cu(OAc)_2_ as the oxidant in MeCN gave the desired [5 + 2] rollover annulation product, tricyclic 2-benzazepine **3aa**,^19^ although in a low 20% yield (Table 1, entry 4). Using O_2_ as the oxidant or classical palladium/benzoquinone oxidative combinations in DMF gave slightly better yields of **3aa** (Table 1, entries 5 and 6). Typical metal oxidants like Cu(OAc)_2_ and AgOAc in DMF gave moderate yields of **3aa** (Table 1, entries 7 and 8). Interestingly, using AgOAc and PivOH (1 equiv) as an additive, to favor a presumable CMD process,^20^ led to **3aa** in a fairly good 75% yield (Table 1, entry 9).^21^ To our delight, when the amount of PivOH was increased to 5 equiv, **3aa** was obtained in an excellent 80% isolated yield (Table 1, entry 10).^22^ Under these conditions but using other solvents (e.g., toluene, DCE, MeCN, dioxane, *t*-AmOH, NMP, HFIP, etc.) at various temperatures gave poorer results.^23^ To evaluate the practicality of this novel protocol, a scaled-up reaction was performed, leading to **3aa** in fairly good yield even with a reduced amount of catalyst (Table 1, entry 11). The structure of compound **3aa** was elucidated by X-ray diffraction analysis.

**Table 1:**
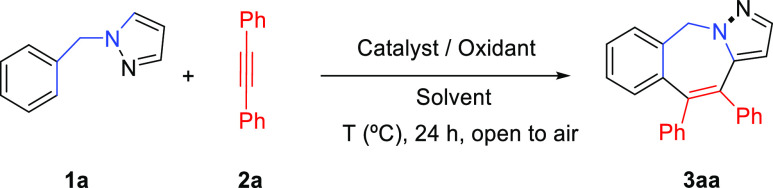
Optimization of the Reaction Conditions^a^

entry	cat.	oxidant	solvent^b^	*T* (°C)	yield (%)^c^
1^d^	[RhCp*Cl_2_]_2_	Cu(OAc)_2_·H_2_O Na_2_CO_3_	*m*-xyl	150	SM
2^d^	[RhCp*Cl_2_]_2_	AgOAc	*m*-xyl	150	SM
3^d^	[RhCp*Cl_2_]_2_	AgOAc	tol	100	SM
4	Pd(OAc)_2_	Cu(OAc)_2_	MeCN	105	20
5	Pd(OAc)_2_	O_2_/NaOAc	DMF	120	20
6	Pd(OAc)_2_	BQ/AcOH	DMF	120	33
7	Pd(OAc)_2_	Cu(OAc)_2_	DMF	120	50
8	Pd(OAc)_2_	AgOAc	DMF	120	64
9	Pd(OAc)_2_	AgOAc + PivOH (1 equiv)	DMF	120	75
10	Pd(OAc)_2_	AgOAc + PivOH (5 equiv)	DMF	120	88 (80)^e^
11^f^	Pd(OAc)_2_	AgOAc + PivOH (5 equiv)	DMF	120	68

a Typical conditions: **1a** (0.2 mmol, 1 equiv), **2a** (0.3 mmol, 1.5 equiv), catalyst (10 mol %), oxidant (2.1 equiv), solvent (2.0 mL), air atmosphere, unless otherwise stated.

b *m*-xyl, *m*-xylene; tol, toluene.

c Determined by ^1^H NMR analysis vs 1,3,5-trimethoxybenzene. The number in parentheses is the isolated yield.

d [RhCp*Cl_2_]_2_ (2.5 mol %).

e At 90 °C, **3aa** was isolated in 73% yield.

f **1a** (3 mmol), Pd(OAc)_2_ (5 mol %).

Having established the optimal conditions (Table 1, entry 10), we next investigated the scope and limitations of both reaction partners (Scheme 2). Symmetrical aryl alkynes **2b**–**2l** bearing an electron-donating group (Me, OMe) or an electron-withdrawing group (CF_3_, F, COOMe) at the *para* or *meta* position were well-tolerated and gave the corresponding pyrazolo-2-benzazepines **3ab**–**3al** in moderate to good yields.^25^ Pleasingly, aryl alkynes bearing halogens (Br, F) or coordinating groups (CN) afforded the products **3ad**, **3ag**, and **3aj** in relatively good yields. Unfortunately, dialkyl alkynes failed to react under the standard conditions.^26^ On the other hand, the unsymmetrical diaryl alkyne **2m** bearing substituents with different electronic properties gave **3am** in 56% yield as a 1.6:1 mixture of regioisomers. Conjugated alkynes such as methyl 3-phenylpropiolate (**2n**) and 1-phenylpropyne (**2o**) regioselectively gave the corresponding pyrazolo-2-benzazepines **3an** and **3ao**, albeit in relatively low yields.

**Scheme 2:**
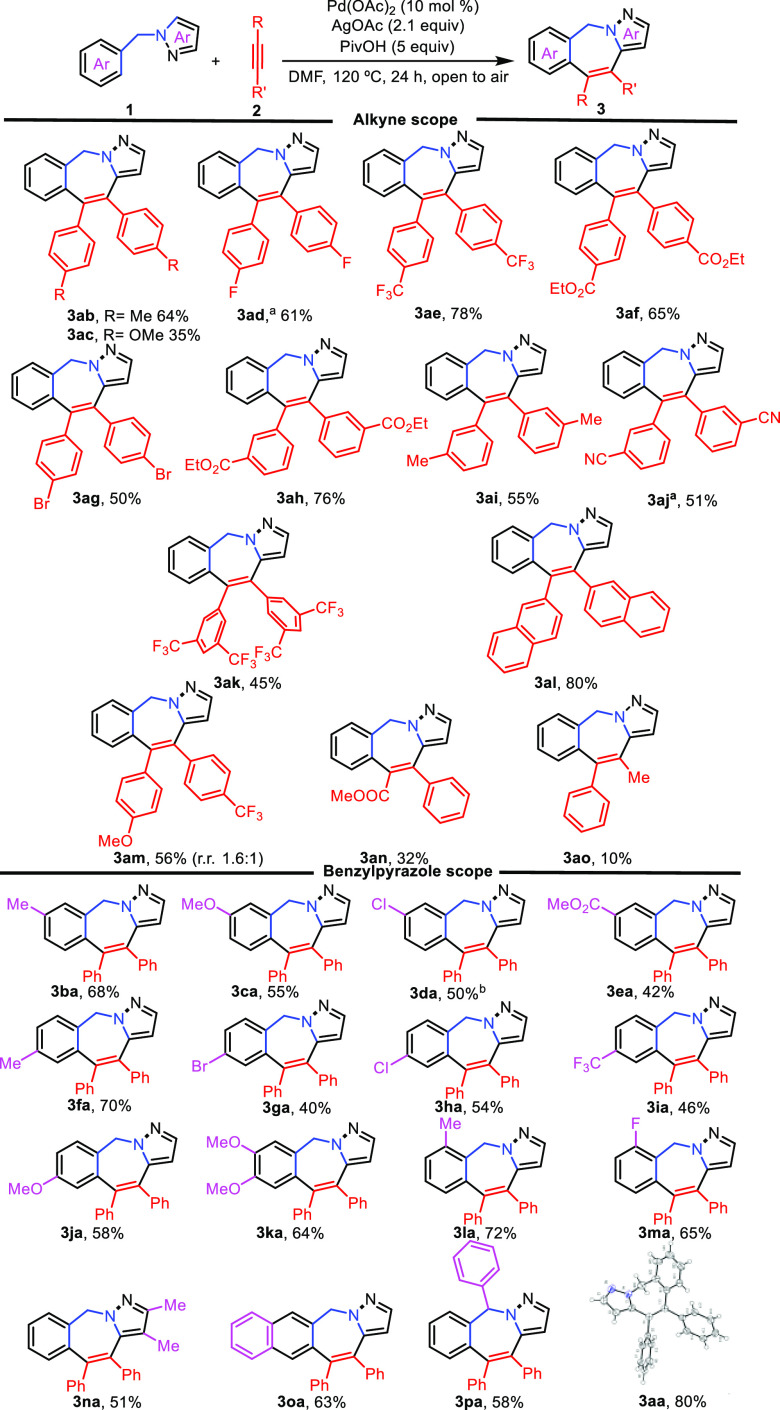
Scope of the Reaction PivOH (10 equiv). PivOH (15 equiv). Reaction conditions: **1** (1 equiv), **2** (1.5 equiv), DMF (0.1 M), 120 °C, 24 h, open to air. The ORTEP drawing of **3aa** shows ellipsoids at the 30% contour probability level.

The electronic effects of aryl substituents in **1** were then analyzed. On the one hand, substrates with electron-withdrawing or electron-donating substituents at the *meta* or *para* position gave comparable results (**3ba**–**3ka**). Pleasingly, halogenated substituents were tolerated at both positions, which would enable their future functionalization (**3da, 3ga, 3ha**), as well as substitution in *ortho* position (**3la, 3ma**). On the other hand, substituents on the pyrazole ring were also allowed (**3na**). In addition, other substituted substrates such as naphthalenyl- and 1-benzhydrylpyrazoles also participated, giving the corresponding pyrazole-2-benzazepines **3oa** and **3pa** in fairly good yields.

To gain insight into the reaction mechanism, a series of stoichiometric and catalytic experiments were conducted. The dimeric six-membered cyclometalated Pd(II) complex **4a**, which could be characterized by X-ray crystallography, was formed in 91% yield by heating **1a** with 1 equiv of Pd(OAc)_2_ in DCM for 5 h (Scheme 3, eq 1).^27^ Unlike the catalytic conditions (Table 1, entry 8), the stoichiometric reaction between dimeric palladacycle **4a** and alkyne **2a** needed the presence of PivOH to give **3aa** in 83% yield (Scheme 3, eq 2).^28^ Pleasingly, palladacycle **4a** can act as a catalyst to give the target product **3aa** in 84% yield under the optimized conditions (Scheme 3, eq 3). The competition between **1a** and the deuterated analogue **1a-*****d***_***5***_ showed a nonconclusive primary kinetic isotopic effect, suggesting that the first C–H bond activation might be the rate-determining step (Scheme 4, eq 4).

**Scheme 3:**
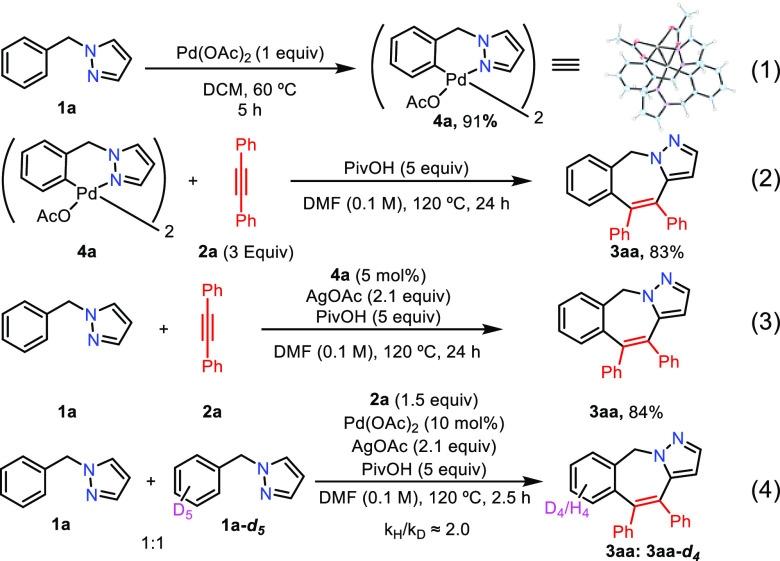
Mechanistic Studies

**Scheme 4:**
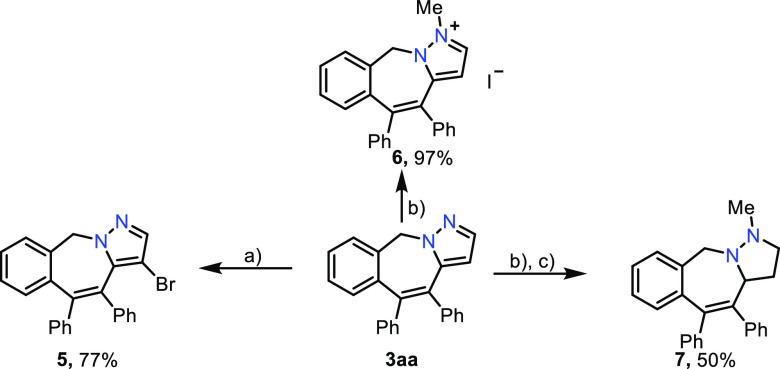
Derivatizations of **3aa**

With all these experimental data on hand, density functional theory (DFT) calculations^23^ for the reaction of **1a** with **2a** catalyzed by ^1^/_2_Pd_2_(OAc)_4_ in the presence of ^1^/_2_(AgOAc)_2_ and AcOH in DMF were performed. According to Fang and co-workers,^29^ starting materials coordinated to mononuclear palladium species represent the most plausible structures of the initial reaction complex under catalytic conditions. We started our calculations from complex **I**, which is isoenergetic with the starting materials (Figure 2).^30^ After agostic interaction of the *ortho* hydrogen of the phenyl ring in intermediate **II**,^30^ C–H activation would take place through **TS**_**II–III**_ (16.8 kcal mol^–1^) to give the six-membered palladacycle **III** lying at −7.4 kcal mol^–1^.^31^ Then 1,2-migratory insertion of the alkyne into the C–Pd bond occurs, most probably from Pd^II^–Ag^I^ bimetallic species **IV** through **TS**_**IV–V**_ at 12.2 kcal mol^–1^, after which N-decoordination gives **V**.^32^ Further decoordination of AgOAc to give **VI**(32) followed by a CMD process through **TS**_**VI–VII**_ (8.8 kcal mol^–1^) affords palladacycle **VII** (rollover process). Recoordination of AgOAc to form **VIII** followed by reductive elimination through **TS**_**VIII–IX**_ (7.5 kcal mol^–1^) would release **3aa** from the Pd^0^–Ag^I^ bimetallic complex **IX** (Δ*G*° = −17.7 kcal mol^–1^).^32^ An alternative mechanism involving a Pd(IV) species to favor a reductive elimination step was discarded since a catalytic reaction in the presence of oxidants (PhI(OAc)_2_, PIFA, Oxone, NFSI) failed while a stoichiometric experiment with Pd^II^(OAc)_2_ and PivOH in the absence of AgOAc gave **3aa** in almost quantitative yield.^23^

**Figure 2:**
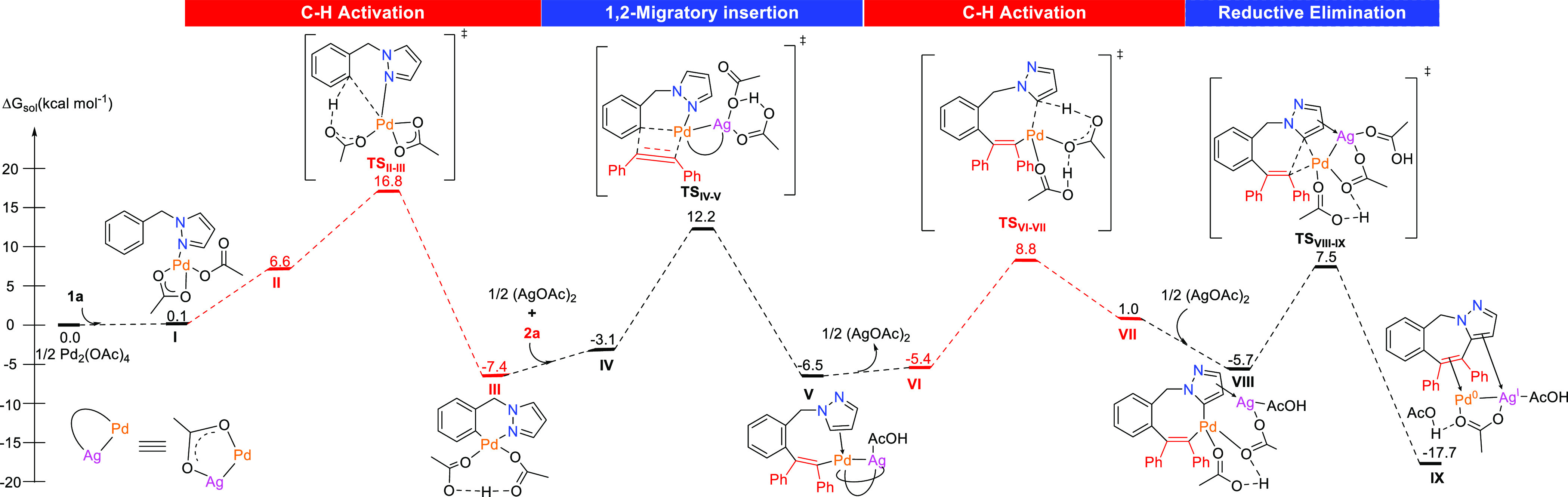
Free energy profile for the [5 + 2] rollover annulation of 1-benzylpyrazole (**1a**) with 1,2-diphenylacetylene (**2a**) catalyzed by monometallic Pd^II^ (in red) and bimetallic Pd^II^–Ag^I^ (in black) species. Computational studies were performed at the B3LYP-D3/6-311++G(d,p)-cc-pVTZ-pp_DMF(SMD)_//B3LYP-D3/6-31G(d,p)-LANL2DZ_DMF(SMD)_ level. Energies are relative to ^1^/_2_Pd_2_(OAc)_4_ combined with those of the relevant substrates.

However, under stoichiometric conditions, formation of the binuclear Pd species **4a** would be more plausible,^29^ which cannot undergo the 1,2-migratory insertion of the alkyne due to the high activation energy barrier (Δ*G*^⧧^ = 35.8 kcal mol^–1^) as experimentally observed.^23^ By using large amounts of an external ligand (PivOH or **1a**; Scheme 3, eqs 2 and 3),^23^ the reaction would return to the mononuclear Pd catalytic cycle, which is able to afford the product **3aa**.

Derivatizations of benzo[*e*]pyrazolo[1,5-*a*]azepine **3aa** were then analyzed (Scheme 4). Electrophilic bromination with NBS at room temperature afforded 4-bromopyrazole derivative **5** in a fairly good yield (77%, a). Alkylation with methyl iodide gave rise to pyrazolium salt **6** in an excellent 97% yield (b). Interestingly, reduction of the pyrazole to the tetrahydro derivative **7** could be accomplished using NaBH_4_ in EtOH at 60 °C in 50% yield (c).^33^

In summary, we have developed a new Pd-catalyzed rollover annulation of 1-benzylpyrazoles with alkynes to obtain benzo[*e*]pyrazolo[1,5-*a*]azepines (tricyclic 2-benzazepines). The seven-membered azepine ring was built based upon a new [5 + 2] rollover annulation that implies a twofold C–H activation of aryl and heteroaryl C_sp^2^_–H bonds (C–H/C–H) of 1-benzylpyrazoles with alkynes. The pyrazole moiety of the tricyclic 2-benzazepines can be readily functionalized, which highlights the potential utility of our approach. Further applications are currently in progress in our laboratory and will be reported in due course.

## Data Availability

The data underlying this study are available in the published article and its Supporting Information.

## Supporting Information Available

General experimental procedures, X-ray crystallographic data, and NMR spectra (PDF)
