# Supplementary material for: Palladium-Catalyzed [5 + 2] Rollover Annulation of 1-Benzylpyrazoles with Alkynes: A Direct Entry to Tricyclic 2-Benzazepines
Source: Org Lett. 2023 Jan 31;25(5):794–9. doi: 10.1021/acs.orglett.2c04300 (PMC9926515; doi:10.1021/acs.orglett.2c04300)
Supplement: Supplementary file 1 — ol2c04300_si_001.pdf [file ol2c04300_si_001.pdf]

Palladium-Catalyzed [5+2] Rollover Annulation of 1-Benzylpyrazoles with Alkynes: A Direct Entry to Tricyclic 2-Benzazepines

Alejandro Suárez-Lustres, Nuria Martínez-Yáñez, Álvaro Velasco-Rubio, Jesús A. Varela and Carlos Saá*

Centro Singular de Investigación en Química Biolóxica e Materiais Moleculares (CiQUS), Dpto. Química Orgánica, Universidade de Santiago de Compostela 15782, Santiago de Compostela, Spain
*Email: carlos.saa@usc.es

Supporting Information

Table of Contents

1. **General Experimental Procedures** S3
2. **Synthesis of Starting Materials** S4
3. **Optimization Data** S14
4. **Palladium-Catalyzed [5+2] Rollover Annulation of 1-Benzylpyrazoles 1 with Alkynes 2** S19
5. **Unsuccessful Substrates in the Pd-catalyzed [5+2] Rollover Annulation** S35
6. **Mechanistic Studies** S36
7. **X-Ray Crystallographic Data** S39
8. **Derivatizations** S41
9. **NMR Spectra** S44
10. **Computational details** S110
11. **DFT study for the [5+2] rollover annulation of 1-benzylpyrazole (1a) with 1,2-diphenylacetylene (2a) catalyzed by Pd^{II} monometallic species** S110
12. **DFT study for the [5+2] rollover annulation of 1-benzylpyrazole (1a) with 1,2-diphenylacetylene (2a) catalyzed by Pd^{II}-Ag^{I} bimetallic species** S111
13. **DFT study for the initial C-H activation in the pyrazole moiety catalyzed by Pd^{II} monometallic species** S112

14. DFT study for the initial C-H activation in the pyrazole moiety catalyzed by Pd^{II}-Ag^{I} bimetallic species S113

15. DFT study for the 1,2-migratory insertion of 1,2-diphenylacetylene (2a) into C-Pd bond of dinuclear complex 4a S114

16. References S115

17. Cartesian coordinates in Å, energy values in Hartrees and imaginary frequencies in cm^{-1} for transition states (TS) of species involved throughout DFT study S118

17.1. Cartesian coordinates in Å, energy values in Hartrees and imaginary frequencies in cm^{-1} for transition states (TS) of common structures S118

17.2. Cartesian coordinates in Å, energy values in Hartrees and imaginary frequencies in cm^{-1} for transition states (TS) of species in Figure S3 .... S123

17.3. Cartesian coordinates in Å, energy values in Hartrees and imaginary frequencies in cm^{-1} for transition states (TS) of species in Figure S4 .... S137

17.4. Cartesian coordinates in Å, energy values in Hartrees and imaginary frequencies in cm^{-1} for transition states (TS) of species in Figure S5 .... S155

17.5. Cartesian coordinates in Å, energy values in Hartrees and imaginary frequencies in cm^{-1} for transition states (TS) of species in Figure S6 .... S158

17.6. Cartesian coordinates in Å, energy values in Hartrees and imaginary frequencies in cm^{-1} for transition states (TS) of species in Figure S7 .... S161

1. General Experimental Procedures

All reactions were performed under an inert atmosphere of argon and with anhydrous solvents in glassware oven or flame dried at 80 ºC unless otherwise stated. All chemicals were purchased from Acros Organics Ltd., Aldrich Chemical Co. Ltd., Alfa Aesar, Strem Chemicals Inc., Fluorochem Ltd. or TCI Europe N. V. chemical companies and used without further purification, unless otherwise stated. Analytical thin layer chromatography was carried out on silica-coated aluminium plates (silica gel 60 F_{254} Merck) or on aluminium sheets (aluminium oxide 60 F_{254} neutral Merck) using UV light as visualizing agent (254 nm) and $KMnO_4$ (solution of 1.5 g of potassium permanganate, 10 g of potassium bicarbonate and 1.25 mL of 10% sodium hydroxide in 200 mL of water) with heat as developing agents. Flash column chromatography was performed on silica gel 60 (Merck, 230-400 mesh) with the indicated eluent. All other reagents and solvents: acetonitrile, dichloromethane, dichloroethane, tetrahydrofuran, toluene, dimethylformamide, xylene, dimethylsulfoxide and methanol were used dry, unless otherwise indicated.

^{1}H-NMR, ^{13}C-NMR, DEPT and ^{19}F-NMR experiments were carried out using a Varian Inova 500, Varian Inova 400 MHz or Varian Mercury 300 MHz. All NMR experiments were recorded at 298 K otherwise stated. All chemical shifts are reported in parts per million (ppm) and referenced to residual solvent peaks. Coupling constants J are given in Hertz (Hz). Multiplicities are reported as follows: s = singlet; d = doublet; t = triplet; q = quartet; aq = apparent quartet; p = pentet; ap = apparent pentet; m = multiplet or as a combination of them. Diastereomeric ration was determined in the ^{1}H-NMR of the crude mixture.

Mass spectrometry analysis was carried out using a Micromass AutoSpec, a TRACE MS or a HP-5988-A with chemical ionization and a Bruker Microtof APCI using chemical ionization spectrometers at CACTUS Facility (Universidade de Santiago de Compostela). Melting points were recorded in a Büchi Melting Point B-540 instrument.

X-ray crystallographic analysis was performed at the CACTUS facility of the University of Santiago de Compostela.

2. Synthesis of Starting Materials

Benzylpyrazoles 1

1-Benzylpyrazoles **1a-b, 1d-i, 1l-m and 1p** were synthesized in one step starting from commercially available benzyl bromides as depicted below.

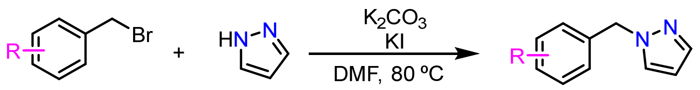

General procedure: N-alkylation with benzyl bromides

A flame-dried round bottom flask equipped with a magnetic stir bar was charged with benzyl bromide (1 equiv), pyrazole (1.5 equiv), potassium carbonate (1.5 equiv) and potassium iodide (0.01 equiv). A 0.8 M solution was prepared by addition of DMF. The reaction was stirred at 80 ºC overnight. After complete consumption of starting material (TLC monitoring, Hexane/EtOAc 8:2) the reaction was washed with brine and with a saturated aqueous solution of NH_4Cl (3 x), dried over anhydrous $MgSO_4$ and concentrated under reduced pressure. The crude product was purified by flash column chromatography on silica gel using typically hexane/EtOAc (8:2) as eluent.

The 1-benzylpyrazole **1c** was synthesized in two steps starting from commercially available (3-methoxyphenyl)methanol as depicted below.

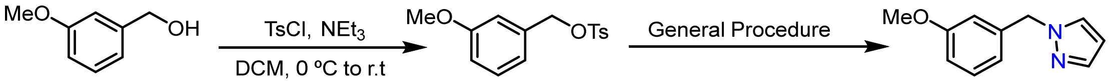

1c

N-alkylation with tosylates

Tosyl chloride (1.1 equiv) was dissolved in DCM (0.5 M) and cooled to 0 ºC. The benzylic alcohol (1 equiv) and triethylamine (2 equiv) were added dropwise. The solution was stirred at room temperature overnight. Then, H_2O was added for quenching. The aqueous layer was extracted with DCM (3 x). The organic layers were combined, dried over anhydrous $MgSO_4$, filtered and concentrated in vacuo. The crude product was used without further purification to carry out the *N*-alkylation following the general procedure.

N-alkylation with benzyl iodides

The 1-benzylpyrazoles **1j** and **1k** were synthesized in two steps starting from commercially available (4-methoxyphenyl)methanol and (3,4-dimethoxyphenyl)methanol, respectively, as depicted below. The 1-benzylpyrazole **1o** was synthesized from the alcohol derived from the reduction of commercially available 2-naphtaldehyde.[1] Deuterated 1-benzylpyrazole **1a-*d*$_5$** was synthesized from the commercially available benzyl alcohol-*d*$_5$.

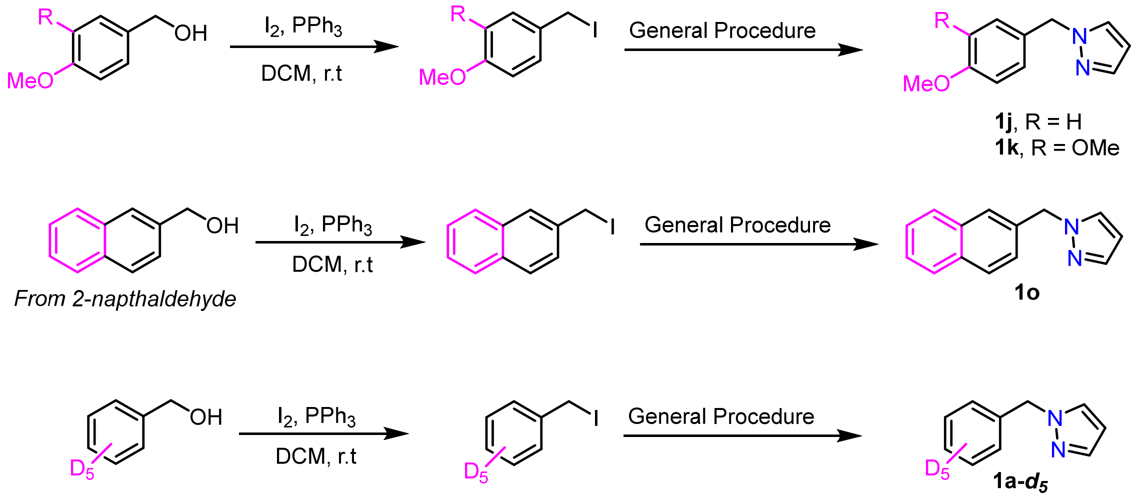

Following the reported procedure[2], a flame-dried flask was charged with PPh_3 (1.1 equiv), I_2 (1.1 equiv) and DCM (0.33 M). The resulting mixture was stirred 10 minutes at 0 ºC before the addition of the benzylic alcohol. The resulting mixture was slowly warmed to room temperature and stirred for 12 h. The reaction was quenched upon the addition of saturated aqueous solution of $Na_2S_2O_3$ (20 mL) and diluted with DCM (10 mL). The aqueous layer was extracted with DCM and the combined organic layers were washed with brine, dried over anhydrous $MgSO_4$, filtered, and concentrated under reduced pressure. The crude product was used without further purification to carry out the *N*-alkylation following the general procedure.

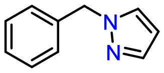

1a

1-Benzyl-*1H*-pyrazole 1a: known compound.[3] 20 mmol of benzyl bromide and 0.005 equiv of KI. The product **1a** was obtained as a colorless oil in 61% yield (1.94 g, 12.26 mmol). **^{1}H-NMR** (300 MHz, $CDCl_3$) δ (ppm): 7.55 (d, *J* = 1.9 Hz, 1H), 7.40 – 7.28 (m, 3H), 7.23 – 7.17 (m, 2H), 6.28 (t, *J* = 2.1 Hz, 1H), 5.33 (s, 2H).

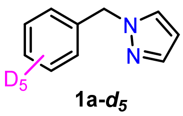

1a-*d*₅

1-((Phenyl-*d*$_5$)methyl)-*1H*-pyrazole 1a-*d*$_5$, 4.4 mmol of benzyl-2,3,4,5,6-*d*$_5$ alcohol and 0.005 equiv of KI. The product was obtained as a pale yellow oil in 67% yield (0.48 g, 2.95 mmol). **^{1}H NMR** (300 MHz, $CDCl_3$) δ (ppm): 7.58 (d, *J* = 1.5 Hz, 1H), 7.40 (d, *J* = 2.2 Hz, 1H), 6.30 (t, *J* = 2.1 Hz, 1H), 5.36 (s, 2H). **^{13}C NMR** (75 MHz, $CDCl_3$) δ (ppm): 139.5 (CH), 136.5 (C), 129.4 (CH), 106.1 (CH), 56.0 (CH_2). **HMRS (APCI-FIA-TOF)** m/z: $[M+H]^+$ Calcd for $C_{10}H_6D_5N_2$ 164.1231; found 164.1227.

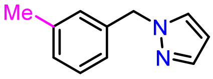

1b

1-(3-Methylbenzyl)-*1H*-pyrazole 1b: known compound.[4] 4.2 mmol of 1-(bromomethyl)-3-methylbenzene and 0.05 equiv of KI. The product **1b** was obtained as a pale-yellow oil in 73% yield (0.53 g, 3.1 mmol). **^{1}H NMR** (300 MHz, $CDCl_3$) δ (ppm): 7.61 – 7.53 (m, 1H), 7.40 (t, *J* = 2.7 Hz, 1H), 7.25 (td, *J* = 7.6, 2.4 Hz, 1H), 7.13 (d, *J* = 7.6 Hz, 1H), 7.07 – 7.00 (m, 2H), 6.32 – 6.27 (m, 1H), 5.31 (s, 1H), 2.35 (s, 2H).

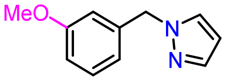

1c

1-(3-Methoxybenzyl)-*1H*-pyrazole 1c, 10 mmol of (4-methylbenzyl 4-methylbenzenesulfonate. Eluent: hexane/EtOAc (1:1). The product **1c** was obtained as a colorless oil in 23% yield (0.43 g, 2.28 mmol). **^{1}H NMR** (300 MHz, $CDCl_3$) δ (ppm): 7.57 (d, *J* = 1.9 Hz, 1H), 7.40 (d, *J* = 2.2 Hz, 1H), 7.31 – 7.23 (m, 1H), 6.89 – 6.79 (m, 2H), 6.76 (s, 1H), 6.30 (t, *J* = 2.1 Hz, 1H), 5.32 (s, 2H), 3.79 (s, 3H). **^{13}C NMR** (75 MHz, $CDCl_3$) δ (ppm): 160.1 (C), 139.6 (CH), 138.3 (C), 130.0 (CH), 129.4 (CH), 120.0 (CH), 113.7 (CH), 113.4 (CH), 106.2 (CH), 56.0 (CH_2), 55.4 (CH_3). **HRMS (APCI-FIA-TOF)** m/z: $[M+H]^+$ Calcd for $C_{11}H_{13}N_2O$ 189.1022; found 189.1023.

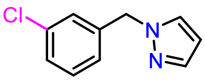

1d

1-(3-Chlorobenzyl)-*1H*-pyrazole 1d, 4.0 mmol of 1-(bromomethyl)-3-chlorobenzene. The product **1d** was obtained as colorless oil in 67% yield (0.52 g, 2.69 mmol). **^{1}H NMR** (300 MHz, $CDCl_3$) δ (ppm): 7.57 (d, *J* = 1.9 Hz, 1H), 7.41 (d, *J* = 2.3 Hz, 1H), 7.31 – 7.26 (m, 2H), 7.18 (s, 1H), 7.11 – 7.06 (m, 1H), 6.31 (t, *J* = 2.1 Hz, 1H), 5.30 (s, 2H). **^{13}C NMR** (75 MHz, $CDCl_3$) δ (ppm): 140.0 (CH), 138.9 (C), 134.8 (C), 130.2 (CH), 129.5 (CH), 128.3 (CH), 127.7 (CH), 125.7 (CH), 106.4 (CH), 55.3 (CH_2). **HRMS (APCI-FIA-TOF)** m/z: $[M+H]^+$ Calcd for $C_{10}H_{10}ClN_2$ 193.0527; found 193.0528.

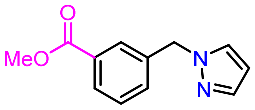

1e

Methyl 3-((*1H*-pyrazol-1-yl)methyl)benzoate 1e: known compound.[5] 5 mmol of methyl 3-(bromomethyl)benzoate. Eluent: hexane/EtOAc (1:1). The product **1e** was obtained as a yellow oil in 66% yield (0.71 g, 3.30 mmol). **^{1}H NMR** (300 MHz, $CDCl_3$) δ (ppm): 8.00 (dd, *J* = 7.2, 1.7 Hz, 1H), 7.94 (s, 1H), 7.59 (d, *J* = 1.9 Hz, 1H), 7.50 – 7.36 (m, 3H), 6.32 (t, *J* = 2.1 Hz, 1H), 5.39 (s, 2H), 3.93 (s, 3H).

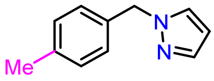

1f

1-(4-Methylbenzyl)-*1H*-pyrazole 1f: known compound.[6] 5 mmol of 1-(bromomethyl)-4-methylbenzene. The product **1f** was obtained as a colorless oil in 85% yield (0.73 g, 4.22 mmol). **^{1}H NMR** (300 MHz, $CDCl_3$) δ (ppm): 7.57 (d, *J* = 1.9 Hz, 1H), 7.38 (d, *J* = 2.3 Hz, 1H), 7.20 – 7.11 (m, 4H), 6.29 (t, *J* = 2.1 Hz, 1H), 5.30 (s, 2H), 2.36 (s, 3H).

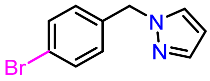

1g

1-(4-Bromobenzyl)-*1H*-pyrazole 1g, 3.76 mmol of 1-bromo-4-(bromomethyl)benzene and 0.005 equiv of KI. The product **1g** was obtained as a yellow solid in 61% yield (0.54 g, 2.29 mmol). **^{1}H NMR** (300 MHz, $CDCl_3$) δ (ppm): 7.56 (d, *J* = 1.9 Hz, 1H), 7.51 – 7.44 (m, 2H), 7.40 (d, *J* = 2.4 Hz, 1H), 7.13 – 7.04 (m, 2H), 6.30 (t, *J* = 2.1 Hz, 1H), 5.28 (s, 2H). **^{13}C NMR** (75 MHz, $CDCl_3$) δ (ppm): 139.8 (CH), 135.8 (C), 131.9 (2 x CH), 129.30 (CH), 129.25 (2 x CH), 122.0 (C), 106.2 (CH), 55.2 (CH_2). **HRMS (APCI-FIA-TOF)** m/z: $[M+H]^+$ Calcd for $C_{10}H_{10}N_2Br$ 237.0022; found 237.0020.

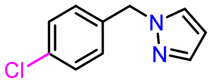

1h

1-(4-Chlorobenzyl)-*1H*-pyrazole 1h, 5.0 mmol of 1-(bromomethyl)-4-chlorobenzene. The product **1h** was obtained as a colorless oil in 72% yield (0.69 g, 3.6 mmol). **^{1}H NMR** (300 MHz, $CDCl_3$) δ (ppm): 7.59 – 7.55 (m, 1H), 7.42 – 7.37 (m, 1H), 7.35 – 7.29 (m, 2H), 7.17 – 7.12 (m, 2H), 6.30 (td, *J* = 2.1, 1.0 Hz, 1H), 5.30 (s, 2H). **^{13}C NMR** (75 MHz, $CDCl_3$) δ (ppm): 139.9 (CH), 135.4 (C), 134.1 (C), 129.3 (CH), 129.1 (CH), 129.0 (CH), 106.3 (CH), 55.3 (CH_2). **HRMS (APCI-FIA-TOF)** m/z: $[M+H]^+$ Calcd for $C_{10}H_{10}N_2Cl$ 193.0527; found 193.0528.

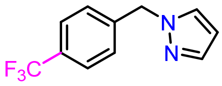

1i

1-(4-(Trifluoromethyl)benzyl)-*1H*-pyrazole 1i, 10 mmol of 1-(bromomethyl)-4-(trifluoromethyl)benzene. The product **1i** was obtained as a colorless oil in 65% yield (0.73 g, 3.24 mmol). **^{1}H NMR** (300 MHz, $CDCl_3$) δ (ppm): 7.61 (d, *J* = 8.2 Hz, 3H), 7.44 (d, *J* = 2.3 Hz, 1H), 7.32 – 7.27 (m, 2H), 6.34 (t, *J* = 2.1 Hz, 1H), 5.40 (s, 2H). **^{13}C NMR** (75 MHz, $CDCl_3$) δ (ppm): 140.9 (C), 140.2 (CH), 130.4* (q, *J* = 32.7 Hz), 129.6 (CH), 127.8 (2xCH), 126.9 (CH), 125.9 (CH, q, *J* = 3.9 Hz), 122.3 (C), 106.5 (CH), 55.4 (CH_2). **^{19}F NMR** (282 MHz, $CDCl_3$) δ (ppm): -62.69. **HRMS (APCI-FIA-TOF)** m/z: $[M+H]^+$ Calcd for $C_{11}H_{10}N_2F_3$ 227.0791; found 227.0788.

*: *Only 2 peaks of the CF_3 (q) are clearly visible*

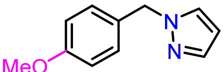

1j

1-(4-Methoxybenzyl)-*1H*-pyrazole 1j: known compound.[5] 7.5 mmol of 1-(iodomethyl)-4-methoxybenzene. The product **1j** was obtained as a colorless oil in 43% yield (0.60 g, 3.2 mmol). **^{1}H NMR** (300 MHz, $CDCl_3$) δ (ppm): 7.53 (d, *J* = 1.4 Hz, 1H), 7.34 (d, *J* = 2.2 Hz, 1H), 7.17 (d, *J* = 8.6 Hz, 2H), 6.87 (d, *J* = 8.6 Hz, 2H), 6.25 (t, *J* = 2.0 Hz, 1H), 5.25 (s, 2H), 3.78 (s, 3H).

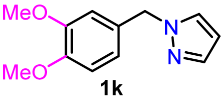

1k

1-(3,4-Dimethoxybenzyl)-*1H*-pyrazole 1k, 5 mmol of 4-(iodomethyl)-1,2-dimethoxybenzene. Eluent: Hex/EtOAc (3:7). The product **1k** was obtained as a pale-yellow oil in 66% yield (0.72 g, 3.31 mmol). **^{1}H NMR** (300 MHz, $CDCl_3$) δ (ppm): 7.51 (d, *J* = 1.9 Hz, 1H), 7.33 (d, *J* = 2.3 Hz, 1H), 6.81 – 6.76 (m, 2H), 6.76 – 6.70 (m, 1H), 6.23 (t, *J* = 2.1 Hz, 1H), 5.21 (s, 2H), 3.82 (s, 3H), 3.79 (s, 3H). ^{13}C NMR (75 MHz, $CDCl_3$) δ (ppm): 149.2 (C), 148.8 (C), 139.3 (CH), 129.1 (C), 128.9 (CH), 120.2 (CH), 111.3 (CH), 111.0 (CH), 105.8 (CH), 55.85 (CH_3), 55.79 (CH_3), 55.6 (CH_2). **HRMS (APCI-FIA-TOF)** m/z: $[M+H]^+$ Calcd for $C_{12}H_{15}N_2O_2$ 219.1128; found 219.1130.

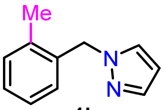

1l

1-(2-Methylbenzyl)-*1H*-pyrazole 1l, 5.0 mmol of 1-(bromomethyl)-2-methylbenzene. The product **1l** was obtained as a yellow oil in 74% yield (0.63g, 3.68 mmol). **^{1}H NMR** (300 MHz, $CDCl_3$) δ (ppm): 7.59 (dd, *J* = 9.0, 2.0 Hz, 1H), 7.28 (d, *J* = 2.3 Hz, 1H), 7.25 – 7.17 (m, 3H), 7.04 (dd, *J* = 6.7, 2.1 Hz, 1H), 6.28 (t, *J* = 2.1 Hz, 1H), 5.35 (s, 2H), 2.29 (s, 3H). **^{13}C NMR** (75 MHz, $CDCl_3$) δ (ppm): 139.4 (CH), 136.6 (C), 134.6 (C), 130.7 (CH), 129.1 (CH), 128.9 (CH), 128.4 (CH), 126.5 (CH), 105.9 (CH), 54.2 (CH_2), 19.1 (CH_3). **HRMS (APCI-FIA-TOF)** m/z: $[M+H]^+$ Calcd for $C_{11}H_{13}N_2$ 173.1073; found 173.1073.

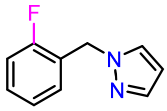

1m

1-(2-Fluorobenzyl)-*1H*-pyrazole, 10 mmol of 1-(bromomethyl)-2-fluorobenzene and 0.005 equiv of KI. The product **1m** was obtained as a colorless oil in 70% yield (1.23 g, 6.98 mmol). **^{1}H NMR** (300 MHz, $CDCl_3$) δ (ppm): 7.56 (s, 1H), 7.46 (d, *J* = 2.3 Hz, 1H), 7.29 (d, *J* = 7.4 Hz, 1H), 7.20 – 7.01 (m, 3H), 6.29 (t, *J* = 2.1 Hz, 1H), 5.39 (s, 2H). **^{13}C NMR** (75 MHz, $CDCl_3$) δ (ppm): 160.6 (C-F, d, *J* = 247.1 Hz), 139.6 (CH), 130.4 (C, *d*, *J* = 2.9 Hz), 130.2 (CH, *d*, *J* = 4.0 Hz), 130.1 (CH, d, *J* = 2.8 Hz), 129.7 (CH, d, *J* = 5.24 Hz), 124.7 (CH, d, *J* = 3.8 Hz), 115.6 (CH, d, *J* = 21.4 Hz), 106.2 (CH), 49.6 (CH2, d, *J* = 4.3 Hz). **HRMS (APCI-FIA-TOF)** m/z: $[M+H]^+$ Calcd for $C_{10}H_{10}N_2F$ 177.0823; found 177.0822.

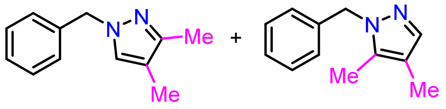

1n **1n'**

Ratio 2.4:1

1-Benzyl-3,4-dimethyl-*1H*-pyrazole 1n and **1-Benzyl-4,5-dimethyl-1H-pyrazole 1n'**, 4.2 mmol of benzyl bromide. A mixture of regioisomers **1n** and **1n'** (2.4:1 ratio) was obtained as a colorless oil in 99% yield (0.78 g, 4.17 mmol). **^{1}H NMR** (500 MHz, $CDCl_3$) δ (ppm): 7.35 – 7.27 (m, 3.3H), 7.25 (d, *J* = 8.1 Hz, 0.6H), 7.21 – 7.17 (m, 1.6H), 7.10 – 7.06 (m, 1H), 7.05 (s, 1H, **1n**), 5.26 (s, 2H, **1n'**), 5.18 (s, 2H, **1n**), 2.20 (s, 3H, **1n**), 2.09 (s, 3H, **1n'**), 1.99 (s, 3H, **1n'**), 1.97 (s, 3H, **1n**). **^{13}C NMR** (126 MHz, $CDCl_3$) δ (ppm): 147.5 (C), 139.1 (CH), 137.5 (C), 137.3 (C), 135.6 (C), 128.83 (2xCH), 128.81 (2xCH), 128.6 (CH, **1n**), 127.9 (2xCH), 127.7 (2xCH), 127.6 (CH), 126.9 (CH), 114.5 (C), 114.0 (C), 55.7 (CH_2, **1n**), 53.4 (CH_2, **1n'**), 11.7 (CH_3, **1n**), 9.4 (CH_3, **1n'**), 8.9 (CH_3, **1n'**), 8.5 (CH_3, **1n**).

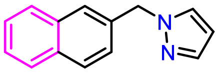

1o

1-(Naphthalen-2-yl)methyl)-*1H*-pyrazole 1o: known compound.[5] The starting naphthalen-2-ylmethanol was obtained from 2-naphthaldehyde (5 mmol) following the reported procedure.[1] Eluent: Hexane/EtOAc (8:2). The product **1o** was obtained as a pale yellow solid in 62% yield (0.58 g, 2.80 mmol). **^{1}H NMR** (300 MHz, $CDCl_3$) δ (ppm): 7.86 – 7.77 (m, 3H), 7.67 (d, *J* = 7.6 Hz, 2H), 7.54 - 7.46 (m, 2H), 7.40 – 7.38 (m, 1H), 7.34 (d, *J* = 8.5 Hz, 1H), 6.33 (t, *J* = 2.1 Hz, 1H), 5.43 (s, 2H).

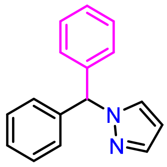

1p

1-Benzhydryl-*1H*-pyrazole 1p: known compound.[7] In a round-bottomed flask provided with a refrigerant and magnetic stirring bar were introduced, in the following order, 66.7 ml of xylene, *1H*-pyrazole (0.68 g, 10 mmol, 1 equiv), potassium hydroxide (0.56 g, 10 mmol, 1 equiv), tetrabutylammonium bromide (0.16 g, 0.5 mmol, 0.05 equiv) and (bromomethylene)dibenzene (2.47 g, 10 mmol, 1 equiv). After refluxing 20 h, the reaction mixture was allowed to cool to rt and purified by column chromatography on silica gel using Hexane/EtOAc (8:2) as eluent to afford **1p** as a white solid in 70% yield (1,66 g, 7.09 mmol). **^{1}H NMR** (300 MHz, $CDCl_3$) δ (ppm): 7.64 (d, J = 1.2 Hz, 1H), 7.40 – 7.32 (m, 6H), 7.31 – 7.29 (m, 1H), 7.16 – 7.09 (m, 4H), 6.85 (s, 1H), 6.31 (t, J = 2.1 Hz, 1H).

Alkynes 2

Diphenylacetylene **2a**, methyl phenylpropiolate **2n** and 1-phenyl-1-propyne **2o** were directly purchased from Aldrich, bis(4-bromophenyl)acetylene **2g** from Apollo Scientific. The other alkynes were synthesized following the reported procedures.

General Procedure: one-pot synthesis of symmetrical bisarylethynes

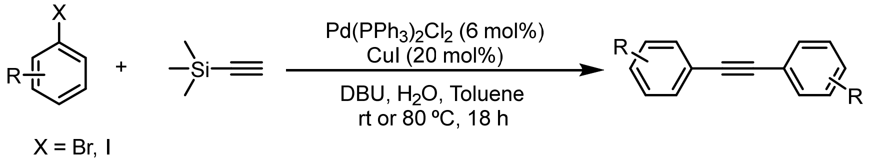

Following the reported procedure,[8] a flame-dried flask was charged with $PdCl_2(PPh_3)_2$ (0.060 equiv) and CuI (0.20 equiv). Then, dry toluene in order to form a 0.1 M solution, DBU (12 equiv), H_2O (0.8 equiv) and the corresponding aryl bromide or iodide were added. Argon was bubbled into the solution for 5 to 10 minutes and finally trimethylsillylacetylene (1 equiv) was added. The resulting mixture was protected from light and stirred at room temperature (X = I) or at 80 °C (X = Br). After 18 h Et_2O (50 mL) and H_2O (50 mL) were added. The aqueous layer was washed with 10% HCl (3 x 75 mL) and with brine (75 mL). The organic layer was dried over anhydrous $MgSO_4$, filtered, and concentrated under reduced pressure. The crude product was purified by flash column chromatography on silica gel using a gradient from pure hexane to hexane: EtOAc (9:1) as eluent.

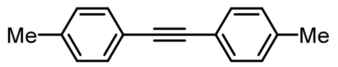

2b

1,2-Di-p-tolylethyne 2b: known compound.[8] From 1-iodo-4-methylbenzene (1.30 mmol). Yellow solid (125 mg, 93%). **^{1}H NMR** (300 MHz, $CDCl_3$) δ (ppm): 7.42 (d, J = 7.7 Hz, 4H), 7.15 (d, J = 7.5 Hz, 4H), 2.37 (s, 6H).

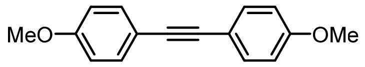

2c

1,2-Bis(4-methoxyphenyl)ethyne 2c: known compound.[8] From 1-iodo-4-methoxybenzene (1.30 mmol). Off-white solid (110 mg, 71%). ^{1}H NMR (300 MHz, $CDCl_3$) δ (ppm): 7.45 (d, J = 8.0 Hz, 4H), 6.87 (d, J = 8.0 Hz, 4H), 3.82 (s, 6H).

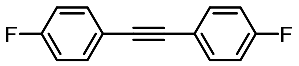

2d

1,2-Bis(4-fluorophenyl)ethyne 2d: known compound.[8] From 1-fluoro-4-iodobenzene (10 mmol). White solid (532 mg, 44%). **^{1}H NMR** (300 MHz, $CDCl_3$), δ (ppm): 7.57 – 7.44 (m, 4H), 7.05 (t, J = 8.6 Hz, 4H).

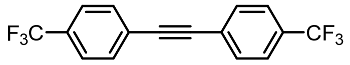

2e

1,2-Bis(4-(trifluoromethyl)phenyl)ethyne 2e: known compound.[8] From 1-iodo-4-(trifluoromethyl)benzene (0.80 mmol). Off-white solid (117 mg, 93%) **^{1}H NMR** (300 MHz, $CDCl_3$), δ (ppm): ^{1}H NMR (300 MHz, $CDCl_3$) δ 7.70 – 7.58 (m, 8H).

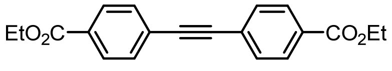

2f

Diethyl 4,4'-(ethyne-1,2-diyl)dibenzoate 2f: known compound.[9] From ethyl 4-bromobenzoate (10 mmol). White solid (740 mg, 46%). **^{1}H NMR** (300 MHz, $CDCl_3$) δ (ppm): ^{1}H NMR (300 MHz, $CDCl_3$) δ (ppm) 8.04 (d, J = 8.2 Hz, 4H), 7.60 (d, J = 8.2 Hz, 4H), 4.39 (q, J = 7.0 Hz, 4H), 1.41 (t, J = 7.1 Hz, 6H).

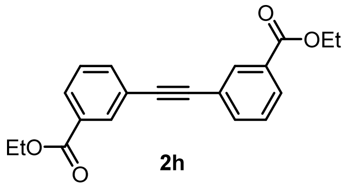

2h

Diethyl 3,3'-(ethyne-1,2-diyl)dibenzoate 2h: known compound.[10] From ethyl 3-bromobenzoate (10 mmol). White solid (1.12 g, 69%). **^{1}H NMR** (300 MHz, $CDCl_3$) δ (ppm): 8.22 (s, 2H), 8.02 (d, J = 7.8 Hz, 2H), 7.71 (d, J = 7.7 Hz, 2H), 7.44 (t, J = 7.7 Hz, 2H), 4.40 (q, J = 7.1 Hz, 4H), 1.42 (t, J = 7.1 Hz, 6H).

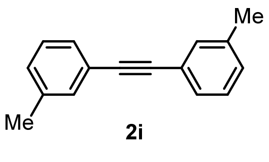

2i

1,2-Di-m-tolylethyne 2i: known compound.[11] From 1-iodo-3-methylbenzene (10 mmol). White solid (0.51 g, 50%). **^{1}H NMR** (300 MHz, $CDCl_3$) δ (ppm): 7.34 (d, J = 10.7 Hz, 4H), 7.28 – 7.20 (m, 2H), 7.14 (d, J = 7.3 Hz, 2H), 2.35 (s, 6H).

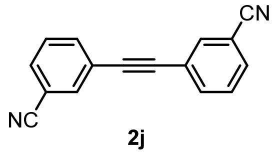

2j

3,3'-(Ethyne-1,2-diyl)dibenzonitrile 2j: known compound.[8] From 3-bromobenzonitrile (10 mmol). Brown solid (0.69 g, 61%). **^{1}H NMR** (300 MHz, $CDCl_3$) δ (ppm): 7.78 – 7.67 (m, 4H), 7.60 (d, J = 7.7 Hz, 2H), 7.46 (t, J = 7.6 Hz, 2H).

General Procedure: synthesis of alkynes through Sonogashira coupling

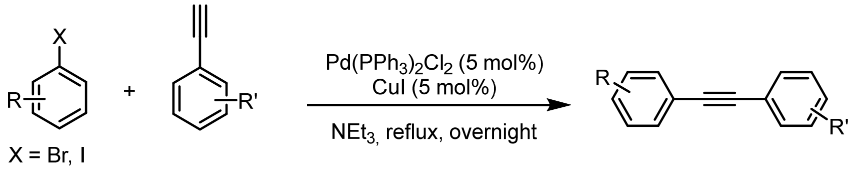

Following the reported procedure,[12] a flame-dried flask was charged with $PdCl_2(PPh_3)_2$ (0.050 equiv) and CuI (0.050 equiv). Then NEt_3 was added in order to form a 0.303 M solution. Argon was bubbled into the solution for 5 to 10 minutes and finally the aryl halide (1.5 equiv) and terminal alkyne (1 equiv) were added. The reaction was stirred under reflux. After completion of the reaction (TLC monitoring) the solvent was evaporated and the mixture was filtered through silica gel using EtOAc as eluent and the filtrate was evaporated to dryness. The residue was dissolved in EtOAc and washed with a saturated solution of NH_4Cl (10 mL) and brine (10 mL). The combined organic layers were dried over anhydrous $MgSO_4$, filtered and evaporated under vacuum. The residue

was purified by flash column chromatography through silica gel using a gradient from pure hexane to hexane: EtOAc (9:1) as eluent.

Deprotection of TMS-acetylenes

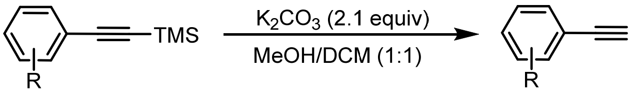

Following a reported procedure,[12] to a solution of alkyne (1 equiv) in MeOH/DCM (1:1, 0.26 M) was added solid K_2CO_3 (2.1 equiv). The reaction mixture was stirred at room temperature until disappearance of the starting material (TLC monitoring, 2h). The solvent was removed under reduced pressure and the residue was dissolved in EtOAc and washed twice with an aqueous solution of HCl 5% and once with brine. The combined organic layers were dried over anhydrous $MgSO_4$, filtered and evaporated to dryness. In all cases the crude product was used without further purification to carry out the Sonogashira coupling.

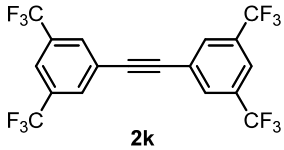

2k

1,2-Bis(3,5-bis(trifluoromethyl)phenyl)ethyne 2k: known compound.[13] From 1-bromo-3,5-bis(trifluoromethyl)benzene (5.13 mmol) and trimethylsilylacetylene (1.5 equiv). White solid (0.56 g, 24%). **^{1}H NMR** (300 MHz, $CDCl_3$) δ (ppm): 8.00 (s, 4H), 7.89 (s, 2H).

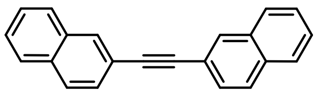

2l

1,2-Di(naphthalen-2-yl)ethyne 2l: known compound.[11] From 2-ethynylnaphthalene (2 mmol) and 2-bromonaphthalene (1.05 equiv). White solid (0.28 g, 50%). **^{1}H NMR** (300 MHz, $CDCl_3$) δ (ppm): 8.11 (s, 2H), 7.89 – 7.80 (m, 6H), 7.63 (d, J = 8.5 Hz, 2H), 7.57 – 7.45 (m, 4H).

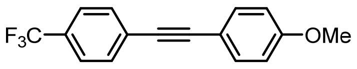

2m

1-Methoxy-4-((4-(trifluoromethyl)phenyl)ethynyl)benzene 2m: known compound.[14] From 1-ethynyl-4-(trifluoromethyl)benzene (5 mmol) and 4-iodoanisole (1.5 equiv). Yellow solid (0.96 g, 69%). **^{1}H NMR** (300 MHz, $CDCl_3$) δ (ppm): ^{1}H NMR (300 MHz, $cdcl_3$) δ 7.59 (s, 4H), 7.49 (d, J = 8.6 Hz, 2H), 6.90 (d, J = 8.5 Hz, 2H), 3.84 (s, 3H).

3. Optimization Data

A 5 mL reaction vial or a 5 mL sealed tube was equipped with a magnetic stir bar and was charged with the solid reagents. Then, the liquid reagents/solvents were added. In cases where an oxygen or air balloon were used as co-oxidants, the solution was saturated in oxygen or air with stirring for 10 min. Then, the reaction was heated in an oil bath for 24 h. 1,3,5-Trimethoxybenzene was used as Internal Standard.

Table S1. Catalyst optimization[a]

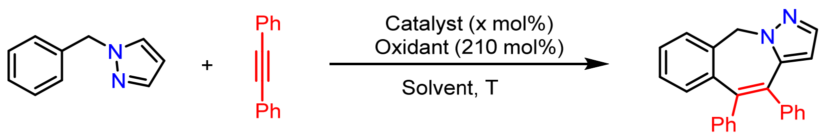

1a **2a** **3aa**

Entry	Catalyst (mol%)	Oxidant	Solvent	T (° C)	Yield (%)[b]
1	$[RhCp*Cl_2]_2$ (2.5)	AgOAc	Toluene	100	-
2	$[RhCp*Cl_2]_2$ (2.5)	AgOAc	*m*-xylene	150	-
3	$[RhCp*Cl_2]_2$ (2.5)	$Cu(OAc)_2 \cdot H_2O$/ Na_2CO_3	*m*-xylene	150	-
4	$Pd(OAc)_2$ (5)	$Cu(OAc)_2$	MeCN	105	20
5	$Pd(OAc)_2$ (5)	$Cu(OAc)_2$	DMF	120	50
6	$Pd(OAc)_2$ (10)	$Cu(OAc)_2$ / BQ (30 mol%)	DMF	120	54

[a]Conditions: **1a** (0.2 mmol), **2a** (0.3 mmol), solvent (2 mL). [b]Determined by ^{1}H NMR

Table S2. Oxidant optimization[a]

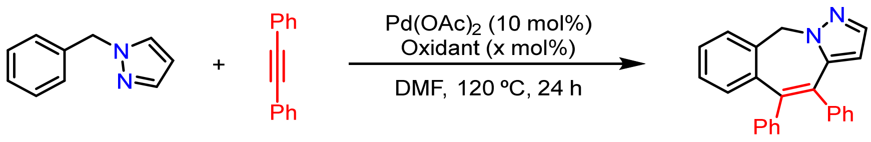

1a **2a** **3aa**

Entry	Oxidant (mol %)	Yield (%)[b]
1	$Cu(OAc)_2$ (300 mol%)	45
2	$Cu(OAc)_2$ (105 mol%) / O_2 balloon	49
3	$Cu(OAc)_2$ (75 mol%) / O_2 balloon	50
4	$Cu(OAc)_2$ (50 mol%) / O_2 balloon	30
5	O_2 balloon	20
6	O_2 balloon/ NaOAc (2.1 equiv)	16
7	Ag_2CO_3 (210 mol%)	49
8	AgOPiv (210 mol%)	28
9	Ag_2O / NaOAc (210 mol%)	6
10	AgOAc (210 mol%)	64
11	AgOAc (210 mol%) / BQ (30 mol%)	44
12	BQ (210 mol%)	Traces
13	BQ (210 mol%)/ HOAc (210 mol%)	33
14	AgOAc (150 mol%)	47
15	AgOAc (300 mol%)	74
16	AgOAc (400 mol%)	60

[a] Typical conditions: **1a** (0.2 mmol), **2a** (0.3 mmol), DMF (2 mL). [b] Determined by ^{1}H NMR. [c]Isolated yield.

Table S3. Temperature optimization[a]

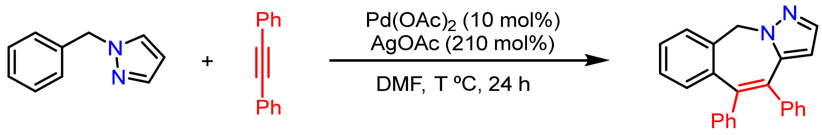

1a **2a** **3aa**

Entry	Temperature (° C)	Yield (%)[b]
1	150	Decomposition
2	120	64
3	80	47
4	70	27
5[c]	90	49

[a]Conditions: **1a** (0.2 mmol), **2a** (0.3 mmol), DMF (2 mL). [b] Determined by ^{1}H NMR. [c] 210 mol% of $Cu(OAc)_2$ instead of AgOAc.

Table S4. Concentration optimization[a]

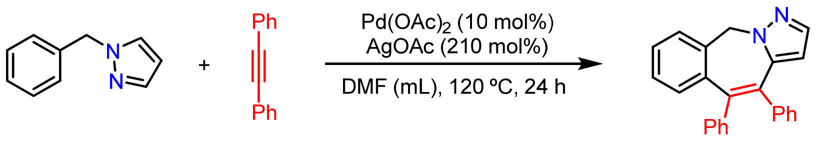

Entry	DMF volume (mL)	Yield (%)[b]
1	4	52
2	2	64
3	0,8	51
4	0,4	53

[a]Conditions: **1a** (0.2 mmol), **2a** (0.3 mmol). [b] Determined by ^{1}H NMR.

Table S5. Alkyne optimization[a]

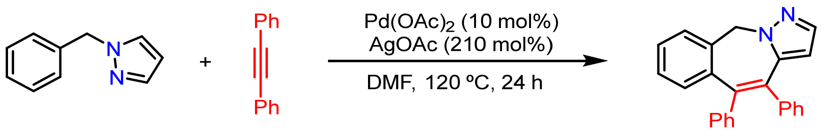

Entry	equiv	Yield (%)[b]
1	1	50
2	1.2	58
3	1.5	64
4	2	50
5	3	43

[a]Conditions: **1a** (0.2 mmol), **2a**, DMF (2 mL). [b] Determined by ^{1}H NMR.

Table S6. Acid additive optimization[a]

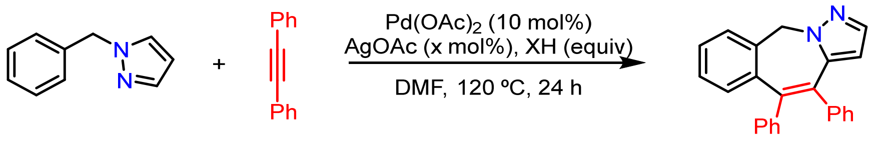

Entry	AgOAc (mol %), XH (equiv)	Yield (%)[b]
1	AgOAc (300)/ PivOH (1)	68
2	AgOAc (210)/ AcOH (1)	69
3	AgOAc (210)/ PivOH (1)	75
4	AgOAc (150)/ PivOH (10)	84

5	AgOAc (100)/ PivOH (10)	48
6	AgOAc (150)/ PivOH (15)	52
7	AgOAc (100)/ PivOH (15)	36
8	AgOAc (210)/ PivOH (10)	88
**9**	**AgOAc (210)/ PivOH (5)**	**88 (80)[c]**
10	Without $Pd(OAc)_2$/ AgOAc (210)	SM (98)

[a] SM = Starting Material. Typical conditions: **1a** (0.2 mmol), **2a** (0.3 mmol), DMF (2 mL). [b] Determined by ^{1}H NMR. [c]Isolated yield.

Table S7. Solvent optimization[a]

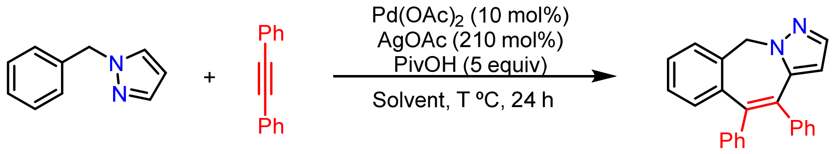

1a **2a** **3aa**

Entry	Solvent	Temperature (° C)	Yield (%)[b]
1	DMF	120	88 (80)
2	DMF	90	77 (73)
3	Toluene	120	56
4	Chlorobenzene	120	58
5	DCE	100	54
6	MeCN	100	58
7	Dioxane	120	62
8	Methanol	90	Traces
9	AcOH	100	50
10	DCM	60	15
11	HFIP	90	---
12	NMP	120	57
13	^{t}AmylOH	120	25

[a] Typical conditions: **1a** (0.2 mmol), **2a** (0.3 mmol), Solvent (2 mL). [b] Determined by ^{1}H NMR. Isolated yield in parenthesis

Table S8. Monoprotected aminoacid ligands[a]

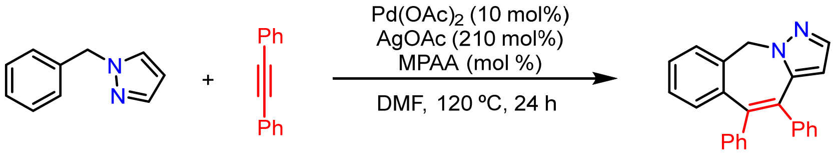

1a **2a** **3aa**

MPAA

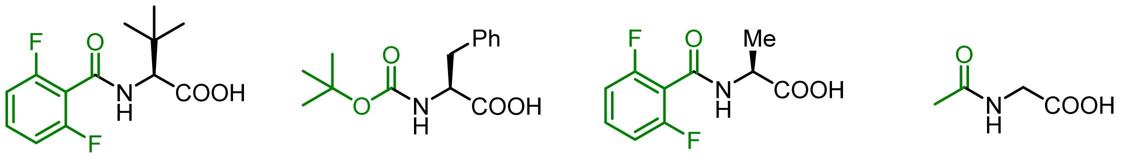

**1**	**2**	**3**	**4**
2,6-F.F-Bz-tert-Leu-OH	Boc-PheAla-OH	2,6-F,F-Bz-Ala-OH	N-AcetylGlycine

Entry	MPAA	Base	Yield (%)[b]
1	**1** (15 mol%)	K_2CO_3 (15 mol%)	60
2	**2** (15 mol%)	K_2CO_3 (15 mol%)	56
3	**3** (15 mol%)	K_2CO_3 (15 mol%)	60
4	**1** (15 mol%)	Cs_2CO_3 (15 mol%)	51
5	**2** (15 mol%)	Cs_2CO_3 (15 mol%)	53
6	**1** (40 mol%)	Cs_2CO_3 (150 mol%)	---
**7**	**2** (40 mol%)	Cs_2CO_3 (150 mol%)	---
8	**4** (15 mol%)	Cs_2CO_3 (150 mol%)	---
9	**1** (40 mol%)	Cs_2CO_3 (40 mol%)	32
10	**2** (40 mol%)	Cs_2CO_3 (40 mol%)	23
11	**4** **(**40 mol%)	Cs_2CO_3 (40 mol%)	25
12	**1** (40 mol%)	---	58
13	**4** (40 mol%)	---	49

[a]Conditions: **1a** (0.2 mmol), **2a** (0.3 mmol), DMF (2 mL). [b] Determined by ^{1}H NMR.

Table S9. Pd^{II} to Pd^{IV} oxidation

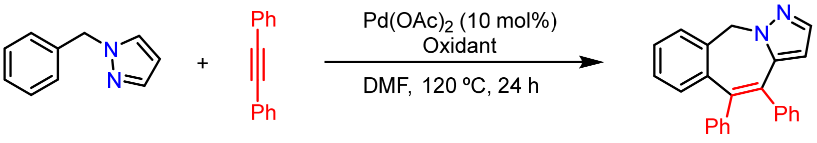

1a **2a** **3aa**

Entry	Oxidant (mol %)	Yield (%)[b]
1	PIFA (2 equiv)	---
2	$PhI(OAc)_2$ (2 equiv)	Traces
3	$PhI(OAc)_2$ (2 equiv)/ PivOH (0.5 equiv)	---
4	Oxone (2 equiv)	---
5	$PhI(OAc)_2$ (2 equiv)/ KPF_6 (1.2 equiv)	Traces
6	NFSI (2.1 equiv)/ PivOH (5 equiv)	---
7	$Pd(OAc)_2$ (100 mol%)/ PivOH (5 equiv)/ no oxidant	90%

[a]Conditions: **1a** (0.2 mmol), **2a** (0.3 mmol), DMF (2 mL). [b]Determined by ^{1}H NMR

4. Palladium-Catalyzed [5+2] Rollover Annulation of 1-Benzylpyrazoles 1 with Alkynes 2

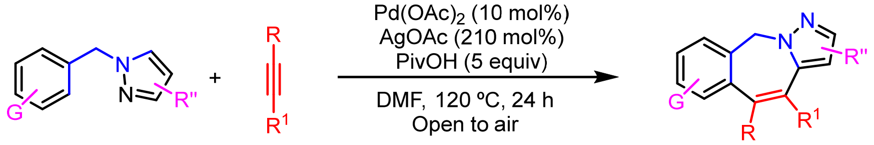

1 **2** (1.5 equiv) **3**

4,5-Diphenyl-10H-benzo[e]pyrazolo[1,5-a]azepine (3aa)

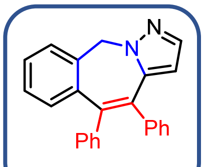

A 5 mL vial equipped with a magnetic stir bar was charged with **1a** (31.6 mg, 0.2 mmol, 1 equiv), **2a** (53.5 mg, 0.3 mmol, 1.5 equiv), $Pd(OAc)_2$ (4.5 mg, 0.02 mmol, 0.1 equiv) and AgOAc (70.1 mg, 0.42 mmol, 2.1 equiv). Then a solution of pivalic acid (102.1 mg, 1 mmol, 5 equiv) in 2 mL of DMF was added. The resulting solution was heated at 120 ºC in an oil bath for 24 h. The reaction mixture was extracted with $NaHCO_{3(aq)}$ (3 x 20 mL). The organic layer was dried over $MgSO_4$ and concentrated *in vacuo*. The product was purified by column chromatography on silica gel using Hexane/EtOAc (8:2) as eluent to afford **3aa** as an orange solid in 80% yield (53.5 mg, 0.160 mmol). mp: 224 – 226 ºC. **^{1}H NMR** (500 MHz, $CDCl_3$) δ (ppm): ^{1}H NMR (500 MHz, $CDCl_3$) δ (ppm): 7.50 (d, J = 7.6 Hz, 1H), 7.40 – 7.36 (m, 1H), 7.35 (dd, J = 7.5, 1.3 Hz, 1H), 7.24 – 7.19 (m, 5H), 7.19 – 7.12 (m, 4H), 7.10 – 7.05 (m, 3H), 6.21 (s, 1H), 5.47 (s, 2H). **^{13}C NMR** (126 MHz, $CDCl_3$) δ (ppm): 141.8 (C), 141.2 (C), 141.1 (C), 139.1 (C), 138.2 (CH), 135.4 (C), 132.5 (C), 131.0 (CH), 130.9 (2xCH), 129.4 (2xCH), 128.7 (CH), 127.9, (2xCH) 127.83 (CH), 127.80 (CH), 127.6 (2xCH), 126.71 (CH), 126.66 (CH), 107.2 (C), 54.9 (CH_2). **HRMS (APCI-FIA-TOF)** m/z: $[M+H]^+$ Calcd for $C_{24}H_{19}N_2$ 335.1543; found 335.1533.

Scale-up preparation of 3aa

A 100 mL round bottom flask equipped with a magnetic stir bar was charged with **1a** (0.47 g, 3 mmol, 1 equiv), **2a** (0.80 g, 4.5 mmol, 1.5 equiv), $Pd(OAc)_2$ (33.7 mg, 0.15 mmol, 0.050 equiv), AgOAc (1.05 g, 6.3 mmol, 2.1 equiv) and 25 mL of DMF. Then a solution of pivalic acid (1.53 g, 15 mmol, 5 equiv) in 5 mL of DMF was added. The resulting solution was heated at 120 ºC in an oil bath for 24 h. The reaction mixture was extracted with $NaHCO_{3(aq)}$ (5 x 50 mL). The organic layer was dried over $MgSO_4$ and concentrated in vacuo. The product was purified by column chromatography on silica gel using Hexane/EtOAc (8:2) as eluent to afford **3aa** as an orange solid in 68% yield (0.69 g, 2.05 mmol).

8-Methyl-4,5-diphenyl-10H-benzo[e]pyrazolo[1,5-a]azepine

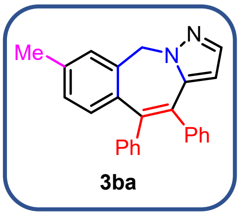

A 5 mL vial equipped with a magnetic stir bar was charged with **1b** (32.0 mg, 0.186 mmol, 1 equiv), **2a** (49.7 mg, 0.279 mmol, 1.5 equiv), $Pd(OAc)_2$ (4.2 mg, 0.019 mmol, 0.1 equiv) and AgOAc (65.2 mg, 0.39 mmol, 2.1 equiv). Then a solution of pivalic acid (94.9 mg, 0.929 mmol, 5 equiv) in 1.9 mL of DMF was added. The resulting solution was heated at 120 ºC in an oil bath for 24 h. The reaction mixture was extracted with $NaHCO_{3(aq)}$ (3 x 20 mL). The organic layer was dried over $MgSO_4$ and concentrated *in vacuo*. The product was purified by column chromatography on silica gel using Hexane/EtOAc (8:2) as eluent to afford **3ba** as a yellow solid in 68% yield (43.9 mg, 0.126 mmol). mp: 164 – 166 ºC. **^{1}H NMR** (500 MHz, $CDCl_3$) δ (ppm): 7.34 (d, *J* = 2.0 Hz, 1H), 7.28 (d, *J* = 1.8 Hz, 1H), 7.20 – 7.16 (m, 4H), 7.15 – 7.09 (m, 4H), 7.07 – 7.04 (m, 2H), 7.00 (dd, *J* = 8.0, 1.8 Hz, 1H), 6.91 (d, *J* = 8.0 Hz, 1H), 6.16 (d, *J* = 1.9 Hz, 1H), 5.40 (s, 2H), 2.36 (s, 3H). **^{13}C NMR** (126 MHz, $CDCl_3$) δ (ppm): 142.1 (C), 141.3 (C), 141.3 (C), 140.7 (C), 139.0 (C), 138.2 (CH), 136.4 (C), 135.5 (C), 131.8 (C), 131.1 (CH), 131.0 (2xCH), 129.6 (2xCH), 128.7 (CH), 128.5 (CH), 128.0 (2xCH), 127.6 (2xCH), 126.73 (CH), 126.69 (CH), 107.0 (CH), 55.1 (CH_2), 21.1 (CH_3). **HRMS (APCI-FIA-TOF)** m/z: $[M+H]^+$ Calcd for $C_{25}H_{21}N_2$ 349.1699; found 349.1701.

8-Methoxy-4,5-diphenyl-10H-benzo[e]pyrazolo[1,5-a]azepine

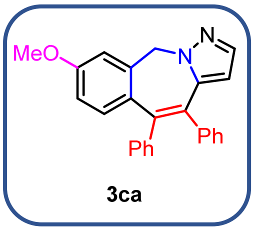

A 5 mL vial equipped with a magnetic stir bar was charged with **1c** (37.6 mg, 0.2 mmol, 1 equiv), **2a** (53.5 mg, 0.3 mmol, 1.5 equiv), $Pd(OAc)_2$ (4.5 mg, 0.02 mmol, 0.1 equiv) and AgOAc (70.1 mg, 0.42 mmol, 2.1 equiv). Then a solution of pivalic acid (102.1 mg, 1.0 mmol, 5 equiv) in 2 mL of DMF was added. The resulting solution was heated at 120 ºC in an oil bath for 24 h. The reaction mixture was extracted with $NaHCO_{3(aq)}$ (3 x 20 mL). The organic layer was dried over $MgSO_4$ and concentrated *in vacuo*. The product was purified by column chromatography on silica gel using Hexane/EtOAc (8:2) as eluent to afford **3ca** as a yellow solid in 55% yield (40.4 mg, 0.111 mmol). mp: 163– 165 ºC. **^{1}H NMR** (500 MHz, $CDCl_3$) δ (ppm): 7.35 (d, *J* = 2.0 Hz, 1H), 7.20 – 7.15 (m, 4H), 7.15 – 7.07 (m, 4H), 7.06 – 7.02 (m, 2H), 6.99 (d, *J* = 2.7 Hz, 1H), 6.93 (d, *J* = 8.8 Hz, 1H), 6.72 (dd, *J* = 8.8, 2.7 Hz, 1H), 6.16 (d, *J* = 2.1 Hz, 1H), 5.39 (s, 2H), 3.82 (s, 3H).**^{13}C NMR** (126 MHz, $CDCl_3$) δ (ppm): 160.1 (C), 142.1 (C), 141.3 (C), 141.0 (C), 140.8 (C), 138.3 (CH), 136.9 (C), 132.6 (CH), 131.8 (C), 131.0 (2xCH), 130.9 (C), 129.7 (2xCH), 128.0 (2xCH), 127.6 (2xCH), 126.7 (2xCH), 114.0 (CH), 112.6 (CH), 106.8 (CH), 55.5 (CH_3), 55.1 (CH_2). **HRMS (APCI-FIA-TOF)** m/z: $[M+H]^+$ Calcd for $C_{25}H_{21}N_2O$ 365.1648; found 365.1650.

8-Chloro-4,5-diphenyl-10H-benzo[e]pyrazolo[1,5-a]azepine

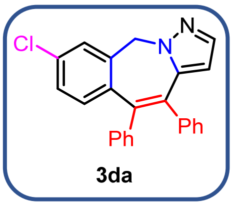

A 5 mL vial equipped with a magnetic stir bar was charged with **1d** (38.5 mg, 0.2 mmol, 1 equiv), **2a** (53.5 mg, 0.3 mmol, 1.5 equiv), $Pd(OAc)_2$ (4.5 mg, 0.02 mmol, 0.1 equiv) and AgOAc (70.1 mg, 0.42 mmol, 2.1 equiv). Then a solution of pivalic acid (306.4 mg, 3 mmol, 15 equiv) in 2 mL of DMF was added. The resulting solution was heated at 120 ºC in an oil bath for 24 h. The reaction mixture was extracted with $NaHCO_{3(aq)}$ (3 x 20 mL). The organic layer was dried over $MgSO_4$ and concentrated *in vacuo*. The product was purified by column chromatography on silica gel using Hexane/AcOEt (9:1) as eluent to afford **3da** as yellow crystals in 50% yield (36.7 mg, 0.099 mmol). mp: 155 – 157 ºC. **^{1}H NMR** (500 MHz, $CDCl_3$) δ (ppm): 7.47 (d, *J* = 2.3 Hz, 1H), 7.37 (d, *J* = 2.0 Hz, 1H), 7.22 – 7.17 (m, 4H), 7.15 (dt, *J* = 8.6, 2.2 Hz, 3H), 7.13 – 7.09 (m, 2H), 7.06 – 7.00 (m, 2H), 6.95 (d, *J* = 8.5 Hz, 1H), 6.19 (d, *J* = 2.0 Hz, 1H), 5.41 (s, 2H). **^{13}C NMR** (126 MHz, $CDCl_3$) δ (ppm): 141.5 (C), 140.9 (C), 140.6 (C), 140.4 (C), 138.4 (CH), 137.7 (C), 136.8 (C), 134.7 (C), 132.9 (C), 132.5 (CH), 131.0 (2xCH), 129.4 (2xCH), 128.1 (3xCH), 127.9 (CH), 127.8 (2xCH), 127.02 (CH), 126.98 (CH), 107.3 (CH), 54.4 (CH_2). **HRMS (APCI-FIA-TOF)** m/z: $[M+H]^+$ Calcd for $C_{24}H_{18}ClN_2$ 369.1153; found 369.1151.

Methyl 4,5-diphenyl-10H-benzo[e]pyrazolo[1,5-a]azepine-8-carboxylate

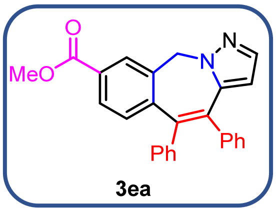

A 5 mL vial equipped with a magnetic stir bar was charged with **1e** (46.0 mg, 0.23 mmol, 1 equiv), **2a** (61.4 mg, 0.345 mmol, 1.5 equiv), $Pd(OAc)_2$ (5.2 mg, 0.023 mmol, 0.1 equiv) and AgOAc (80.5 mg, 0.482 mmol, 2.1 equiv). Then a solution of pivalic acid (117.3 mg, 1.149 mmol, 5 equiv) in 2.3 mL of DMF was added. The resulting solution was heated at 120 ºC in an oil bath for 24 h. The reaction mixture was extracted with $NaHCO_{3(aq)}$ (3 x 20 mL). The organic layer was dried over $MgSO_4$ and concentrated *in vacuo*. The product was purified by column chromatography on silica gel using Hexane/EtOAc (8:2) as eluent to afford **3ea** as an amorphous yellow solid in 42% yield (32.7 mg, 0.083 mmol). **^{1}H NMR** (500 MHz, $CDCl_3$) δ (ppm): 8.15 (s, 1H), 7.83 (d, *J* = 8.3 Hz, 1H), 7.36 (m, 1H), 7.22 – 7.08 (m, 9H), 7.06 – 7.00 (m, 2H), 6.18 (d, *J* = 2.1 Hz, 1H), 5.49 (s, 2H), 3.91 (s, 3H). **^{13}C NMR** (126 MHz, $CDCl_3$) δ (ppm): 166.5 (CO), 143.6 (C), 141.4 (C), 140.8 (C), 140.40 (C), 140.36 (C), 138.5 (CH), 135.6 (C), 134.2 (C), 131.3(CH), 131.0 (2xCH), 130.1 (C), 129.3 (2xCH), 129.2 (CH), 128.9 (CH), 128.1 (2xCH), 127.8 (2xCH), 127.1 (CH), 127.0 (CH), 107.6 (CH), 54.8 (CH_2), 52.4 (CH_3). **HRMS (APCI-FIA-TOF)** m/z: $[M+H]^+$ Calcd for $C_{26}H_{21}N_2O_2$ 393.1598; found 393.1607.

7-Methyl-4,5-diphenyl-10H-benzo[e]pyrazolo[1,5-a]azepine

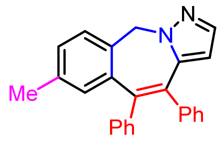

3fa

A 5 mL vial equipped with a magnetic stir bar was charged with **1f** (34.4 mg, 0.2 mmol, 1 equiv), **2a** (53.5 mg, 0.3 mmol, 1.5 equiv), $Pd(OAc)_2$ (4.5 mg, 0.02 mmol, 0.1 equiv) and AgOAc (70.1 mg, 0.42 mmol, 2.1 equiv). Then a solution of pivalic acid (102.1 mg, 1.0 mmol, 5 equiv) in 2 mL of DMF was added. The resulting solution was heated at 120 ºC in an oil bath for 24 h. The reaction mixture was extracted with $NaHCO_{3(aq)}$ (3 x 20 mL). The organic layer was dried over $MgSO_4$ and concentrated *in vacuo*. The product was purified by column chromatography on silica gel using Hexane/EtOAc (8:2) as eluent to afford **3fa** as a yellow solid in 70% yield (48.9 mg, 0.140 mmol). mp: 186 – 188 ºC. **^{1}H NMR** (500 MHz, $CDCl_3$) δ (ppm): 7.37 – 7.32 (m, 2H), 7.20 – 7.17 (m, 4H), 7.17 – 7.09 (m, 5H), 7.06 (d, J = 7.8 Hz, 2H), 6.84 (s, 1H), 6.15 (s, 1H), 5.41 (s, 2H), 2.19 (s, 3H). **^{13}C NMR** (126 MHz, $CDCl_3$) δ (ppm): 141.9 (C), 141.32 (C), 141.30 (C), 140.5 (C), 139.0 (C), 138.1 (CH), 137.6 (C), 132.9 (C), 132.5 (C), 131.5 (CH), 131.0 (2xCH), 129.6 (CH), 129.5 (2xCH), 128.0 (2xCH), 127.8 (CH), 127.6 (2xCH), 126.73 (CH), 126.70 (CH), 107.1 (CH), 54.7 (CH_2), 21.3 (CH_3). **HRMS (APCI-FIA-TOF)** m/z: $[M+H]^+$ Calcd for $C_{25}H_{21}N_2$ 349.1699; found 349.1703.

7-Bromo-4,5-diphenyl-10H-benzo[e]pyrazolo[1,5-a]azepine

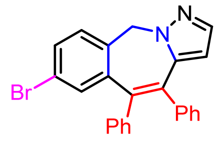

3ga

A 5 mL vial equipped with a magnetic stir bar was charged with **1g** (47.4 mg, 0.2 mmol, 1 equiv), **2a** (53.5 mg, 0.3 mmol, 1.5 equiv), $Pd(OAc)_2$ (4.5 mg, 0.02 mmol, 0.1 equiv) and AgOAc (70.1 mg, 0.42 mmol, 2.1 equiv). Then a solution of pivalic acid (102.1 mg, 1.0 mmol, 5 equiv) in 2 mL of DMF was added. The resulting solution was heated at 120 ºC in an oil bath for 24 h. The reaction mixture was extracted with $NaHCO_{3(aq)}$ (3 x 20 mL). The organic layer was dried over $MgSO_4$ and concentrated *in vacuo*. The product was purified by column chromatography on silica gel using Hexane/EtOAc (85:15) as eluent to afford **3ga** as an amorphous orange solid in 40% yield (32.8 mg, 0.080 mmol). **^{1}H NMR** (500 MHz, $CDCl_3$) δ (ppm): 7.45 (dd, J = 8.2, 2.0 Hz, 1H), 7.35 – 7.32 (m, 2H), 7.21 – 7.11 (m, 9H), 7.03 (m, 2H), 6.16 (d, J = 2.0 Hz, 1H), 5.39 (s, 2H). **^{13}C NMR** (126 MHz, $CDCl_3$) δ (ppm): 141.2 (C), 141.1 (C), 140.9 (C), 140.3 (C), 139.9 (C), 138.4 (CH), 134.3 (C), 133.8 (C), 133.7 (CH), 131.8 (CH), 131.0 (2xCH), 129.5 (CH), 129.4 (2xCH), 128.1 (2xCH), 127.9 (2xCH), 127.2 (CH), 127.0 (CH), 122.0 (C), 107.4 (CH), 54.3 (CH_2). **HRMS (APCI-FIA-TOF)** m/z: $[M+H]^+$ Calcd for $C_{24}H_{18}BrN_2$ 413.0648; found 413.0648

7-Chloro-4,5-diphenyl-10H-benzo[e]pyrazolo[1,5-a]azepine

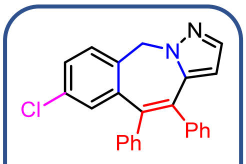

3ha

A 5 mL vial equipped with a magnetic stir bar was charged with **1h** (38.5 mg, 0.2 mmol, 1 equiv), **2a** (53.5 mg, 0.3 mmol, 1.5 equiv), $Pd(OAc)_2$ (4.5 mg, 0.02 mmol, 0.1 equiv) and AgOAc (70.1 mg, 0.42 mmol, 2.1 equiv). Then a solution of pivalic acid (102.1 mg, 1.0 mmol, 5 equiv) in 2 mL of DMF was added. The resulting solution was heated at 120 ºC in an oil bath for 24 h. The reaction mixture was extracted with $NaHCO_{3(aq)}$ (3 x 20 mL). The organic layer was dried over $MgSO_4$ and concentrated *in vacuo*. The product was purified by column chromatography on silica gel using Hexane/EtOAc (8:2) as eluent to afford **3ha** as an amorphous yellow solid in 54% yield (38.84 mg, 0.108 mmol). **^{1}H NMR** (500 MHz, $CDCl_3$) δ (ppm): 7.41 (d, *J* = 8.2 Hz, 1H), 7.36 (d, *J* = 2.0 Hz, 1H), 7.30 (dd, *J* = 8.1, 2.1 Hz, 1H), 7.21 – 7.10 (m, 8H), 7.05 – 7.02 (m, 2H), 7.01 (d, *J* = 2.1 Hz, 1H), 6.17 (d, *J* = 2.0 Hz, 1H), 5.42 (s, 2H). **^{13}C NMR** (126 MHz, $CDCl_3$) δ (ppm): 141.1 (C), 140.9 (C), 140.8 (C), 140.4 (C), 140.3 (C), 138.2 (CH), 134.0 (C), 133.8 (C), 133.6 (C), 130.9 (2xCH), 130.9 (CH), 129.4 (2xCH), 129.3 (CH), 128.9 (CH), 128.1 (2xCH), 127.9 (2xCH), 127.2 (CH), 127.0 (CH), 107.5 (CH), 54.2 (CH_2). **HRMS (APCI-FIA-TOF)** m/z: $[M+H]^+$ Calcd for $C_{24}H_{18}ClN_2$ 369.1153; found 369.1148.

4,5-Diphenyl-7-(trifluoromethyl)-10H-benzo[e]pyrazolo[1,5-a]azepine

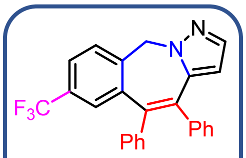

3ia

A 5 mL vial equipped with a magnetic stir bar was charged with **1i** (45.2 mg, 0.2 mmol, 1 equiv), **2a** (53.5 mg, 0.3 mmol, 1.5 equiv), $Pd(OAc)_2$ (4.5 mg, 0.02 mmol, 0.1 equiv) and AgOAc (70.1 mg, 0.42 mmol, 2.1 equiv). Then a solution of pivalic acid (102.1 mg, 1.0 mmol, 5 equiv) in 2 mL of DMF was added. The resulting solution was heated at 120 ºC in an oil bath for 24 h. The reaction mixture was extracted with $NaHCO_{3(aq)}$ (3 x 20 mL). The organic layer was dried over $MgSO_4$ and concentrated *in vacuo*. The product was purified by column chromatography on silica gel using Hexane/EtOAc (8:2) as eluent to afford **3ia** as yellow crystals in 46% yield (36.9 mg, 0.092 mmol). mp: 212-214 ºC. **^{1}H NMR** (500 MHz, $CDCl_3$) δ 7.65 – 7.57 (m, 2H), 7.40 (s, 1H), 7.29 (s, 1H), 7.24 – 7.13 (m, 8H), 7.08 – 7.00 (m, 2H), 6.22 (d, *J* = 1.6 Hz, 1H), 5.54 (s, 2H). **^{13}C NMR** 126 MHz, $CDCl_3$) δ (ppm): 140.8 (C), 140.7 (C), 140.5 (C), 139.9 (C), 138.4 (C), 138.0 (CH), 133.7 (C), 130.9 (2xCH), 130.5 (C) (d, *J* = 32.7 Hz), 130.3 (C), 129.3 (2xCH), 128.7 (CH), 128.2 (2xCH), 128.0 (2xCH), 127.9 (CH) (q, *J* = 3.6 Hz), 127.4 (CH), 127.2 (CH), 125.6 (CH) (q, *J* = 3.6 Hz), 123.8 (C), (d, *J* = 272.5 Hz), 107.6 (CH), 54.3 (CH_2). ^{19}F NMR (471 MHz, $CDCl_3$) δ (ppm): -62.7. **HRMS (APCI-FIA-TOF)** m/z: $[M+H]^+$ Calcd for $C_{25}H_{18}F_3N_2$ 403.1417; found 403.1417.

7-Methoxy-4,5-diphenyl-10H-benzo[e]pyrazolo[1,5-a]azepine

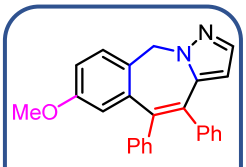

3ja

A 5 mL vial equipped with a magnetic stir bar was charged with **1j** (37.6 mg, 0.2 mmol, 1 equiv), **2a** (53.5 mg, 0.3 mmol, 1.5 equiv), $Pd(OAc)_2$ (4.5 mg, 0.02 mmol, 0.1 equiv) and AgOAc (70.1 mg, 0.42 mmol, 2.1 equiv). Then a solution of pivalic acid (102.1 mg, 1.0 mmol, 5 equiv) in 2 mL of DMF was added. The resulting solution was heated at 120 ºC in an oil bath for 24 h. The reaction mixture was extracted with $NaHCO_{3(aq)}$ (3 x 20 mL). The organic layer was dried over $MgSO_4$ and concentrated *in vacuo*. The product was purified by column chromatography on silica gel using Hexane/EtOAc (8:2) as eluent to afford **3ja** as a yellow solid in 58% yield (42.3 mg, 0.116 mmol). mp: 168 – 170 ºC. **^{1}H NMR** (500 MHz, $CDCl_3$) δ (ppm): 7.39 (d, J = 8.4 Hz, 1H), 7.34 (d, J = 2.1 Hz, 1H), 7.22 – 7.16 (m, 4H), 7.15 – 7.04 (m, 6H), 6.87 (dd, J = 8.4, 2.7 Hz, 1H), 6.56 (d, J = 2.7 Hz, 1H), 6.15 (d, J = 2.0 Hz, 1H), 5.41 (s, 2H), 3.62 (s, 3H). **^{13}C NMR** (126 MHz, $CDCl_3$) δ (ppm): 159.0 (C), 141.7 (C), 141.22 (C), 141.17 (C), 140.5 (C), 140.4 (C), 137.9 (CH), 132.6 (C), 130.9 (2xCH), 129.5 (2xCH), 129.1 (CH), 128.5 (C), 128.0 (2xCH), 127.7 (2xCH), 126.8 (2xCH), 116.8 (CH), 114.0 (CH), 107.2 (CH), 55.3 (CH_3), 54.3 (CH_2). **HRMS (APCI-FIA-TOF)** m/z: $[M+H]^+$ Calcd for $C_{25}H_{21}N_2O$ 365.1648; found 365.1650.

7,8-Dimethoxy-4,5-diphenyl-10H-benzo[e]pyrazolo[1,5-a]azepine

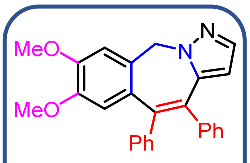

3ka

A 5 mL vial equipped with a magnetic stir bar was charged with **1k** (43.7 mg, 0.2 mmol, 1 equiv), **2a** (53.5 mg, 0.3 mmol, 1.5 equiv), $Pd(OAc)_2$ (4.5 mg, 0.02 mmol, 0.1 equiv) and AgOAc (70.1 mg, 0.42 mmol, 2.1 equiv). Then a solution of pivalic acid (102.1 mg, 1.0 mmol, 5 equiv) in 2 mL of DMF was added. The resulting solution was heated at 120 ºC in an oil bath for 24 h. The reaction mixture was extracted with $NaHCO_{3(aq)}$ (3 x 20 mL). The organic layer was dried over $MgSO_4$ and concentrated *in vacuo*. The product was purified by column chromatography on silica gel using Hexane/EtOAc (1:1) as eluent to afford **3ka** as a yellow solid in 64% yield (50.4 mg, 0.128 mmol). mp: 210 – 212 ºC. **^{1}H NMR** (500 MHz, $CDCl_3$) δ (ppm): 7.35 (d, J = 2.1 Hz, 1H), 7.20 – 7.16 (m, 4H), 7.15 – 7.11 (m, 3H), 7.11 – 7.08 (m, 1H), 7.07 (d, J = 1.7 Hz, 1H), 7.05 (t, J = 1.5 Hz, 1H), 6.96 (s, 1H), 6.47 (s, 1H), 6.17 (d, J = 2.0 Hz, 1H), 5.37 (s, 2H), 3.93 (s, 3H), 3.55 (s, 3H). **^{13}C NMR** (126 MHz, $CDCl_3$) δ (ppm): 149.6 (C), 148.1 (C), 141.9 (C), 141.3 (C), 141.2 (C), 140.7 (C), 138.0 (CH), 131.8 (C), 131.3 (C), 131.0 (2xCH), 129.6 (2xCH), 128.7 (C), 128.0 (2xCH), 127.7 (2xCH), 126.8 (CH), 126.7 (CH), 114.0 (CH), 110.5 (CH), 106.8 (CH), 56.1 (CH_3), 55.9 (CH_3), 54.5 (CH_2). **HRMS (APCI-FIA-TOF)** m/z: $[M+H]^+$ Calcd for $C_{26}H_{23}N_2O_2$ 395.1754; found 395.1758.

9-Methyl-4,5-diphenyl-10H-benzo[e]pyrazolo[1,5-a]azepine

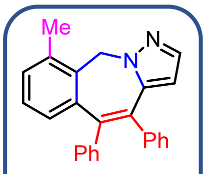

3la

A 5 mL vial equipped with a magnetic stir bar was charged with **1l** (34.4 mg, 0.2 mmol, 1 equiv), **2a** (53.5 mg, 0.3 mmol, 1.5 equiv), $Pd(OAc)_2$ (4.5 mg, 0.02 mmol, 0.1 equiv) and AgOAc (70.1 mg, 0.42 mmol, 2.1 equiv). Then a solution of pivalic acid (102.1 mg, 1.0 mmol, 5 equiv) in 2 mL of DMF was added. The resulting solution was heated at 120 ºC in an oil bath for 24 h. The reaction mixture was extracted with $NaHCO_{3(aq)}$ (3 x 20 mL). The organic layer was dried over $MgSO_4$ and concentrated *in vacuo*. The product was purified by column chromatography on silica gel using Hexane/EtOAc (8:2) as eluent to afford **3la** as a yellow solid in 72% yield (49.9 mg, 0.143 mmol). mp: 206– 208 ºC. **^{1}H NMR** (500 MHz, $CDCl_3$) δ (ppm): 7.35 (d, *J* = 1.8 Hz, 1H), 7.21 – 7.18 (m, 5H), 7.16 – 7.09 (m, 4H), 7.07 – 7.03 (m, 3H), 6.88 (d, *J* = 7.9 Hz, 1H), 6.18 (d, *J* = 1.8 Hz, 1H), 5.53 (s, 2H), 2.68 (s, 3H). **^{13}C NMR** (126 MHz, $CDCl_3$) δ (ppm): 142.2 (C), 141.8 (C), 141.2 (C), 140.7 (C), 139.9 (C), 138.1 (CH), 134.63 (C), 134.62 (C), 132.5 (C), 131.0 (2xCH), 130.5 (CH), 129.5 (2xCH), 129.0 (CH), 128.0 (2xCH), 127.6 (2xCH), 127.3 (CH), 126.8 (CH), 126.7 (CH), 106.4 (CH), 49.7 (CH_2), 20.0 (CH_3). **HRMS (APCI-FIA-TOF)** m/z: $[M+H]^+$ Calcd for $C_{25}H_{21}N_2$ 349.1699; found 349.1698.

9-Fluoro-4,5-diphenyl-10H-benzo[e]pyrazolo[1,5-a]azepine

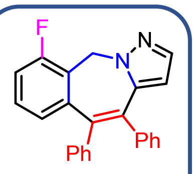

3ma

A 5 mL vial equipped with a magnetic stir bar was charged with **1m** (35.2 mg, 0.2 mmol, 1 equiv), **2a** (53.5 mg, 0.3 mmol, 1.5 equiv), $Pd(OAc)_2$ (4.5 mg, 0.02 mmol, 0.1 equiv) and AgOAc (70.1 mg, 0.42 mmol, 2.1 equiv). Then a solution of pivalic acid (102.1 mg, 1.0 mmol, 5 equiv) in 2 mL of DMF was added. The resulting solution was heated at 120 ºC in an oil bath for 24 h. The reaction mixture was extracted with $NaHCO_{3(aq)}$ (3 x 20 mL). The organic layer was dried over $MgSO_4$ and concentrated *in vacuo*. The product was purified by column chromatography on silica gel using Hexane/Et_2O (7:3) as eluent to afford **3ma** as a yellow solid in 65% yield (45.6 mg, 0.129 mmol). mp: 181 – 183 ºC. **^{1}H NMR** (500 MHz, $CDCl_3$) δ (ppm): 7.36 (d, *J* = 2.0 Hz, 1H), 7.23 – 7.16 (m, 4H), 7.16 – 7.07 (m, 6H), 7.07 – 7.03 (m, 2H), 6.81 (d, *J* = 7.8 Hz, 1H), 6.16 (d, *J* = 2.0 Hz, 1H), 5.59 (s, 2H). **^{13}C NMR** (126 MHz, $CDCl_3$) δ (ppm): 159.0 (C) (d, *J* = 247.0 Hz), 141.7 (C) (d, *J* = 2.7 Hz), 141.6 (C), 140.9 (C), 140.7 (C), 140.3 (C) (d, *J* = 2.7 Hz), 138.4 (CH), 133.4 (C), 131.0 (2xCH), 129.4 (2xCH), 128.60 (CH) (d, *J* = 8.7 Hz), 128.1 (2xCH), 127.8 (2xCH), 127.0 (2xCH), 126.8 (CH) (d, *J* = 3.6 Hz), 123.3 (C) (d, *J* = 16.8 Hz),115.0 (CH) (d, *J* = 22.7 Hz), 107.5 (CH), 45.7 d, *J* = 6.8 Hz) (CH_2). **^{19}F NMR** (282 MHz, $CDCl_3$) δ (ppm): -120.27 (t, *J* = 7.4 Hz). **HRMS (APCI-FIA-TOF)** m/z: $[M+H]^+$ Calcd for $C_{24}H_{18}FN_2$ 353.1449; found 353.1447.

2,3-Dimethyl-4,5-diphenyl-10H-benzo[e]pyrazolo[1,5-a]azepine

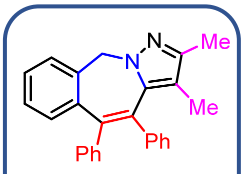

3na

A 5 mL vial equipped with a magnetic stir bar was charged with a mixture of **1n** and **1n'** (ratio 2.4:1), (37.3 mg, 0.2 mmol, 1 equiv), **2a** (53.5 mg, 0.3 mmol, 1.5 equiv), $Pd(OAc)_2$ (4.5 mg, 0.02 mmol, 0.1 equiv) and AgOAc (70.1 mg, 0.42 mmol, 2.1 equiv). Then a solution of pivalic acid (102.1 mg, 1.0 mmol, 5 equiv) in 2 mL of DMF was added. The resulting solution was heated at 120 ºC in an oil bath for 24 h. The reaction mixture was extracted with $NaHCO_{3(aq)}$ (3 x 20 mL). The organic layer was dried over $MgSO_4$ and concentrated *in vacuo*. The product was purified by column chromatography on silica gel using Hexane/EtOAc (8:2) as eluent to afford **3na** as an orange solid in 51% yield (26.0 mg, 0.072 mmol). mp: 218 – 220 ºC. **^{1}H NMR** (500 MHz, $CDCl_3$) δ (ppm) 7.42 (td, *J* = 7.8, 1.4 Hz, 2H), 7.28 (dd, *J* = 7.7, 1.4 Hz, 1H), 7.25 – 7.18 (m, 5H), 7.17 – 7.04 (m, 6H), 5.36 (s, 2H), 2.10 (s, 3H), 1.31 (s, 3H). **^{13}C NMR** (126 MHz, $CDCl_3$) δ (ppm): 145.9 (C), 142.3 (C), 141.3 (C), 139.6 (C), 138.5 (C), 138.1(C), 136.0 (C), 133.4 (C), 130.9 (2xCH), 130.5 (2xCH), 130.2 (CH), 128.6 (CH), 127.97 (CH), 127.95 (CH), 127.8 (2xCH), 127.5 (2xCH), 127.0 (CH), 126.8 (CH), 114.3 (C), 54.3 (CH_2), 11.5 (CH_3), 8.5 (CH_3). **HRMS (APCI-FIA-TOF)** m/z: $[M+H]^+$ Calcd for $C_{26}H_{23}N_2$ 363.1856; found 363.1860.

4,5-Diphenyl-12H-naphtho[2,3-e]pyrazolo[1,5-a]azepine

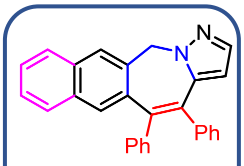

3oa

A 5 mL vial equipped with a magnetic stir bar was charged with **1o** (41.7 mg, 0.2 mmol, 1 equiv), **2a** (53.5 mg, 0.3 mmol, 1.5 equiv), $Pd(OAc)_2$ (4.5 mg, 0.02 mmol, 0.1 equiv) and AgOAc (70.1 mg, 0.42 mmol, 2.1 equiv). Then a solution of pivalic acid (102.1 mg, 1.0 mmol, 5 equiv) in 2 mL of DMF was added. The resulting solution was heated at 120 ºC in an oil bath for 24 h. The reaction mixture was extracted with $NaHCO_{3(aq)}$ (3 x 20 mL). The organic layer was dried over $MgSO_4$ and concentrated *in vacuo*. The product was purified by column chromatography on silica gel using Hexane/EtOAc (8:2) as eluent to afford **3oa** as a pale yellow solid in 63% yield (48.2 mg, 0.125 mmol). mp: 207 – 209 ºC. **^{1}H NMR** (500 MHz, $CDCl_3$) δ (ppm): 7.95 (s, 1H), 7.86 (d, *J* = 8.3 Hz, 1H), 7.61 (d, *J* = 8.3 Hz, 1H), 7.51 (s, 1H), 7.46 (ddd, *J* = 8.2, 6.8, 1.3 Hz, 1H), 7.40 (ddd, *J* = 8.2, 6.8, 1.3 Hz, 1H), 7.37 (d, *J* = 2.0 Hz, 1H), 7.24 – 7.12 (m, 10H), 6.18 (d, *J* = 2.0 Hz, 1H), 5.65 (s, 2H). **^{13}C NMR** (126 MHz, $CDCl_3$) δ (ppm): 142.3 (C), 141.5 (C), 141.3 (C), 140.6 (C), 138.2 (CH), 137.2 (C), 134.1 (C), 133.0 (C), 132.6 (C), 132.2 (C), 131.4 (CH), 131.2 (2xCH), 129.5 (2xCH), 128.4 (CH), 128.1 (2xCH), 127.7 (2xCH), 127.5 (CH), 127.0 (CH), 126.8 (2xCH), 126.60 (CH), 126.55 (CH), 107.3 (CH), 55.3 (CH_2). **HRMS (APCI-FIA-TOF)** m/z: $[M+H]^+$ Calcd for $C_{28}H_{21}N_2$ 385.1699; found 385.1702.

4,5,10-Triphenyl-10H-benzo[*e*]pyrazolo[1,5-a]azepine

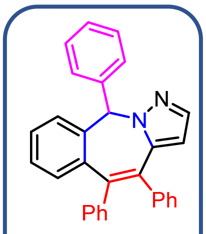

3pa

A 5 mL vial equipped with a magnetic stir bar was charged with **1p** (46.9 mg, 0.2 mmol, 1 equiv), **2a** (53.5 mg, 0.3 mmol, 1.5 equiv), $Pd(OAc)_2$ (4.5 mg, 0.02 mmol, 0.1 equiv) and AgOAc (70.1 mg, 0.42 mmol, 2.1 equiv). Then a solution of pivalic acid (102.1 mg, 1.0 mmol, 5 equiv) in 2 mL of DMF was added. The resulting solution was heated at 120 ºC in an oil bath for 24 h. The reaction mixture was extracted with $NaHCO_{3(aq)}$ (3 x 20 mL). The organic layer was dried over $MgSO_4$ and concentrated *in vacuo*. The product was purified by column chromatography on silica gel using Hexane/EtOAc (9:1) as eluent to afford **3pa** as pale yellow solid in 58% yield (47.5 mg, 0.116 mmol). mp: 200 – 202 ºC. **^{1}H NMR** (500 MHz, $CDCl_3$) δ (ppm): 7.53 (d, *J* = 7.7 Hz, 1H), 7.41 (d, *J* = 2.0 Hz, 1H), 7.36 (t, *J* = 6.7 Hz, 1H), 7.24 – 7.16 (m, 4H), 7.01 (d, *J* = 8.0 Hz, 1H), 6.99 – 6.85 (m, 7H), 6.71 – 6.65 (m, 2H), 6.63 – 6.51 (m, 4H), 6.17 (d, *J* = 2.0 Hz, 1H). **^{13}C NMR** (126 MHz, $CDCl_3$) δ (ppm): 141.8 (C), 141.1 (C), 140.7 (C), 140.6 (C), 140.3 (C), 138.4 (CH), 138.3 (C), 138.2 (C), 132.5 (CH), 131.1 (C), 130.5 (2xCH), 130.0 (CH), 129.2 (2xCH), 128.8 (CH), 128.3 (2xCH), 128.1 (CH), 127.8 (3xCH), 127.4 (2xCH), 126.6 (CH), 126.5 (CH), 126.2 (2xCH), 109.0 (CH), 70.1 (CH). **HRMS (APCI-FIA-TOF)** m/z: $[M+H]^+$ Calcd for $C_{30}H_{23}N_2$ 411.1856; found 411.1865.

4,5-Di-*p*-tolyl-10H-benzo[e]pyrazolo[1,5-a]azepine

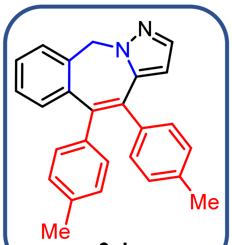

3ab

A 5 mL vial equipped with a magnetic stir bar was charged with **1a** (31.6 mg, 0.2 mmol, 1 equiv), **2b** (61.9 mg, 0.3 mmol, 1.5 equiv), $Pd(OAc)_2$ (4.5 mg, 0.02 mmol, 0.1 equiv) and AgOAc (70.1 mg, 0.42 mmol, 2.1 equiv). Then a solution of pivalic acid (102.1 mg, 1.0 mmol, 5 equiv) in 2 mL of DMF was added. The resulting solution was heated at 120 ºC in an oil bath for 24 h. The reaction mixture was extracted with $NaHCO_{3(aq)}$ (3 x 20 mL). The organic layer was dried over $MgSO_4$ and concentrated *in vacuo*. The product was purified by by column chromatography on silica gel using Hexane/EtOAc (8:2) as eluent to afford **3ab** as yellow solid in 64% yield (46.7 mg, 0.129 mmol). mp: 181 – 183 ºC. **^{1}H NMR** (500 MHz, $CDCl_3$) δ (ppm): 7.45 (dd, *J* = 7.7, 1.4 Hz, 1H), 7.35-7.33 (m, 1H), 7.31 (td, *J* = 7.5, 1.3 Hz, 1H), 7.17 (td, *J* = 7.7, 1.4 Hz, 1H), 7.09 – 7.06 (m, 2H), 7.04 – 6.99 (m, 3H), 6.96 – 6.91 (m, 4H), 6.15 (s, 1H), 5.43 (s, 2H), 2.27 (s, 3H), 2.25 (s, 3H). **^{13}C NMR** (126 MHz, $CDCl_3$) δ (ppm): 141.4 (C), 141.1 (C), 139.6 (C), 139.1 (C), 138.3 (C), 137.9 (CH), 136.3 (2xC), 135.5 (C), 132.2 (C), 131.2 (CH), 130.9 (2xCH), 129.4 (2xCH), 128.8 (2xCH), 128.7 (CH), 128.4 (2xCH), 127.9 (CH), 127.8 (CH), 107.0 (CH), 54.9 (CH_2), 21.31 (CH_3), 21.30 (CH_3). **HRMS (APCI-FIA-TOF)** m/z: $[M+H]^+$ Calcd for $C_{26}H_{23}N_2$ 363.1856; found 363.1855.

4,5-Bis(4-methoxyphenyl)-10H-benzo[e]pyrazolo[1,5-a]azepine

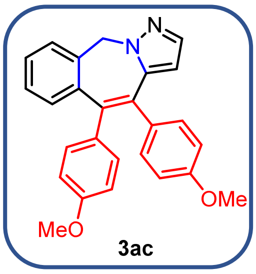

A 5 mL vial equipped with a magnetic stir bar was charged with **1a** (31.6 mg, 0.2 mmol, 1 equiv), **2c** (71.5 mg, 0.3 mmol, 1.5 equiv), $Pd(OAc)_2$ (4.5 mg, 0.02 mmol, 0.1 equiv) and AgOAc (70.1 mg, 0.42 mmol, 2.1 equiv). Then a solution of pivalic acid (102.1 mg, 1.0 mmol, 5 equiv) in 2 mL of DMF was added. The resulting solution was heated at 120 ºC in an oil bath for 24 h. The reaction mixture was extracted with $NaHCO_{3(aq)}$ (3 x 20 mL). The organic layer was dried over $MgSO_4$ and concentrated *in vacuo*. The product was purified by column chromatography on silica gel using Hexane/EtOAc (8:2) as eluent to afford **3ac** as yellow solid in 35% yield (27.4 mg, 0.070 mmol). mp: 171-173 ºC. ^{1}**H NMR** (500 MHz, $CDCl_3$) δ (ppm): 7.38 (dd, *J* = 7.7, 1.4 Hz, 1H), 7.27 (d, *J* = 2.0 Hz, 1H), 7.24 (td, *J* = 7.5, 1.3 Hz, 1H), 7.11 (td, *J* = 7.7, 1.4 Hz, 1H), 7.05 – 7.01 (m, 2H), 6.97 – 6.94 (m, 1H), 6.90 – 6.86 (m, 2H), 6.69 – 6.65 (m, 2H), 6.63 – 6.58 (m, 2H), 6.08 (d, *J* = 2.1 Hz, 1H), 5.35 (s, 2H), 3.68 (s, 3H), 3.67 (s, 3H). ^{13}**C NMR** (126 MHz, $CDCl_3$) δ (ppm): 158.3 (C), 158.2 (C), 141.5 (C), 141.2 (C), 139.6 (C), 137.8 (CH), 135.5 (C), 134.5 (C), 133.8 (C), 132.3 (2xCH), 131.7 (C), 131.2 (CH), 130.6 (2xCH), 128.8 (CH), 127.9 (2xCH), 113.6 (2xCH), 113.1 (2xCH), 106.9 (CH), 55.3 (CH_3), 55.2 (CH_3), 54.8 (CH_2). **HRMS (APCI-FIA-TOF)** m/z: $[M+H]^+$ Calcd for $C_{26}H_{23}N_2O_2$ 395.1754; found 395.1763.

4,5-Bis(4-fluorophenyl)-10H-benzo[e]pyrazolo[1,5-a]azepine

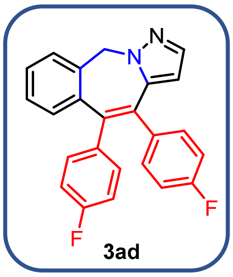

A 5 mL vial equipped with a magnetic stir bar was charged with **1a** (31.6 mg, 0.2 mmol, 1 equiv), **2d** (64.3 mg, 0.3 mmol, 1.5 equiv), $Pd(OAc)_2$ (4.5 mg, 0.02 mmol, 0.1 equiv) and AgOAc (70.1 mg, 0.42 mmol, 2.1 equiv). Then a solution of pivalic acid (204.3 mg, 2.0 mmol, 10 equiv) in 2 mL of DMF was added. The resulting solution was heated at 120 ºC in an oil bath for 24 h. The reaction mixture was extracted with $NaHCO_{3(aq)}$ (3 x 20 mL). The organic layer was dried over $MgSO_4$ and concentrated *in vacuo*. The product was purified by column chromatography on silica gel using Hexane/EtOAc (7:3) as eluent to afford **3ad** as yellow solid in 61% yield (45.5 mg, 0.123 mmol). mp: 181 - 183 ºC. ^{1}**H NMR** (500 MHz, $CDCl_3$) δ (ppm): 7.47 (dd, *J* = 7.5, 1.4 Hz, 1H), 7.37 – 7.32 (m, 2H), 7.21 (td, *J* = 7.7, 1.4 Hz, 1H), 7.16 – 7.12 (m, 2H), 7.03 – 6.98 (m, 3H), 6.94 – 6.88 (m, 2H), 6.88 – 6.83 (m, 2H), 6.13 (d, *J* = 1.9 Hz, 1H), 5.42 (s, 2H). ^{13}**C NMR** (126 MHz, $CDCl_3$) δ (ppm): 161.7 (C, d, *J* = 246.6 Hz), 161.6 (C, d, *J* = 247.0 Hz), 140.8 (C), 140.4 (C), 138.9 (C), 138.2 (CH), 137.7 (C, d, *J* = 3.6 Hz), 137.0 (C, d, *J* = 3.6 Hz), 135.5 (C), 132.6 (2xCH, d, *J* = 8.1 Hz), 131.9 (C), 131.1 (2xCH, d, *J* = 7.9 Hz), 131.0 (CH), 129.1 (CH) 128.1 (2xCH, d, *J* = 9.9 Hz), 115.2 (2xCH, d, *J* = 21.4 Hz), 114.9 (2xCH, d, *J* = 21.4 Hz), 107.2 (CH), 55.1 (CH_2). ^{19}**F NMR** (471 MHz, $CDCl_3$) δ (ppm): -114.9, -114.8. **HRMS (APCI-FIA-TOF)** m/z: $[M+H]^+$ Calcd for $C_{24}H_{17}F_2N_2$ 371.1354; found 371.1364

4,5-Bis(4-(trifluoromethyl)phenyl)-10H-benzo[e]pyrazolo[1,5-a]azepine

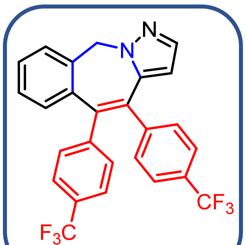

3ae

A 5 mL vial equipped with a magnetic stir bar was charged with **1a** (31.6 mg, 0.2 mmol, 1 equiv), **2e** (94.3 mg, 0.3 mmol, 1.5 equiv), $Pd(OAc)_2$ (4.5 mg, 0.02 mmol, 0.1 equiv) and AgOAc (70.1 mg, 0.42 mmol, 2.1 equiv). Then a solution of pivalic acid (102.1 mg, 1.0 mmol, 5 equiv) in 2 mL of DMF was added. The resulting solution was heated at 120 ºC in an oil bath for 24 h. The reaction mixture was extracted with $NaHCO_{3(aq)}$ (3 x 20 mL). The organic layer was dried over $MgSO_4$ and concentrated *in vacuo*. The product was purified by column chromatography on silica gel using Hexane/EtOAc (8:2) as eluent to afford **3ae** as yellow solid in 78% yield (73.8 mg, 0.157 mmol). mp: 167 – 169 ºC. **^{1}H NMR** (500 MHz, $CDCl_3$) δ (ppm): 7.49 (dd, *J* = 8.1, 4.0 Hz, 3H), 7.43 (d, *J* = 8.0 Hz, 2H), 7.40 – 7.36 (m, 2H), 7.31 (d, *J* = 7.9 Hz, 2H), 7.22 (t, *J* = 7.7, 1H), 7.18 (d, *J* = 7.9 Hz, 2H), 6.93 (d, *J* = 7.9 Hz, 1H), 6.12 (d, *J* = 2.0 Hz, 1H), 5.46 (s, 2H). **^{13}C NMR** (126 MHz, $CDCl_3$) δ (ppm): 145.0 (C, q, *J* = 1.0 Hz), 144.2 (C, q, *J* = 1.1 Hz), 140.3 (C), 139.4 (C), 138.5 (CH), 138.0 (C), 135.6 (C), 132.0 (C), 131.3 (2xCH), 130.9 (CH), 129.9 (2xCH), 129.6 (CH), 129.5 (C, q, *J* = 32.5 Hz), 129.3 (C, q, *J* = 34.5 Hz), 128.3 (CH), 128.2 (CH), 125.4 (2xCH, q, *J* = 3.7 Hz), 125.0 (2xCH, q, *J* = 3.8 Hz), 124.0 (2xCF_3, q, *J* = 272.3 Hz) 107.5 (CH), 55.1 (CH_2). **^{19}F NMR** (471 MHz, $CDCl_3$) δ (ppm): -62.66, -62.65. **HRMS (APCI-FIA-TOF)** m/z: $[M+H]^+$ Calcd for $C_{26}H_{17}F_6N_2$ 471.1290; found 471.1290.

Diethyl 4,4'-(10H-benzo[e]pyrazolo[1,5-a]azepine-4,5-diyl)dibenzoate

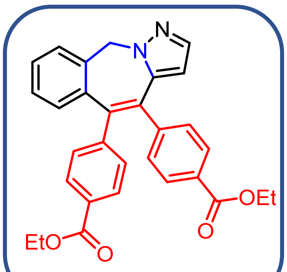

3af

A 5 mL vial equipped with a magnetic stir bar was charged with **1a** (31.6 mg, 0.2 mmol, 1 equiv), **2f** (96.7 mg, 0.3 mmol, 1.5 equiv), $Pd(OAc)_2$ (4.5 mg, 0.02 mmol, 0.1 equiv) and AgOAc (70.1 mg, 0.42 mmol, 2.1 equiv). Then a solution of pivalic acid (102.1 mg, 1.0 mmol, 5 equiv) in 2 mL of DMF was added. The resulting solution was heated at 120 ºC in an oil bath for 24 h. The reaction mixture was extracted with $NaHCO_{3(aq)}$ (3 x 20 mL). The organic layer was dried over $MgSO_4$ and concentrated *in vacuo*. The product was purified by column chromatography on silica gel using Hexane/EtOAc (1:1) as eluent to afford **3af** as yellow solid in 65% (61.8 mg, 0.129 mmol). mp: 158 – 160 ºC. **^{1}H NMR** (500 MHz, $CDCl_3$) δ (ppm): 7.90 – 7.87 (m, 2H), 7.85 – 7.79 (m, 2H), 7.52 – 7.46 (m, 1H), 7.39 – 7.32 (m, 2H), 7.27 – 7.24 (m, 2H), 7.19 (td, *J* = 7.6, 1.3 Hz, 1H), 7.14 – 7.10 (m, 2H), 6.94 (dd, *J* = 8.0, 1.2 Hz, 1H), 6.13 (d, *J* = 2.0 Hz, 1H), 5.47 (s, 2H), 4.33 (ap, *J* = 7.2 Hz, 4H), 1.36 (aq, *J* = 7.2, 6H). **^{13}C NMR** (126 MHz, $CDCl_3$) δ (ppm): 166.30 (C), 166.28 (C), 146.1 (C), 145.2 (C), 140.7 (C), 139.7 (C), 138.24 (CH), 138.18 (C), 135.5 (C), 132.1 (C), 131.0 (2xCH), 130.9 (CH), 129.6 (2xCH), 129.5 (2xCH), 129.4 (CH), 129.3 (C), 129.18 (C), 129.17 (2xCH), 128.3 (CH), 128.1 (CH), 107.3 (CH), 61.2

(2xCH$_2$), 55.0 (CH$_2$), 14.4 (2xCH$_3$). **HRMS (APCI-FIA-TOF)** m/z: $[M+H]^+$ Calcd for $C_{30}H_{27}N_2O_4$ 479.1965; found 479.1966.

4,5-Bis(4-bromophenyl)-10H-benzo[e]pyrazolo[1,5-a]azepine

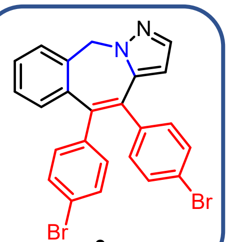

3ag

A 5 mL vial equipped with a magnetic stir bar was charged with **1a** (31.6 mg, 0.2 mmol, 1 equiv), **2g** (100.8 mg, 0.2 mmol, 1.0 equiv), Pd(OAc)$_2$ (4.5 mg, 0.02 mmol, 0.1 equiv) and AgOAc (70.1 mg, 0.42 mmol, 2.1 equiv). Then a solution of pivalic acid (102.1 mg, 1.0 mmol, 5 equiv) in 2 mL of DMF was added. The resulting solution was heated at 120 ºC in an oil bath for 24 h. The reaction mixture was extracted with $NaHCO_{3(aq)}$ (3 x 20 mL). The organic layer was dried over $MgSO_4$ and concentrated *in vacuo*. The product was purified by column chromatography on silica gel using Hexane/EtOAc (8:2) as eluent to afford **3ag** as orange solid in 50% yield (49.6 mg, 0.101 mmol). mp: 240 – 242 ºC. **^{1}H NMR** (500 MHz, CDCl$_3$) δ (ppm): 7.49 – 7.43 (m, 1H), 7.38 – 7.33 (m, 4H), 7.32 – 7.29 (m, 2H), 7.20 (td, *J* = 7.7, 1.4 Hz, 1H), 7.09 – 7.02 (m, 2H), 6.96 (dd, *J* = 8.0, 1.0 Hz, 1H), 6.94 – 6.88 (m, 2H), 6.11 (d, *J* = 2.0 Hz, 1H), 5.41 (s, 2H). **^{13}C NMR** (126 MHz, CDCl$_3$) δ (ppm): 140.5 (C), 140.4 (C), 139.9 (C), 139.8 (C), 138.5 (C), 138.4 (CH), 135.6 (C), 132.6 (2xCH), 131.7 (C), 131.5 (2xCH), 131.2 (2xCH), 131.1 (2xCH), 131.0 (CH), 129.3 (CH), 128.2 (CH), 128.1 (CH), 121.3 (C), 121.2 (C), 107.3 (CH), 55.0 (CH$_2$).**HRMS (APCI-FIA-TOF)** m/z: $[M+H]^+$ Calcd for $C_{24}H_{17}Br_2N_2$ 490.9753; found 490.9763.

Diethyl 3,3'-(10H-benzo[e]pyrazolo[1,5-a]azepine-4,5-diyl)dibenzoate

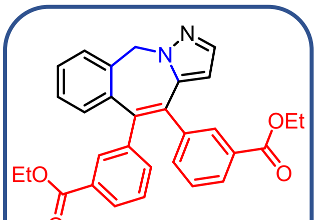

3ah

A 5 mL vial equipped with a magnetic stir bar was charged with **1a** (31.6 mg, 0.2 mmol, 1 equiv), **2h** (96.7 mg, 0.3 mmol, 1.5 equiv), Pd(OAc)$_2$ (4.5 mg, 0.02 mmol, 0.1 equiv) and AgOAc (70.1 mg, 0.42 mmol, 2.1 equiv). Then a solution of pivalic acid (102.1 mg, 1.0 mmol, 5 equiv) in 2 mL of DMF was added. The resulting solution was heated at 120 ºC in an oil bath for 24 h. The reaction mixture was extracted with $NaHCO_{3(aq)}$ (3 x 20 mL). The organic layer was dried over $MgSO_4$ and concentrated *in vacuo*. The product was purified by column chromatography on silica gel using Hexane/EtOAc (4:6) as eluent to afford **3ah** as an amorphous brown solid in 76% (72.6 mg, 0.152 mmol). **^{1}H NMR** (500 MHz, CDCl$_3$) δ (ppm): 7.93 (s, 1H), 7.83 (dd, *J* = 15.5, 7.7 Hz, 2H), 7.77 (s, 1H), 7.51 (d, *J* = 7.5 Hz, 1H), 7.40 – 7.34 (m, 3H), 7.30 – 7.18 (m, 4H), 6.99 (d, *J* = 7.9 Hz, 1H), 6.14 (s, 1H), 5.50 (s, 2H), 4.35 (q, *J* = 7.1 Hz, 2H), 4.32 (q, *J* = 7.1 Hz, 2H), 1.37 (aq, *J* = 7.1 Hz, 6H). **^{13}C NMR (126 MHz, CDCl$_3$)** δ (ppm): 166.3 (C), 166.2 (C), 141.7 (C), 140.9 (C), 140.7 (C), 140.0 (C), 138.4 (C), 138.2 (CH), 135.5 (C), 135.3 (CH), 133.9 (CH), 132.1 (C), 131.9

(CH), 130.9 (CH), 130.5 (CH), 130.4 (C), 130.2 (C), 129.2 (CH), 128.3 (CH), 128.22 (CH), 128.19 (CH), 128.17 (CH), 128.1 (CH), 127.9 (CH), 107.3 (CH), 61.14 (CH_2), 61.12 (CH_2), 55.0 (CH_2), 14.4 (CH_3), 14.3 (CH_3). **HRMS (APCI-FIA-TOF)** m/z: $[M+H]^+$ Calcd for $C_{30}H_{27}N_2O_4$ 479.1965; found 479.1967.

4,5-Di-*m*-tolyl-10H-benzo[e]pyrazolo[1,5-a]azepine

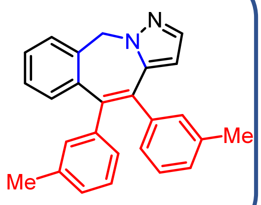

3ai

A 5 mL vial equipped with a magnetic stir bar was charged with **1a** (31.6 mg, 0.2 mmol, 1 equiv), **2i** (61.9 mg, 0.3 mmol, 1.5 equiv), $Pd(OAc)_2$ (4.5 mg, 0.02 mmol, 0.1 equiv) and AgOAc (70.1 mg, 0.42 mmol, 2.1 equiv). Then a solution of pivalic acid (40.9 mg, 0.4 mmol, 2 equiv) in 2 mL of DMF was added. The resulting solution was heated at 120 ºC in an oil bath for 24 h. The reaction mixture was extracted with $NaHCO_{3(aq)}$ (3 x 20 mL). The organic layer was dried over $MgSO_4$ and concentrated *in vacuo*. The product was purified by column chromatography on silica gel using Hexane/EtOAc (8:2) as eluent to afford **3ai** as a yellow solid in 55% (39.7 mg, 0.110 mmol). mp: 132 -134 ºC. **^{1}H NMR** (500 MHz, $CDCl_3$) δ (ppm): 7.47 (d, *J* = 7.5 Hz, 1H), 7.39 – 7.28 (m, 2H), 7.19 (t, *J* = 7.7 Hz, 1H), 7.07 (p, *J* = 7.7 Hz, 2H), 7.03 – 6.97 (m, 3H), 6.92 (dd, *J* = 15.8, 7.6 Hz, 2H), 6.88 – 6.85 (m, 2H), 6.16 (s, 1H), 5.45 (s, 2H), 2.24 (s, 3H), 2.20 (s, 3H). **^{13}C NMR** (126 MHz, $CDCl_3$) δ (ppm): 141.8 (C), 141.5 (C), 141.0 (C), 140.9 (C), 139.3 (C), 137.9 (CH), 137.5 (C), 137.1 (C), 135.4 (C), 132.4 (C), 131.7 (CH), 131.1 (CH), 130.2 (CH), 128.8 (CH), 128.1 (CH), 127.91 (CH), 127.90 (CH), 127.8 (CH), 127.50 (CH), 127.46 (CH), 127.4 (CH), 126.6 (CH), 107.1 (CH), 54.9 (CH_2), 21.5 (CH_3), 21.4 (CH_3). **HRMS (APCI-FIA-TOF)** m/z: $[M+H]^+$ Calcd for $C_{26}H_{23}N_2$ 363.1856; found 363.1861.

3,3'-(10H-benzo[e]pyrazolo[1,5-a]azepine-4,5-diyl)dibenzonitrile

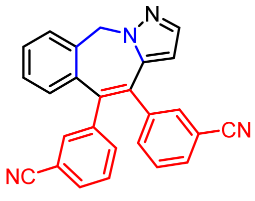

3aj

A 5 mL vial equipped with a magnetic stir bar was charged with **1a** (31.6 mg, 0.2 mmol, 1 equiv), **2j** (68.5 mg, 0.3 mmol, 1.0 equiv), $Pd(OAc)_2$ (4.5 mg, 0.02 mmol, 0.1 equiv) and AgOAc (70.1 mg, 0.42 mmol, 2.1 equiv). Then a solution of pivalic acid (204.2 mg, 2 mmol, 10 equiv) in 2 mL of DMF was added. The resulting solution was heated at 120 ºC in an oil bath for 24 h. The reaction mixture was extracted with $NaHCO_{3(aq)}$ (3 x 20 mL). The organic layer was dried over $MgSO_4$ and concentrated *in vacuo*. The product was purified by column chromatography on silica gel using Hexane/EtOAc (1:1) as eluent to afford **3aj** as a yellow solid in 51% yield (39.5 mg, 0.103 mmol). mp: 214 -216 ºC. **^{1}H NMR** (500 MHz, $CDCl_3$) δ (ppm): 7.52 – 7.47 (m, 2H), 7.47 – 7.44 (m, 3H), 7.42 – 7.39 (m, 1H), 7.38 (m, 2H), 7.34 – 7.32 (m, 1H), 7.32 – 7.30 (m, 2H), 7.26 – 7.22 (m, 1H), 6.90 (dd, *J* = 7.9, 0.9 Hz, 1H), 6.09 (d, *J* = 1.9 Hz, 1H), 5.45 (s, 2H). **^{13}C NMR** (126 MHz, $CDCl_3$) δ (ppm): 142.4 (C), 141.6 (C), 139.9

(C), 139.0 (C), 138.6 (CH), 137.4 (C), 135.6 (C), 135.2 (CH), 134.1 (CH), 133.9 (CH), 132.8 (CH), 131.4 (C), 131.2 (CH), 131.0 (CH), 130.8 (CH), 129.9 (CH), 129.4 (CH), 129.0 (CH), 128.5 (CH), 128.4 (CH), 118.24 (C), 118.22 (C), 112.8 (C), 112.6 (C), 107.5 (CH), 55.0 (CH_2). **HRMS (APCI-FIA-TOF)** m/z: $[M+H]^+$ Calcd for $C_{26}H_{17}N_4$ 385.1448; found 385.1456.

4,5-Bis(3,5-bis(trifluoromethyl)phenyl)-10H-benzo[e]pyrazolo[1,5-a]azepine

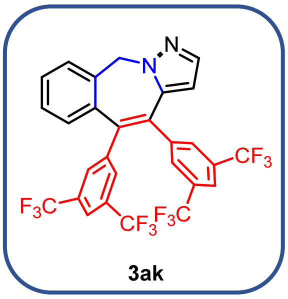

A 5 mL vial equipped with a magnetic stir bar was charged with **1a** (31.6 mg, 0.2 mmol, 1 equiv), **2k** (135.1 mg, 0.3 mmol, 1.5 equiv), $Pd(OAc)_2$ (4.5 mg, 0.02 mmol, 0.1 equiv) and AgOAc (70.1 mg, 0.42 mmol, 2.1 equiv). Then a solution of pivalic acid (102.1 mg, 1.0 mmol, 5 equiv) in 2 mL of DMF was added. The resulting solution was heated at 120 ºC in an oil bath for 24 h. The reaction mixture was extracted with $NaHCO_{3(aq)}$ (3 x 20 mL). The organic layer was dried over $MgSO_4$ and concentrated *in vacuo*. The product was by column chromatography on silica gel using Hexane/EtOAc (8:2) as eluent to afford **3ak** as yellow solid in 45% (54.8 mg, 0.090 mmol). mp: 224-226 ºC. **^{1}H NMR** (500 MHz, $CDCl_3$) δ (ppm): 7.70 (s, 1H), 7.67 (s, 1H), 7.60 (s, 2H), 7.56 (d, J = 7.6 Hz, 1H), 7.49 – 7.44 (m, 3H), 7.43 (d, J = 2.0 Hz, 1H), 7.30 (td, J = 7.7, 1.4 Hz, 1H), 6.94 (d, J = 7.9 Hz, 1H), 6.12 (d, J = 2.0 Hz, 1H), 5.52 (s, 2H). **^{13}C NMR** (126 MHz, $CDCl_3$) δ (ppm): 142.9 (C), 142.1 (C), 139.9 (C), 138.9 (CH), 138.3 (C), 136.6 (C), 135.6 (C), 132.2 (C, q, J = 33.8 Hz), 131.9 (C, q, J = 33.6 Hz), 131.6 (C), 130.9 (2xCH, q, J = 4.1 Hz), 130.6 (CH), 130.4 (CH), 129.7 (2xCH, q, J = 3.6 Hz), 129.0 (CH), 128.7 (CH), 122.9 (4xC, q, J = 272.2 Hz), 121.3 (2xCH, h, J = 3.9 Hz), 107.7 (CH), 55.2 (CH_2). **^{19}F NMR** (471 MHz, $CDCl_3$) δ (ppm): -63.3, -63.2. **HRMS (APCI-FIA-TOF)** m/z: $[M+H]^+$ Calcd for $C_{28}H_{15}F_{12}N_2$ 607.1038; found 607.1060.

4,5-di(naphthalen-2-yl)-10H-benzo[e]pyrazolo[1,5-a]azepine

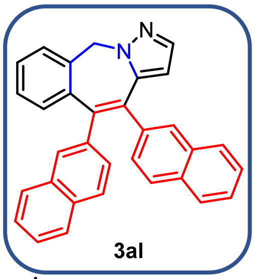

A 5 mL vial equipped with a magnetic stir bar was charged with **1a** (31.6 mg, 0.2 mmol, 1 equiv), **2l** (83.5 mg, 0.3 mmol, 1.0 equiv), $Pd(OAc)_2$ (4.5 mg, 0.02 mmol, 0.1 equiv) and AgOAc (70.1 mg, 0.42 mmol, 2.1 equiv). Then a solution of pivalic acid (102.1 mg, 1.0 mmol, 5 equiv) in 2 mL of DMF was added. The resulting solution was heated at 120 ºC in an oil bath for 24 h. The reaction mixture was extracted with $NaHCO_{3(aq)}$ (3 x 20 mL). The organic layer was dried over $MgSO_4$ and concentrated *in vacuo*. The product was purified by column chromatography on silica gel using Hexane/EtOAc (8:2) as eluent to afford **3al** as yellow solid in 80% yield (69.3 mg, 0.159 mmol). mp: 204-206 ºC. **^{1}H NMR** (500 MHz, $CDCl_3$) δ (ppm):

7.78 (s, 1H), 7.74 – 7.68 (m, 2H), 7.68 – 7.60 (m, 4H), 7.57 (d, *J* = 8.4 Hz, 1H), 7.54 (d, *J* = 7.6 Hz, 1H), 7.43 – 7.34 (m, 7H), 7.28 (dd, *J* = 8.4, 1.6 Hz, 1H), 7.21 – 7.14 (m, 1H), 7.06 (d, *J* = 7.9 Hz, 1H), 6.21 (d, *J* = 2.0 Hz, 1H), 5.57 (s, 2H). **^{13}C NMR** (126 MHz, $CDCl_3$) δ (ppm): 141.4 (C), 140.8 (C), 139.4 (C), 139.3 (C), 138.6 (C), 138.3 (CH), 135.7 (C), 133.1 (C), 132.8 (C), 132.7 (C), 132.2 (C), 132.1 (C), 131.3 (CH), 130.2 (CH), 129.0 (CH), 128.9 (CH), 128.3 (CH), 128.03 (CH), 128.00 (CH), 127.97 (2xCH), 127.9 (CH), 127.8 (CH), 127.7 (CH), 127.6 (CH), 127.3 (CH), 126.2 (CH), 126.13 (CH), 126.10 (CH), 126.0 (CH), 107.4 (CH), 55.1 (CH_2). **HRMS (APCI-FIA-TOF)** m/z: $[M+H]^+$ Calcd for $C_{32}H_{23}N_2$ 435.1856; found 435.1856.

5-(4-Methoxyphenyl)-4-(4-(trifluoromethyl)phenyl)-10H-benzo[e]pyrazolo[1,5-a]azepine

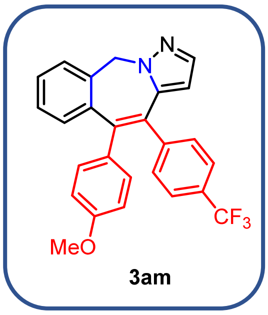

A 5 mL vial equipped with a magnetic stir bar was charged with **1a** (31.6 mg, 0.2 mmol, 1 equiv), **2m** (82.9 mg, 0.3 mmol, 1.5 equiv), $Pd(OAc)_2$ (4.5 mg, 0.02 mmol, 0.1 equiv) and AgOAc (70.1 mg, 0.42 mmol, 2.1 equiv). Then a solution of pivalic acid (102.1 mg, 1.0 mmol, 5 equiv) in 2 mL of DMF was added. The resulting solution was heated at 120 ºC in an oil bath for 24 h. The reaction mixture was extracted with $NaHCO_{3(aq)}$ (3 x 20 mL). The organic layer was dried over $MgSO_4$ and concentrated *in vacuo*. The product was purified by column chromatography on silica gel using Hexane/EtOAc (7:3) as eluent to afford **3am** (as a mixture of regioisomers 1.6:1) as yellow solid in 56% (48.8 mg, 0.113 mmol). **^{1}H NMR** (500 MHz, $CDCl_3$) δ (ppm): 7.50 – 7.44 (m, 6H), 7.42 (d, *J* = 8.1 Hz, 2H), 7.38 – 7.30 (m, 8H), 7.24 – 7.16 (m, 5H), 7.10 – 7.06 (m, 2H), 7.05 – 7.02 (m, 2H), 6.96 – 6.89 (m, 4H), 6.77 – 6.71 (m, 2H), 6.70 – 6.65 (m, 3H), 6.16 (minor, d, *J* = 2.0 Hz, 1H), 6.12 (major, d, *J* = 2.0 Hz, 1.4H), 5.43 (minor, s, 2H), 5.42 (major, s, 3H), 3.75 (minor, s, 3H), 3.74 (major, s, 5H). **^{13}C NMR** (126 MHz, $CDCl_3$) δ (ppm): 158.6 (C), 158.5 (C), 145.9 (C), 145.2 (C), 141.7 (C), 140.7 (C), 139.83 (C), 139.79 (C), 139.2 (C), 138.5 (C), 138.3 (CH), 135.7 (C), 135.6 (C), 133.7 (C), 133.1 (C), 133.0 (C), 132.2 (CH), 131.4 (CH), 131.3 (CH), 130.91 (C), 130.85 (CH), 130.5 (CH), 130.0 (CH), 129.2 (CH), 129.1 (CH), 128.1 (CH), 128.03 (CH), 127.98 (CH), 127.95 (CH), 125.3 (C), 125.2 (major, q, *J* = 3.6 Hz), 124.74 (minor, q, *J* = 3.6 Hz, CH), 124.68 (minor, q, *J* = 3.6 Hz, CH), 113.7 (minor, CH), 113.3 (major, CH), 107.3 (minor, CH), 107.0 (major, CH), 55.29 (CH_3), 55.26 (CH_3), 55.0 (2xCH_2). **^{19}F NMR** (471 MHz, $CDCl_3$) δ (ppm): -62.48, -62.49. **HRMS (APCI-FIA-TOF)** m/z: $[M+H]^+$ Calcd for $C_{26}H_{20}N_2F_3O$ 433.1522; found 433.1528.

Methyl 4-phenyl-10H-benzo[e]pyrazolo[1,5-a]azepine-5-carboxylate

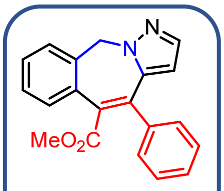

3an

A 5 mL vial equipped with a magnetic stir bar was charged with **1a** (31.6 mg, 0.2 mmol, 1 equiv), **2n** (48.1 mg, 0.3 mmol, 1.5 equiv), $Pd(OAc)_2$ (4.5 mg, 0.02 mmol, 0.1 equiv) and AgOAc (70.1 mg, 0.42 mmol, 2.1 equiv). Then a solution of pivalic acid (102.1 mg, 1.0 mmol, 5 equiv) in 2 mL of DMF was added. The resulting solution was heated at 120 ºC in an oil bath for 24 h. The reaction mixture was extracted with $NaHCO_{3(aq)}$ (3 x 20 mL). The organic layer was dried over $MgSO_4$ and concentrated *in vacuo*. The product was purified by column chromatography on silica gel using Hexane/EtOAc (8:2) as eluent to afford **3an** as light yellow crystals in 32% yield (20.2 mg, 0.064 mmol). mp: 152-154 ºC. **^{1}H NMR** (500 MHz, $CDCl_3$) δ (ppm): 7.88 (dd, *J* = 7.6, 1.7 Hz, 1H), 7.46 (dd, *J* = 7.4, 1.9 Hz, 1H), 7.42 (dd, *J* = 7.4, 1.6 Hz, 1H), 7.41 – 7.36 (m, 7H), 5.97 (d, *J* = 2.1 Hz, 1H), 5.38 (s, 2H), 3.44 (s, 3H). **^{13}C NMR** (126 MHz, $CDCl_3$) δ (ppm): 169.2 (CO), 139.9 (C), 139.6 (C), 138.3 (CH), 135.5 (C), 134.9 (C), 133.4 (C), 132.9 (C), 129.6 (CH), 129.4 (2xCH), 128.6 (2xCH), 128.5 (CH), 128.3 (2xCH), 128.2 (CH), 108.5 (CH), 54.9 (CH_2), 52.2 (CH_3). **HRMS (APCI-FIA-TOF)** m/z: $[M+H]^+$ Calcd for $C_{20}H_{17}N_2O_2$ 317.1285; found 317.1282.

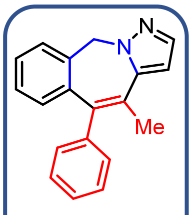

3ao

5-Methyl-4-phenyl-10H-benzo[e]pyrazolo[1,5-a]azepine

A 5 mL vial equipped with a magnetic stir bar was charged with **1a** (31.6 mg, 0.2 mmol, 1 equiv), **2o** (23.2 mg, 0.2 mmol, 1.0 equiv), $Pd(OAc)_2$ (4.5 mg, 0.02 mmol, 0.1 equiv) and AgOAc (70.1 mg, 0.42 mmol, 2.1 equiv). Then a solution of pivalic acid (102.1 mg, 1.0 mmol, 5 equiv) in 2 mL of DMF was added. The resulting solution was heated at 120 ºC in an oil bath for 24 h. The reaction mixture was extracted with $NaHCO_{3(aq)}$ (3 x 20 mL). The organic layer was dried over $MgSO_4$ and concentrated *in vacuo*. The product was purified by column chromatography on silica gel using Hexane/EtOAc (8:2) as eluent and by Preparative Chromatography TLC Silica Gel 60 F254 plates using Hexanes/Et_2O (8:2) as eluent to afford **3ao** as an amorphous brown solid in 10% yield (5.3 mg, 0.019 mmol). **^{1}H NMR** (500 MHz, $CDCl_3$) δ (ppm): 7.50 (d, *J* = 7.6 Hz, 1H), 7.32 (m, 6H), 7.23 – 7.17 (m, 4H), 5.65 (s, 1H), 5.23 (s, 2H), 2.17 (s, 3H). **^{13}C NMR** (126 MHz, $CDCl_3$) δ (ppm): 141.8 (C), 141.5 (C), 140.4 (C), 137.6 (CH), 135.1 (C), 134.8 (C), 131.3 (C), 129.8 (2xCH), 128.6 (CH), 128.5 (2xCH), 128.3 (CH), 128.1 (CH), 127.9 (CH), 127.6 (CH), 106.9 (CH), 54.6 (CH_2), 22.6 (CH_3). **HRMS (APCI-FIA-TOF)** m/z: $[M+H]^+$ Calcd for $C_{19}H_{17}N_2$ 273.1386; found 273.1380.

5. Unsuccessful Substrates in the Pd-catalyzed [5+2] Rollover Annulation

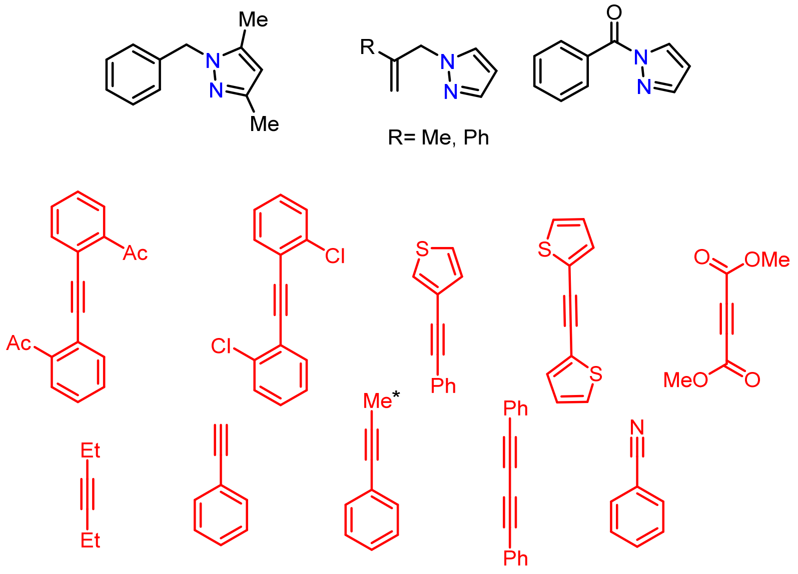

6. Mechanistic Studies

Formation of palladacycle 4a

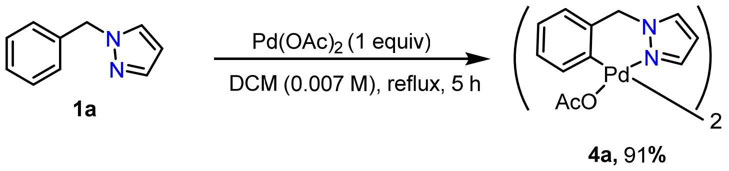

A flame-dried round-bottom flask equipped with a magnetic stir bar was charged with **1a** (38.6 mg, 0.244 mmol, 1 equiv) and $Pd(OAc)_2$ (55.8 mg, 0.249 mmol, 1 equiv). Then, 35 mL of DCM were added and the mixture heated at 60 ºC under reflux in an oil bath for 5h. The reaction was filtrated through a Celite® plug. The solvent was evaporated in vacuo and the residue was dissolved in a minimum amount of DCM. Then, hexanes were added, and a brown precipitate was observed. After filtration, the residue was washed with hexanes to give the palladacycle **4a** as a brown solid in 91% yield (71.9 mg, 0.222 mmol). mp: decomposition at 217 ºC. **^{1}H NMR** (500 MHz, $CDCl_3$) δ (ppm): 7.43 – 7.25 (m, 1H), 7.17 (d, *J* = 18.5 Hz, 1H), 6.90 – 6.61 (m, 4H), 6.06 (s, 1H), 4.96 (d, *J* = 13.9 Hz, 1H), 4.67 (d, *J* = 13.8 Hz, 1H), 2.12 (s, 3H). **^{13}C NMR** (126 MHz, $CDCl_3$) δ (ppm): 181.1 (CO), 141.7 (CH), 137.7 (C), 134.6 (CH), 134.6 (C), 130.5 (CH), 126.4 (CH), 125.1 (CH), 123.9 (CH), 106.4 (CH), 58.2 (CH_2), 24.6 (CH_3). **HRMS (APCI-DIP-TOF)** m/z: $[M+H]^+$ Calcd for $C_{24}H_{24}N_4O_4Pd_2$ 643.9862; found 643.9881.

Stoichiometric reactions

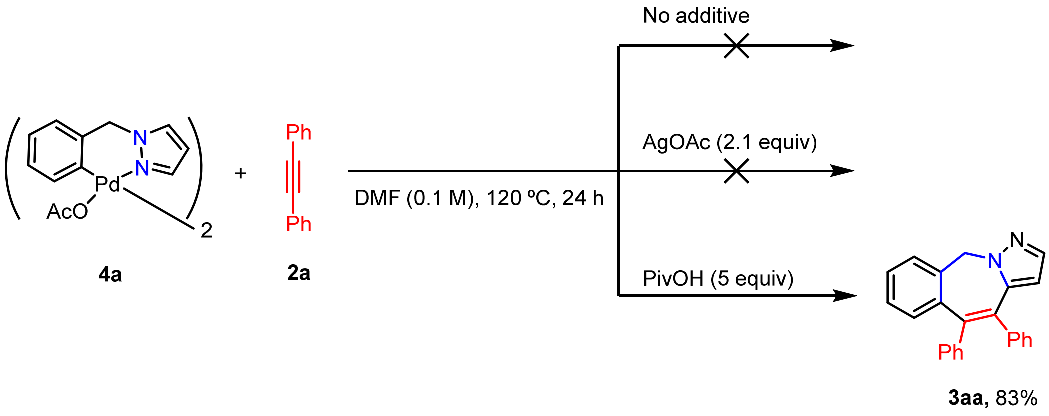

A 5 mL vial equipped with a magnetic stir bar was charged with **4a** (15.0 mg, 0.023 mmol, 1 equiv) and **2a** (12.4 mg, 0.070 mmol, 3.0 equiv). Then a solution of pivalic acid (11.9 mg, 0.116 mmol, 5 equiv) in 0.23 mL of DMF was added. The resulting solution was heated at 120 ºC in an oil bath for 24 h. The reaction mixture was extracted with $NaHCO_{3(aq)}$ (3 x 20 mL). The organic layer was dried over $MgSO_4$ and concentrated *in vacuo*. The product **3aa** was obtained in a 83% NMR yield.

Palladacycle 4a as catalyst

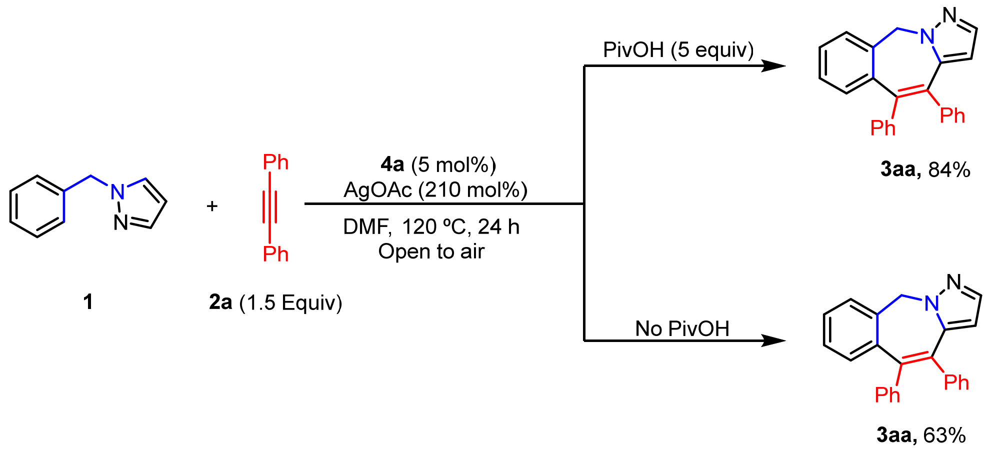

A 5 mL vial equipped with a magnetic stir bar was charged with **1a** (13.2 mg, 0.084 mmol, 1 equiv), **2a** (22.4 mg, 0.126 mmol, 1.5 equiv), palladacycle **4a** (2.7 mg, 0.004 mmol, 0.050 equiv) and AgOAc (29.3 mg, 0.176 mmol, 2.1 equiv). Then, a solution of pivalic acid (42.7 mg, 0.418 mmol, 5 equiv) in 0.84 mL of DMF was added. The resulting solution was heated at 120 ºC in an oil bath for 24 h. The reaction mixture was extracted with $NaHCO_{3(aq)}$ (3 x 20 mL). The organic layer was dried over $MgSO_4$ and concentrated *in vacuo*. The product **3aa** was obtained in an 84% NMR yield in the presence of PivOH and in a 63% NMR yield in the absence of PivOH.

Kinetic Isotopic Effect (KIE) by a competition test

A 5 mL vial equipped with a magnetic stir bar was charged with **1a** (15.8 mg, 0.1 mmol, 1 equiv), **1a-*d5*** (16.3 mg, 0.1 mmol, 1 equiv), **2a** (53.5 mg, 0.3 mmol, 1.5 equiv), $Pd(OAc)_2$ (4.5 mg, 0.02 mmol, 0.1 equiv) and AgOAc (70.1 mg, 0.42 mmol, 2.1 equiv). Then a solution of pivalic acid (102.1 mg, 1.0 mmol, 5 equiv) in 2 mL of DMF was added. The resulting solution was heated at 120 ºC in an oil bath for 2.5 h. The reaction mixture was extracted with $NaHCO_{3(aq)}$ (3 x 20 mL). The organic layer was dried over $MgSO_4$ and concentrated *in vacuo*. The product was purified by column chromatography on silica gel using Hexane/EtOAc (8:2) as eluent to afford 25.7 mg of a mixture of starting materials and products (ratio 0.65:1). After crystallization of a mixture of **3aa** and **3aa-*d4***, a KIE value of 2.0 was obtained by the ratio of H_a signal (red) and the H_b signal (green).

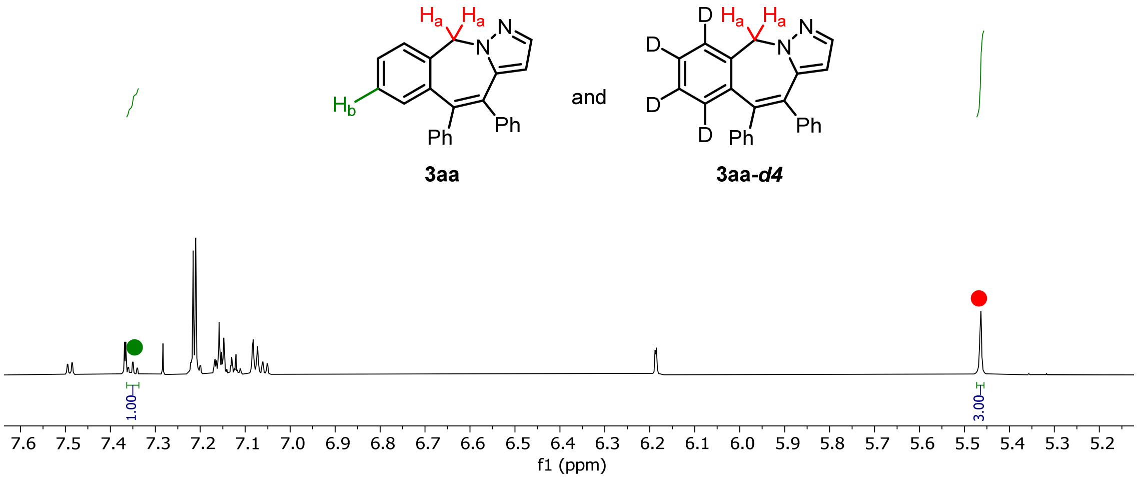

D/H Exchange Experiment

A 5 mL vial equipped with a magnetic stir bar was charged with **1a-*d5*** (32.6 mg, 0.2 mmol, 1 equiv), **2a** (53.5 mg, 0.3 mmol, 1.5 equiv), $Pd(OAc)_2$ (4.5 mg, 0.02 mmol, 0.1 equiv) and AgOAc (70.1 mg, 0.42 mmol, 2.1 equiv). Then a solution of pivalic acid (102.1 mg, 1.0 mmol, 5 equiv) in 2 mL of DMF was added. The resulting solution was heated at 120 ºC in an oil bath for 24h. The reaction mixture was extracted with $NaHCO_{3(aq)}$ (3 x 20 mL). The organic layer was dried over $MgSO_4$ and concentrated *in vacuo*. The product was purified by column chromatography on silica gel using Hexane/EtOAc (8:2) as eluent to afford 32.9 mg of a mixture of **1a-*d5*** and **3aa-*d4*** (4:1 ratio). After crystallization of this mixture in DCM, no proton incorporation in the *ortho* position was found.

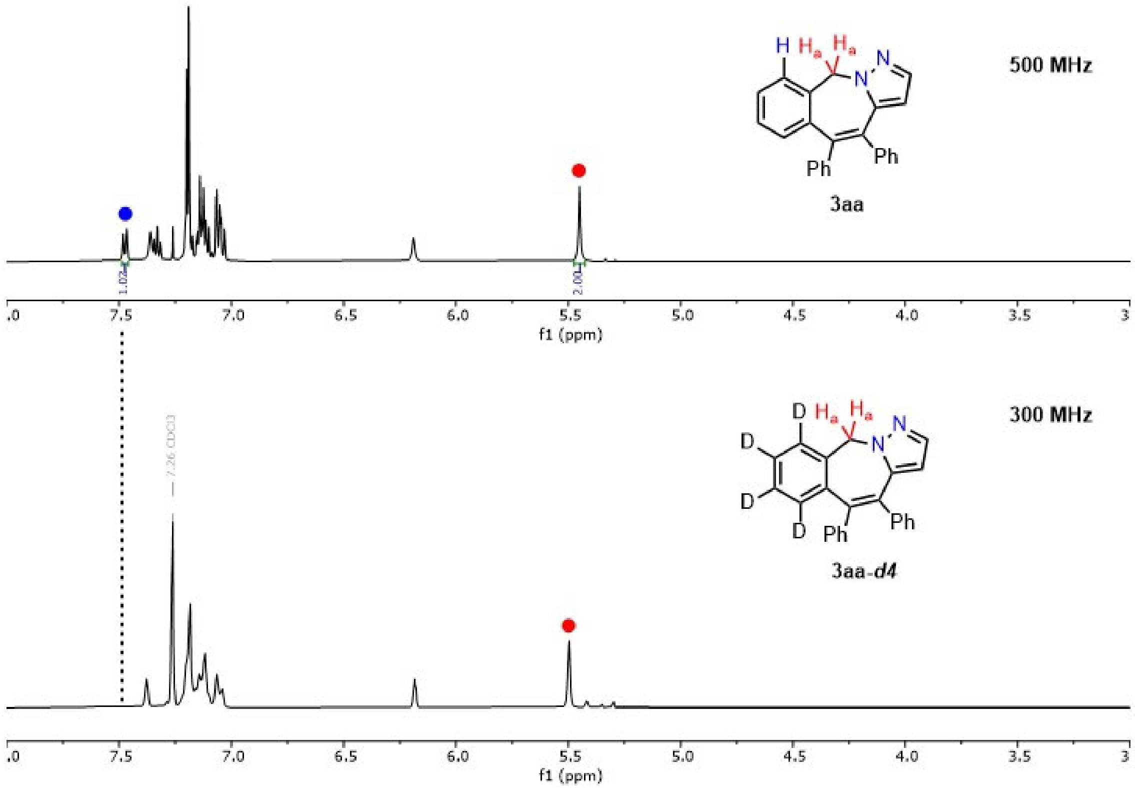

7. X-Ray Crystallographic Data

All X-ray structure were collected in a Bruker D8 VENTURE PHOTON-III C14 Diffractometer, using a microfocus sealed tube Incoatec IμS 3.0 multilayer mirror monochromator as radiation source and a detector resolution of 7.3910 pixels mm^{-1}.

Data collection: Bruker APEX3 software; cell refinement: SAINT V8.40A (Bruker Nano, Inc., 2019); data reduction: SAINT V8.40A (Bruker Nano, Inc., 2019); program(s) used to solve structure: SHELXT 2018/2 (Sheldrick, 2015); program(s) used to refine structure: SHELXL2018/3 (Sheldrick, 2018); molecular graphics: ORTEP 2014.1 (Farrugia, 2012); software used to prepare material for publication: IUCr Journals printCIF.

Crystallographic data for compound 3aa

An X-ray crystal of compound **3aa** was grown by slow evaporation using DCM and hexanes as mixture of solvents.

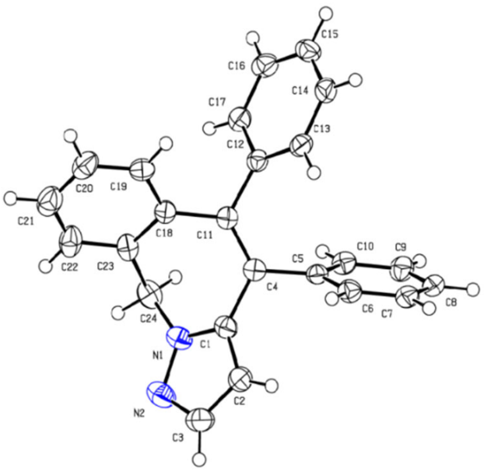

Figure S1. X-Ray coordinate of compound **3aa**. ORTEP drawing showing ellipsoids at the 50% contour probability level.

Table S10. Crystal data and structure refinement for **3aa** CCDC 2208317

Deposition Number CCDC		
Chemical Formula	$C_{24}H_{18}N_2$	
Formula weight	334.40	
Temperature	100 (2) K	
Wavelength	0.71073 Å	
Crystal size	0.10 x 0.15 x 0.21 mm^3	
Crystal system	Monoclinic	
Space group	P 21/c	
	a = 11.7992 (15) Å	α = 90º
Unit cell dimensions	b = 8.4308 (13) Å	β = 96.255 (5)º
	c = 17.709 (2) Å	γ = 90º
Volume	1751.1 (4) Å^3	
Z	4	
Density (calculated)	1.268 Mg m^{-3}	
Absorption coefficient	0.075 mm^{-1}	
F(000)	704	

Crystallographic data for compound 4a

An X-ray crystal of compound **4a** was grown by slow evaporation using DCM and hexanes as mixture of solvents.

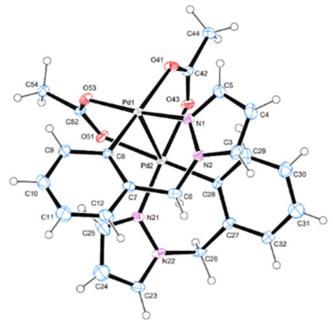

Figure S2. X-Ray coordinate of compound **4a**. ORTEP drawing showing ellipsoids at the 50% contour probability level.

Table S11. Crystal data and structure refinement for **4a** CCDC 2208318

Deposition Number CCDC	
Chemical Formula	$C_{24}H_{24}N_4O_4Pd_2$
Formula weight	645.27
Temperature	100 K
Wavelength	0.71073 Å
Crystal size	0.24 × 0.09 × 0.01 mm
Crystal habit	Plate, clear colourless
Crystal system	Orthorhombic
Space group	*P*212121
Unit cell dimensions	*a* = 9.3674 (10) Å<br>*b* = 13.9484 (15) Å<br>*c* = 18.259 (2) Å
Volume	2385.7 (5) Å3
Z	4
Density (calculated)	1.797 Mg m^{-3}
Absorption coefficient	1.55 mm^{-1}
F(000)	1280

8. Derivatizations

Bromination

3-Bromo-4,5-diphenyl-10H-benzo[e]pyrazolo[1,5-a]azepine

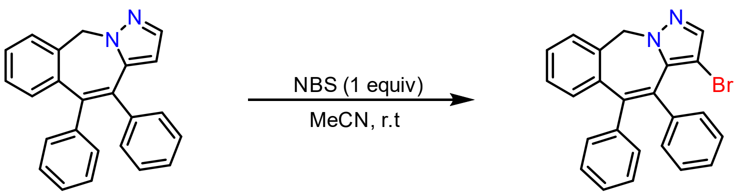

3aa **5**, 77%

Following a similar reported procedure,[15] a 5 mL tube was charged with **3aa** (66.9 mg, 0.20 mmol, 1 equiv) and NBS (35.6 mg, 0.20 mmol, 1 equiv) under an Ar atmosphere. Then, dry MeCN (0.56 mL) was added and the resulting mixture was stirred for 24 h at room temperature. The solvent was removed in vacuo and the crude product was directly purified by column chromatography on silica gel using Hex/EtOAc 7:3 as eluent to afford the brominated product **5** in 77% yield (63.9 mg, 0.155 mmol). **^{1}H NMR** (500 MHz, $CDCl_3$) δ (ppm): 7.35 (t, *J* = 7.6 Hz, 2H), 7.23 (td, *J* = 7.5, 1.4 Hz, 1H), 7.20 – 7.09 (m, 8H), 7.09 – 6.99 (m, 4H), 5.37 (s, 2H). **^{13}C NMR** (126 MHz, $CDCl_3$) δ (ppm): 144.0 (C), 140.9 (C), 140.0 (CH), 138.5 (C), 138.3 (C), 137.4 (C), 135.6 (C), 132.0 (C), 131.3 (2xCH), 130.5 (CH), 130.3 (2xCH), 128.8 (CH), 128.2 (CH), 127.9 (CH), 127.8 (2xCH), 127.3 (2xCH), 127.2 (CH), 127.0 (CH), 95.1 (C), 55.7 (CH_2). **HRMS (APCI-FIA-TOF)** m/z: $[M+H]^+$ Calcd for $C_{24}H_{18}N_2Br$ 413.0648; found 413.0641. m.p: 204 - 206 ºC

N-Alkylation

1-Methyl-4,5-diphenyl-10H-benzo[e]pyrazolo[1,5-a]azepin-1-ium iodide

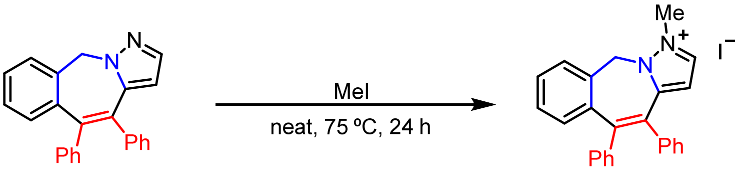

3aa **6**

A 5 mL sealed tube equipped with a magnetic stir bar was charged with **3aa** (452 mg, 1.3 mmol) and MeI (0.4 mL, 6.43 mmol, 5 equiv) under an Ar atmosphere. The mixture was heated at 75 ºC in an oil bath for 24 h. The reaction mixture was concentrated *in vacuo* affording the pyrazolium salt **6** as an amorphous dark yellow solid in 97% yield (596.7 mg, 1.253 mmol). **^{1}H NMR** (500 MHz, $CDCl_3$) δ (ppm): 8.51 (s, 1H), 7.86 (d, *J* = 7.5 Hz, 1H), 7.37 (t, *J* = 7.3 Hz, 1H), 7.23 – 7.04 (m, 9H), 7.02 (d, *J* = 7.8 Hz, 1H), 6.96 (m, 2H), 6.39 (s, 1H), 5.90 (s, 2H), 4.63 (s, 3H). **^{13}C NMR** (126 MHz, $CDCl_3$) δ (ppm): 148.6 (C), 146.0 (C), 139.9 (C), 138.2 (C), 137.7 (C), 137.0 (CH), 133.0 (C), 131.5 (CH), 130.7 (CH), 130.3 (2xCH), 129.8 (2xCH), 129.5 (CH), 129.0 (C), 128.6 (3xCH), 128.0 (CH), 127.9 (2xCH), 127.8 (CH), 107.0 (CH), 52.3 (CH_2), 40.4 (CH_3). **HRMS (ESI-FIA-TOF)** m/z: $[M+H]^+$ Calcd for $C_{25}H_{21}N_2$ 349.1699; found 349.1697.

Reduction of the pyrazolium salt

1-Methyl-4,5-diphenyl-2,3,3a,10-tetrahydro-1H-benzo[e]pyrazolo[1,5-a]azepine

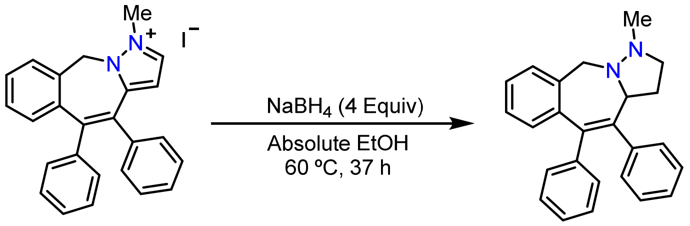

5 **7**

To a stirred solution of the pyrazolium salt **5** (95.3 mg, 0.20 mmol, 1 equiv) in absolute ethanol (1.3 mL) under an Ar atmosphere, was added $NaBH_4$ (15.2 mg, 0.40 mmol, 2 equiv) in portions at room temperature . After that, the solution was heated at 60 ºC with a reflux condenser for 15 h. Then, $NaBH_4$ was recharged (15.2 mg, 0.40 mmol, 2 equiv) and the reaction was heated at 60 ºC for another 10h. Saturated aqueous ammonium chloride (5 mL) was added slowly to a mixture which was then extracted with ethyl acetate (3 x 20 mL). The combined extracts were dried over $MgSO_4$ and evaporated. The crude mixture could be separated by column chromatography using Hex/EtOAc 3:7 as eluent to afford **7** in 50% yield (35.0 mg, 0.099 mmol). **^{1}H NMR** (500 MHz, $CDCl_3$) δ (ppm): 7.37 (d, J = 7.2 Hz, 1H), 7.19 – 7.10 (m, 2H), 7.09 – 6.96 (m, 8H), 6.85 – 6.81 (m, 2H), 6.78 (d, J = 7.5 Hz, 1H), 4.23 (d, J = 13.1 Hz, 2H), 3.90 (d, J = 13.2 Hz, 1H), 2.66 (q, J = 9.5 Hz, 1H), 2.49 (s, 3H), 2.47 – 2.38 (m, 1H), 2.07 (dq, J = 13.7, 6.7 Hz, 1H), 1.70 (s, 1H). **^{13}C NMR** (126 MHz, $CDCl_3$) δ (ppm): 143.3 (C), 142.6 (C), 142.1 (C), 141.9 (C), 140.9 (C), 139.6 (C), 131.2 (2xCH), 129.93 (CH), 129.85 (2xCH), 129.6 (CH), 127.8 (2xCH), 127.6 (2xCH), 126.6 (CH), 126.4 (CH), 65.0 (CH), 57.8 (CH_2), 55.8 (CH_2), 45.8 (CH_3), 29.8 (CH_2). **HRMS (APCI-FIA-TOF)** m/z: $[M+H]^+$ Calcd for $C_{25}H_{25}N_2$ 353.2012; found 353.2023. m.p: 144 - 145ºC.

9. NMR Spectra

^{1}H NMR (300 MHz, $CDCl_3$)

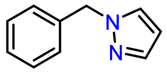

1a

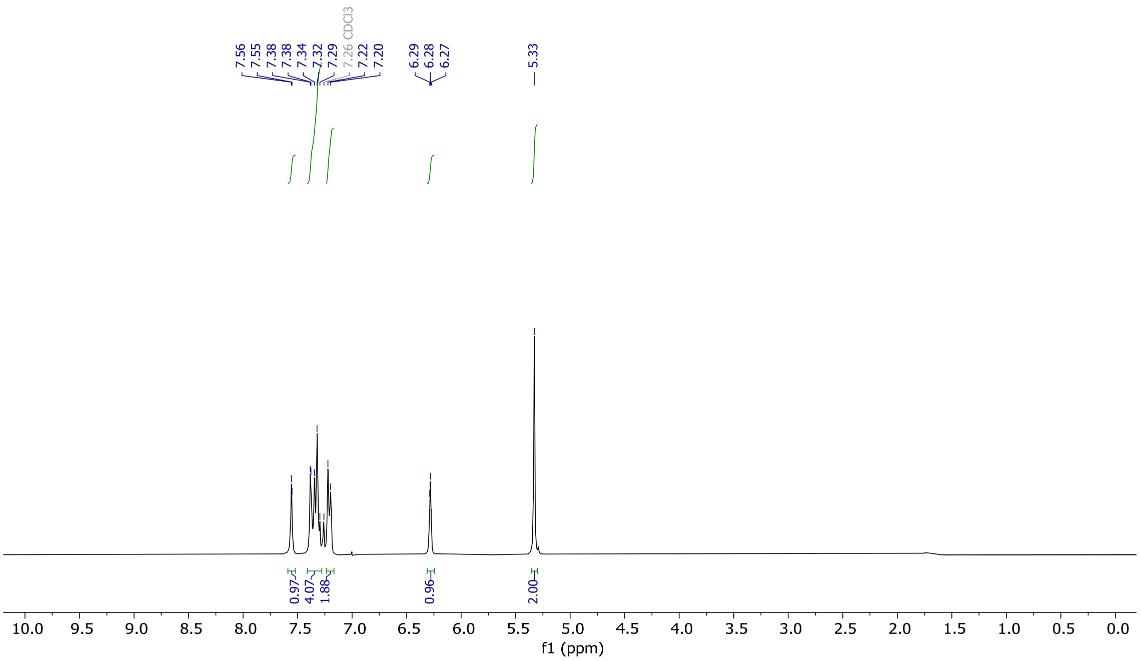

^{1}H NMR (300 MHz, $CDCl_3$)

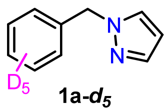

1a-*d₅*

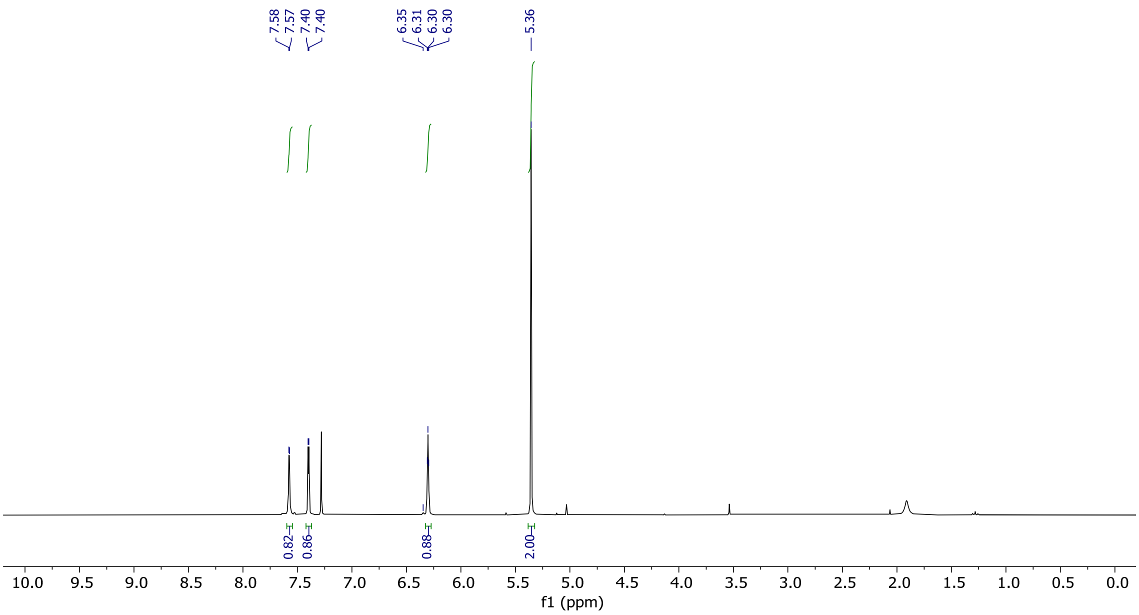

^{13}C NMR (75 MHz, $CDCl_3$)

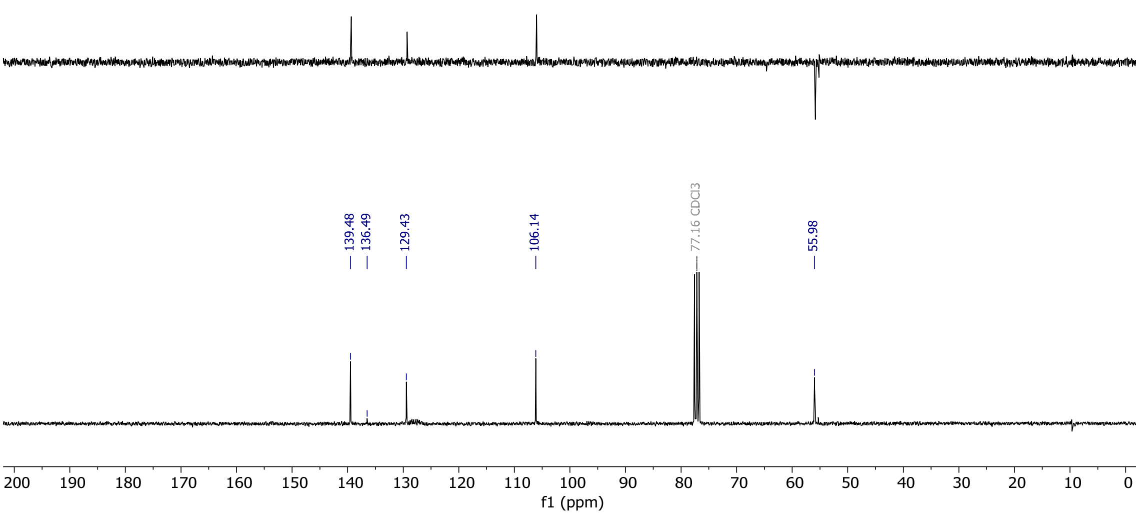

^{1}H NMR (300 MHz, $CDCl_3$)

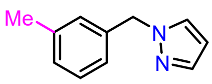

1b

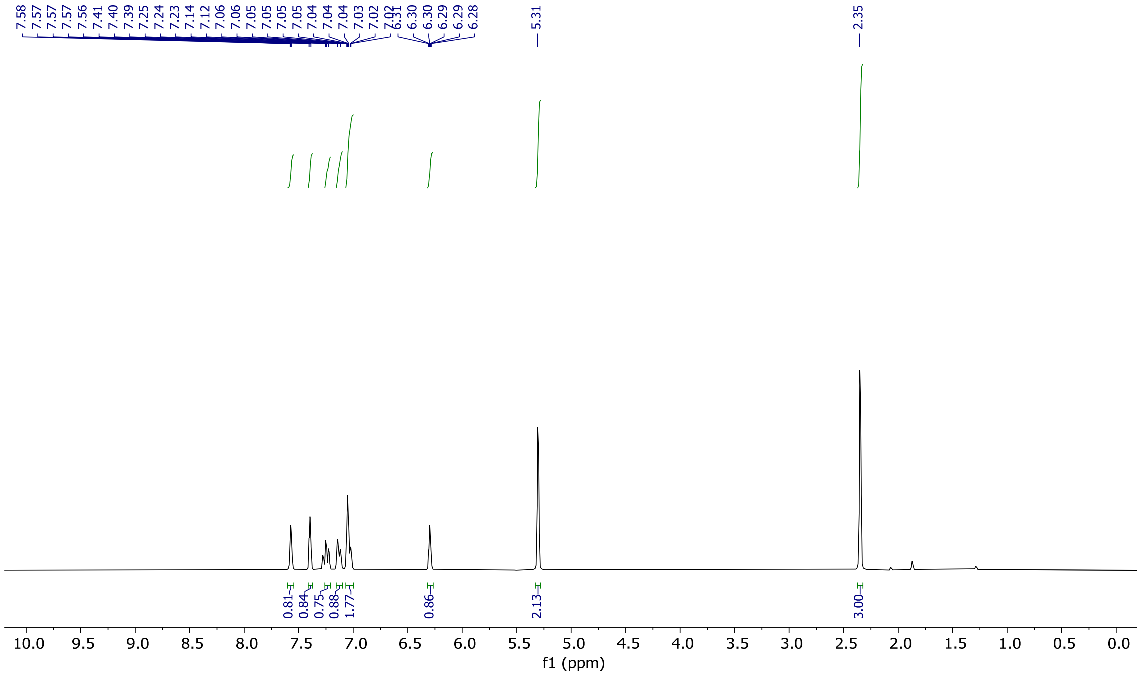

^{1}H NMR (300 MHz, $CDCl_3$)

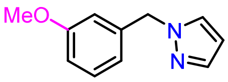

1c

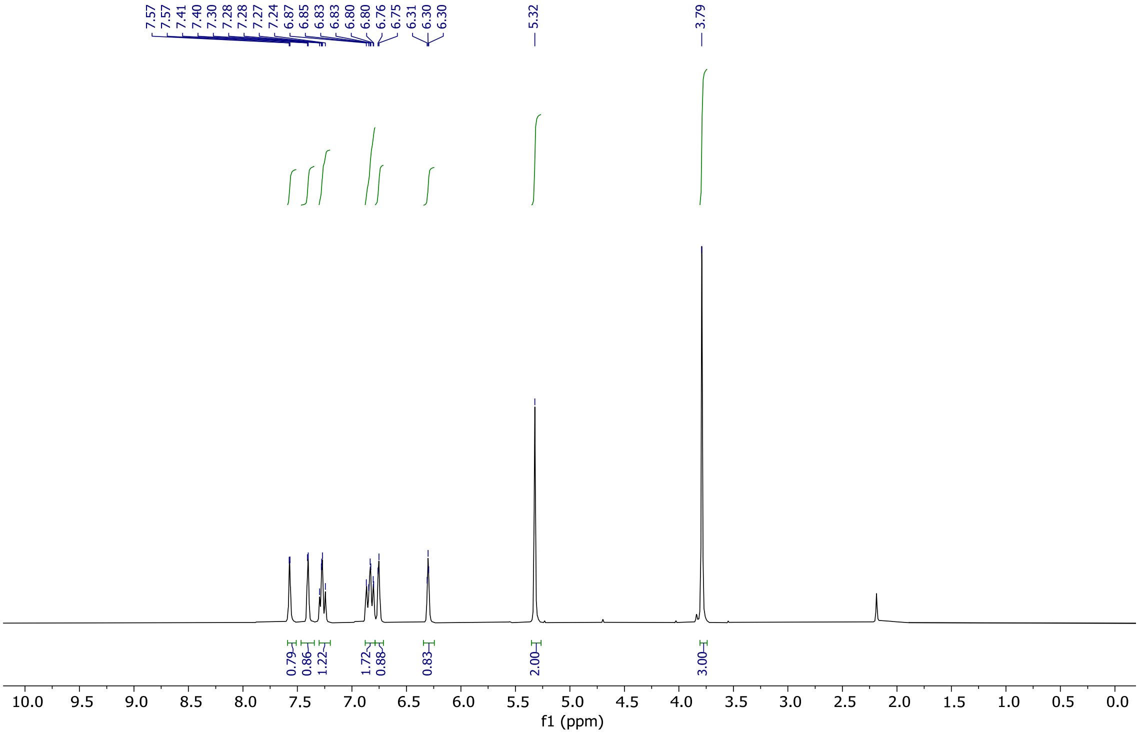

^{13}C NMR (75 MHz, $CDCl_3$)

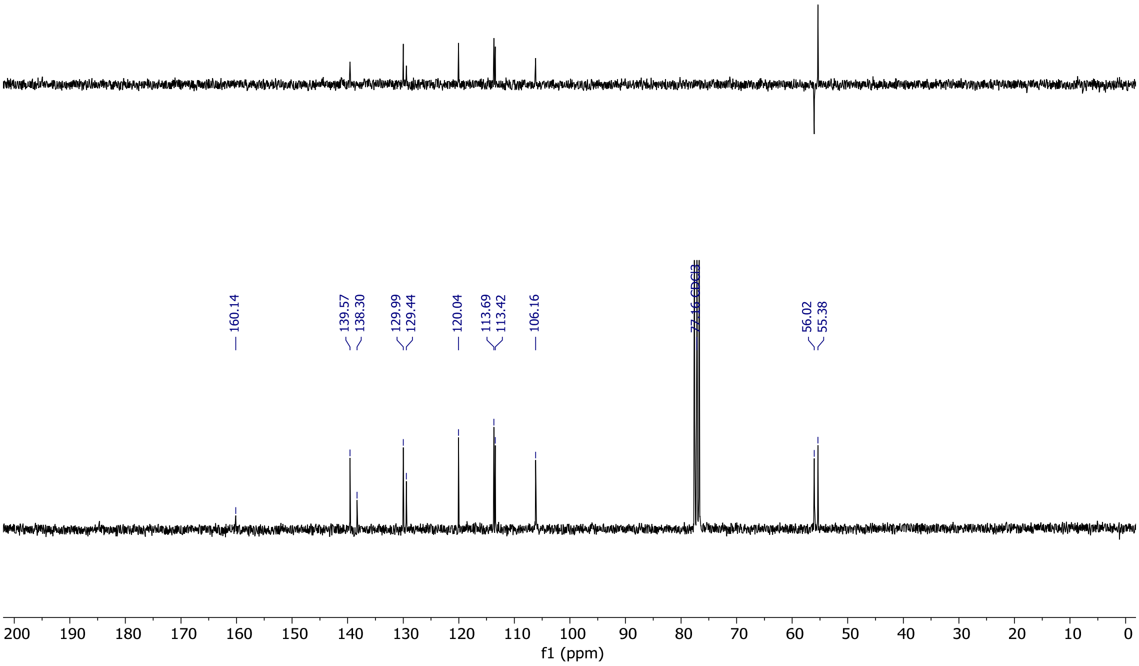

^{1}H NMR (300 MHz, $CDCl_3$)

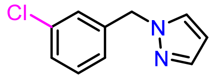

1d

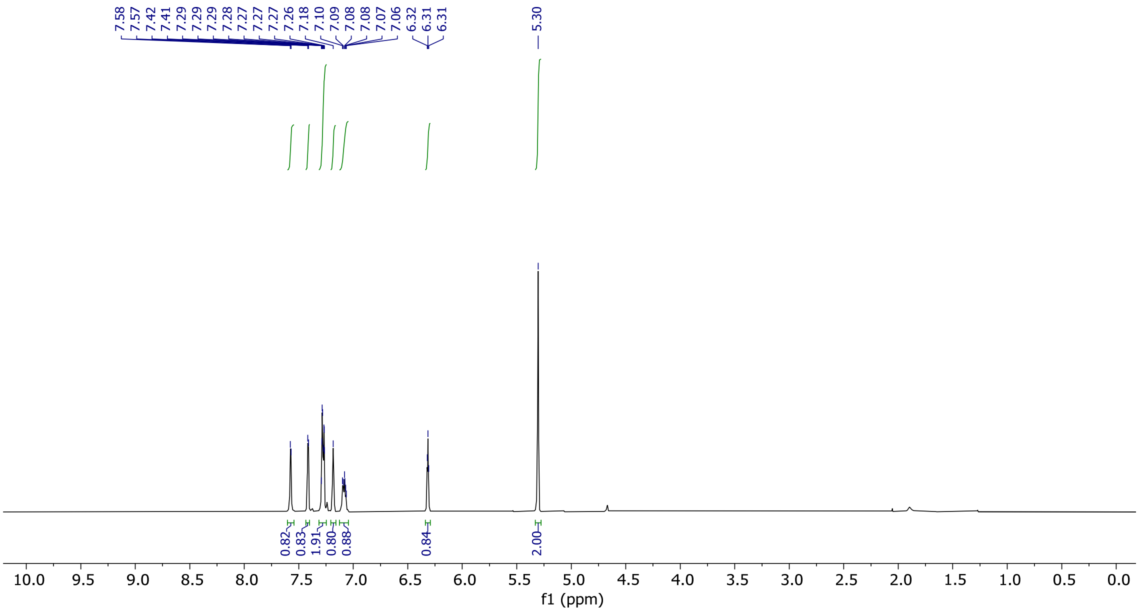

^{13}C NMR (75 MHz, $CDCl_3$)

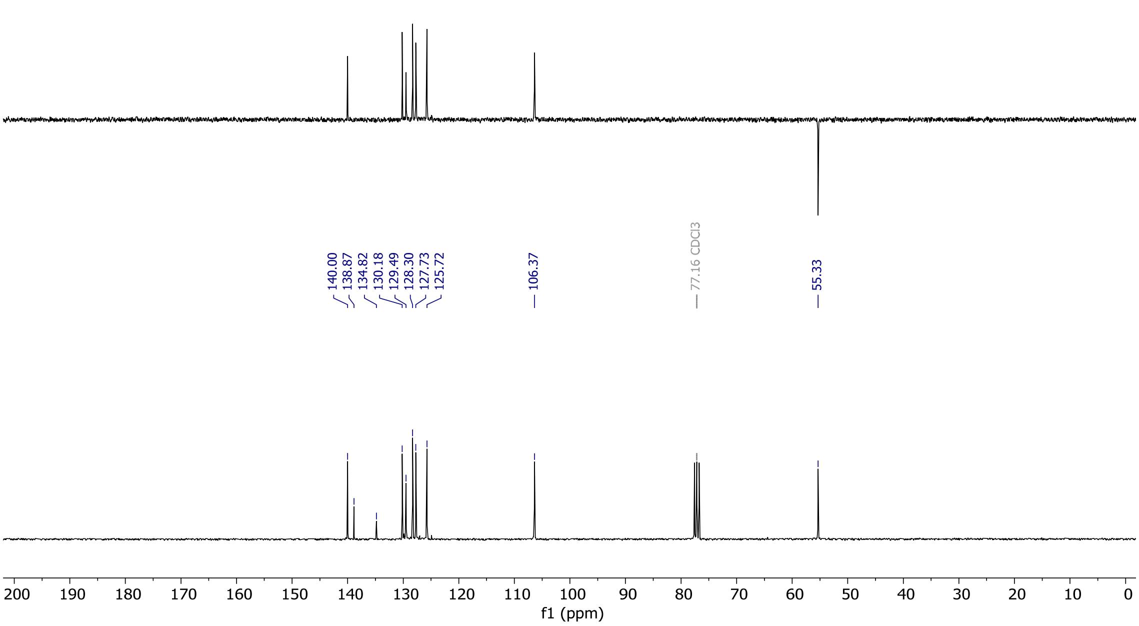

^{1}H NMR (300 MHz, $CDCl_3$)

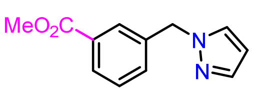

1e

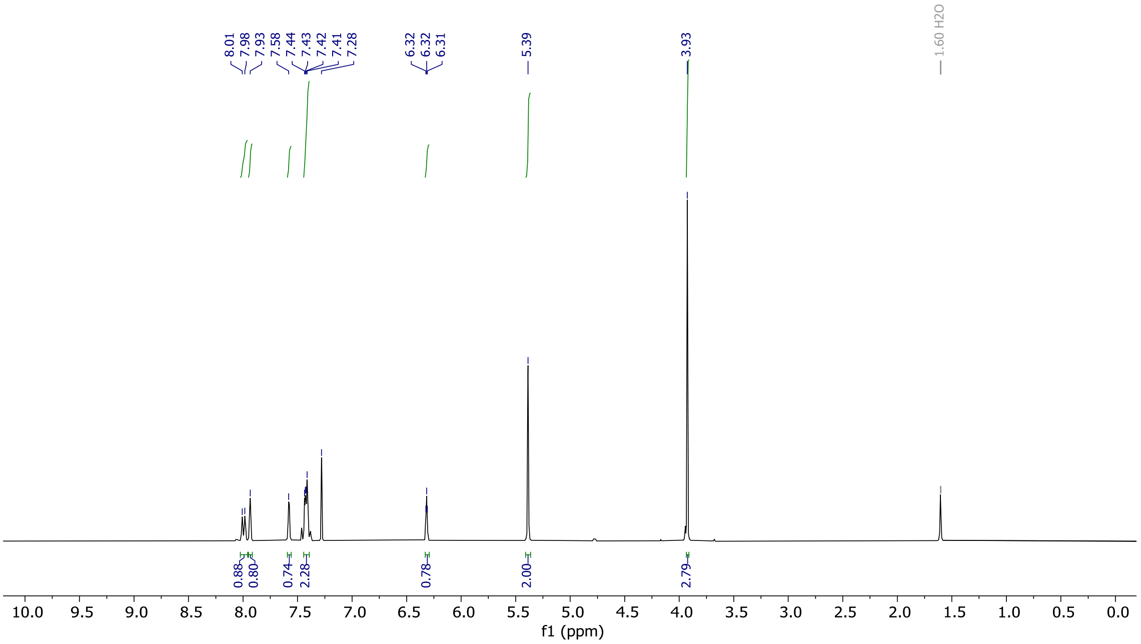

^{1}H NMR (300 MHz, $CDCl_3$)

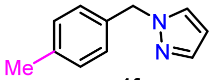

1f

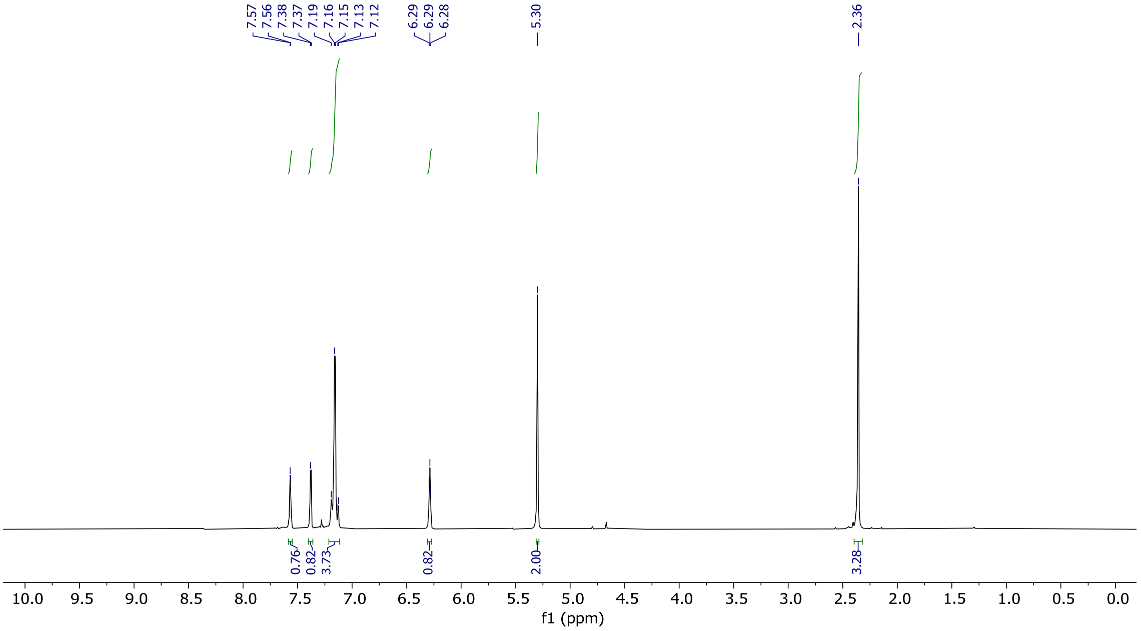

^{1}H NMR (300 MHz, CDCl$_3$)

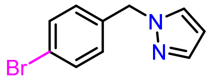

1g

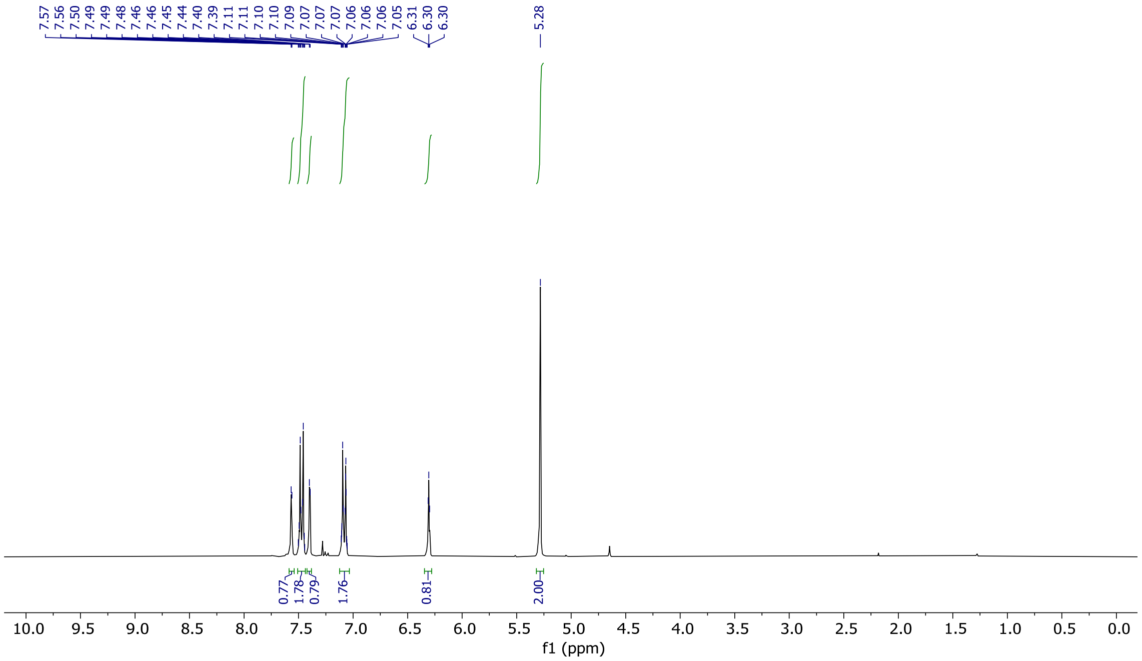

^{13}C NMR (75 MHz, CDCl$_3$)

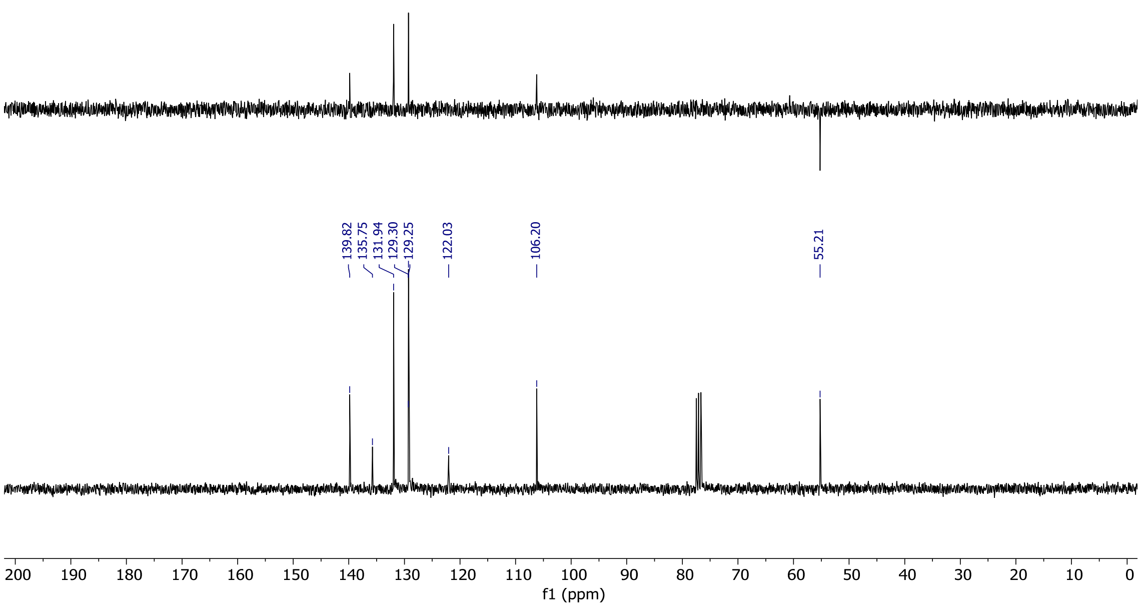

^{1}H NMR (300 MHz, $CDCl_3$)

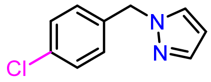

1h

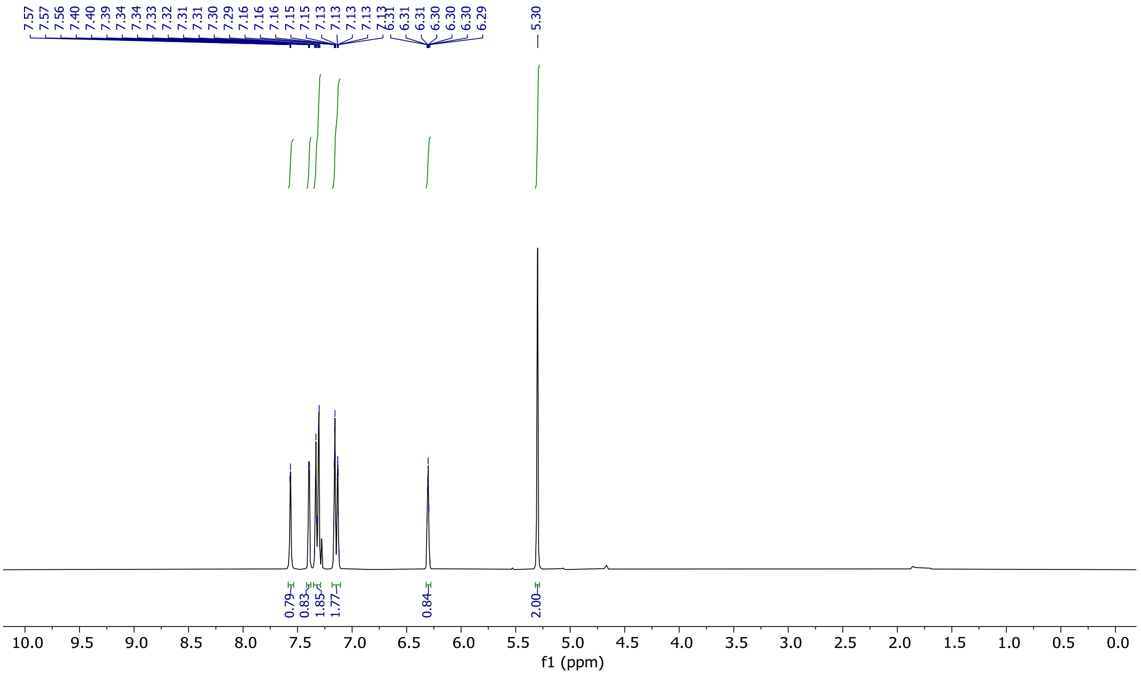

^{13}C NMR (75 MHz, $CDCl_3$)

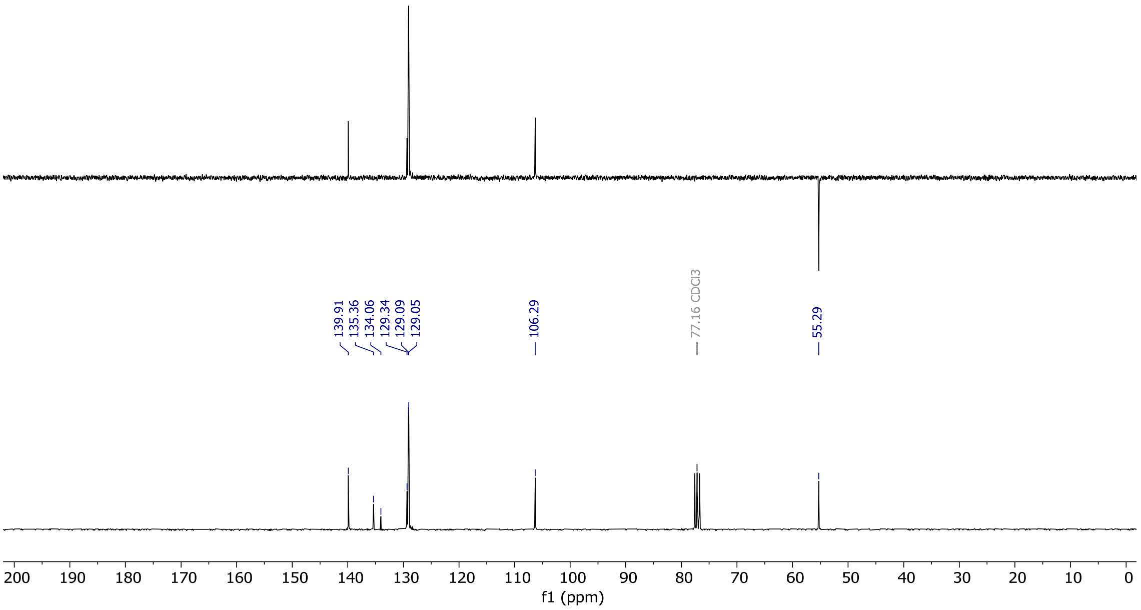

^{1}H NMR (300 MHz, $CDCl_3$)

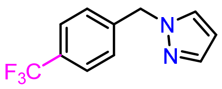

1i

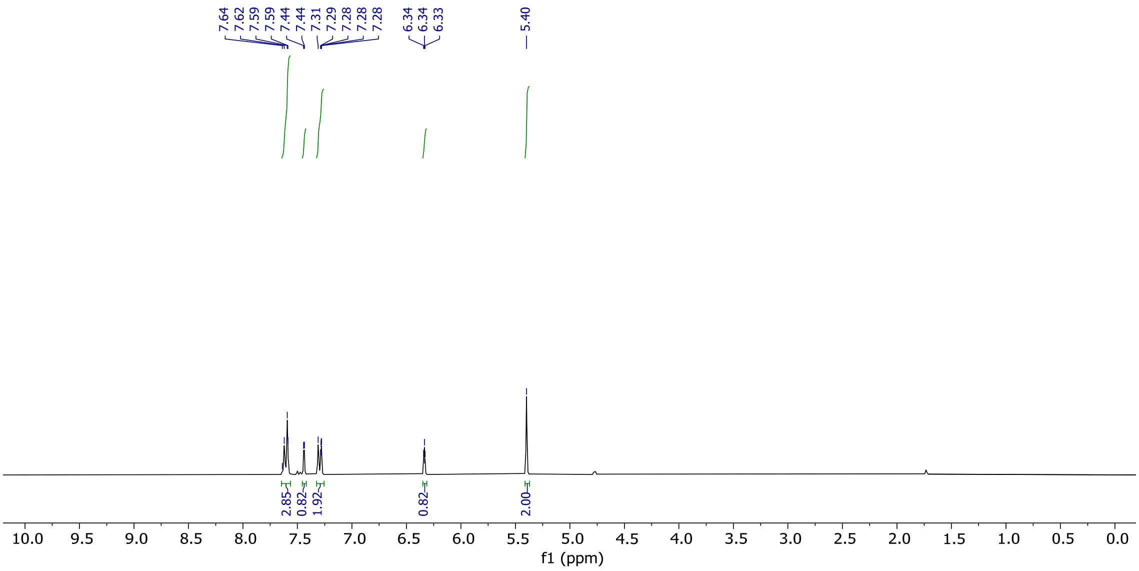

^{13}C NMR (75 MHz, $CDCl_3$)

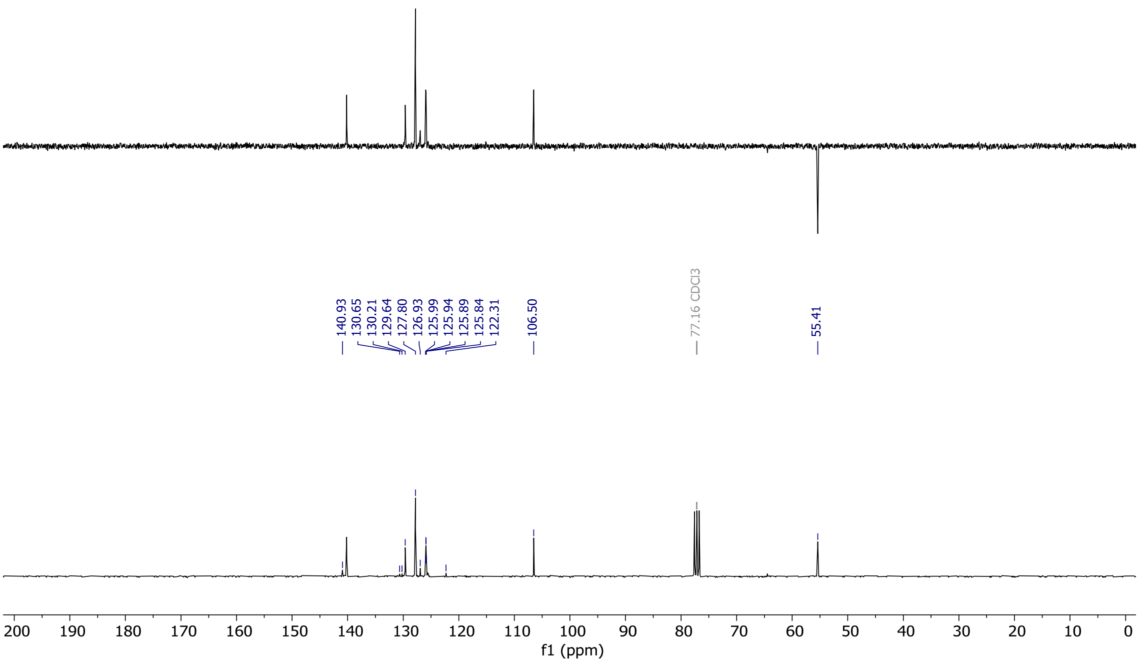

^{19}F NMR (282 MHz, CDCl$_3$)

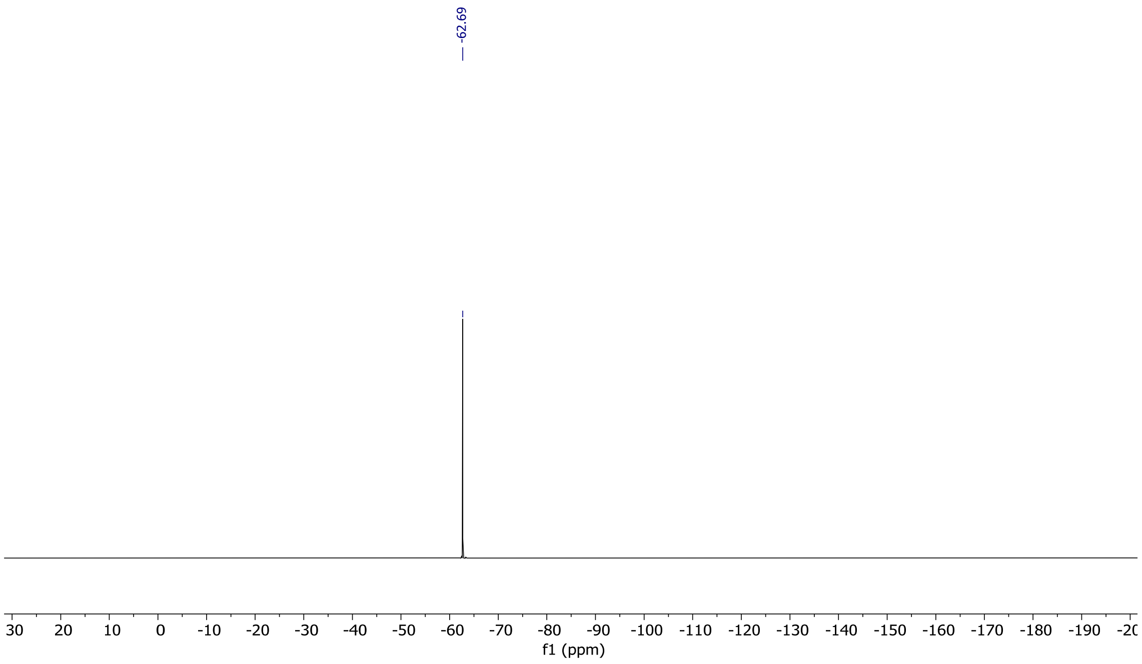

^{1}H NMR (300 MHz, $CDCl_3$)

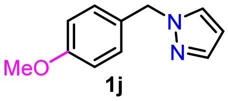

1j

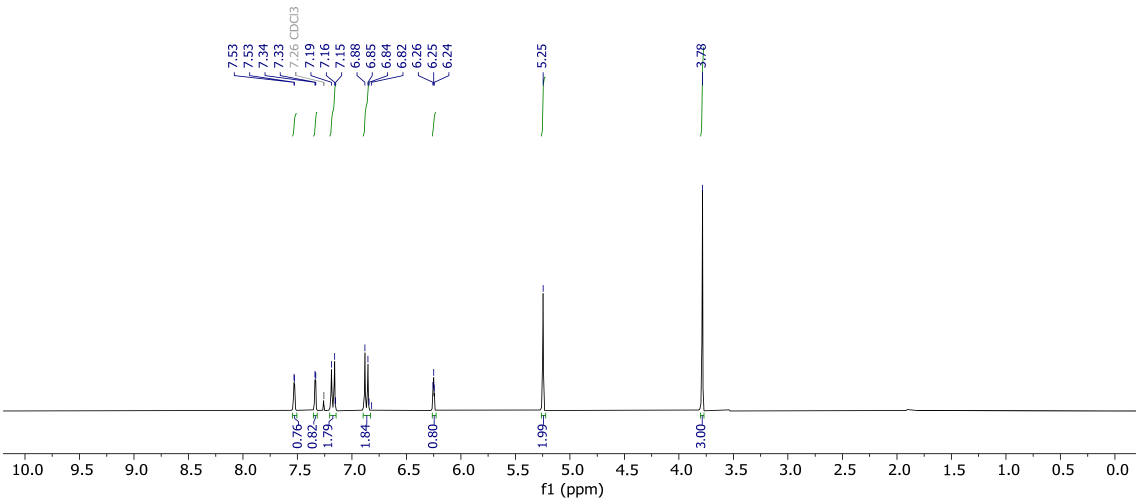

^{13}C NMR (75 MHz, $CDCl_3$)

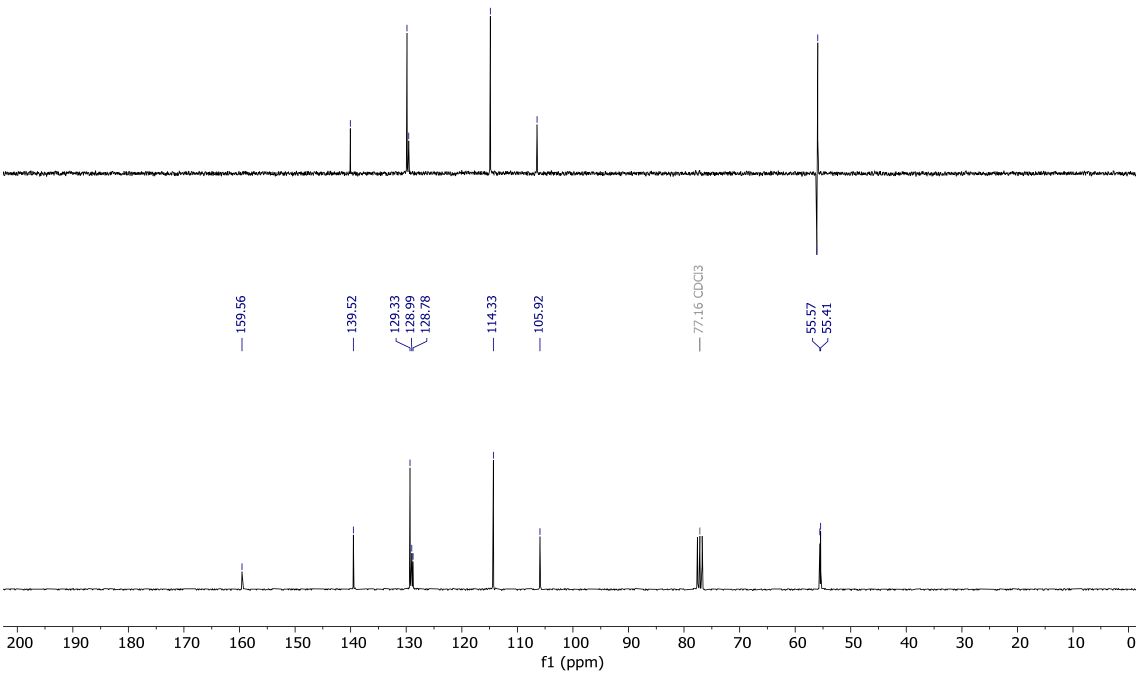

^{1}H NMR (300 MHz, $CDCl_3$)

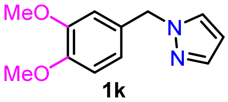

1k

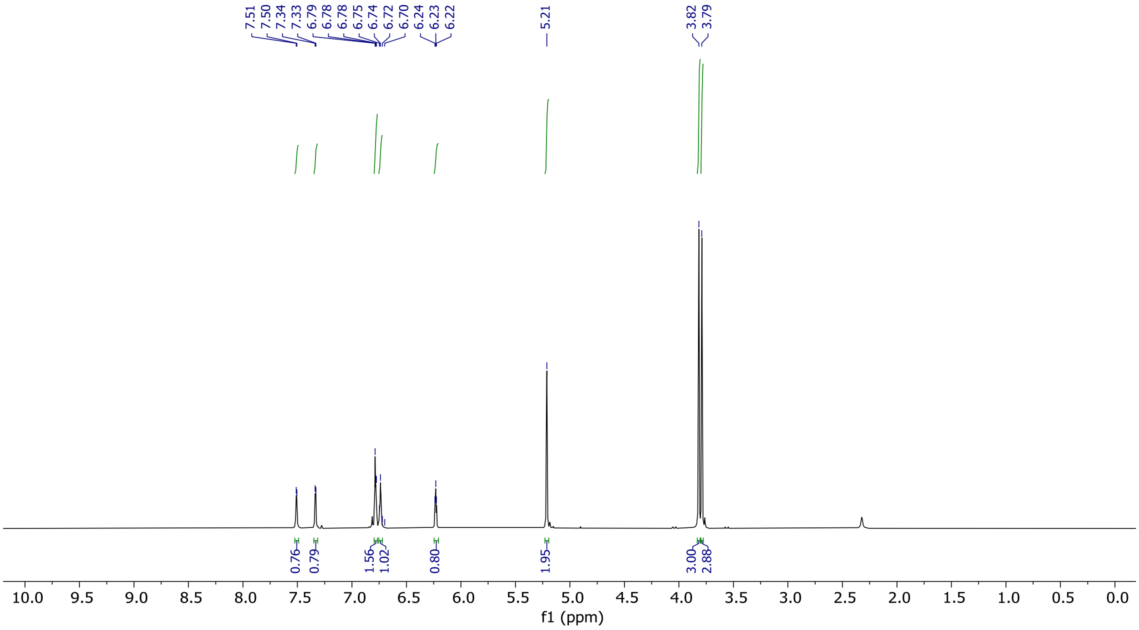

^{13}C NMR (75 MHz, $CDCl_3$)

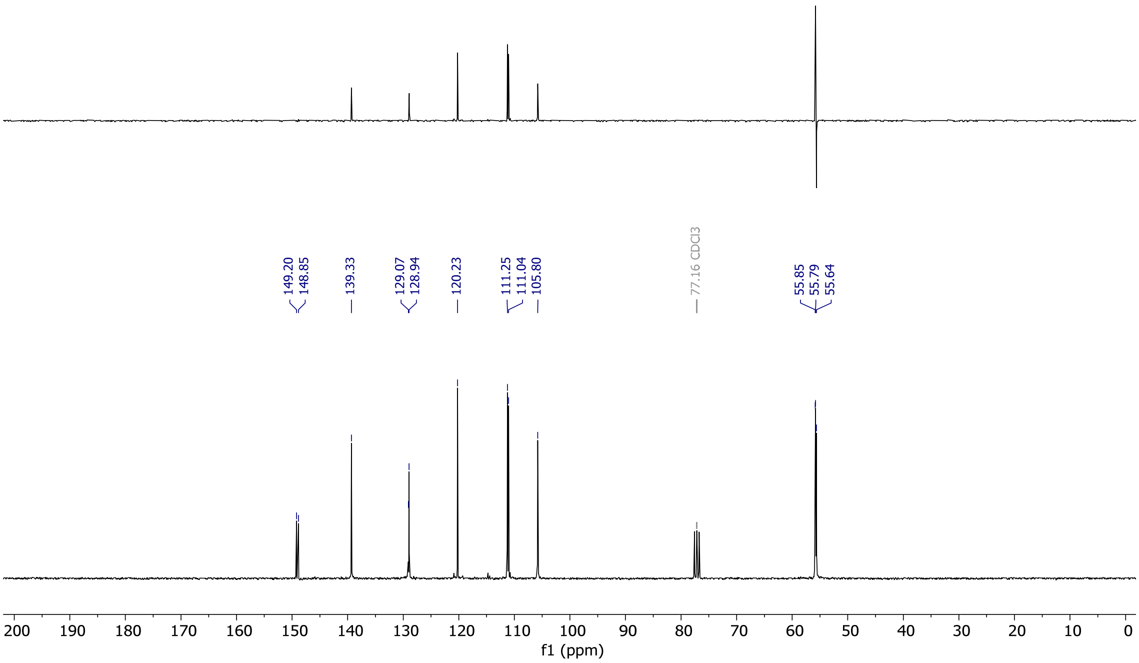

^{1}H NMR (300 MHz, $CDCl_3$)

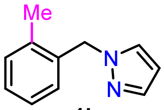

1l

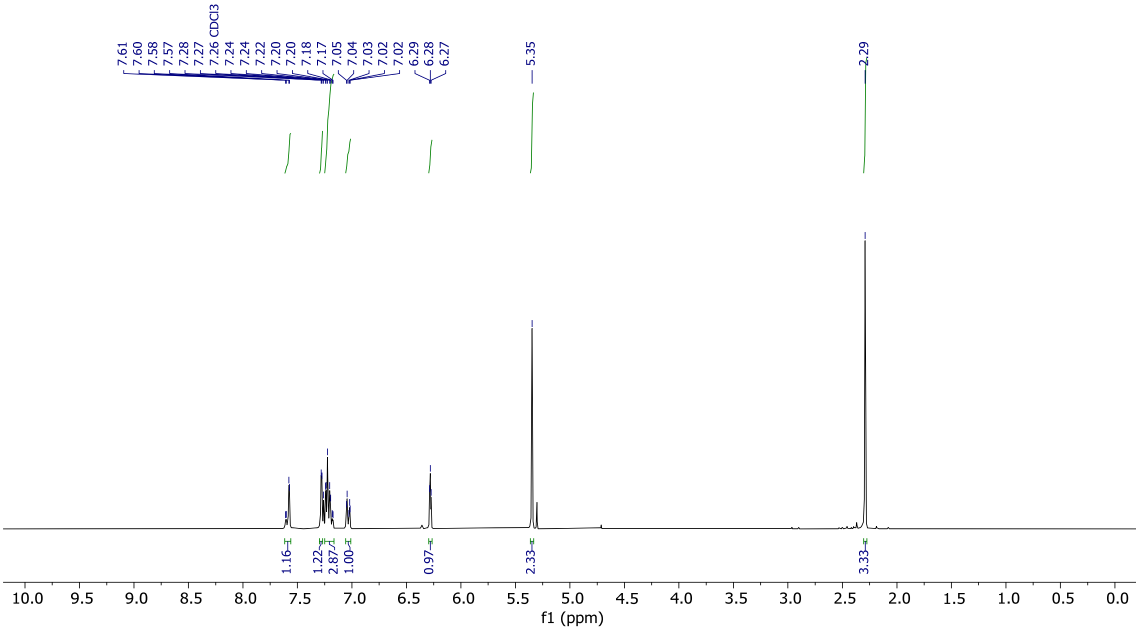

^{13}C NMR (75 MHz, $CDCl_3$)

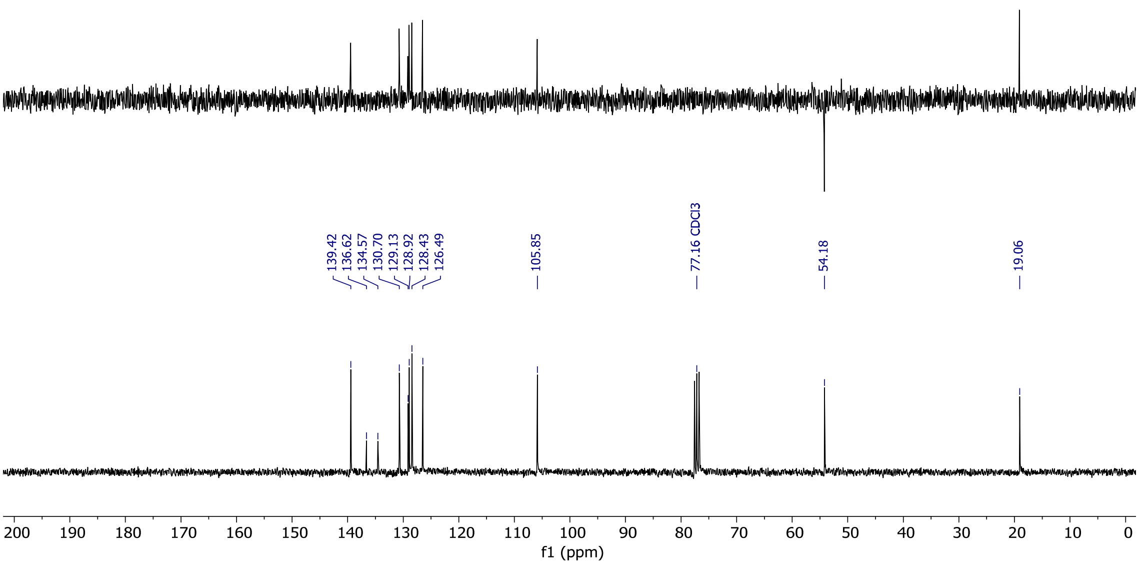

^1H NMR (300 MHz, $CDCl_3$)

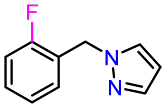

1m

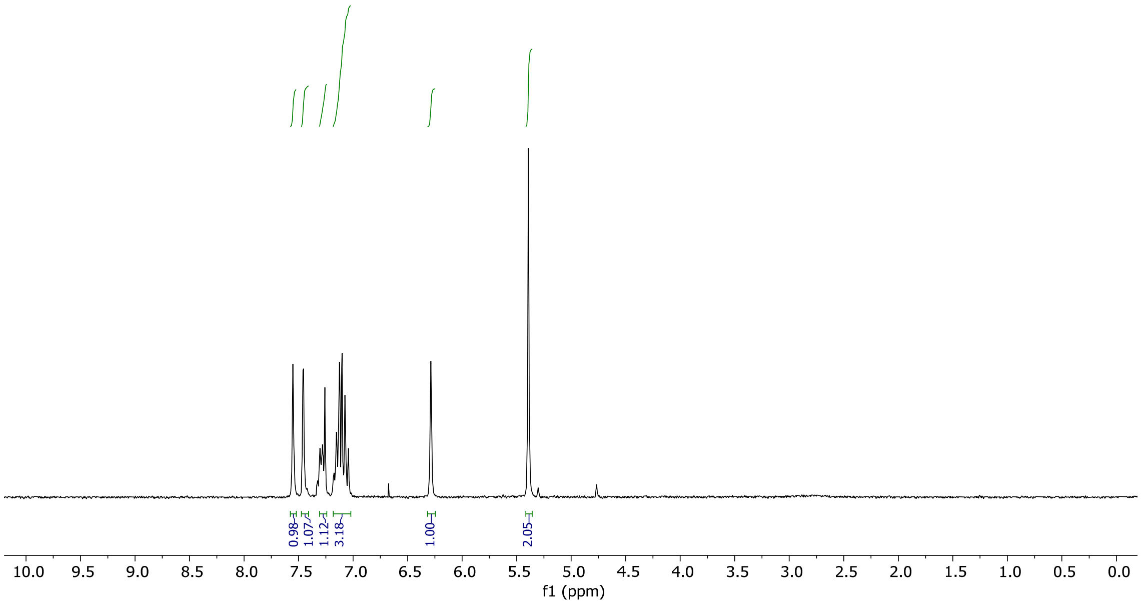

^{13}C NMR (75 MHz, $CDCl_3$)

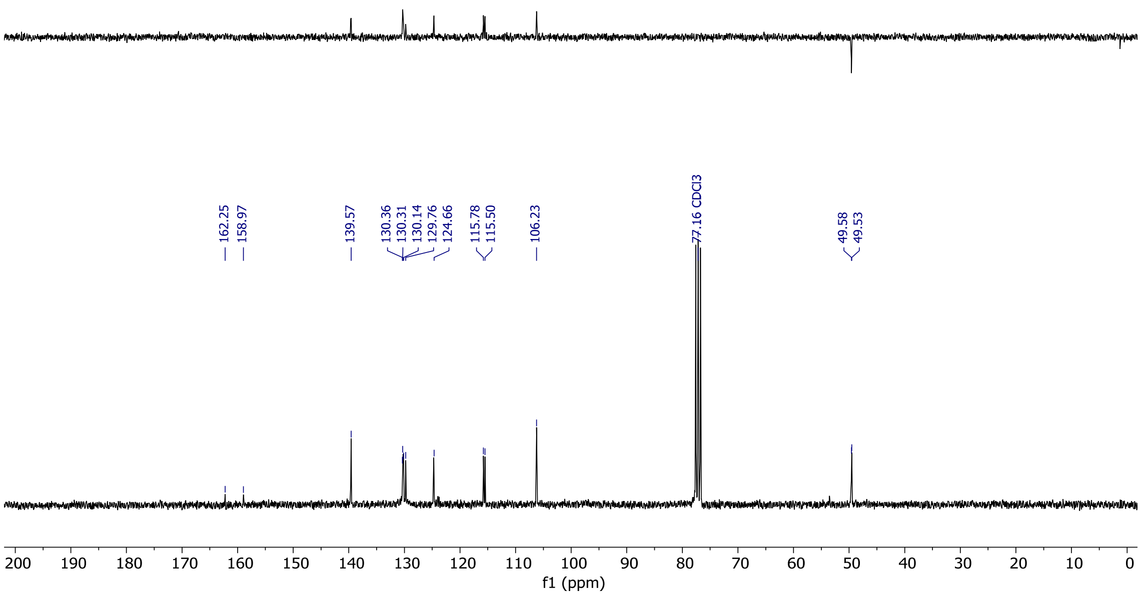

^{1}H NMR (500 MHz, CDCl3)

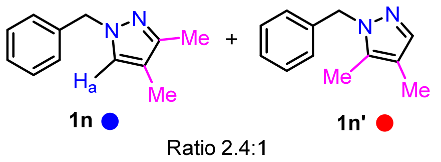

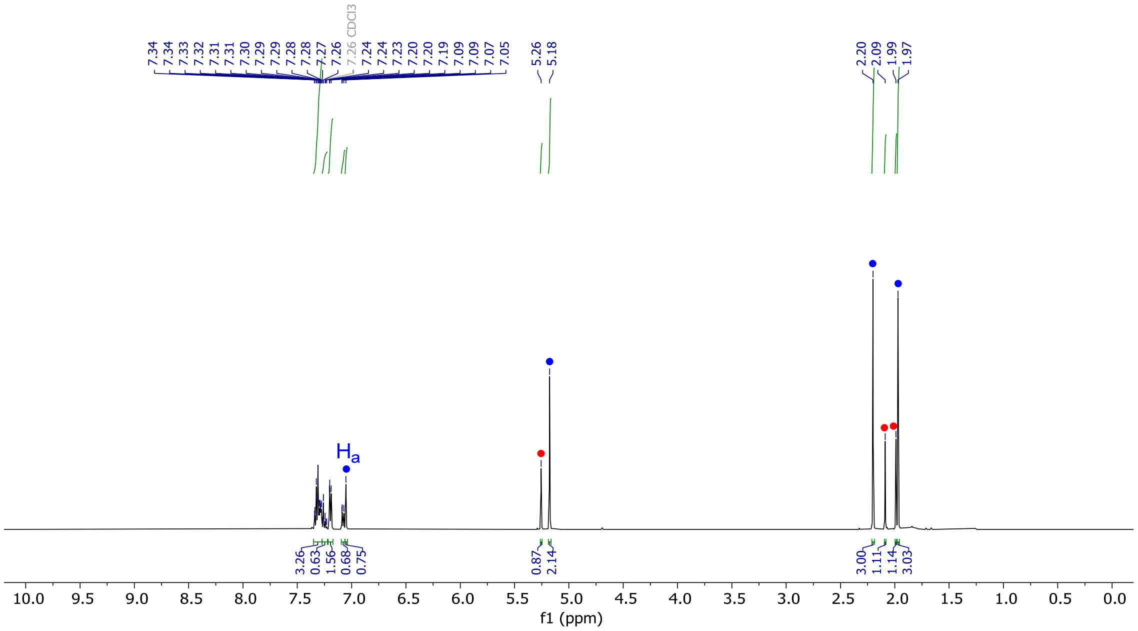

^{13}C NMR (126 MHz, CDCl3)

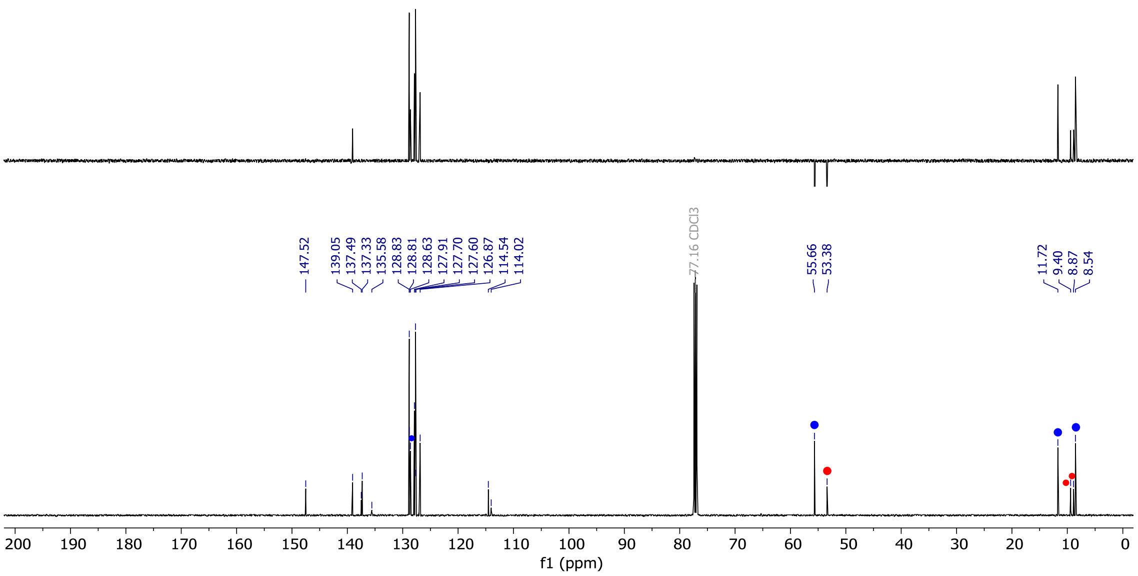

^{1}H NMR (300 MHz, $CDCl_3$)

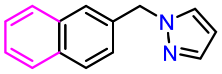

1o

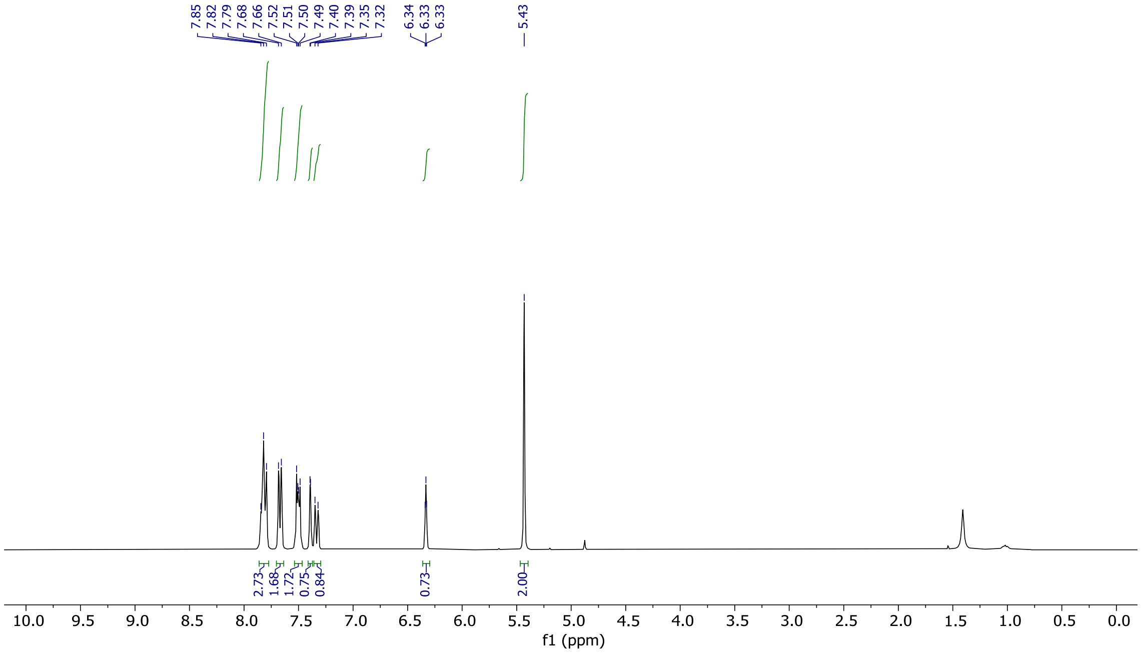

^{1}H NMR (300 MHz, $CDCl_3$)

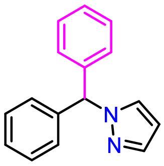

1p

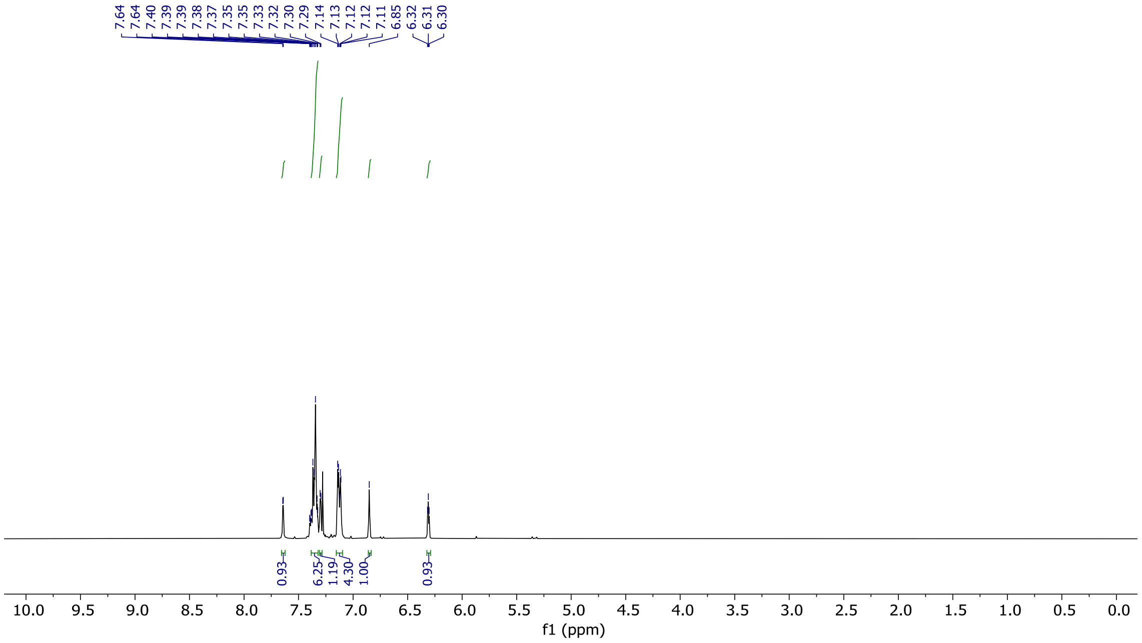

^{1}H NMR (300 MHz, $CDCl_3$)

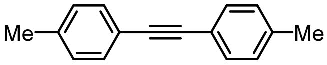

2b

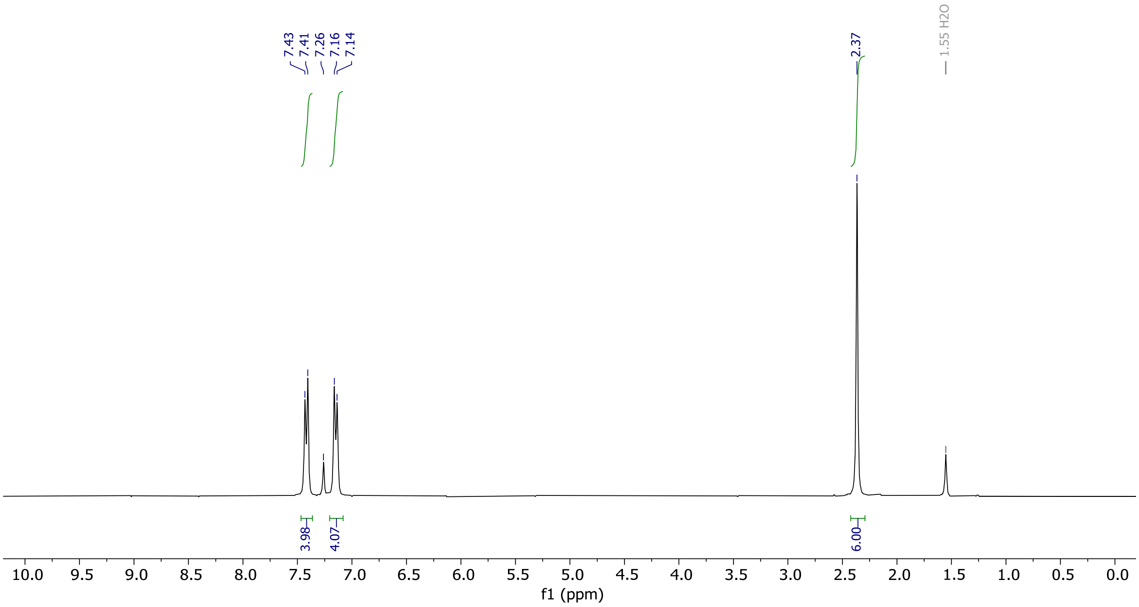

^{1}H NMR (300 MHz, $CDCl_3$)

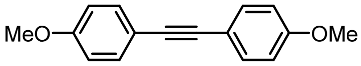

2c

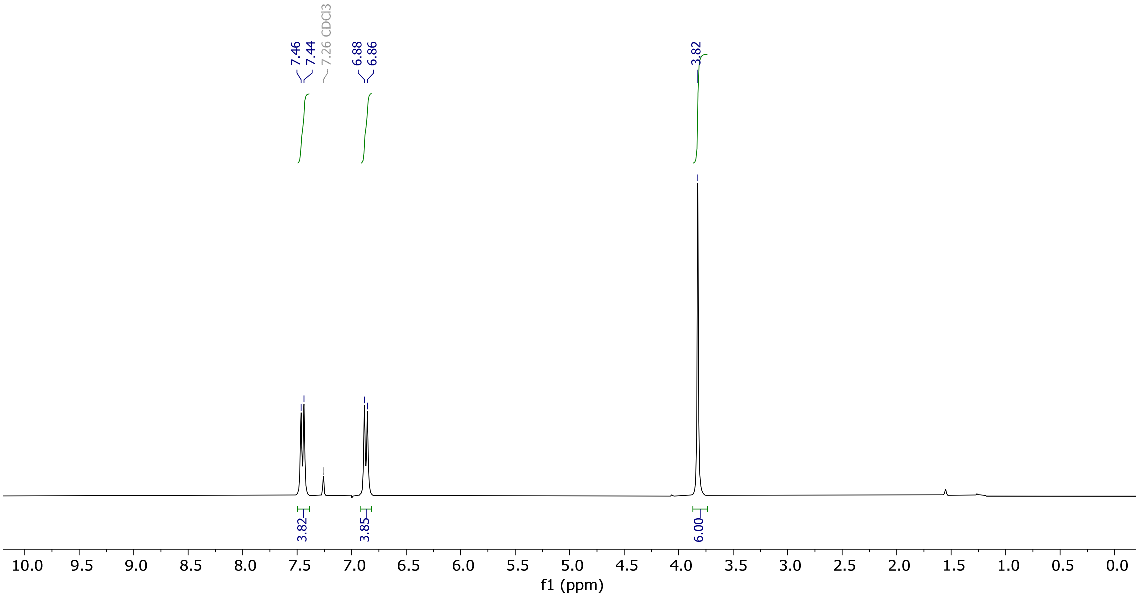

^{1}H NMR (300 MHz, $CDCl_3$)

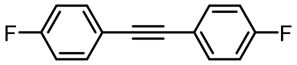

2d

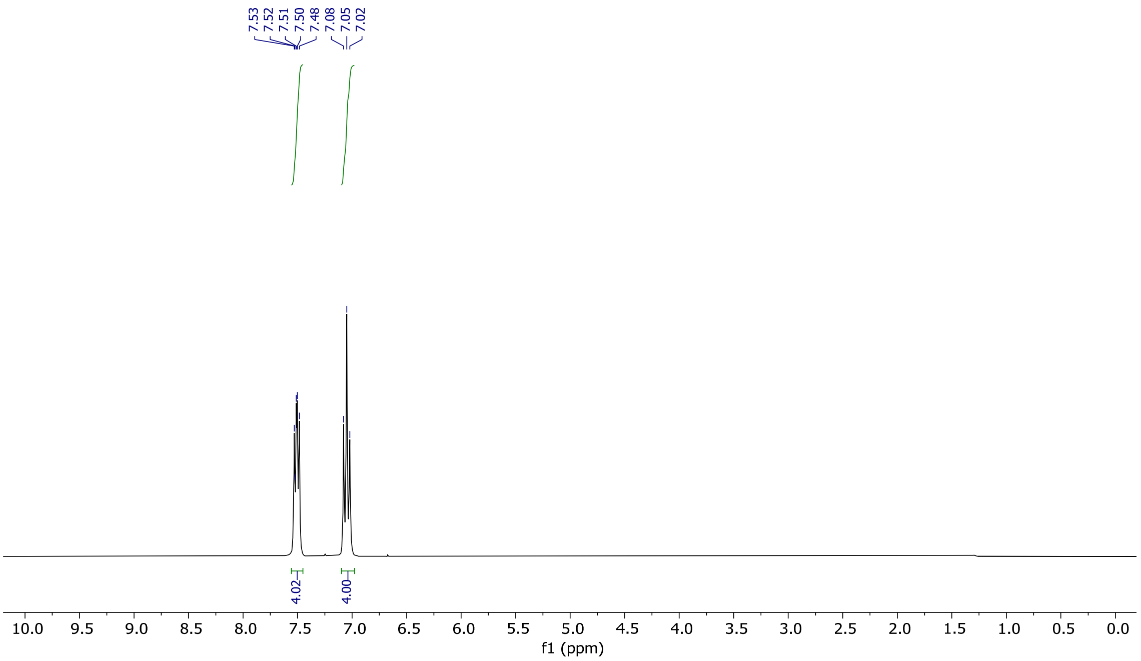

^{1}H NMR (300 MHz, $CDCl_3$)

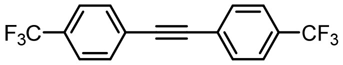

2e

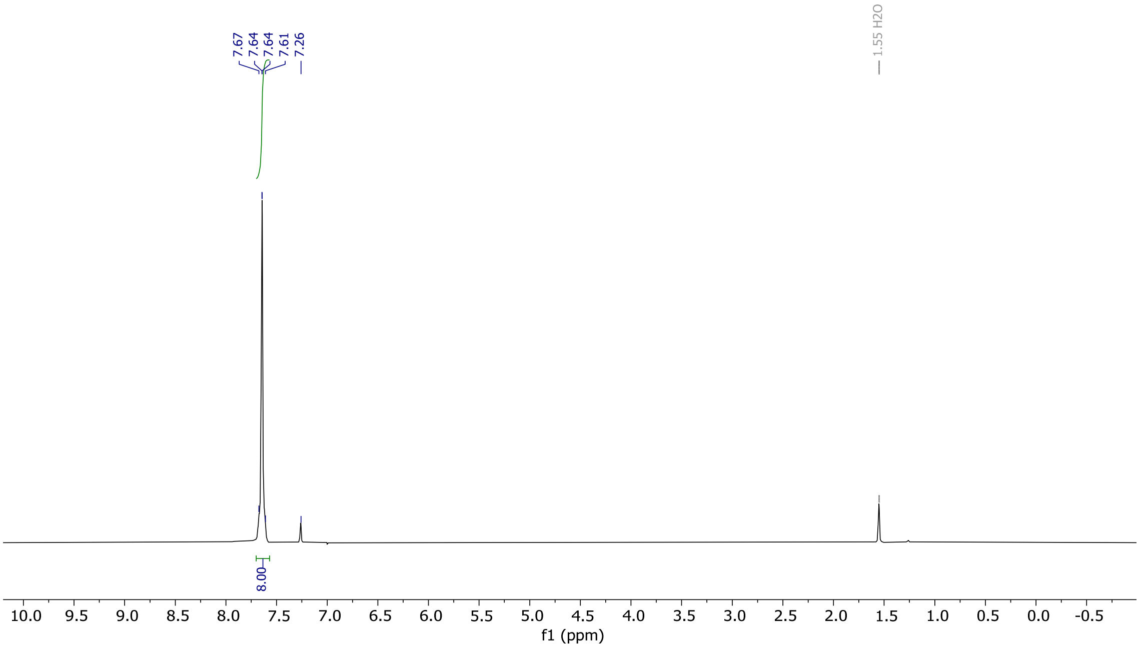

^{1}H NMR (300 MHz, $CDCl_3$)

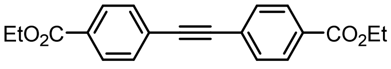

2f

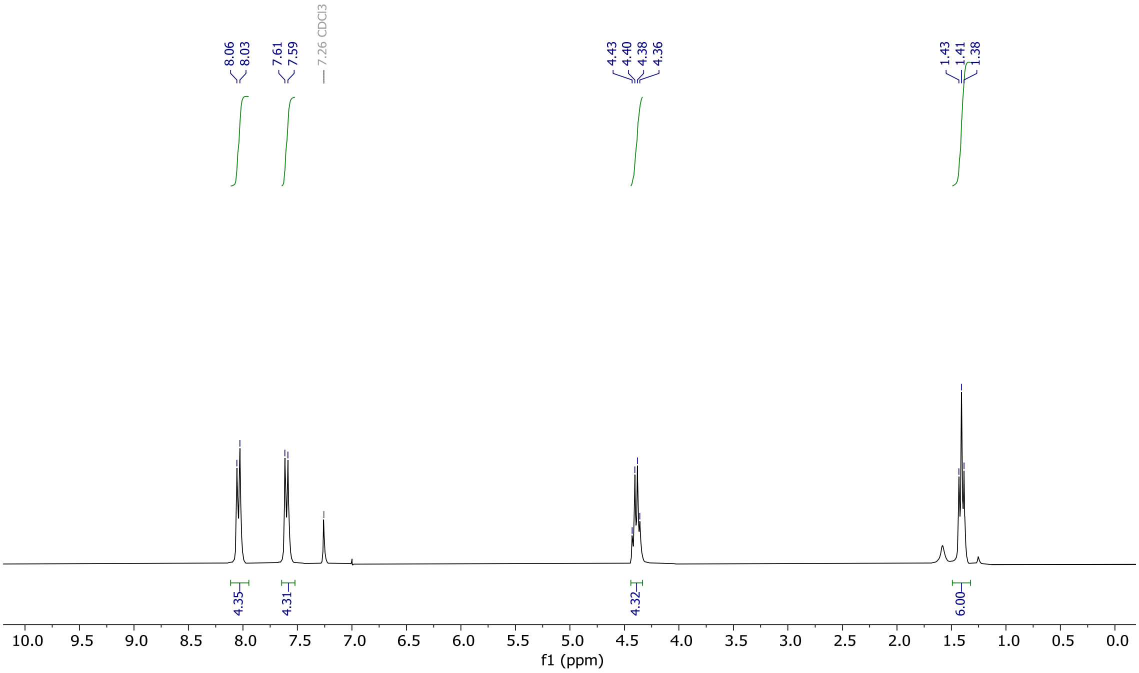

^{1}H NMR (300 MHz, $CDCl_3$)

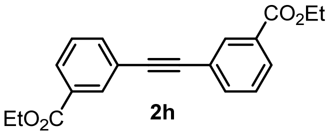

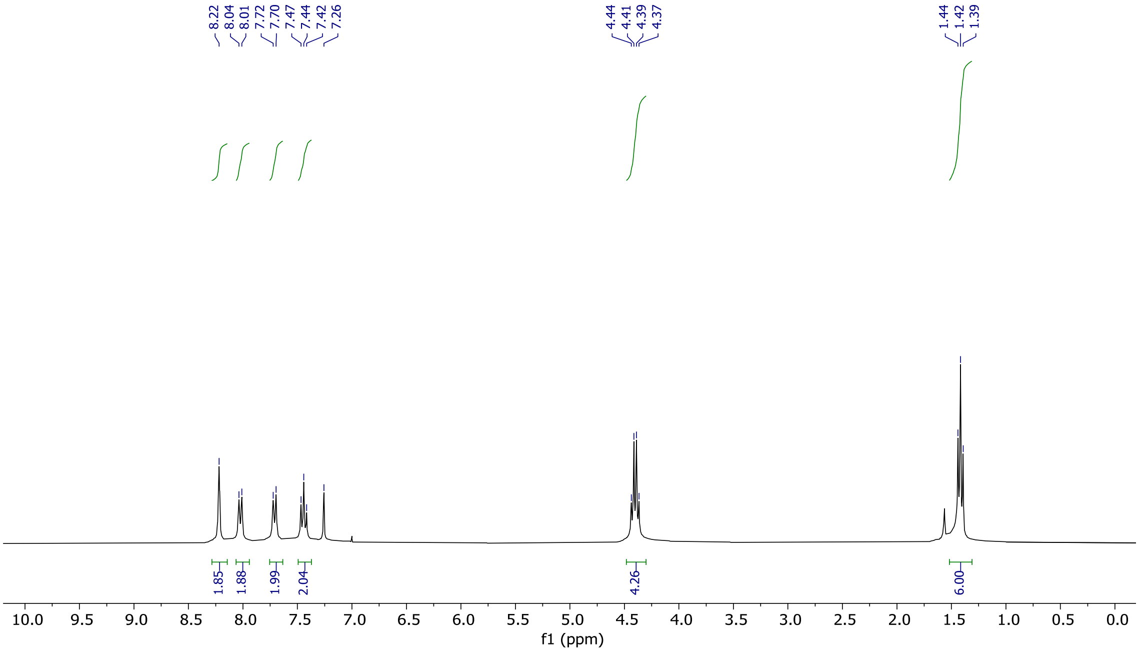

^{1}H NMR (300 MHz, $CDCl_3$)

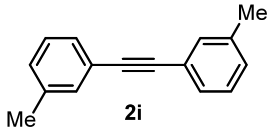

2i

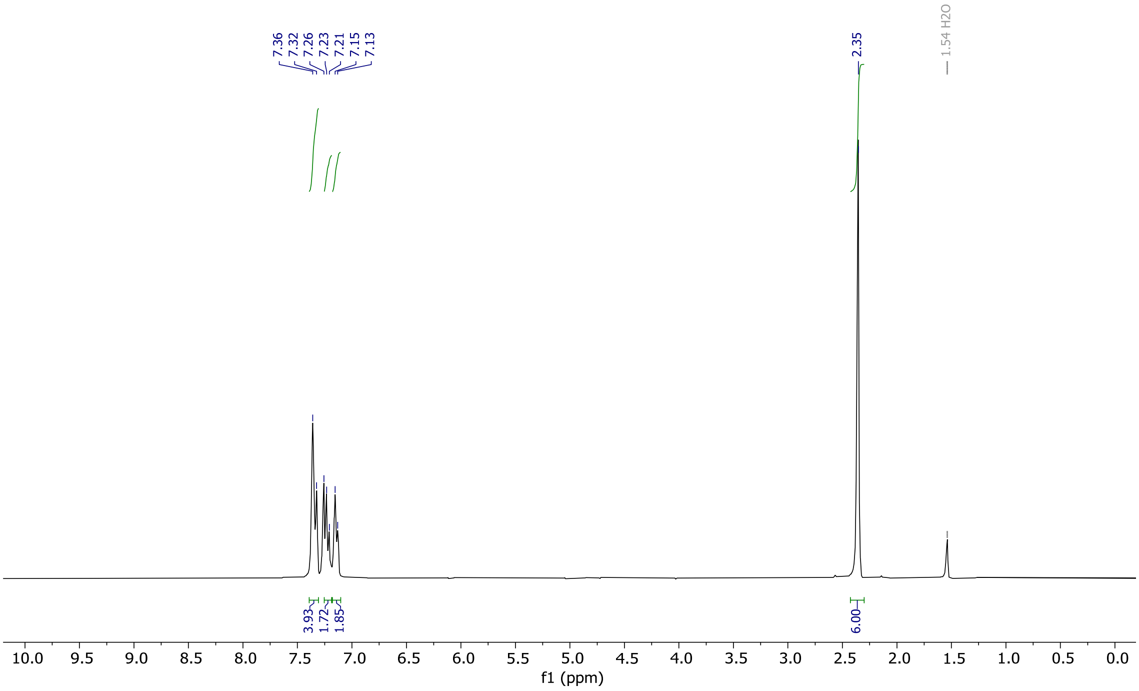

^{1}H NMR (300 MHz, $CDCl_3$)

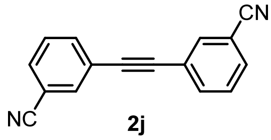

2j

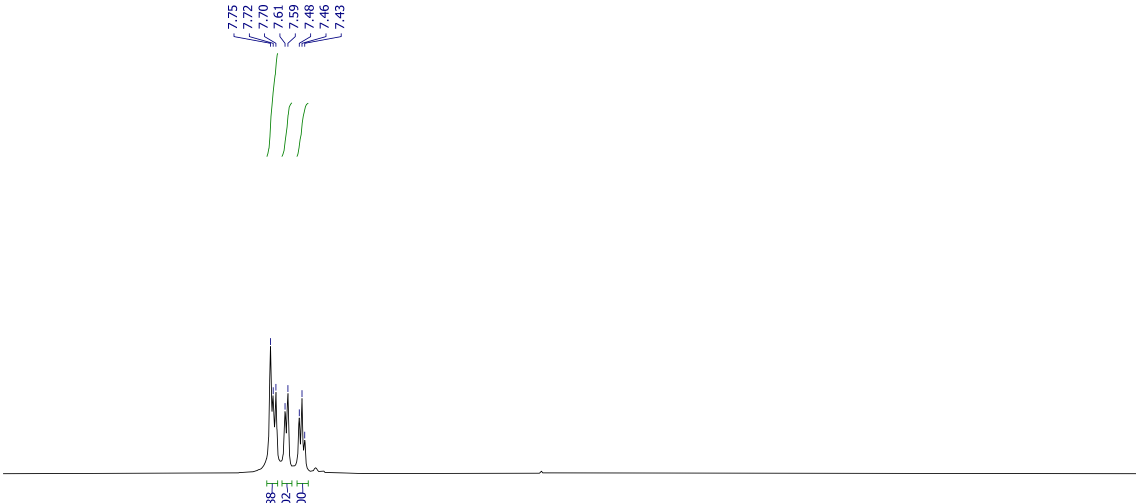

^{1}H NMR (300 MHz, $CDCl_3$)

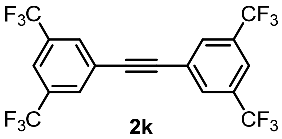

2k

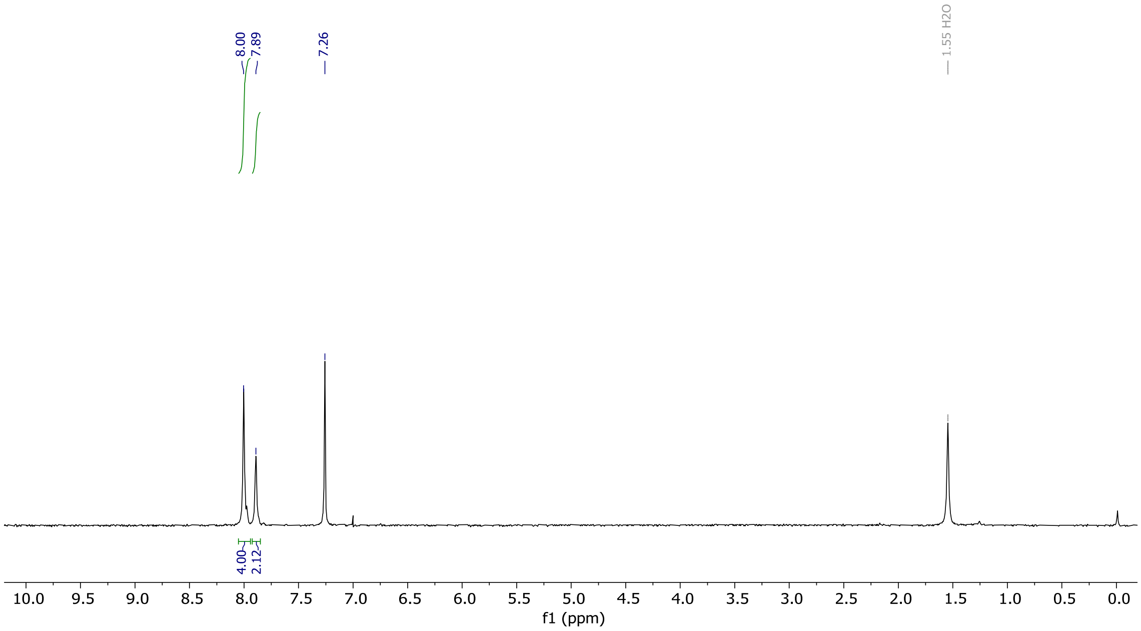

^{1}H NMR (300 MHz, $CDCl_3$)

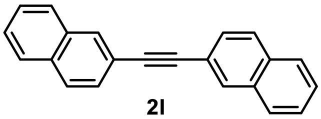

2l

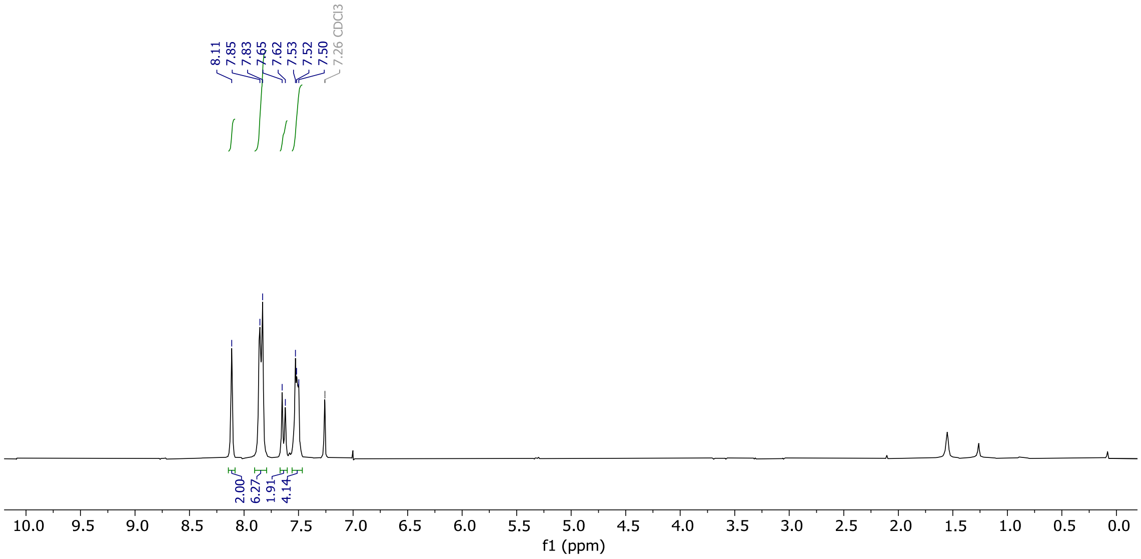

^{1}H NMR (300 MHz, CDCl$_3$)

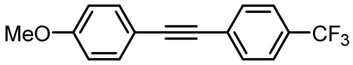

2m

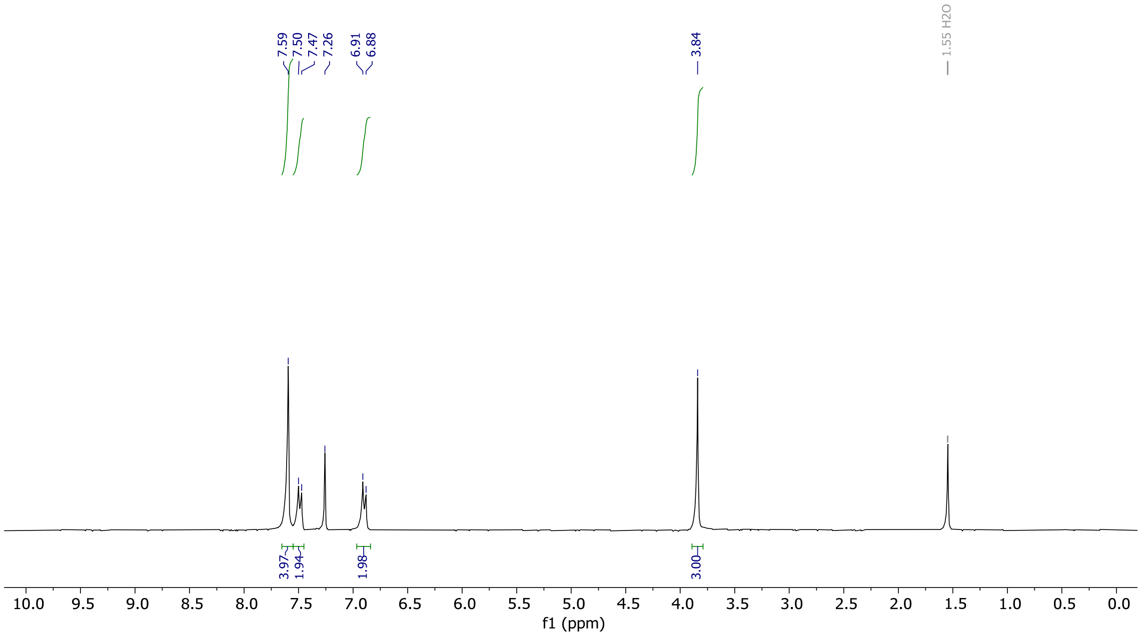

^{1}H NMR (500 MHz, $CDCl_3$)

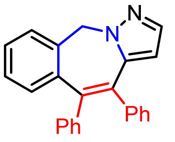

3aa

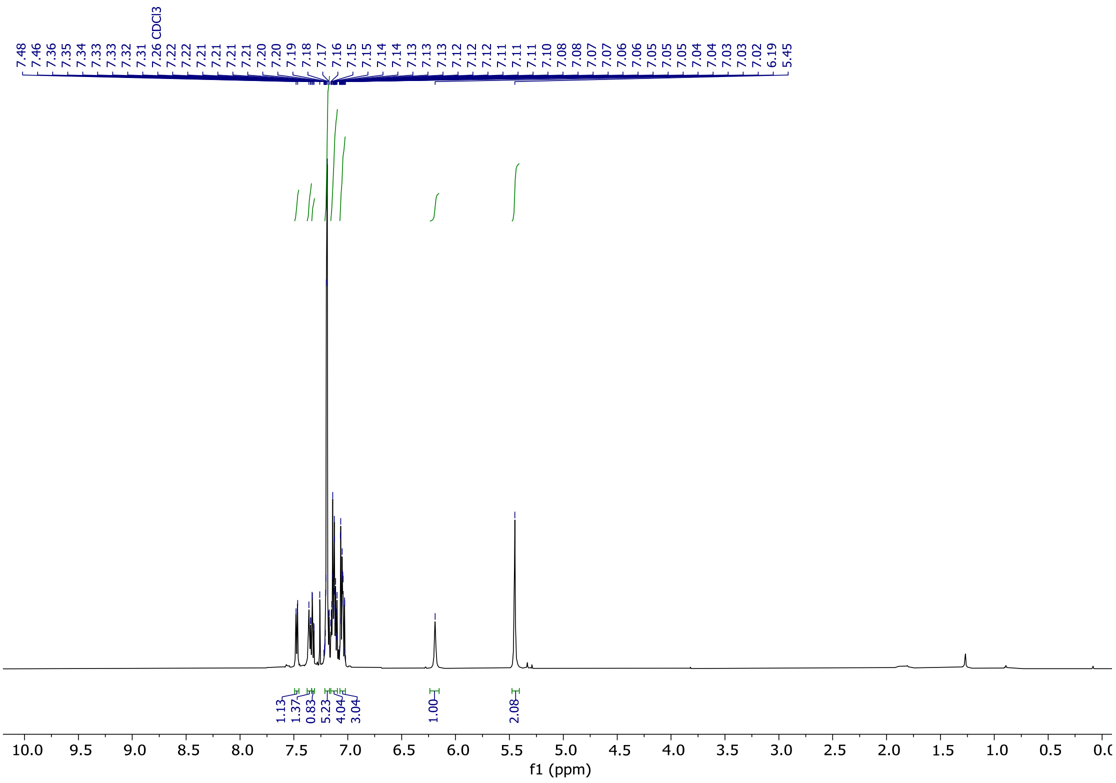

^{13}C NMR (126 MHz, $CDCl_3$)

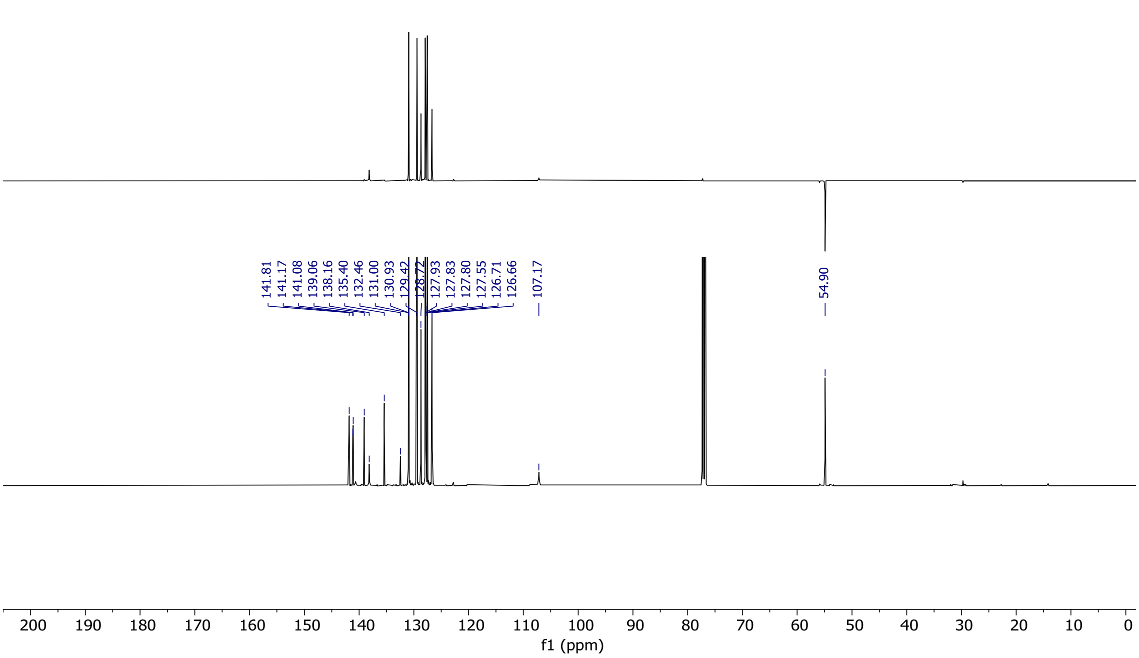

^{1}H NMR (500 MHz, $CDCl_3$)

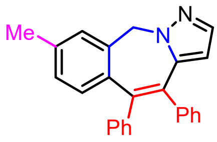

3ba

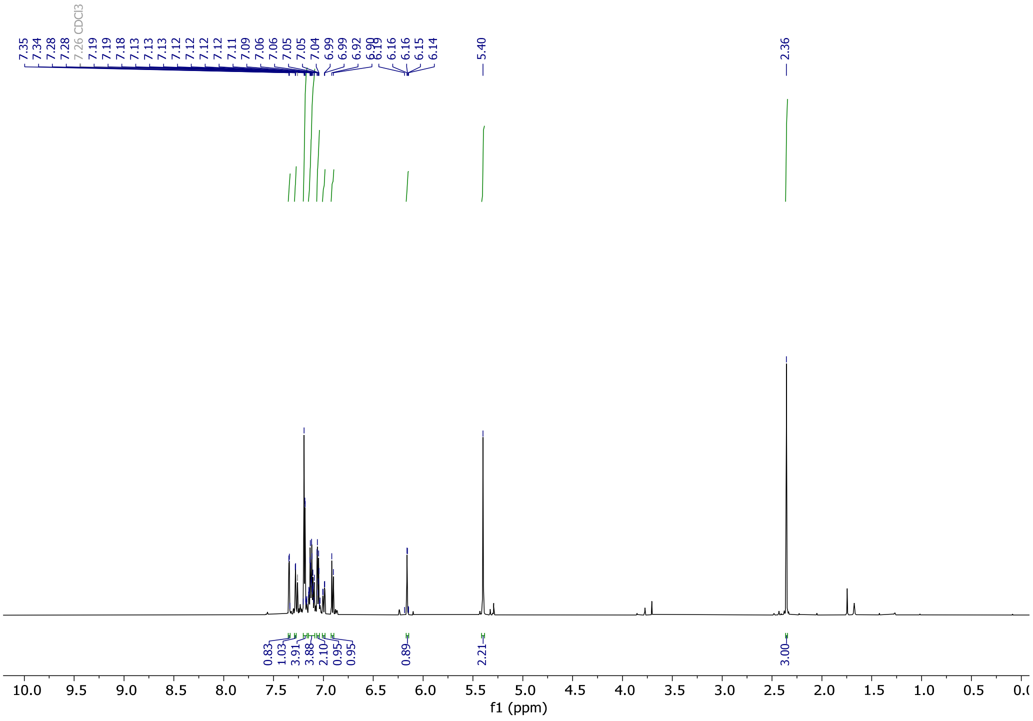

^{13}C NMR (126 MHz, $CDCl_3$)

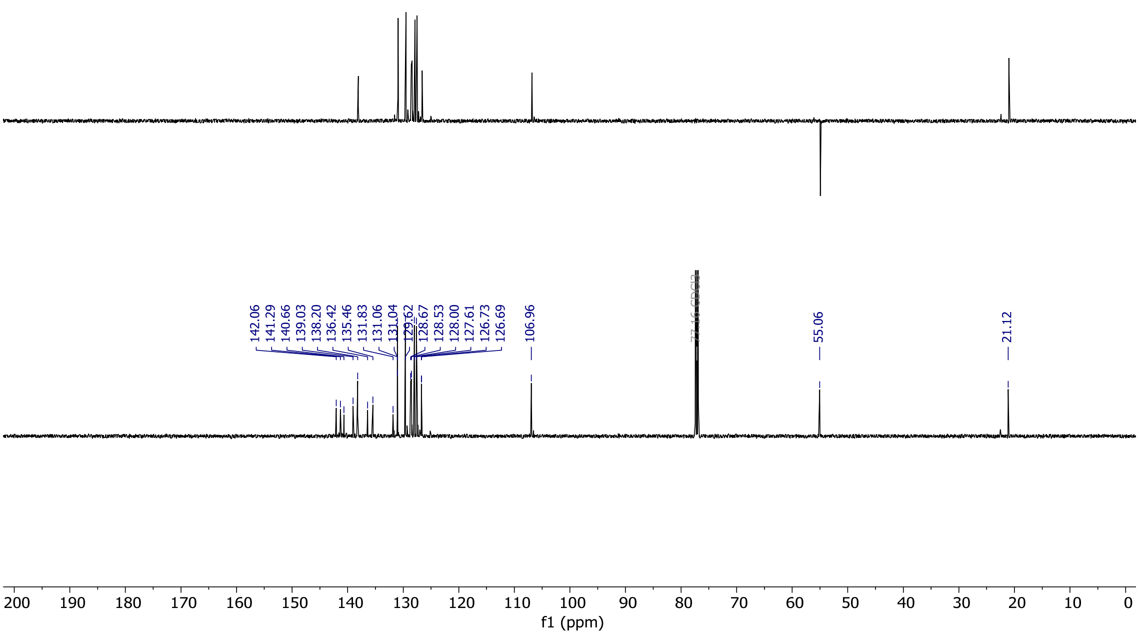

^{1}H NMR (500 MHz, $CDCl_3$)

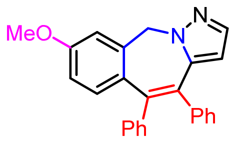

3ca

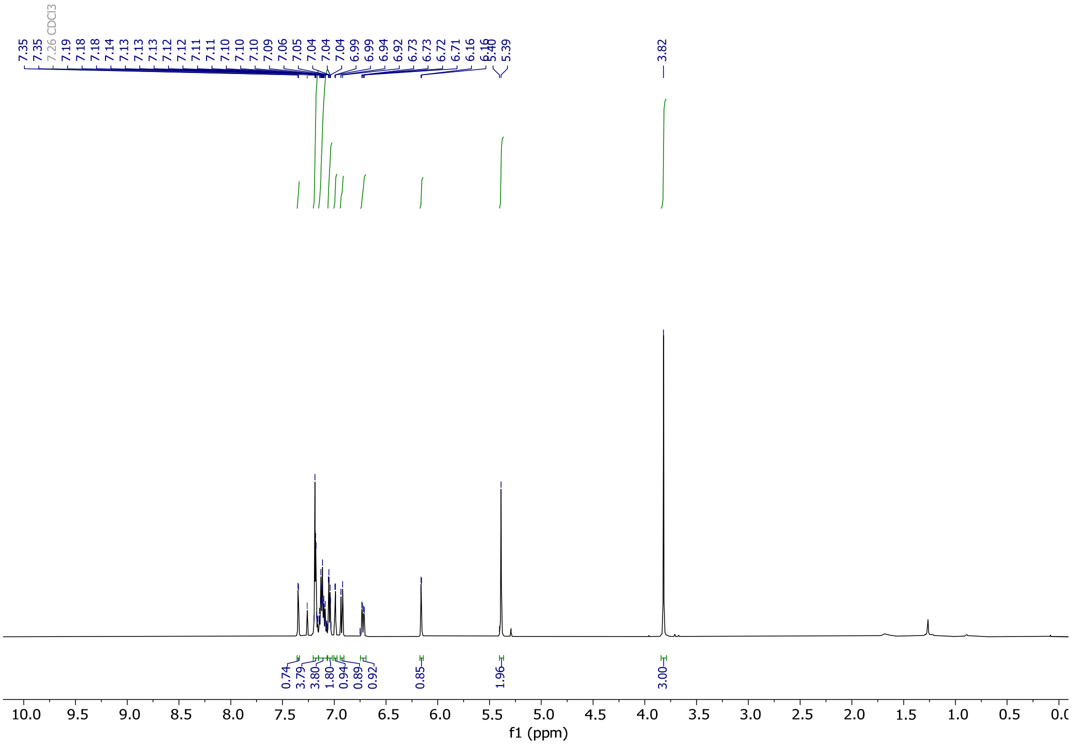

^{13}C NMR (126 MHz, $CDCl_3$)

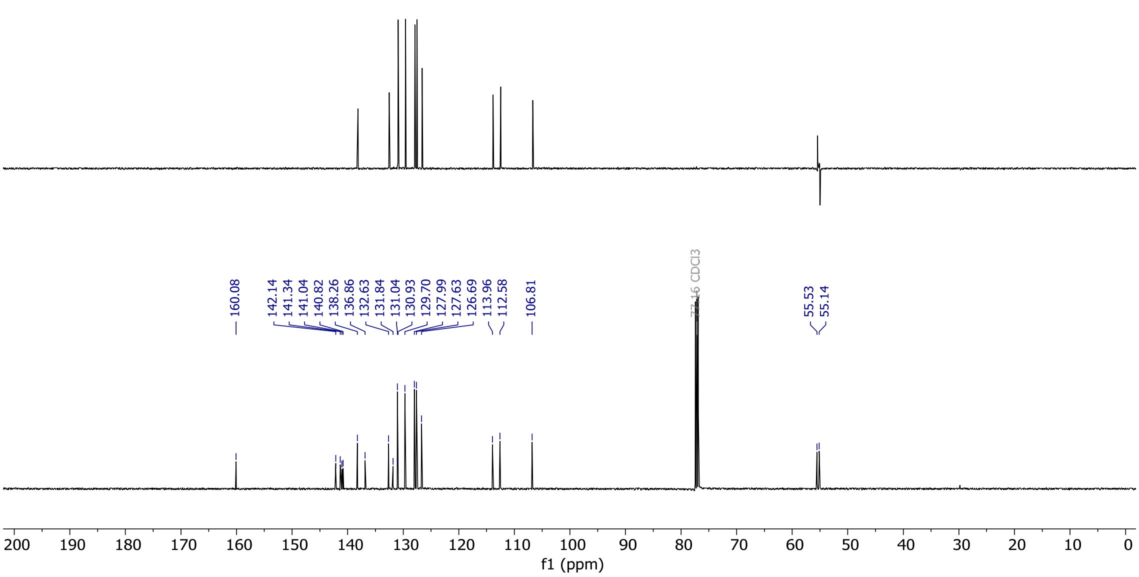

^{1}H NMR (500 MHz, $CDCl_3$)

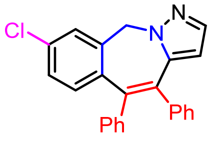

3da

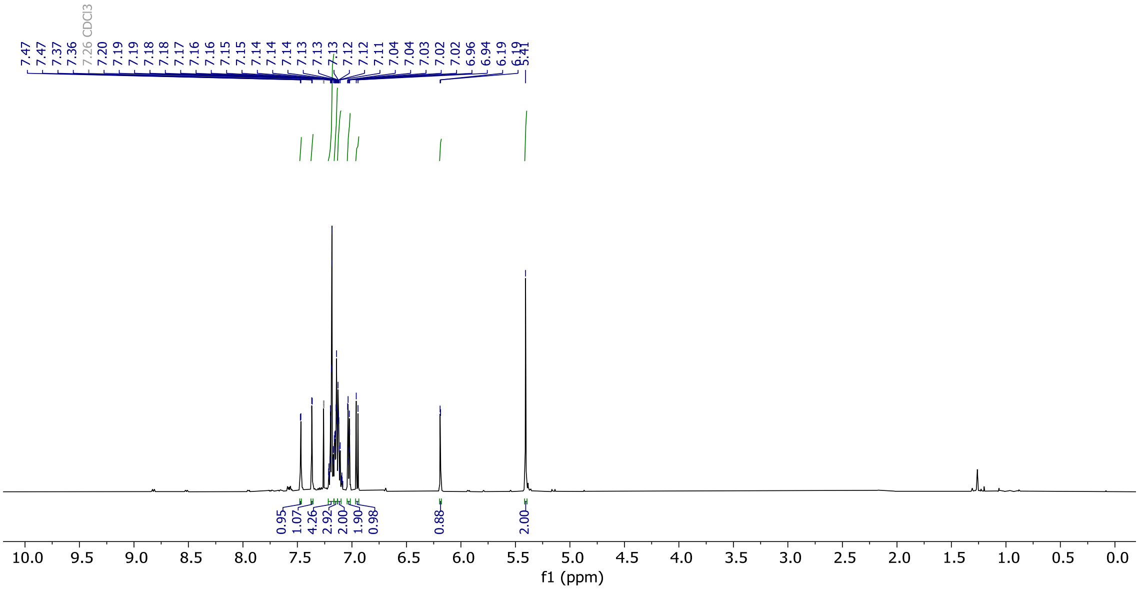

^{13}C NMR (126 MHz, $CDCl_3$)

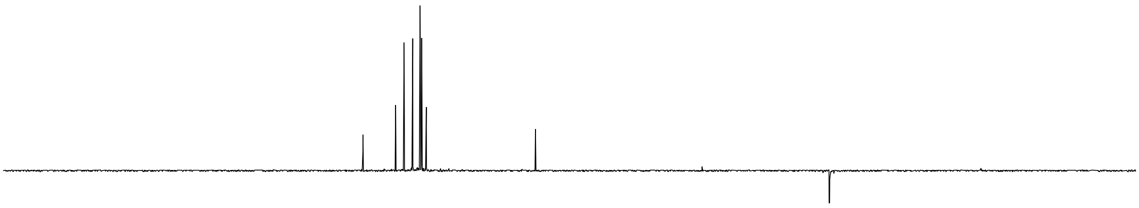

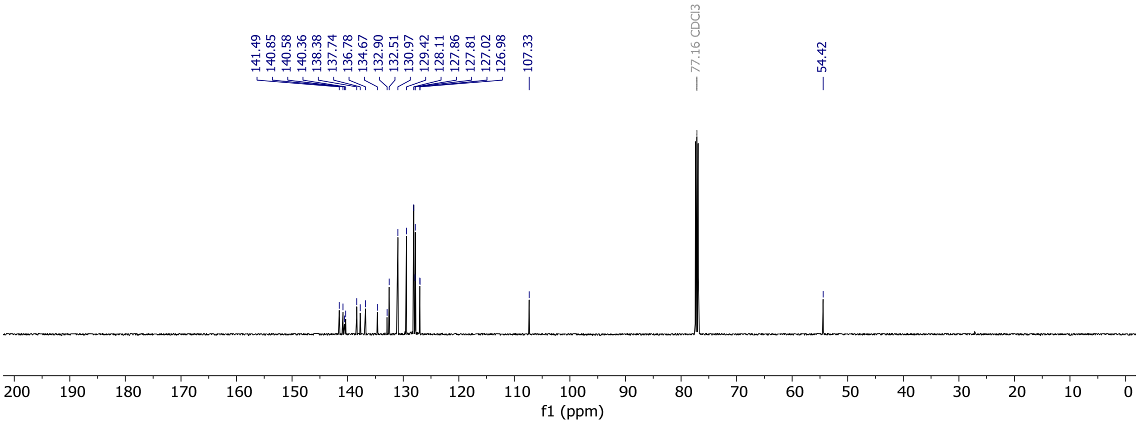

^{1}H NMR (500 MHz, $CDCl_3$)

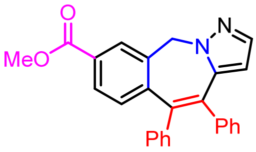

3ea

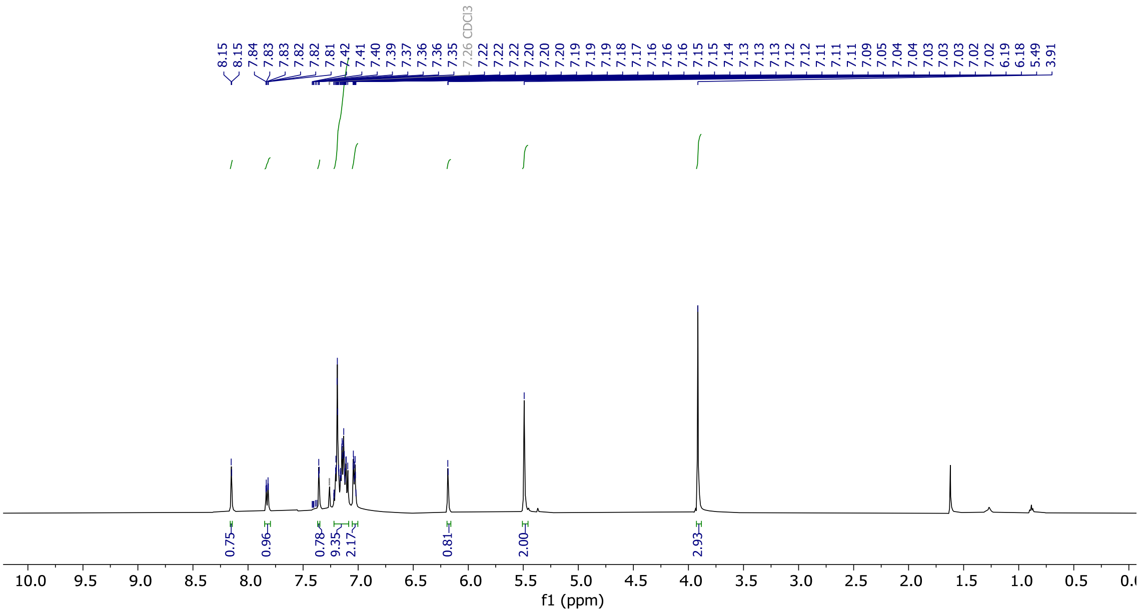

^{13}C NMR (126 MHz, $CDCl_3$)

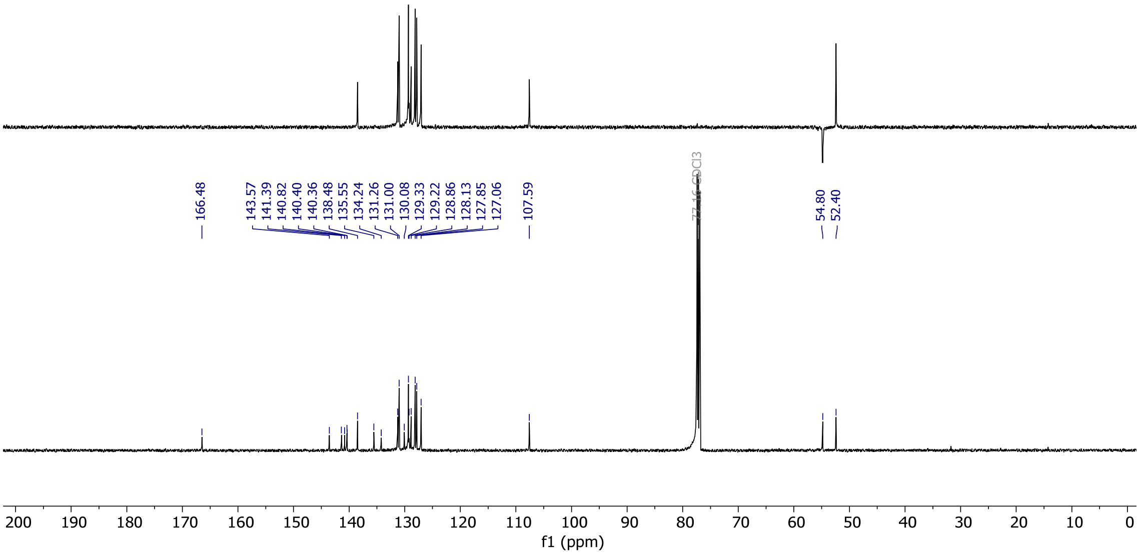

^{1}H NMR (500 MHz, CDCl$_3$)

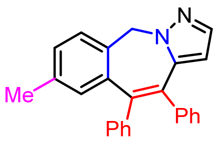

3fa

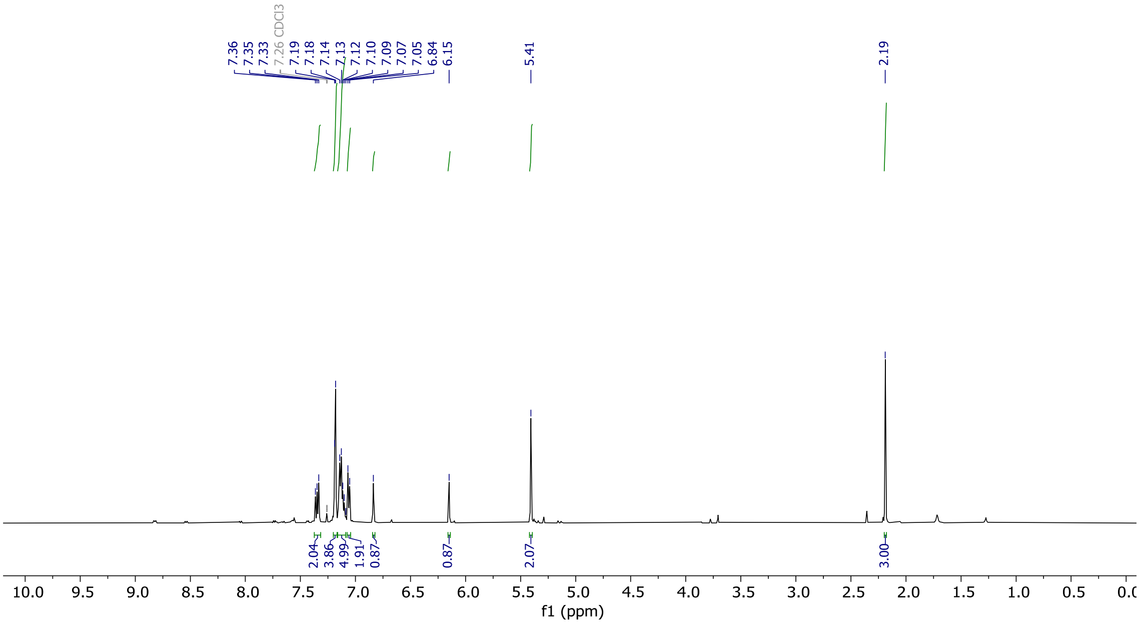

^{13}C NMR (126 MHz, CDCl$_3$)

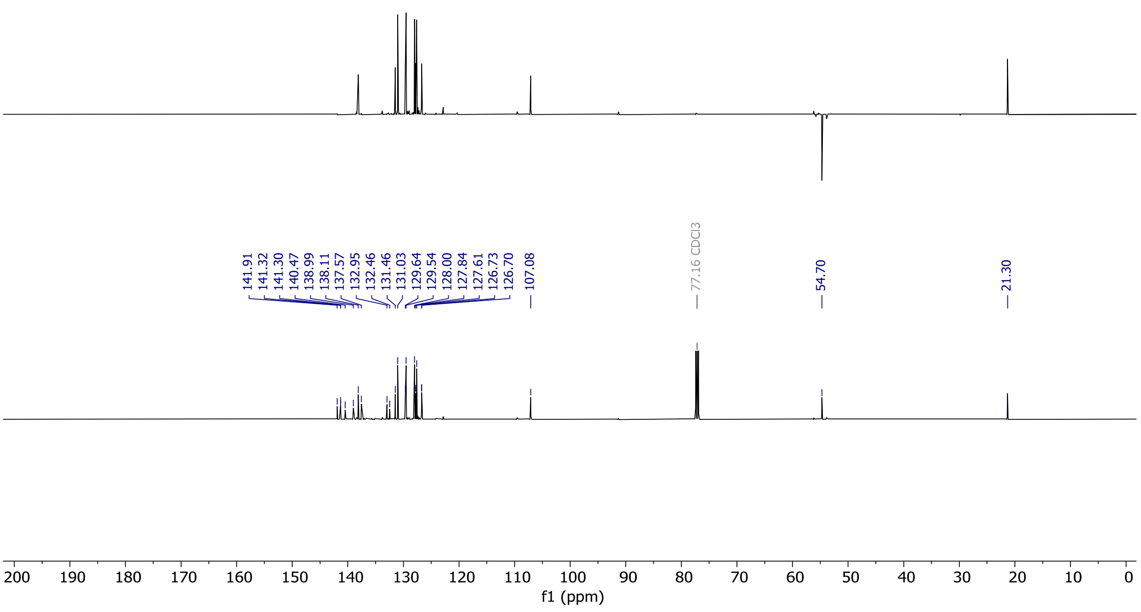

^{1}H NMR (500 MHz, $CDCl_3$)

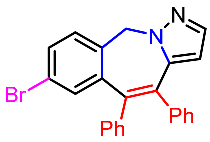
3ga

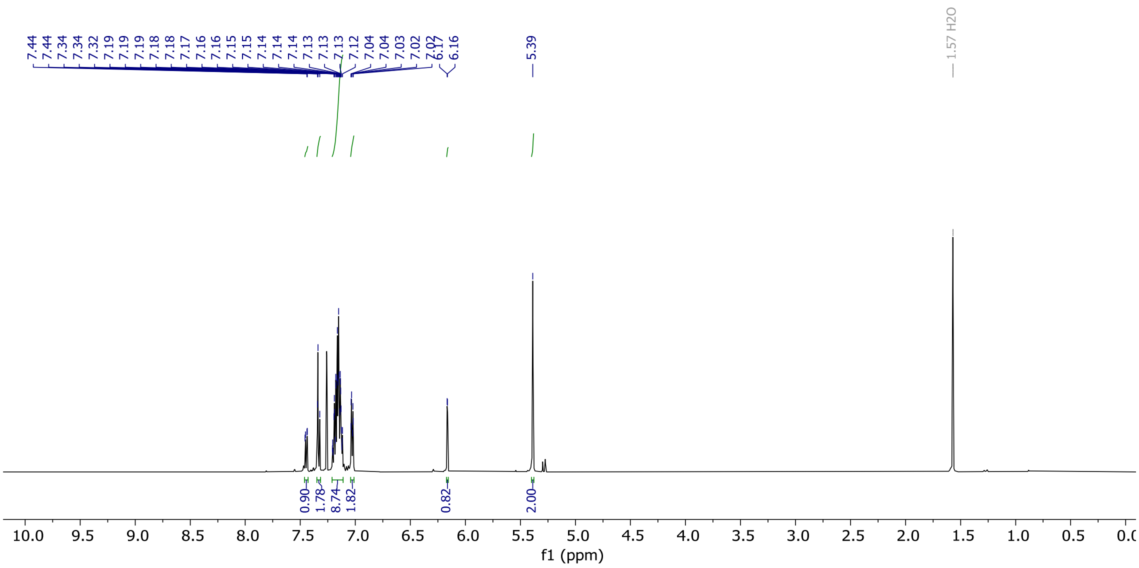

^{13}C NMR (126 MHz, $CDCl_3$)

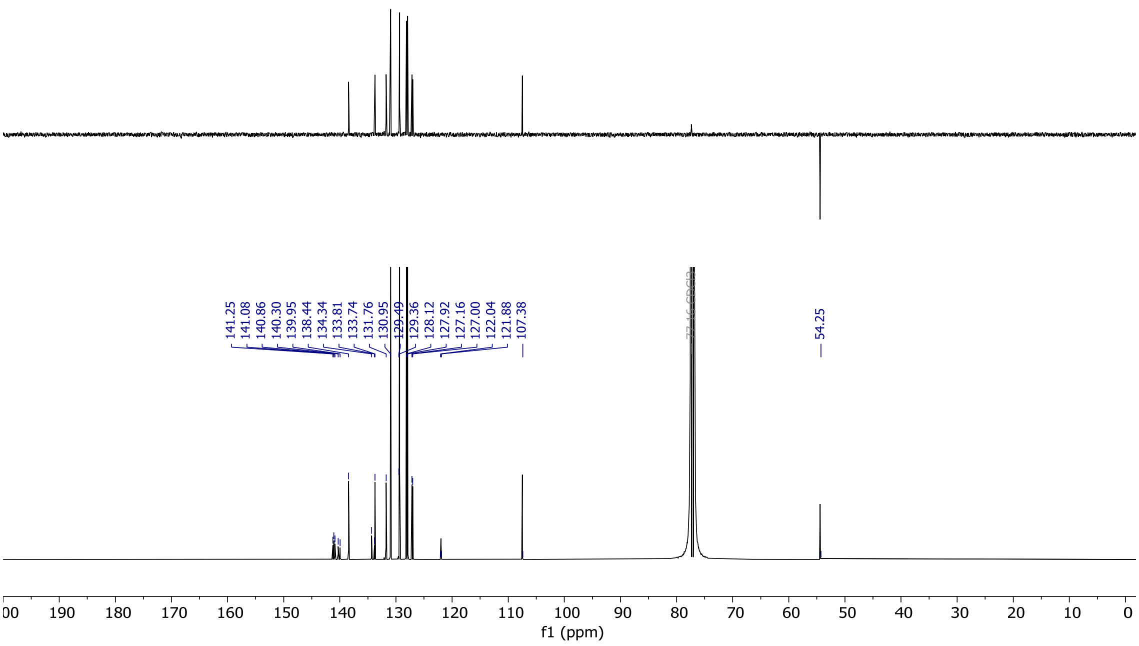

^{1}H NMR (500 MHz, $CDCl_3$)

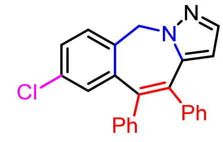

3ha

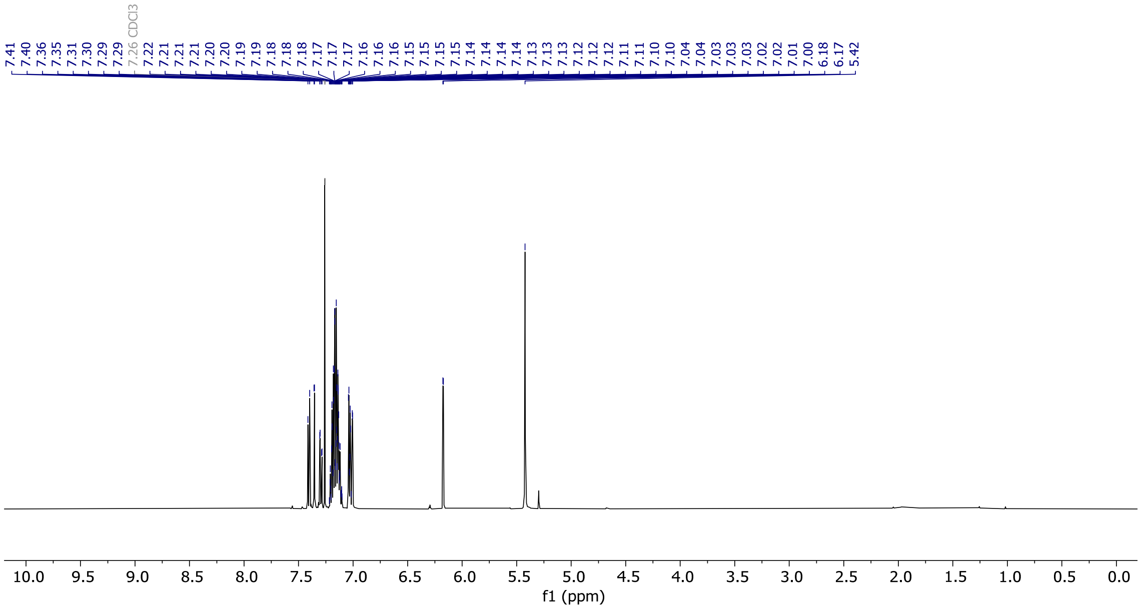

^{13}C NMR (126 MHz, $CDCl_3$)

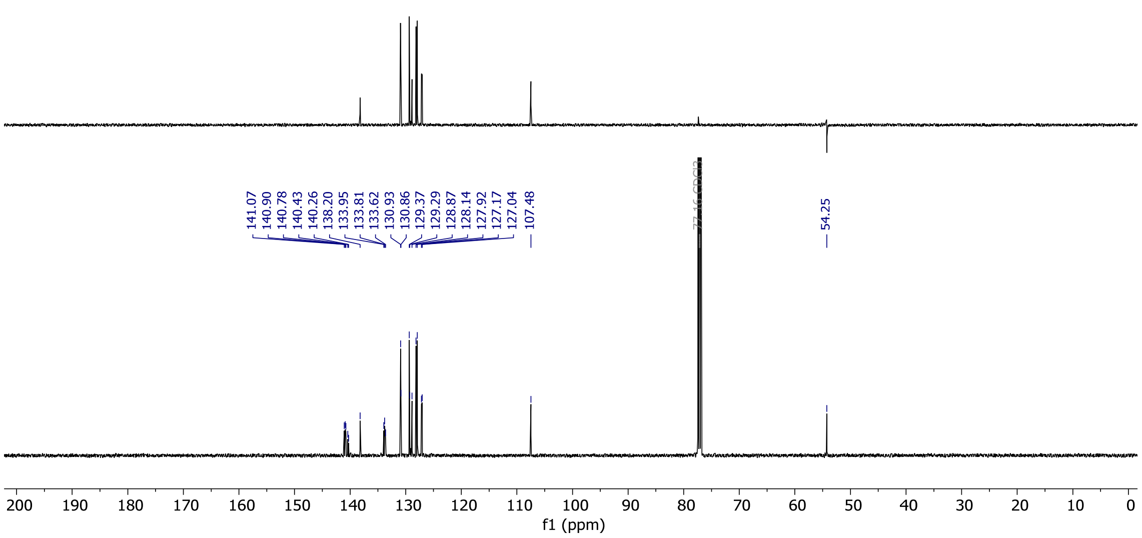

^{1}H NMR (500 MHz, $CDCl_3$)

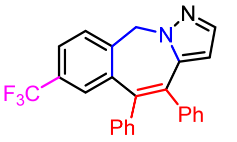

3ia

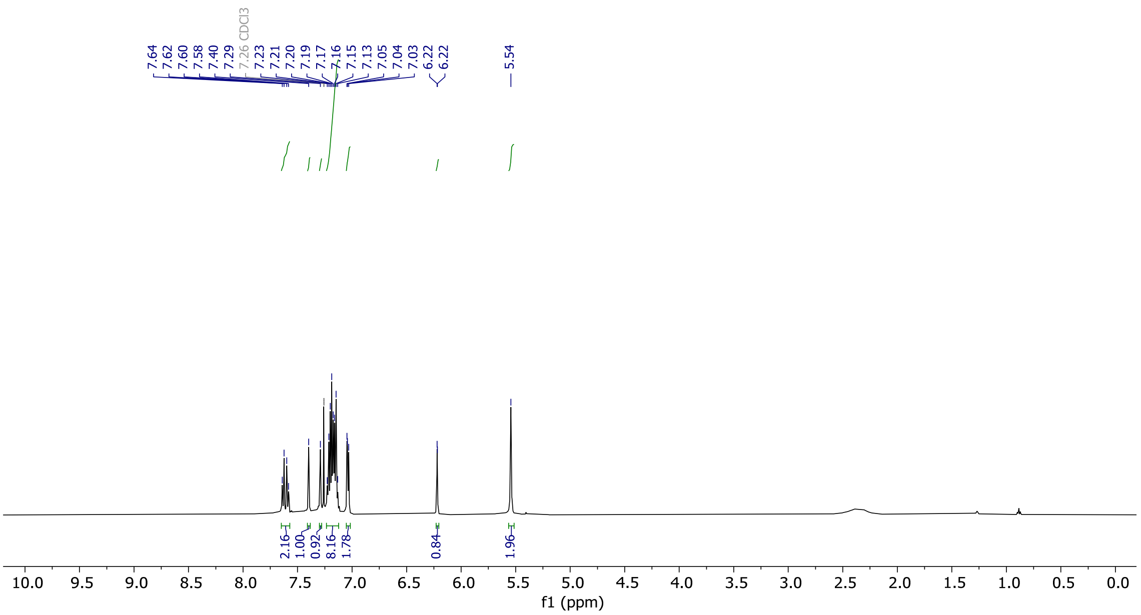

^{13}C NMR (126 MHz, $CDCl_3$)

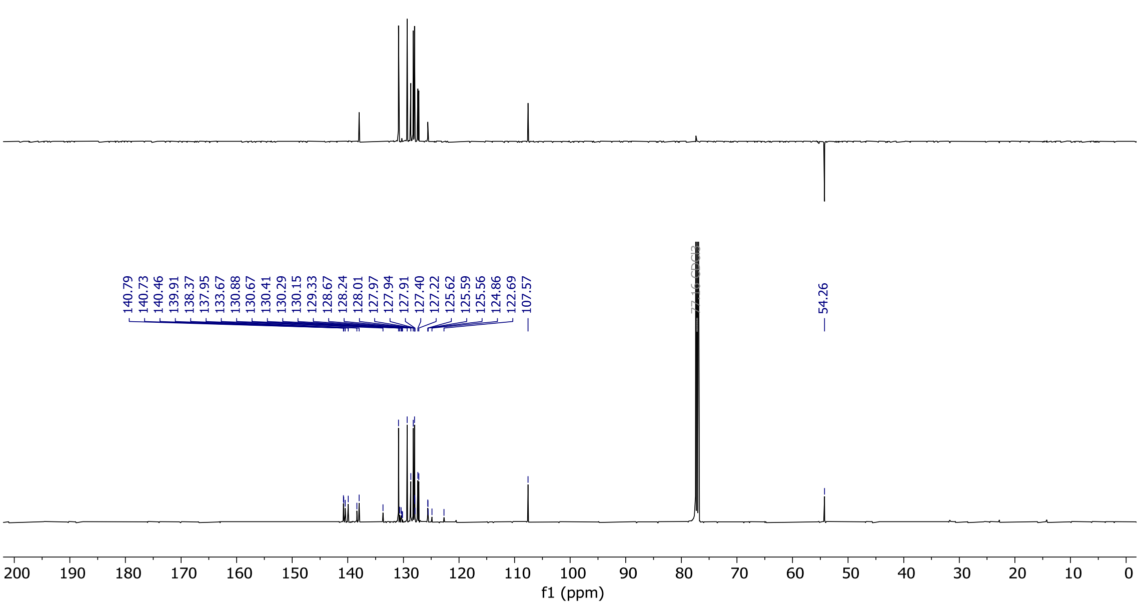

^{19}F NMR (471 MHz, $CDCl_3$)

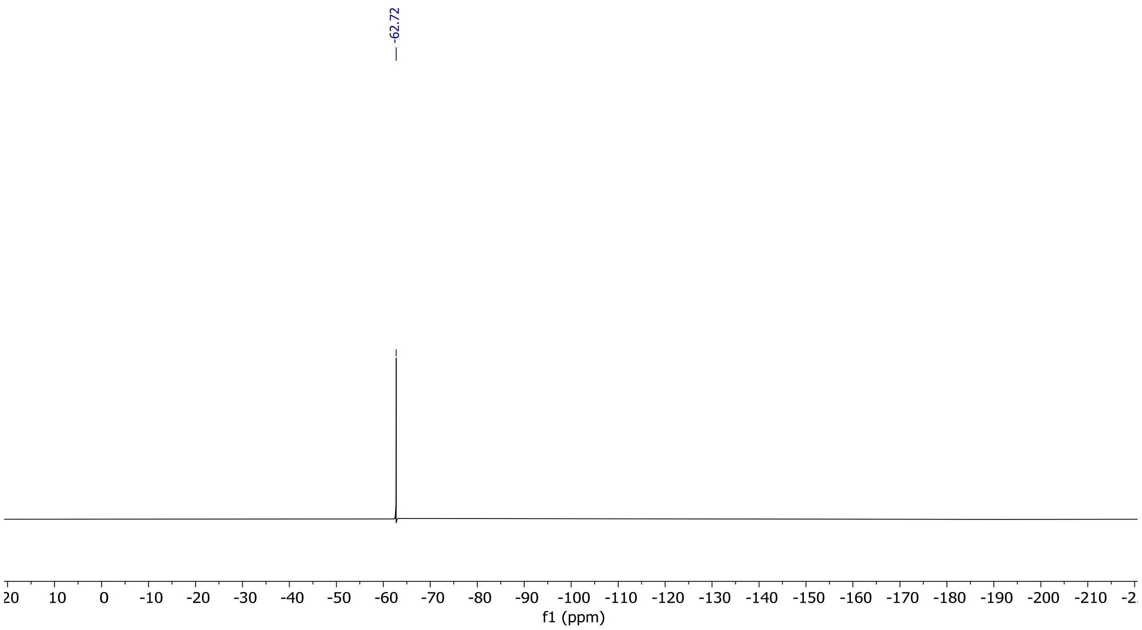

^{1}H NMR (500 MHz, $CDCl_3$)

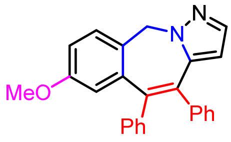

3ja

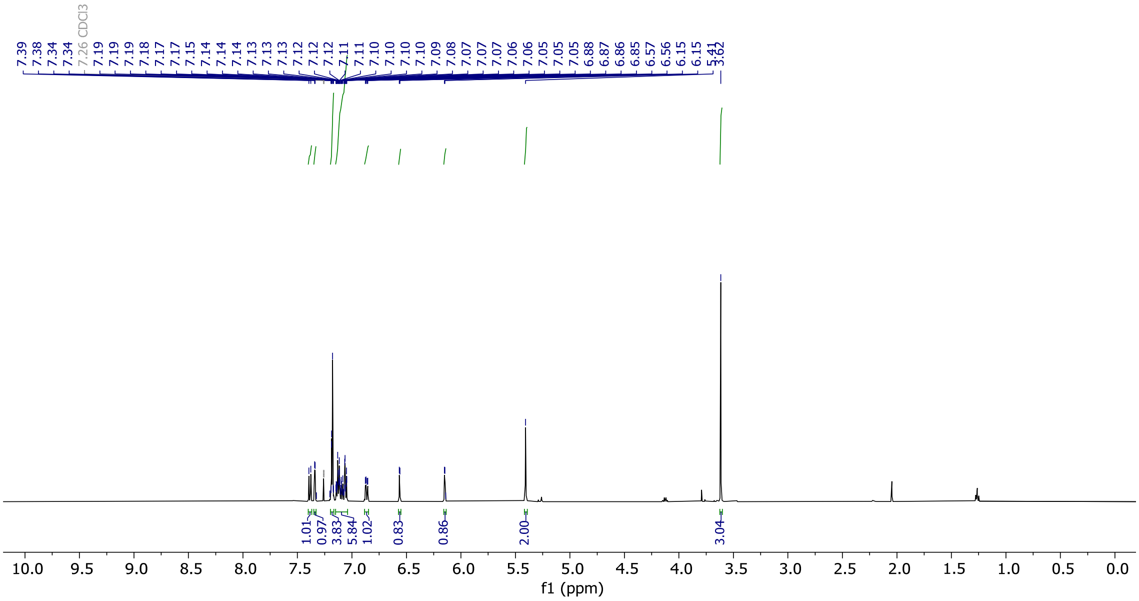

^{13}C NMR (126 MHz, $CDCl_3$)

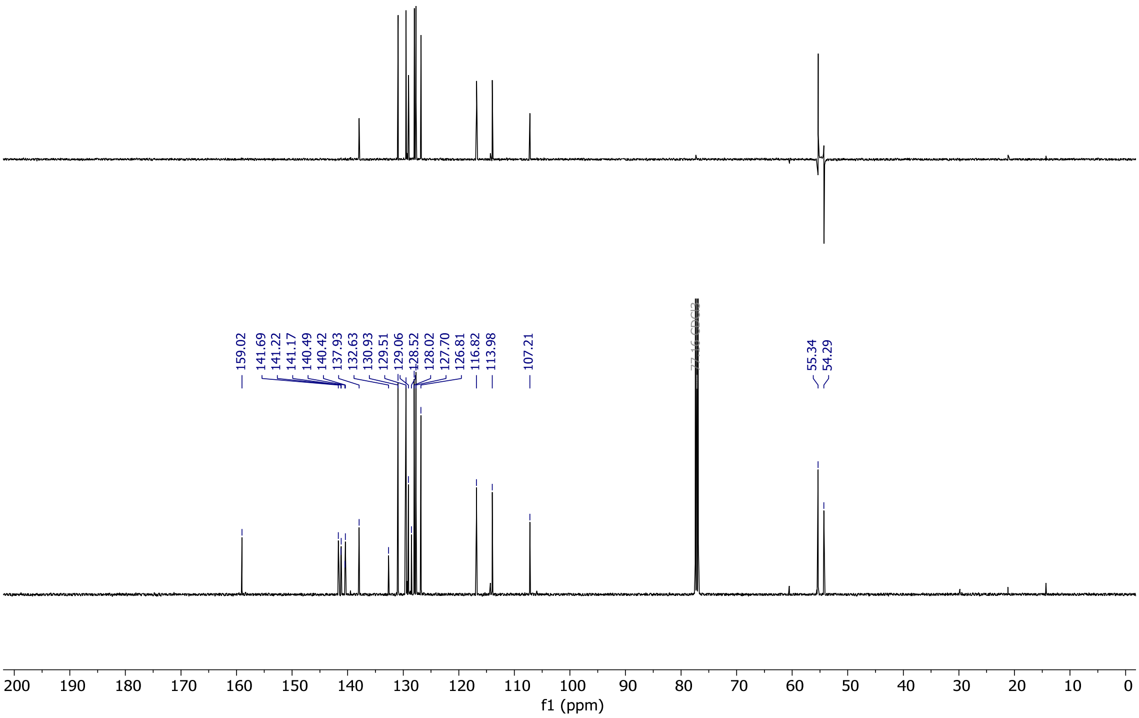

^{1}H NMR (500 MHz, $CDCl_3$)

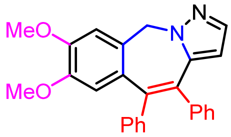

3ka

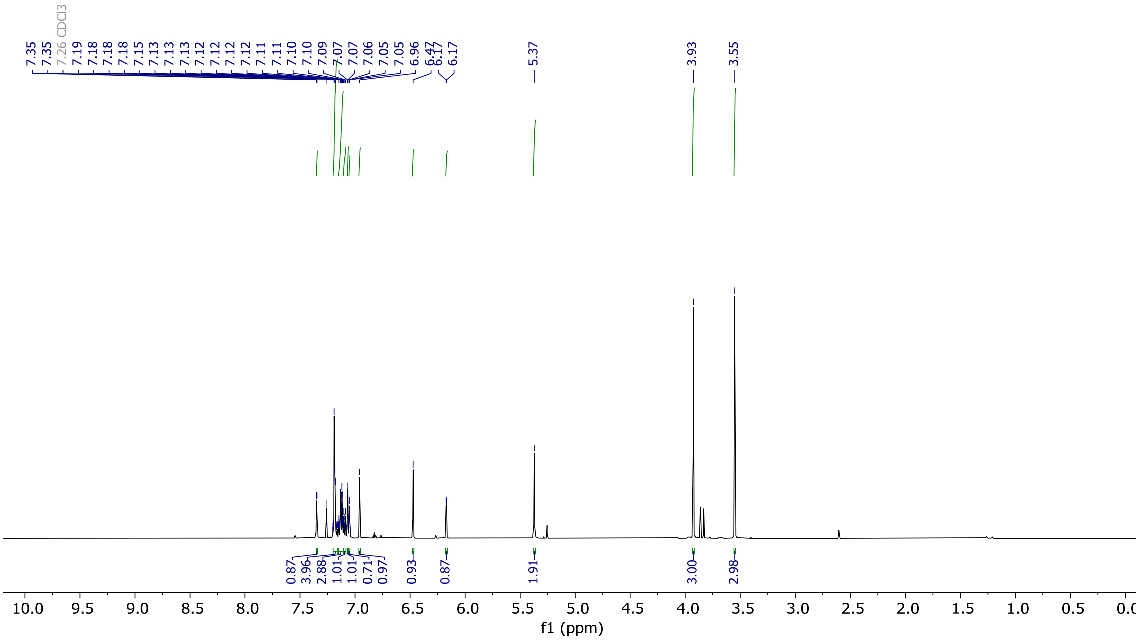

^{13}C NMR (126 MHz, $CDCl_3$)

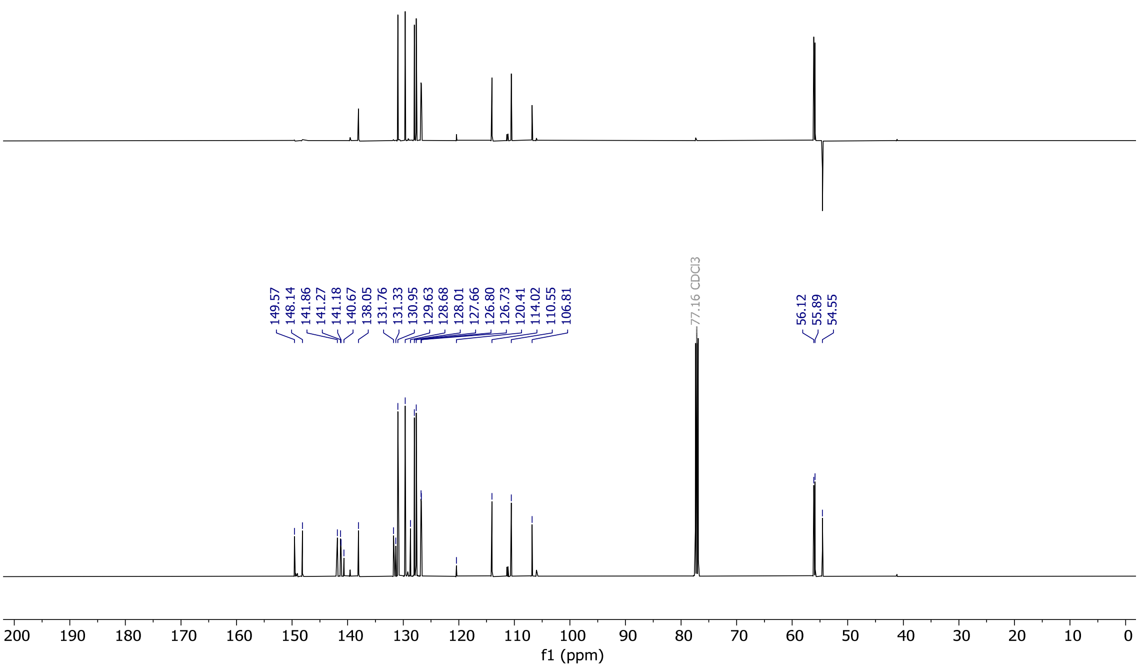

^{1}H NMR (500 MHz, $CDCl_3$)

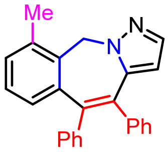

3la

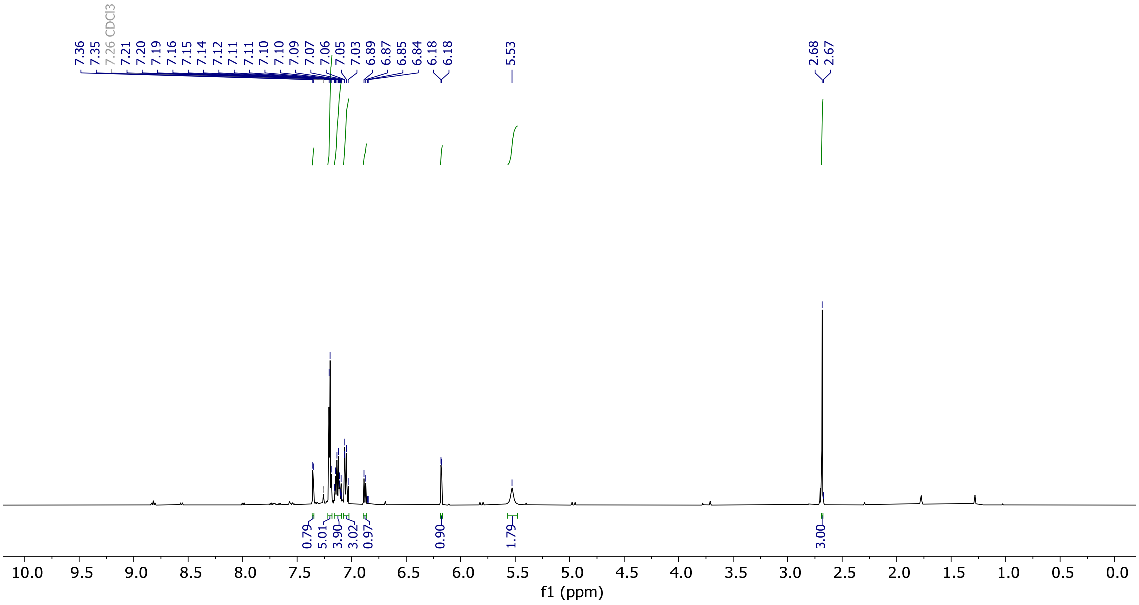

^{13}C NMR (126 MHz, $CDCl_3$)

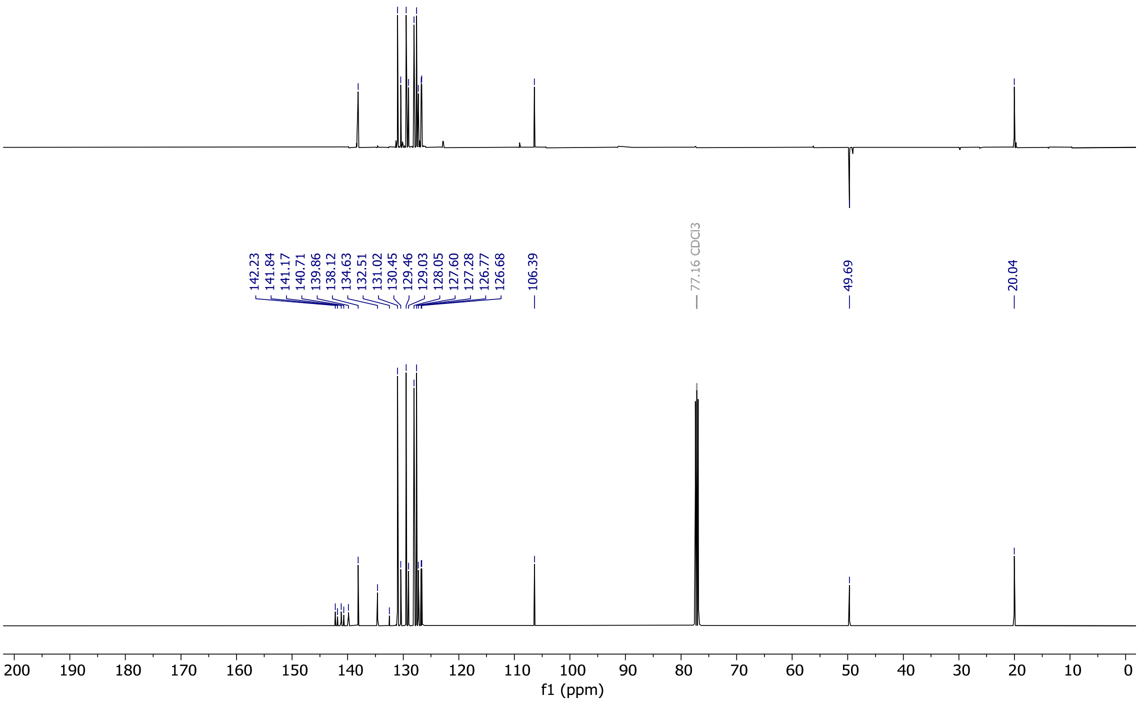

^{1}H NMR (500 MHz, $CDCl_3$)

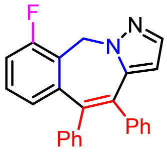

3ma

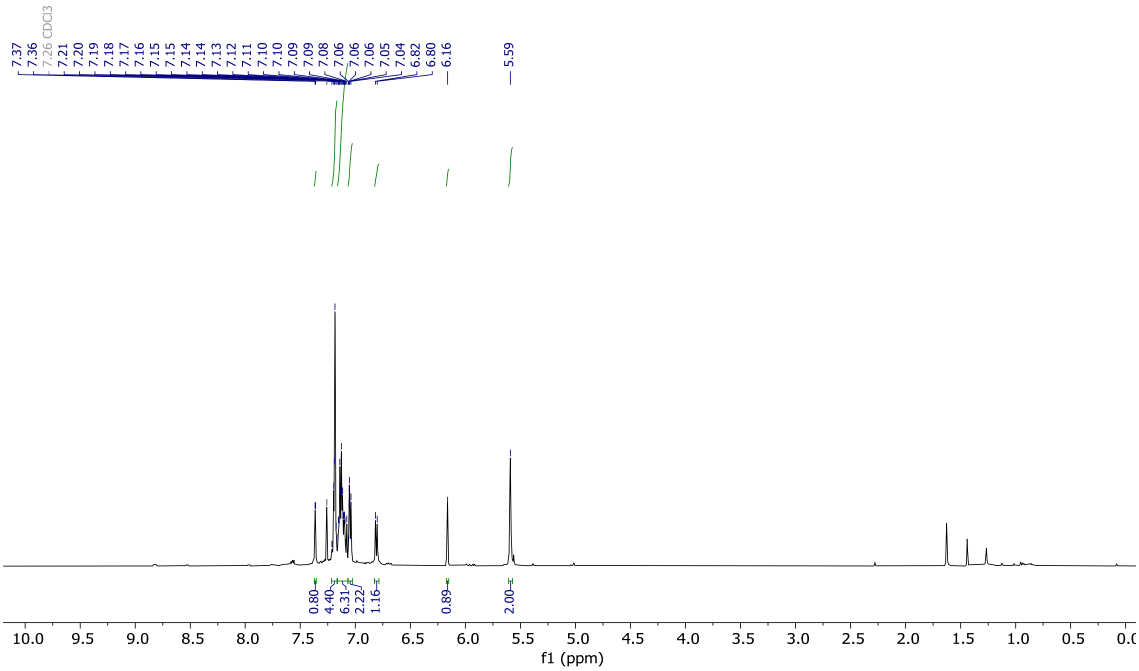

^{13}C NMR (126 MHz, $CDCl_3$)

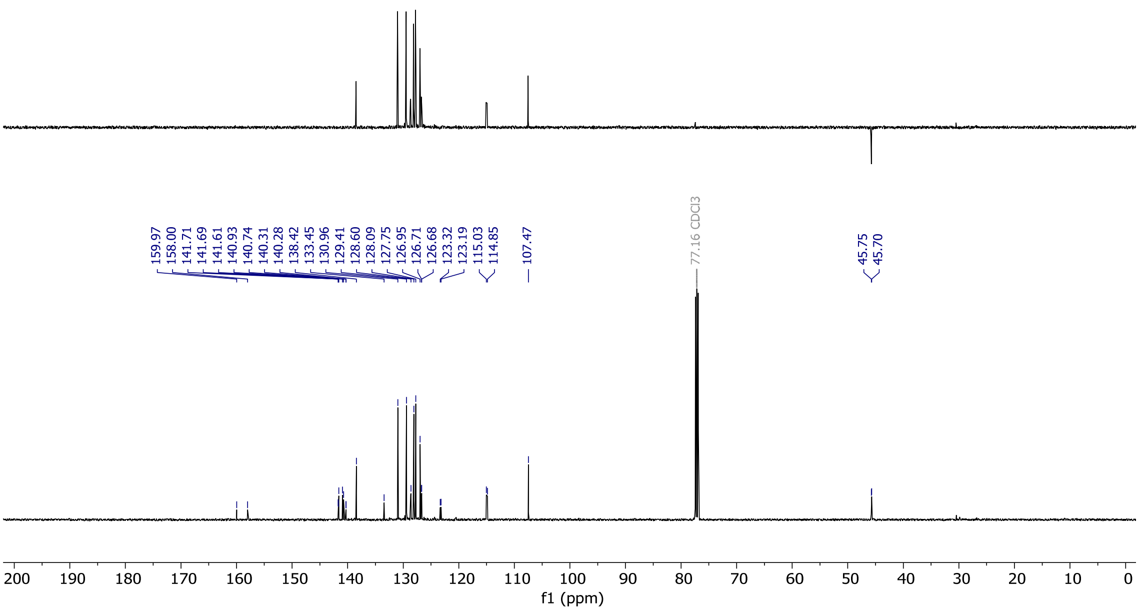

^{19}F NMR (282 MHz, $CDCl_3$)

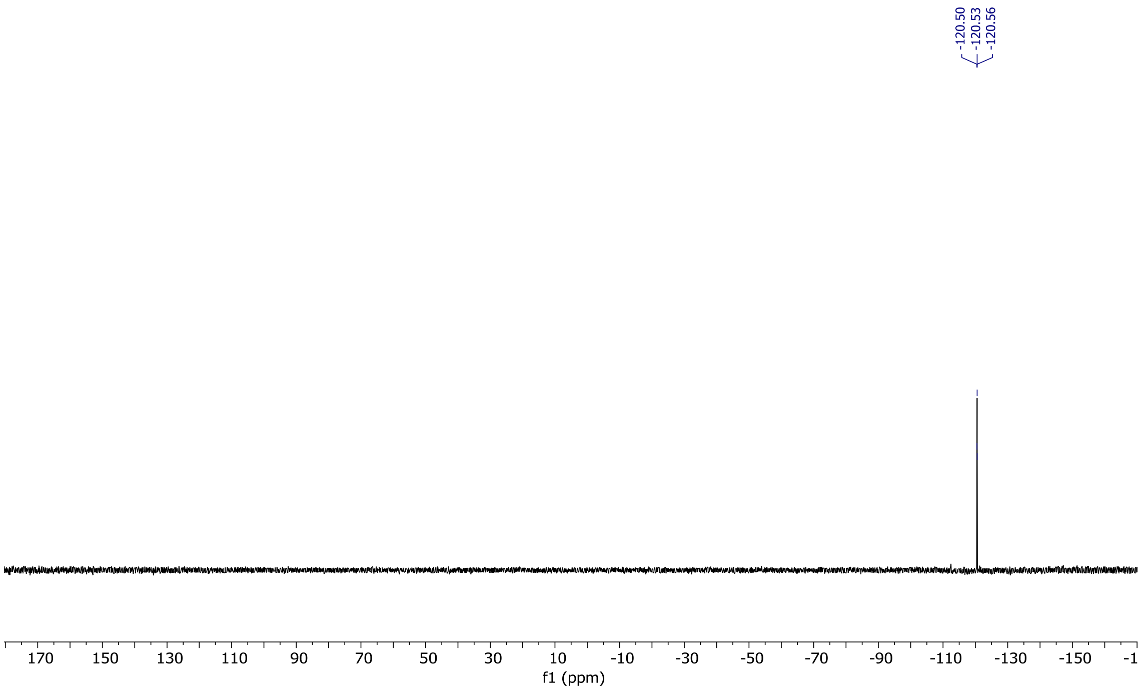

^{1}H NMR (500 MHz, CDCl$_3$)

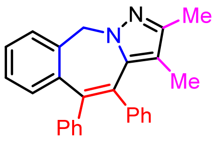

3na

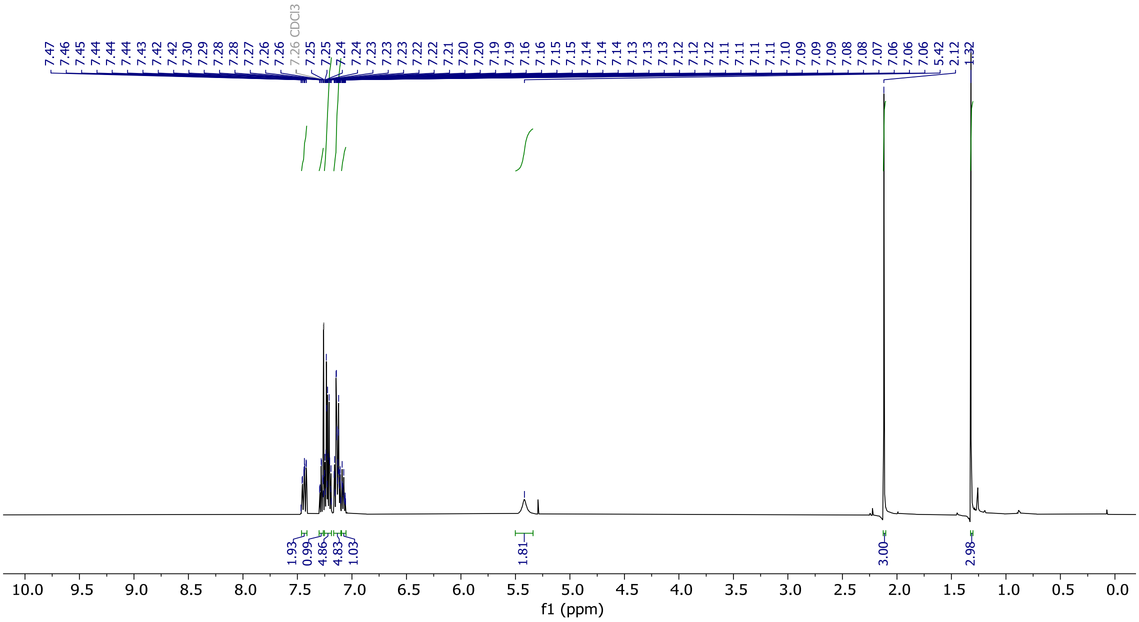

^{13}C NMR (126 MHz, CDCl$_3$)

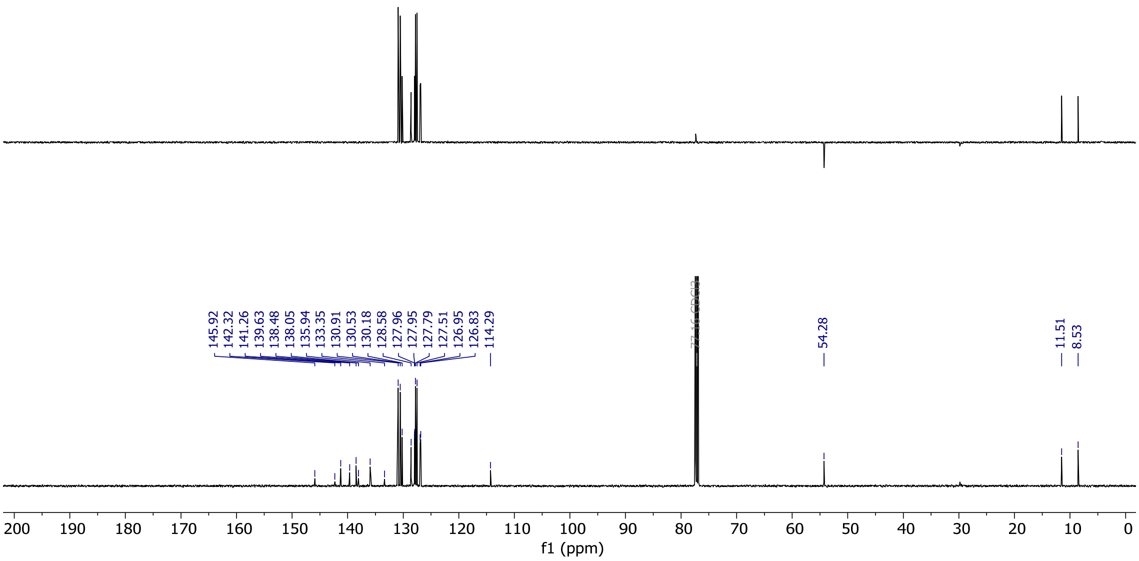

^{1}H NMR (500 MHz, $CDCl_3$)

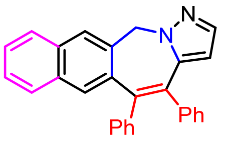

3oa

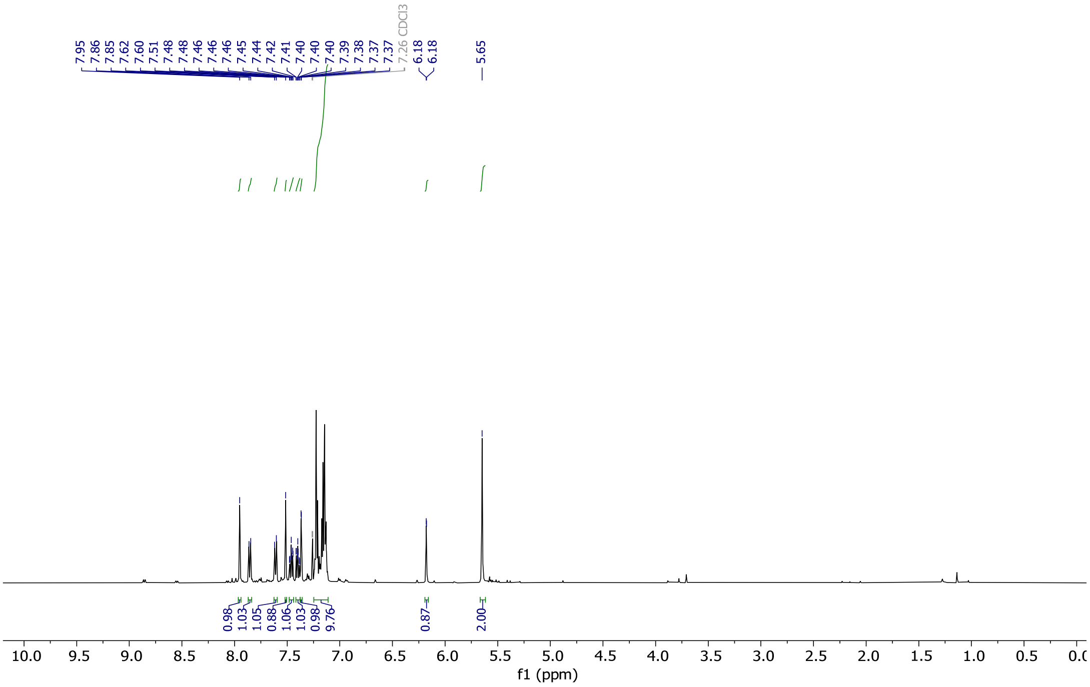

^{13}C NMR (126 MHz, $CDCl_3$)

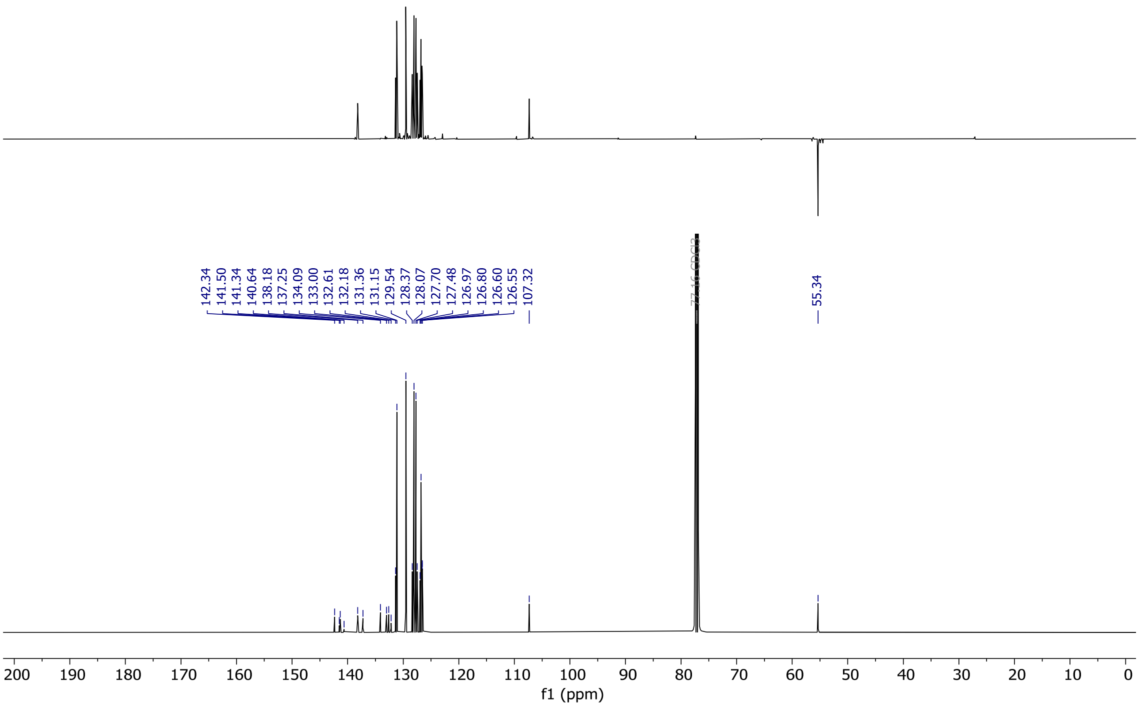

^{1}H NMR (500 MHz, $CDCl_3$)

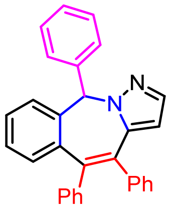

3pa

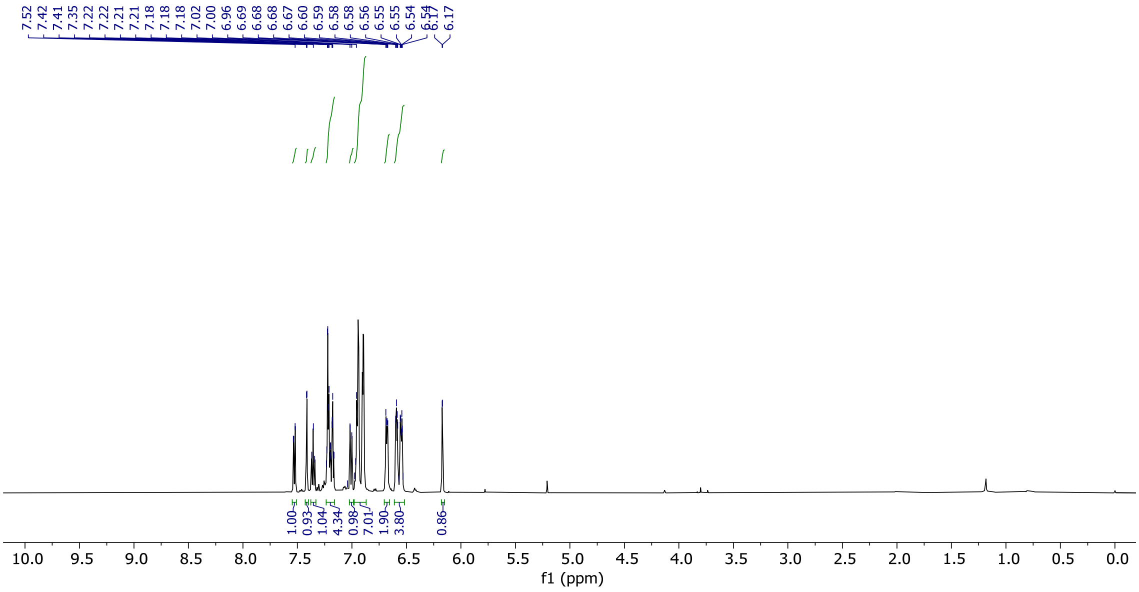

^{13}C NMR (126 MHz, $CDCl_3$)

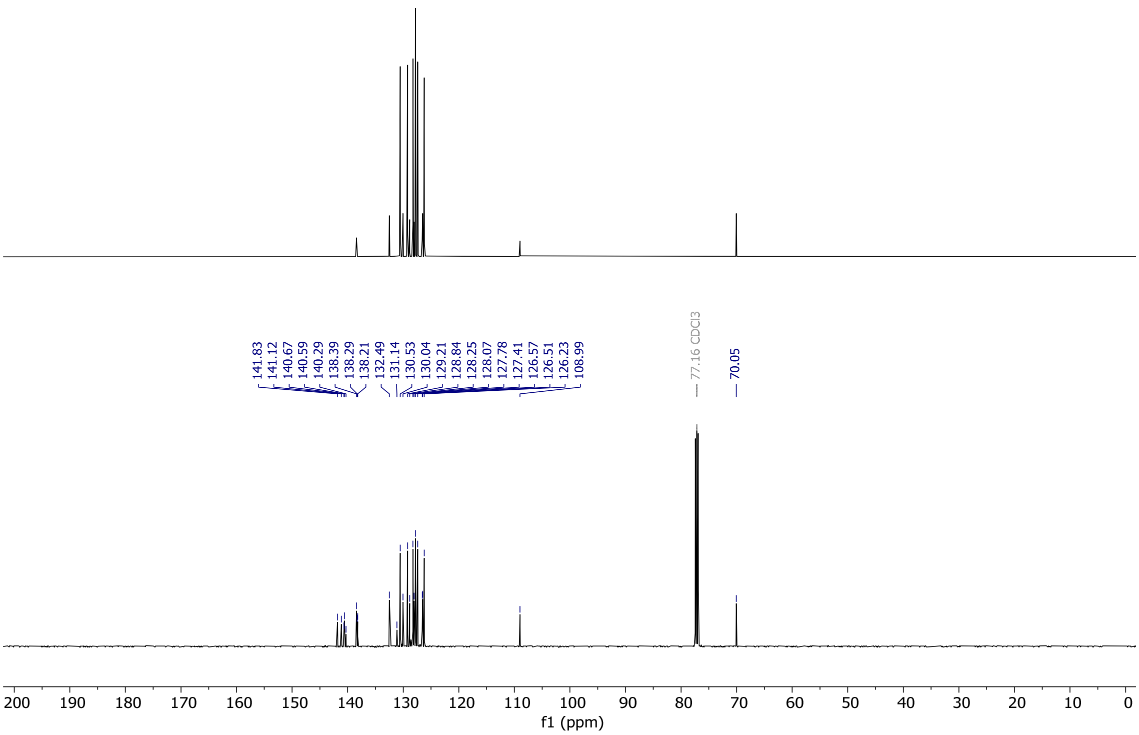

^{1}H NMR (500 MHz, CDCl$_3$)

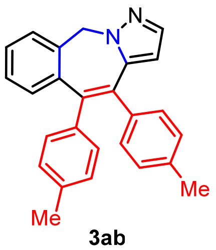

3ab

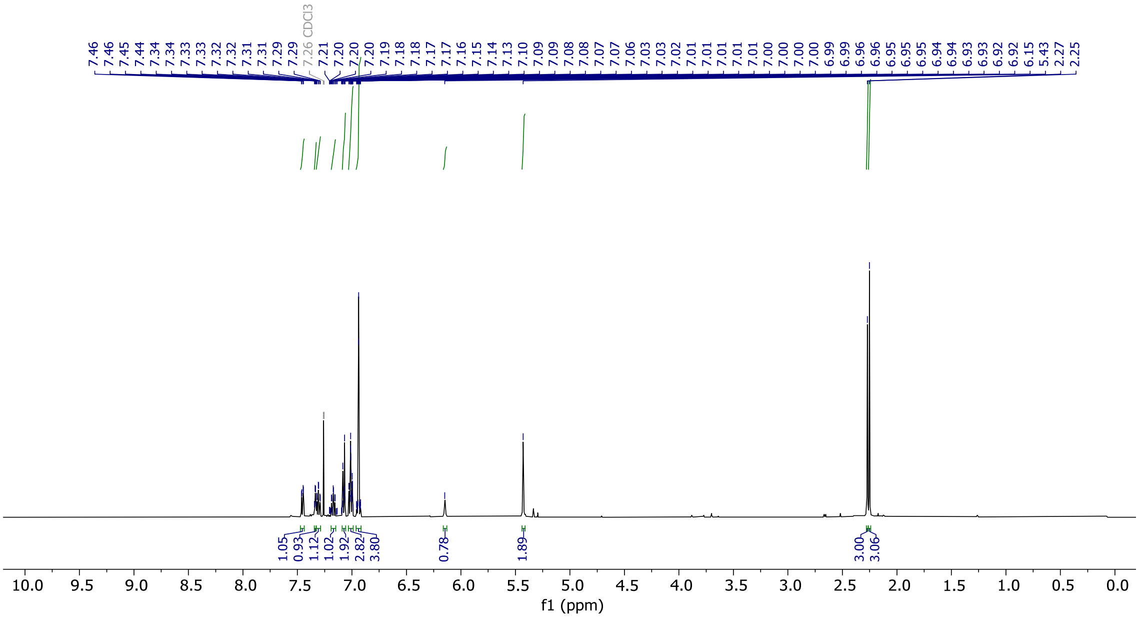

^{13}C NMR (126 MHz, CDCl$_3$)

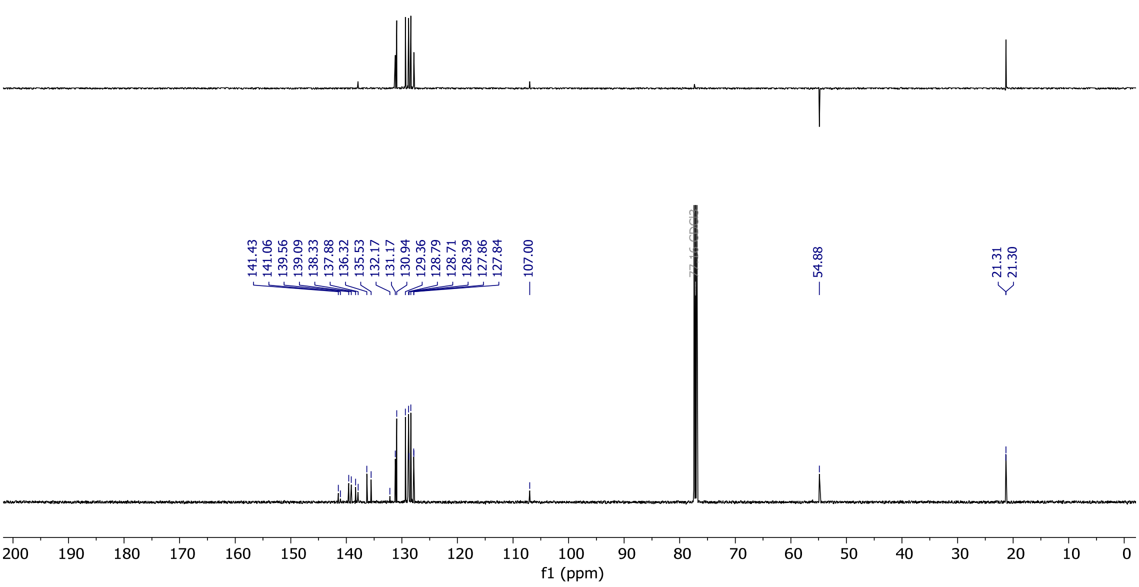

^{1}H NMR (500 MHz, $CDCl_3$)

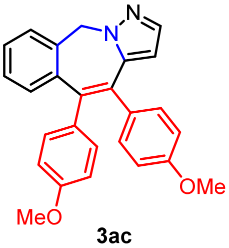

3ac

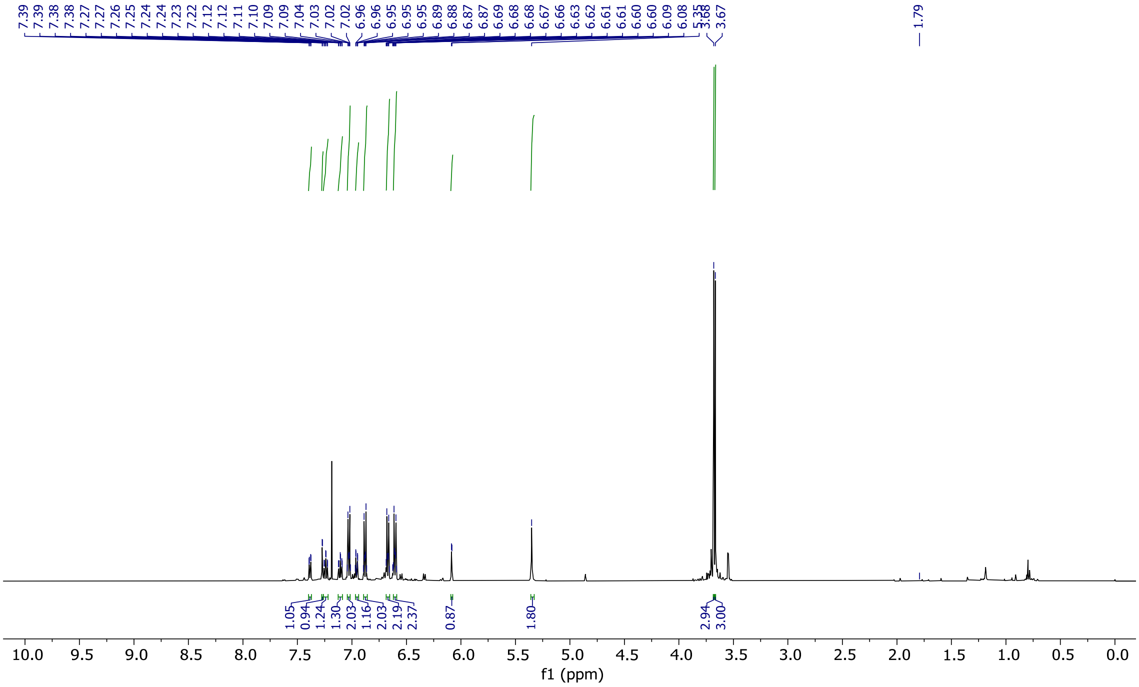

^{13}C NMR (126 MHz, $CDCl_3$)

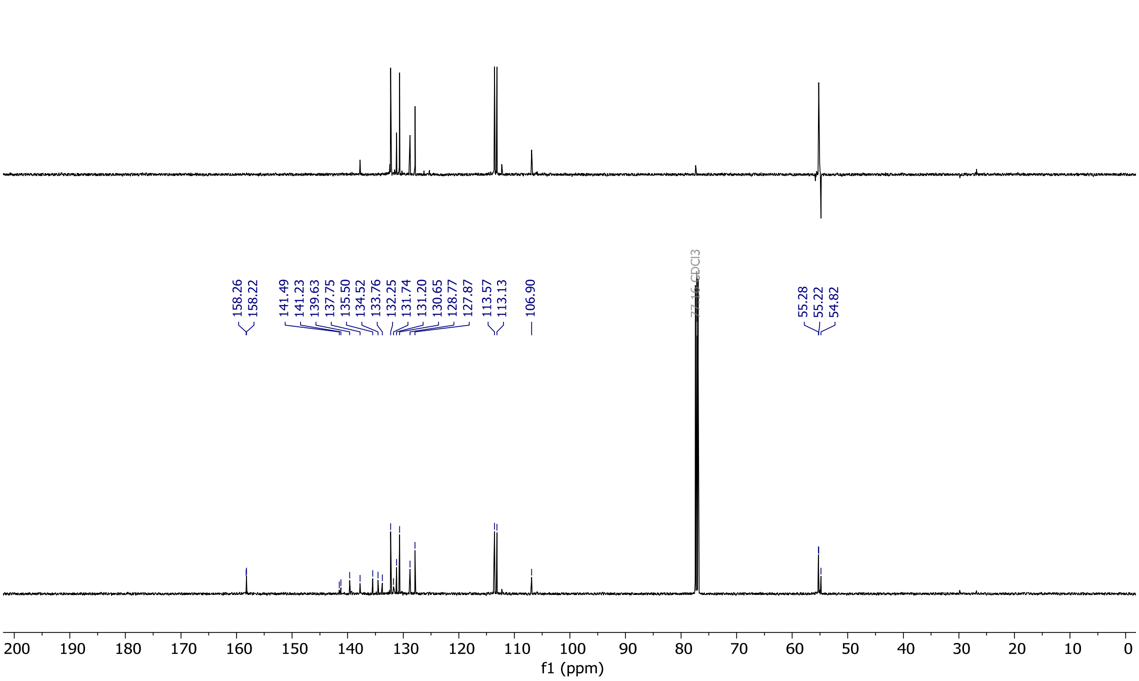

^{1}H NMR (500 MHz, CDCl$_3$)

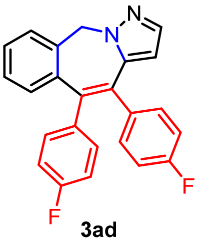

3ad

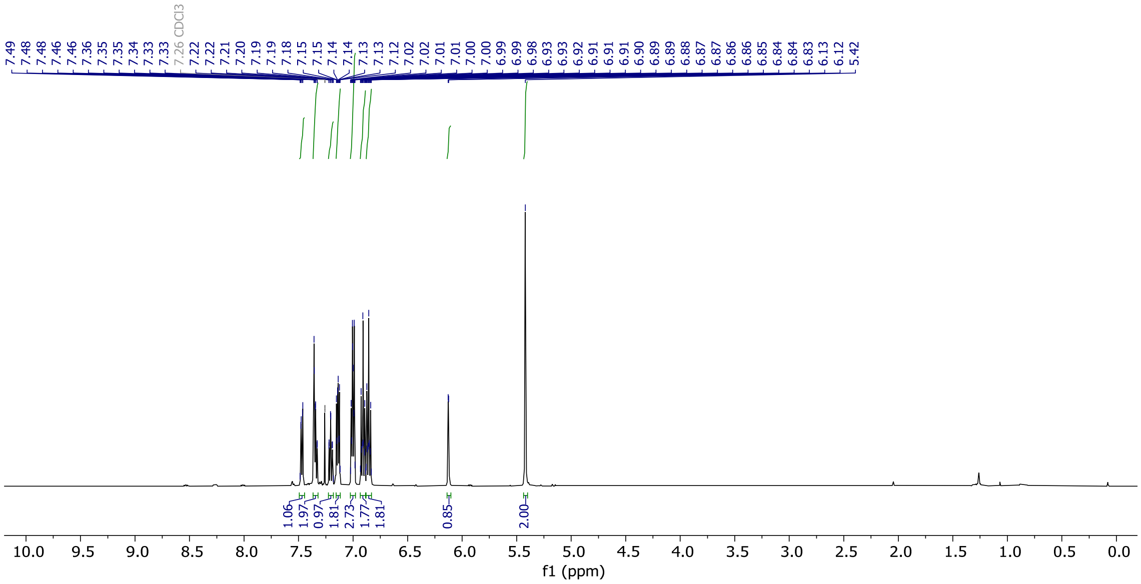

^{13}C NMR (126 MHz, CDCl$_3$)

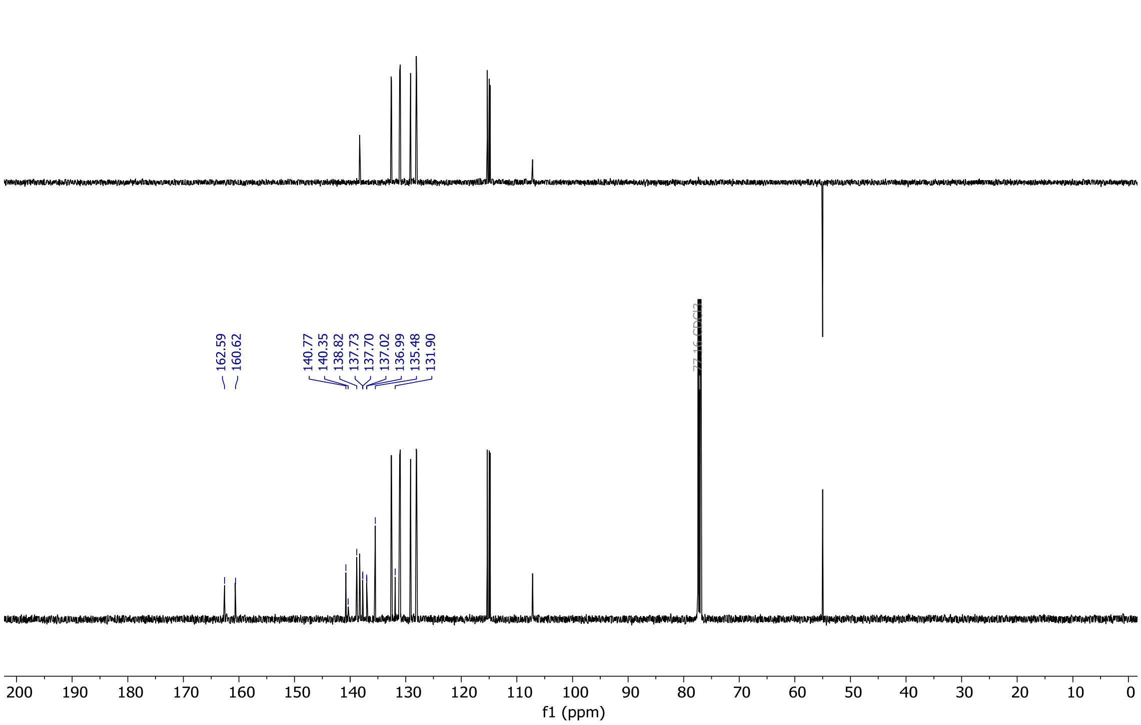

^{19}F NMR (471 MHz, CDCl$_3$)

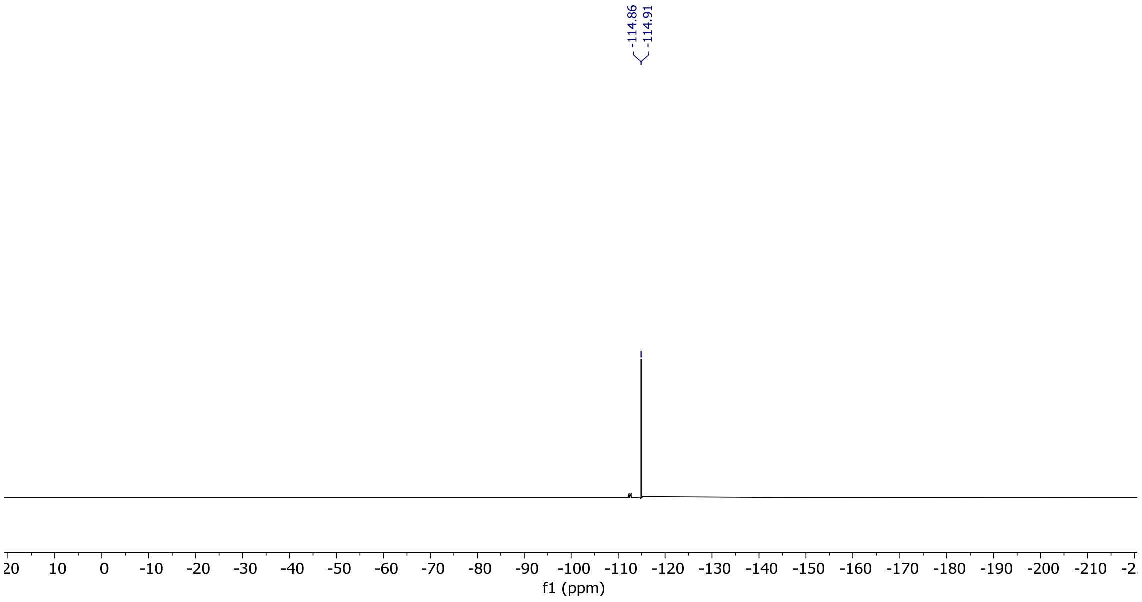

^{1}H NMR (500 MHz, CDCl3)

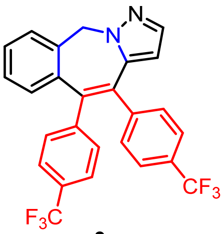

3ae

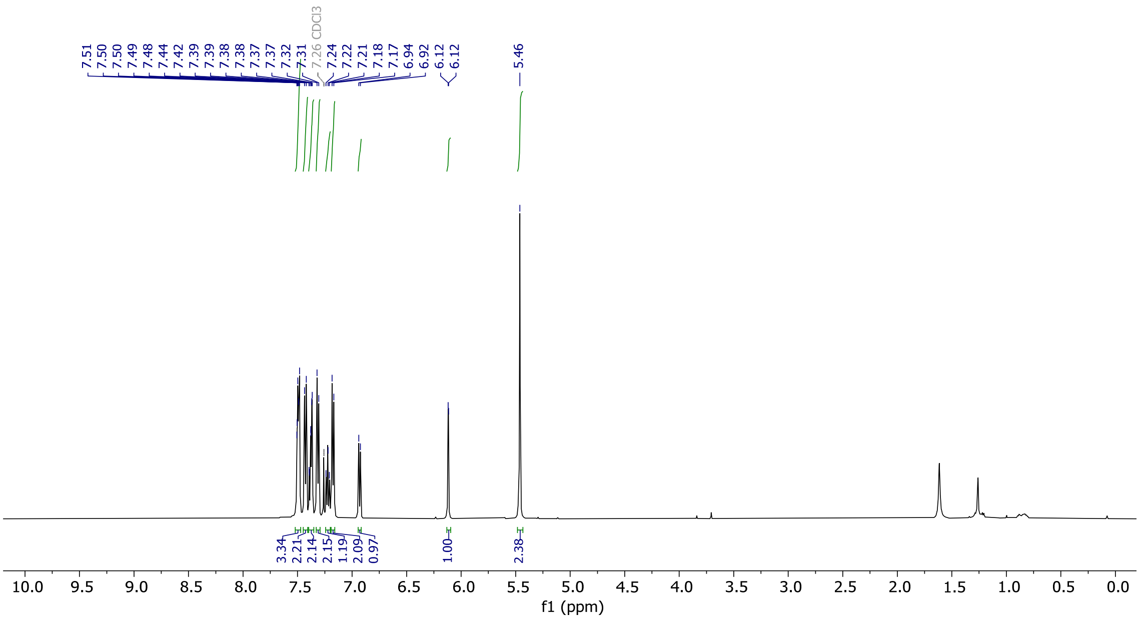

^{13}C NMR (126 MHz, CDCl3)

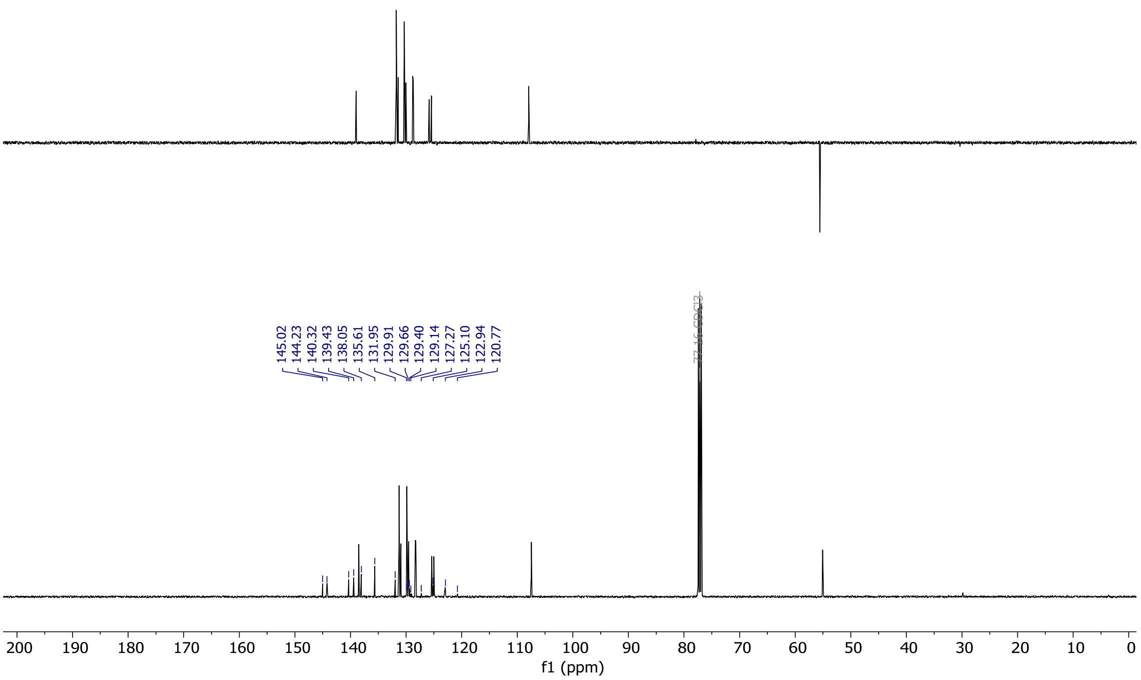

^{19}F NMR (471 MHz, $CDCl_3$)

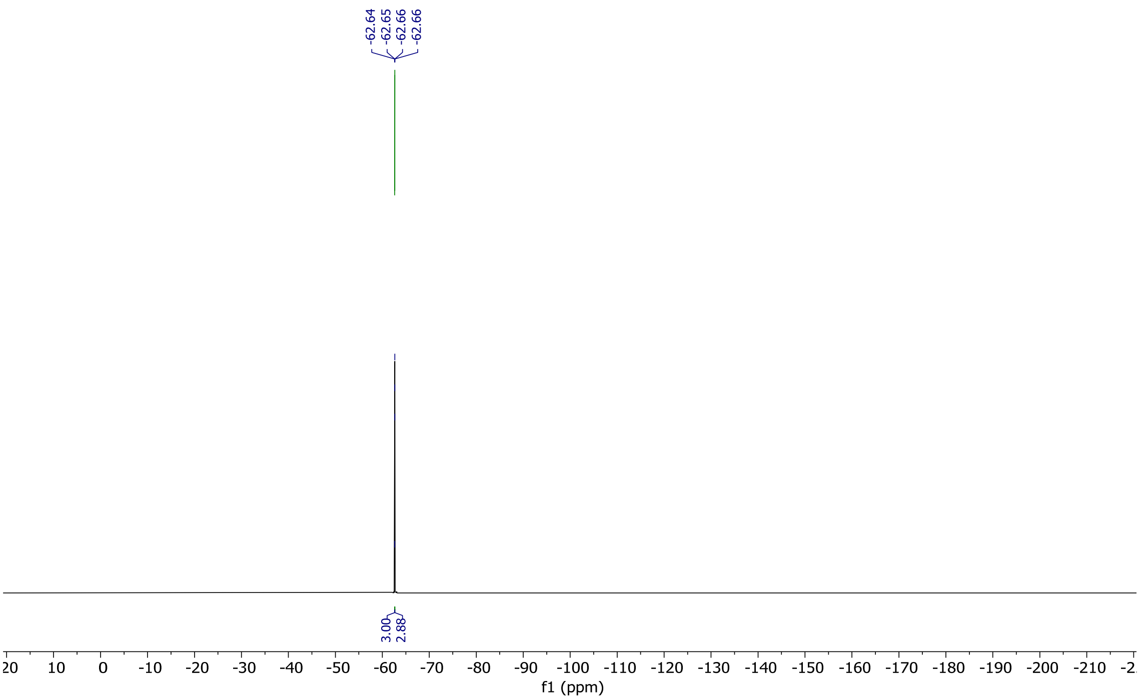

^{1}H NMR (500 MHz, CDCl$_3$)

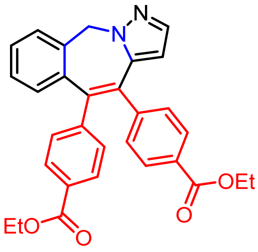

3af

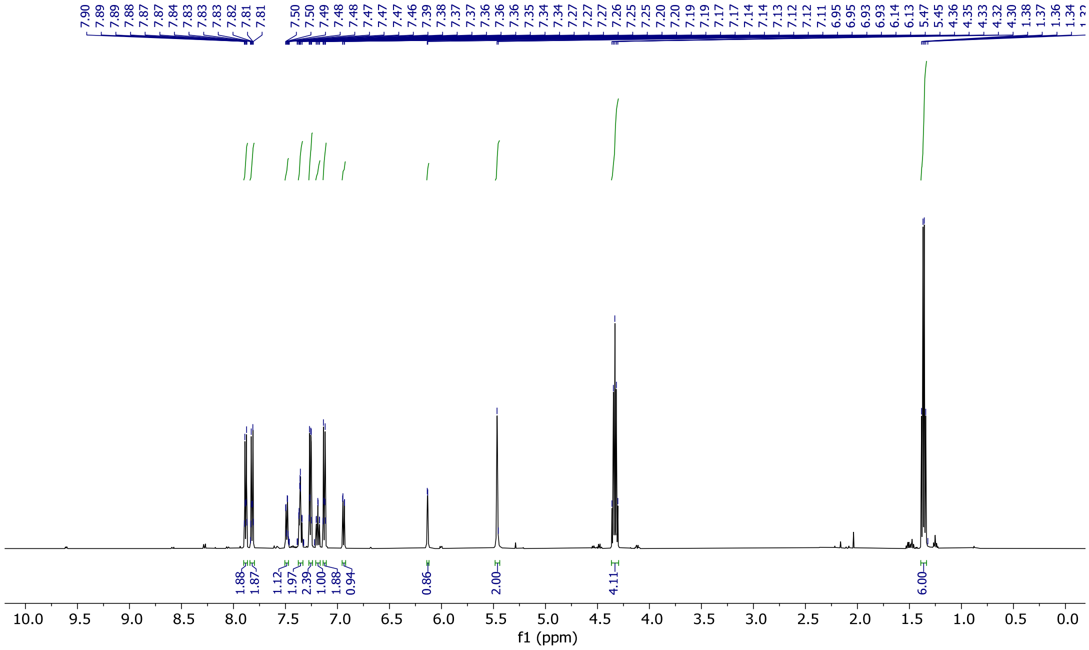

^{13}C NMR (126 MHz, CDCl$_3$)

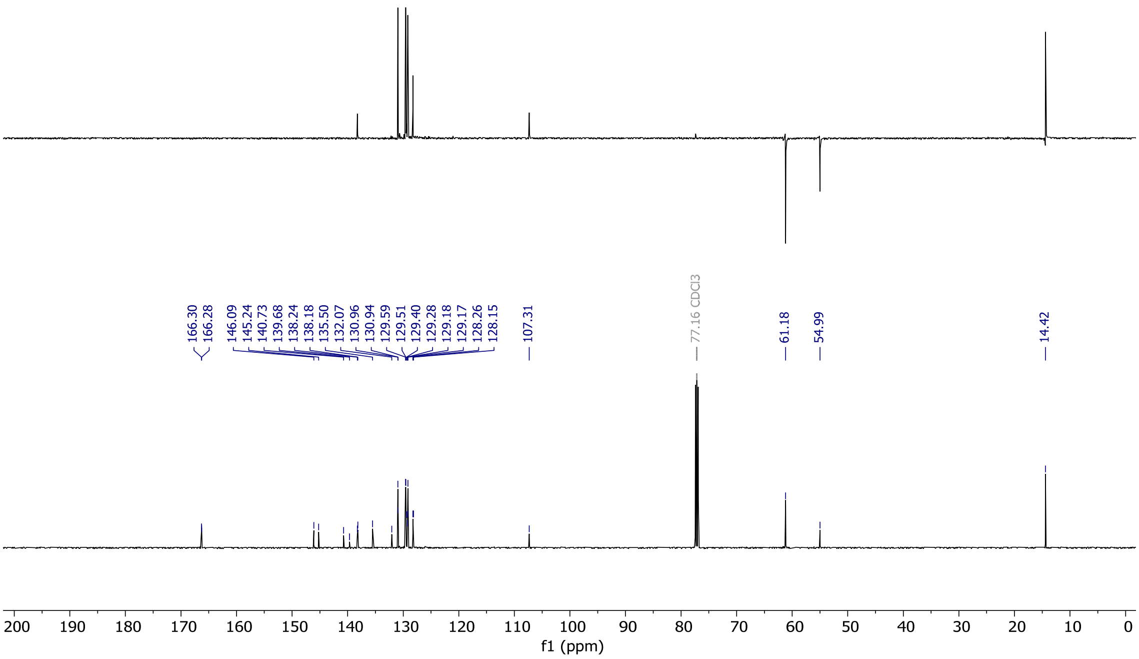

^{1}H NMR (500 MHz, $CDCl_3$)

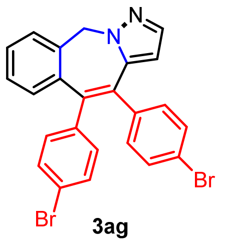

3ag

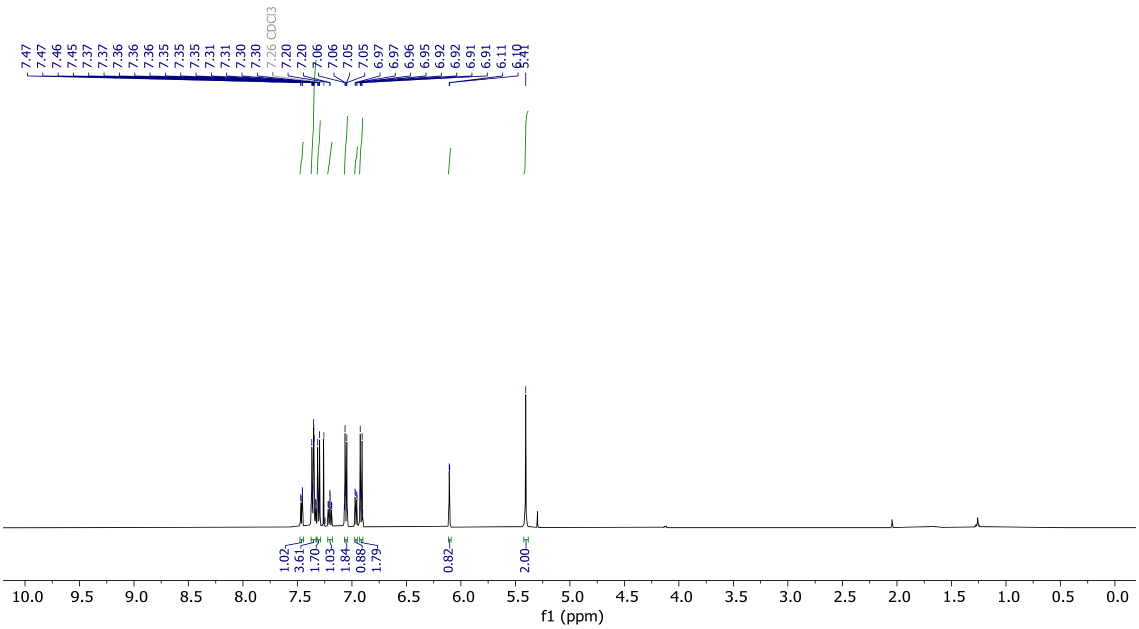

^{13}C NMR (126 MHz, $CDCl_3$)

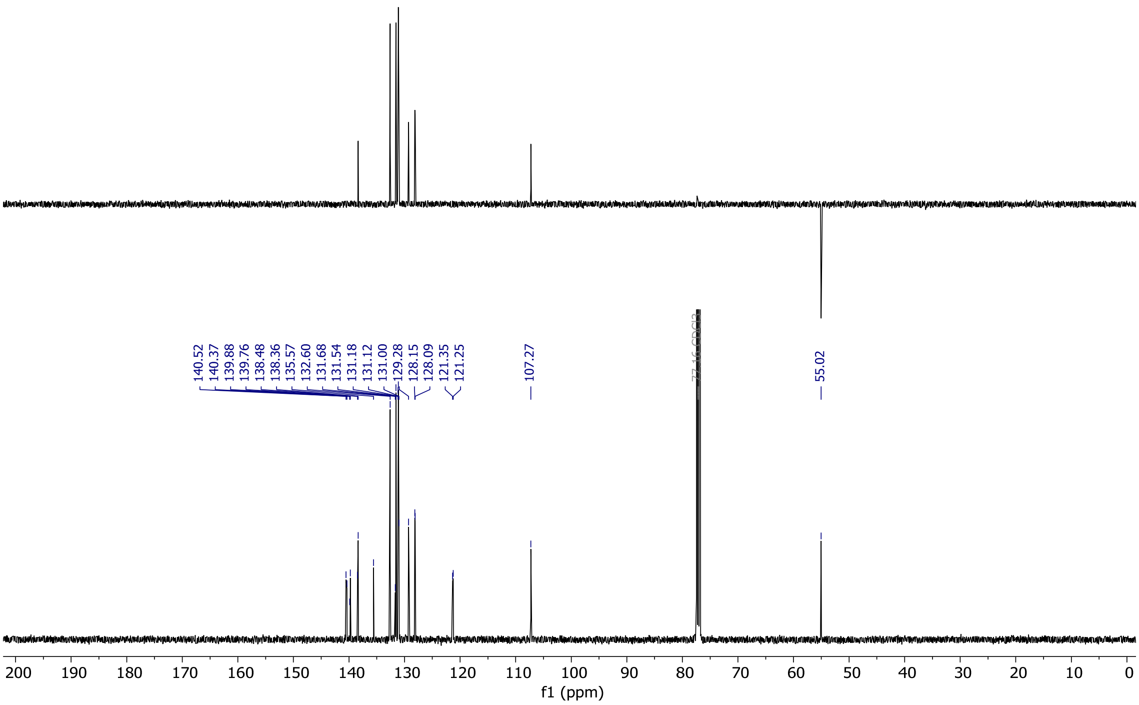

^{1}H NMR (500 MHz, $CDCl_3$)

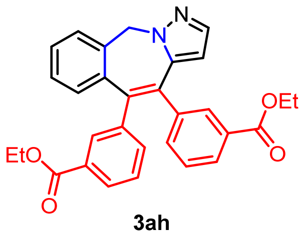

3ah

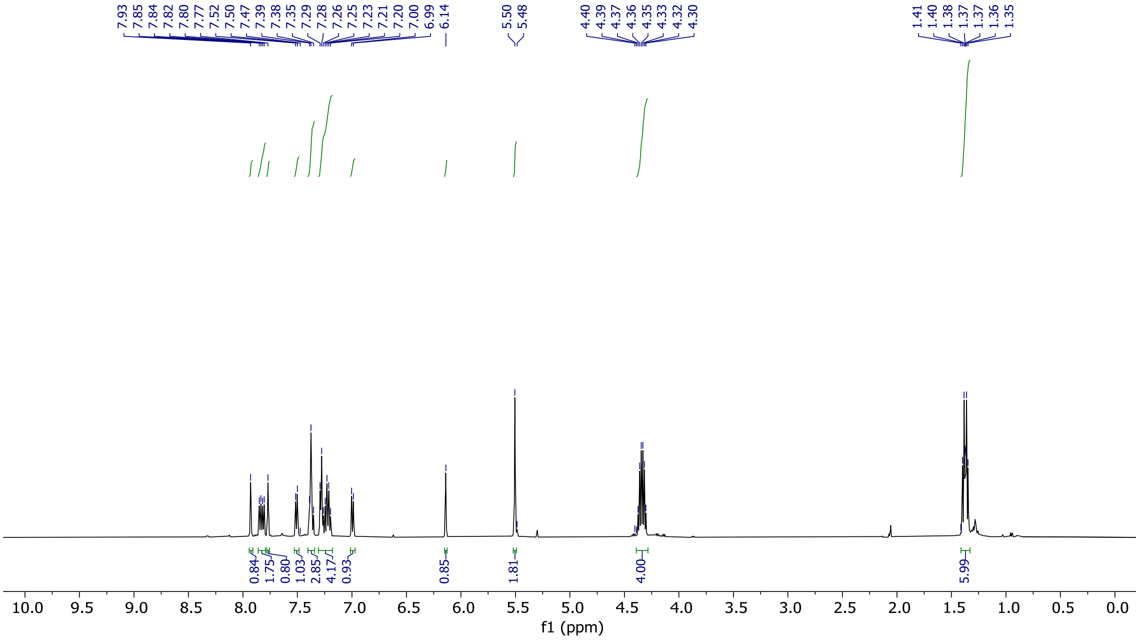

^{13}C NMR (126 MHz, $CDCl_3$)

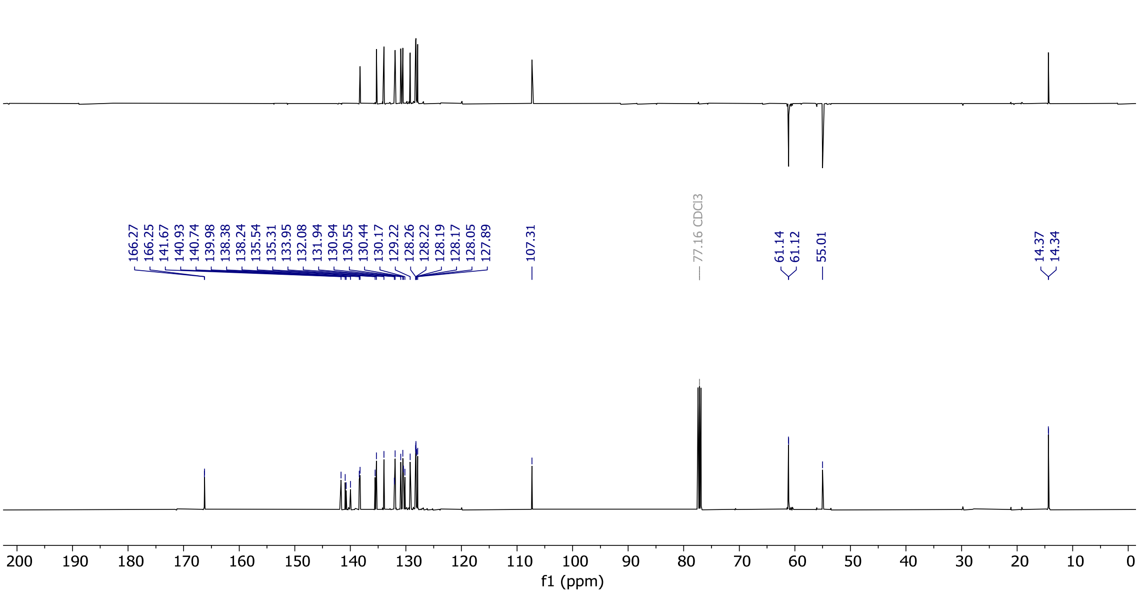

^{1}H NMR (500 MHz, $CDCl_3$)

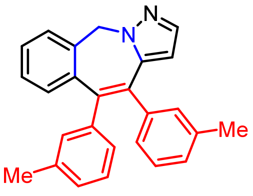

3ai

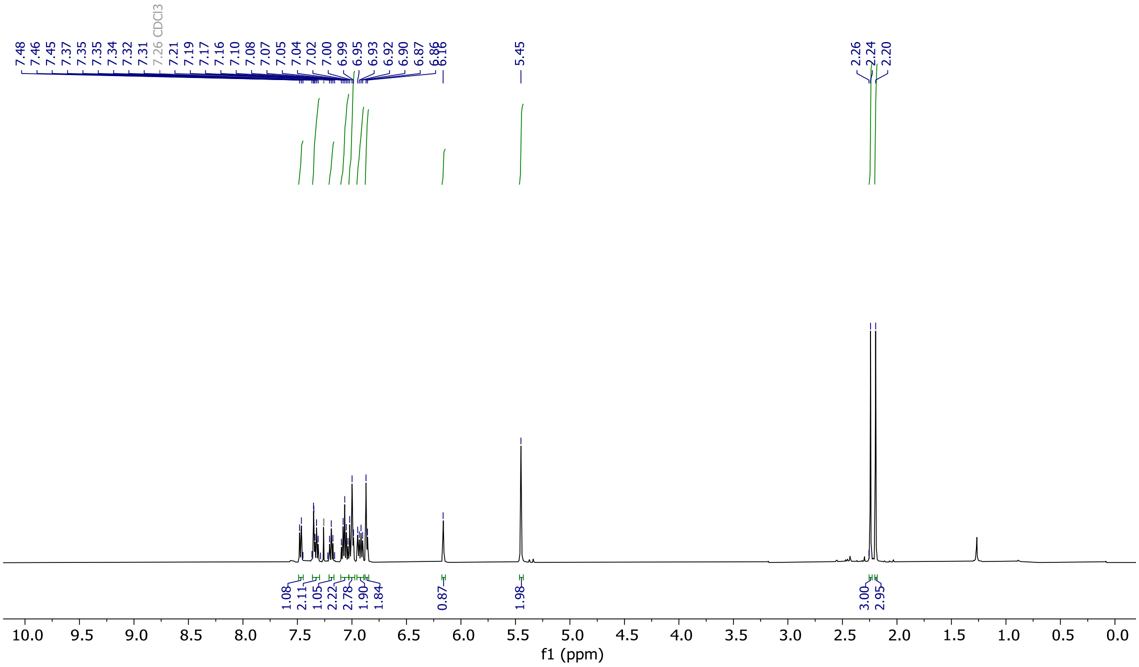

^{13}C NMR (126 MHz, $CDCl_3$)

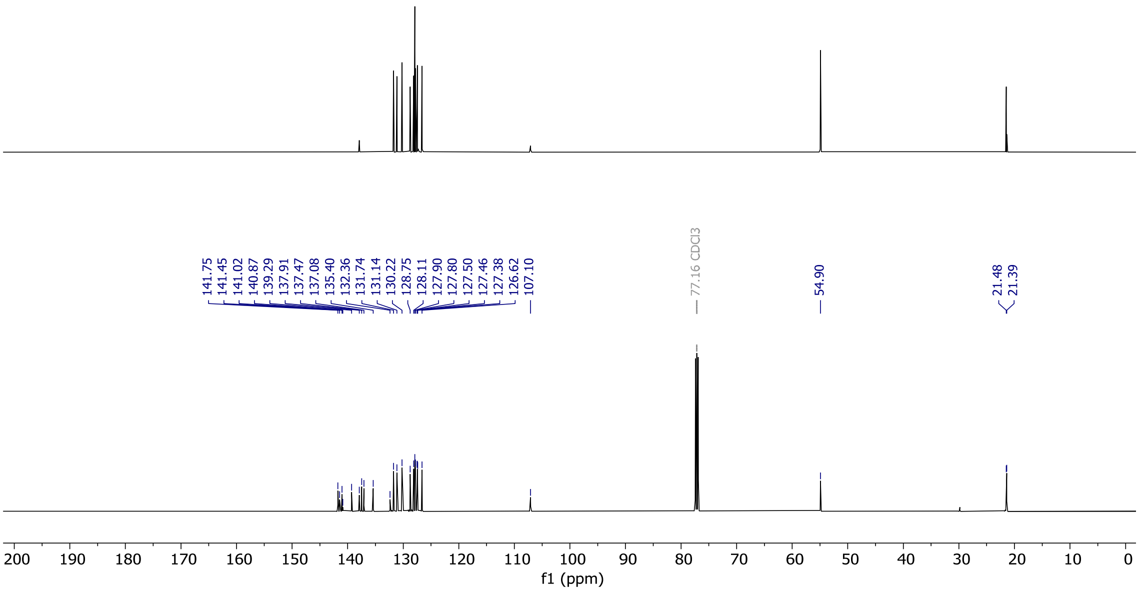

^{1}H NMR (500 MHz, $CDCl_3$)

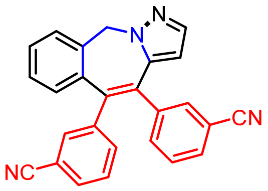

3aj

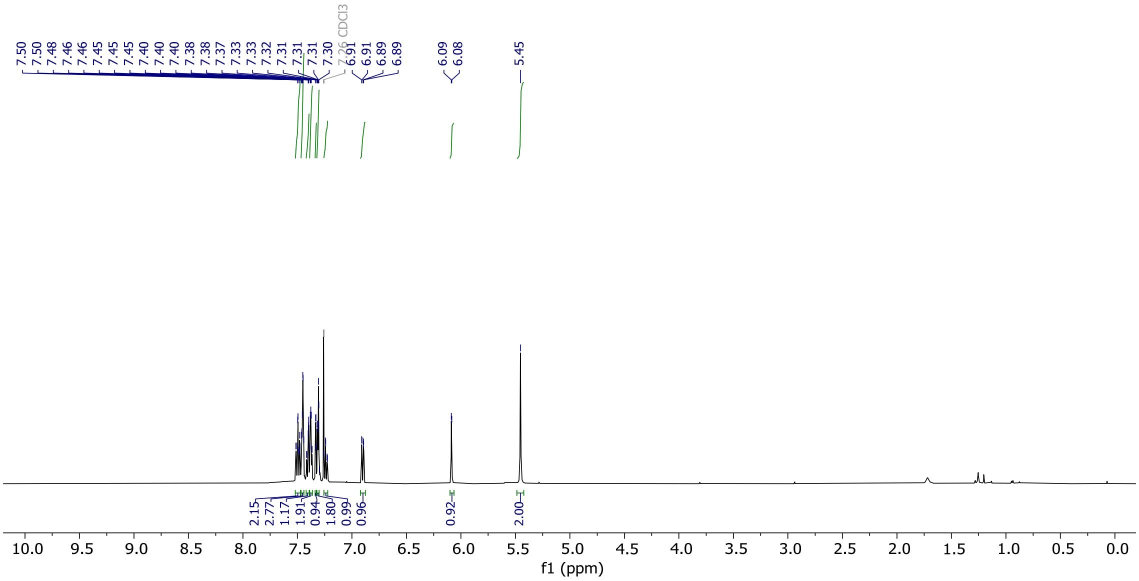

^{13}C NMR (126 MHz, $CDCl_3$)

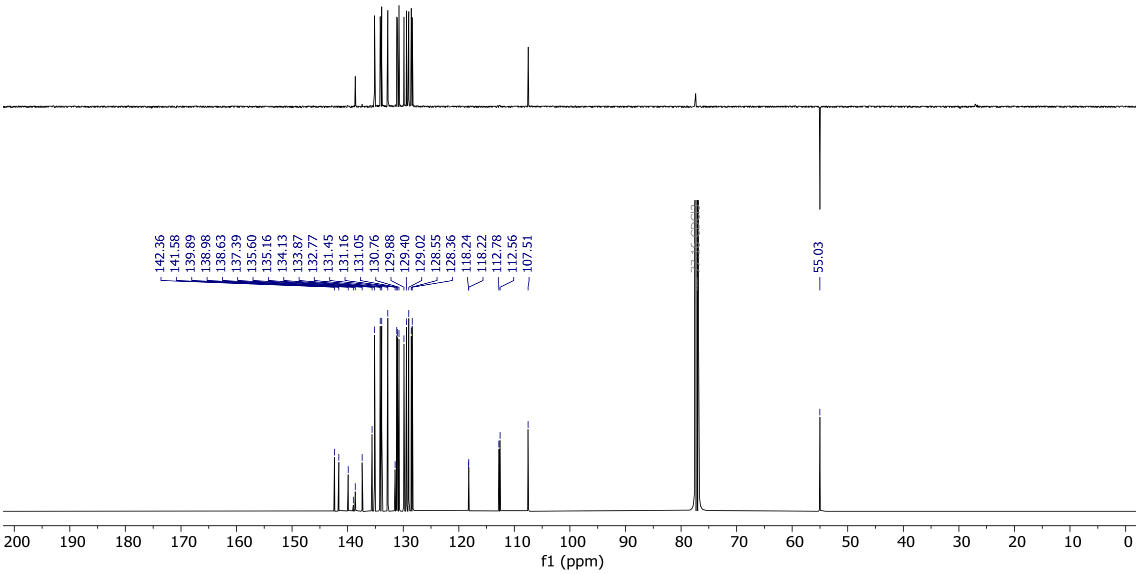

^{1}H NMR (500 MHz, $CDCl_3$)

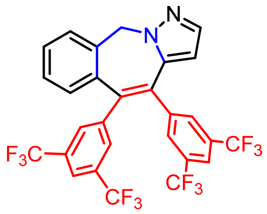

3ak

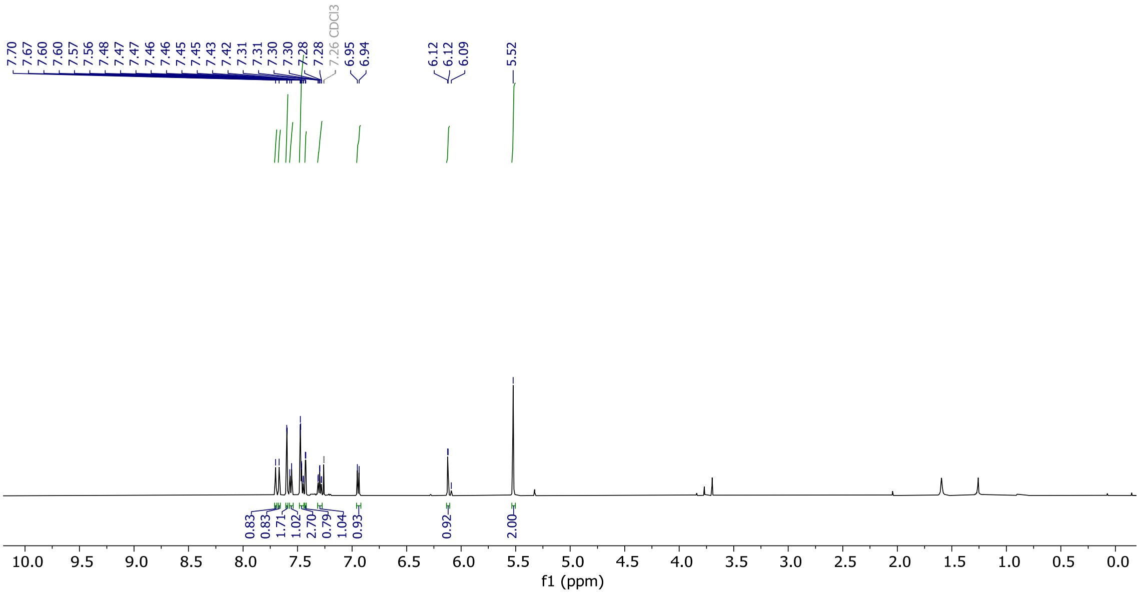

^{13}C NMR (126 MHz, $CDCl_3$)

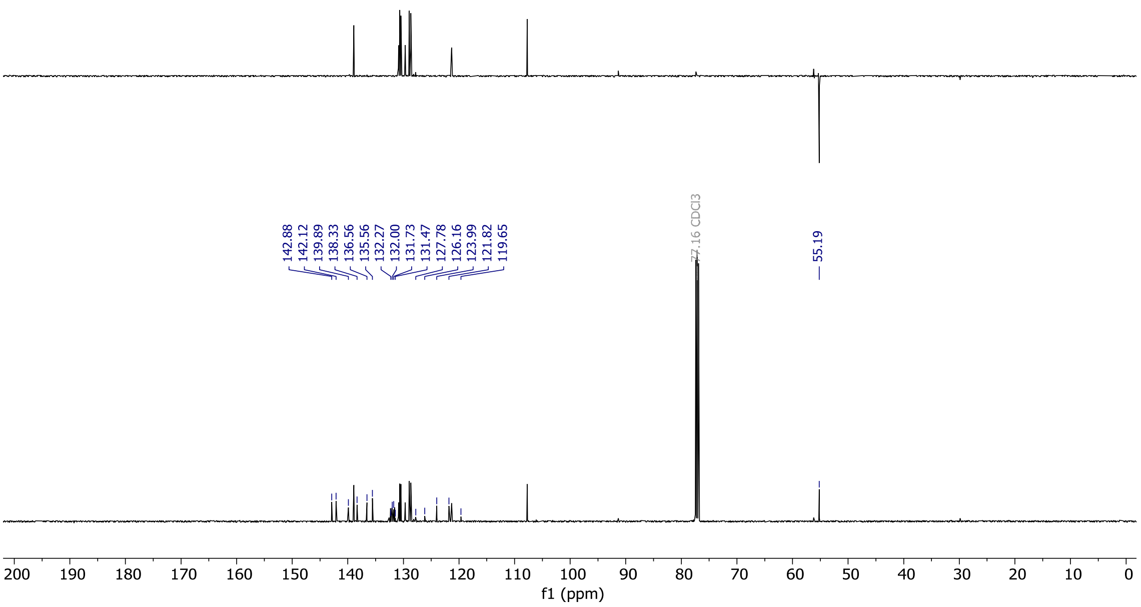

^{19}F NMR (471 MHz, $CDCl_3$)

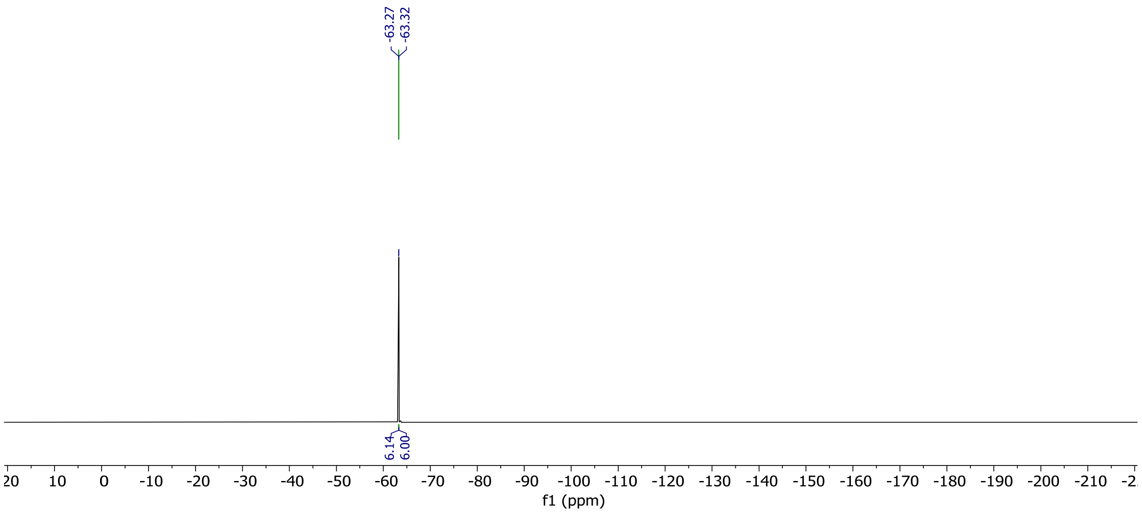

^{1}H NMR (500 MHz, $CDCl_3$)

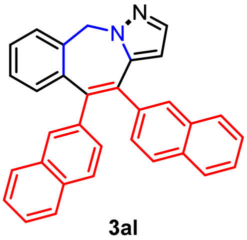

3al

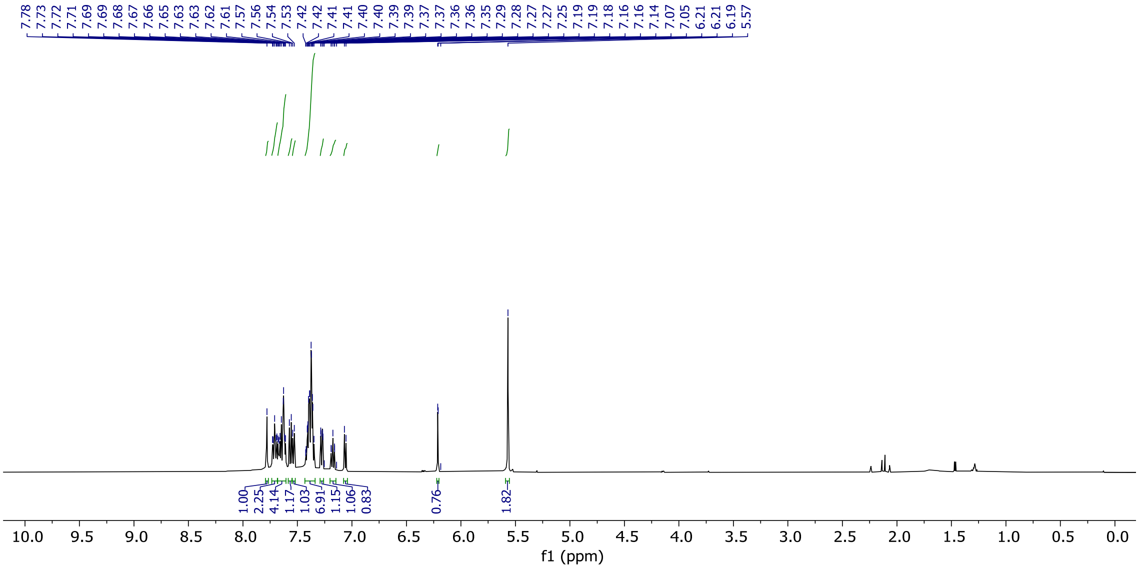

^{13}C NMR (126 MHz, $CDCl_3$)

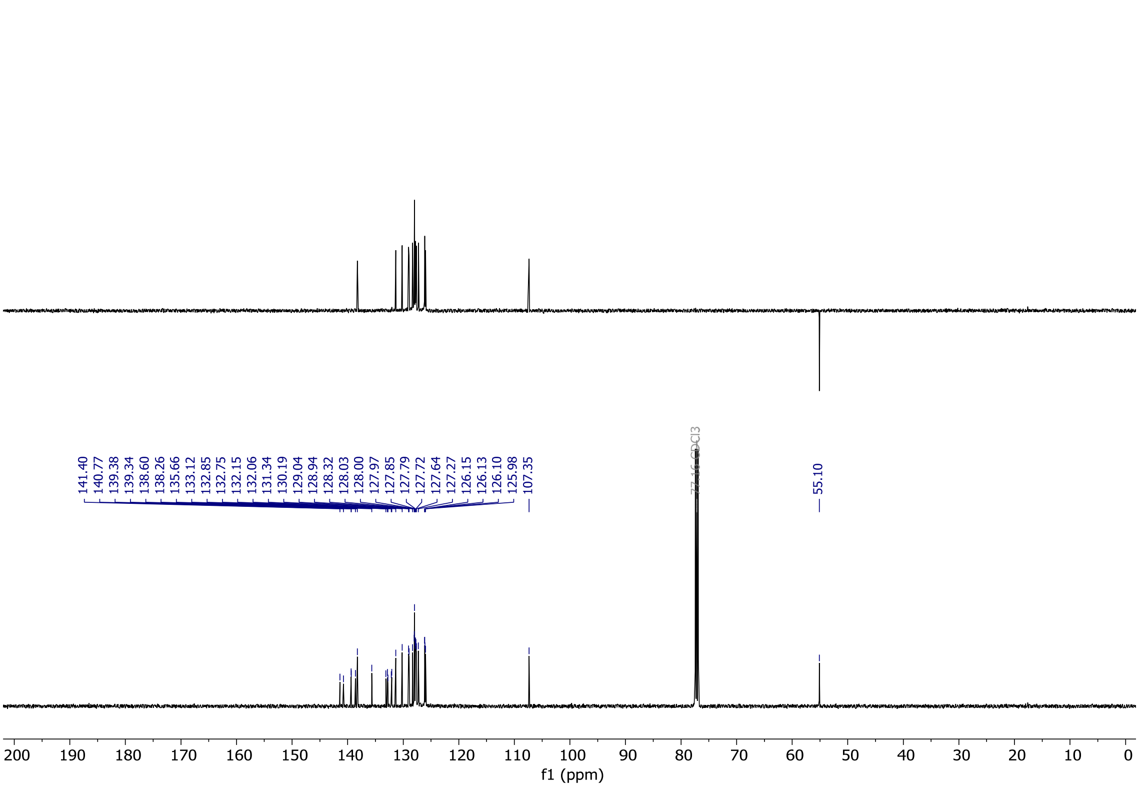

^{1}H NMR (500 MHz, CDCl$_3$)

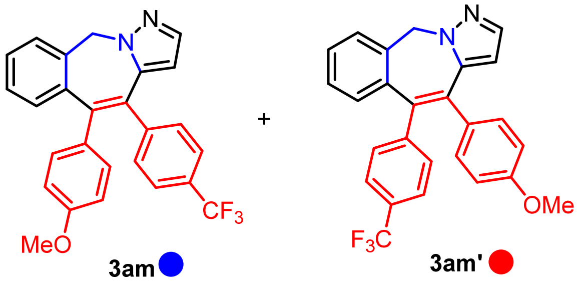

Ratio 1.6:1

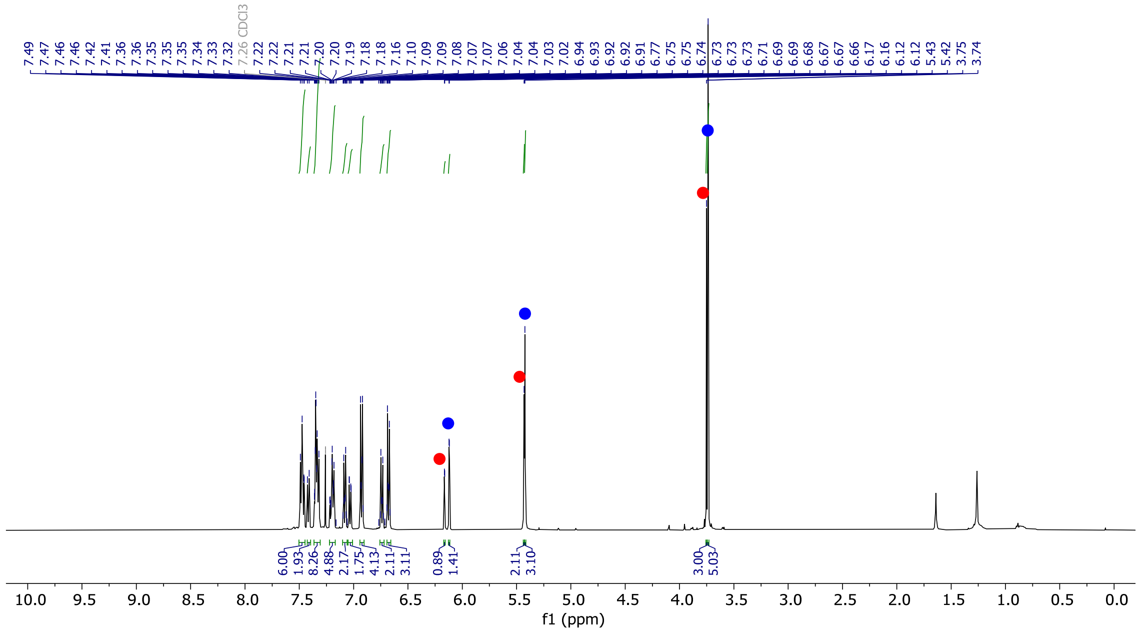

^{13}C NMR (126 MHz, CDCl$_3$)

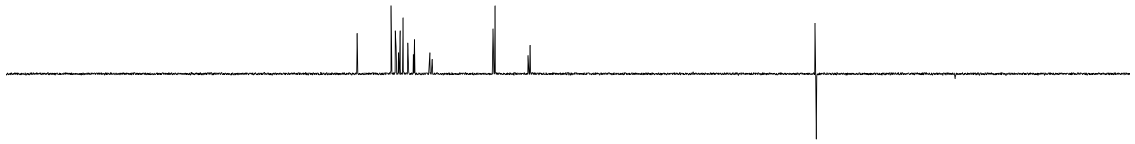

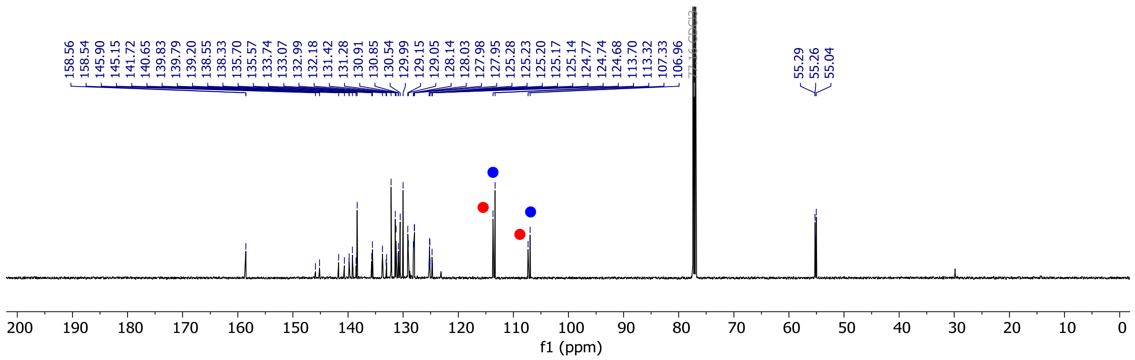

^{19}F NMR (471 MHz, CDCl$_3$)

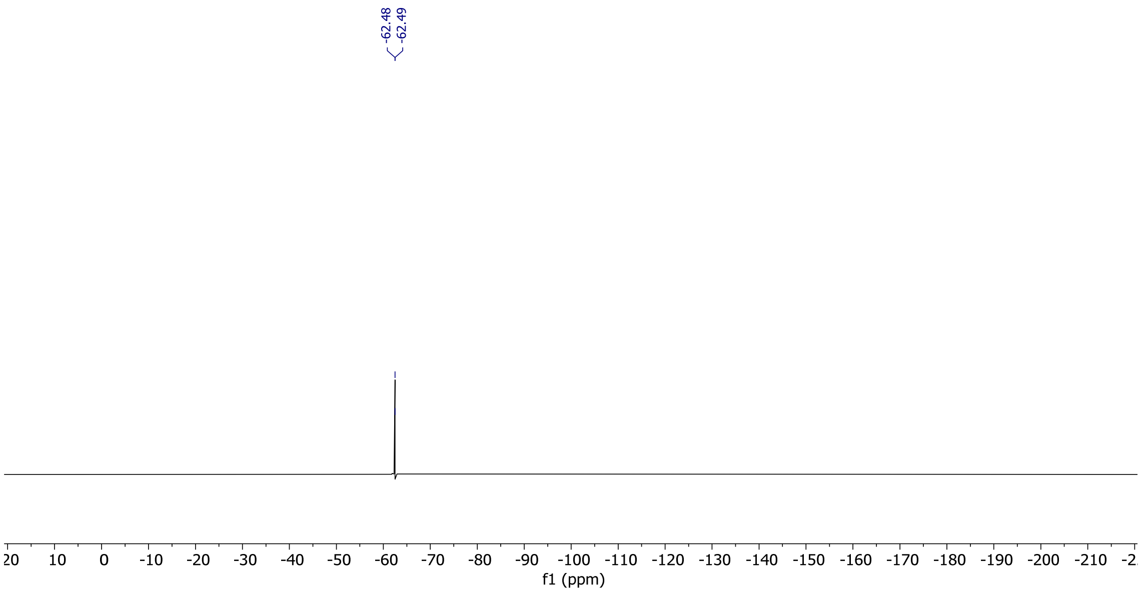

^{1}H NMR (500 MHz, $CDCl_3$)

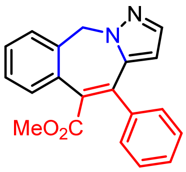

3an

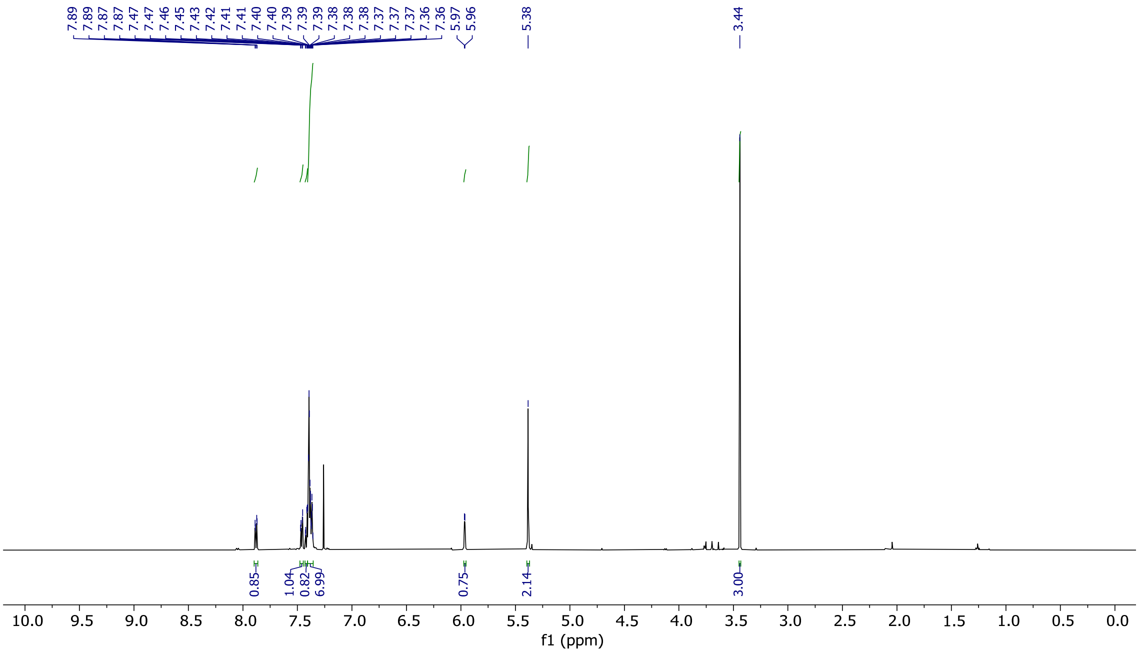

^{13}C NMR (126 MHz, $CDCl_3$)

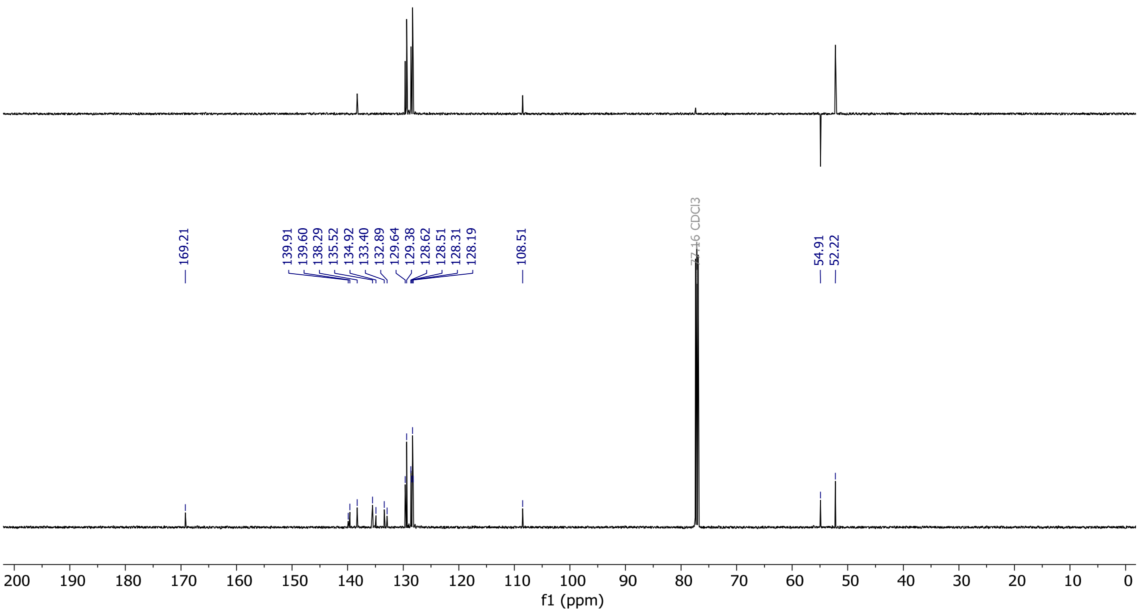

^{1}H, ^{13}C-HSQC NMR

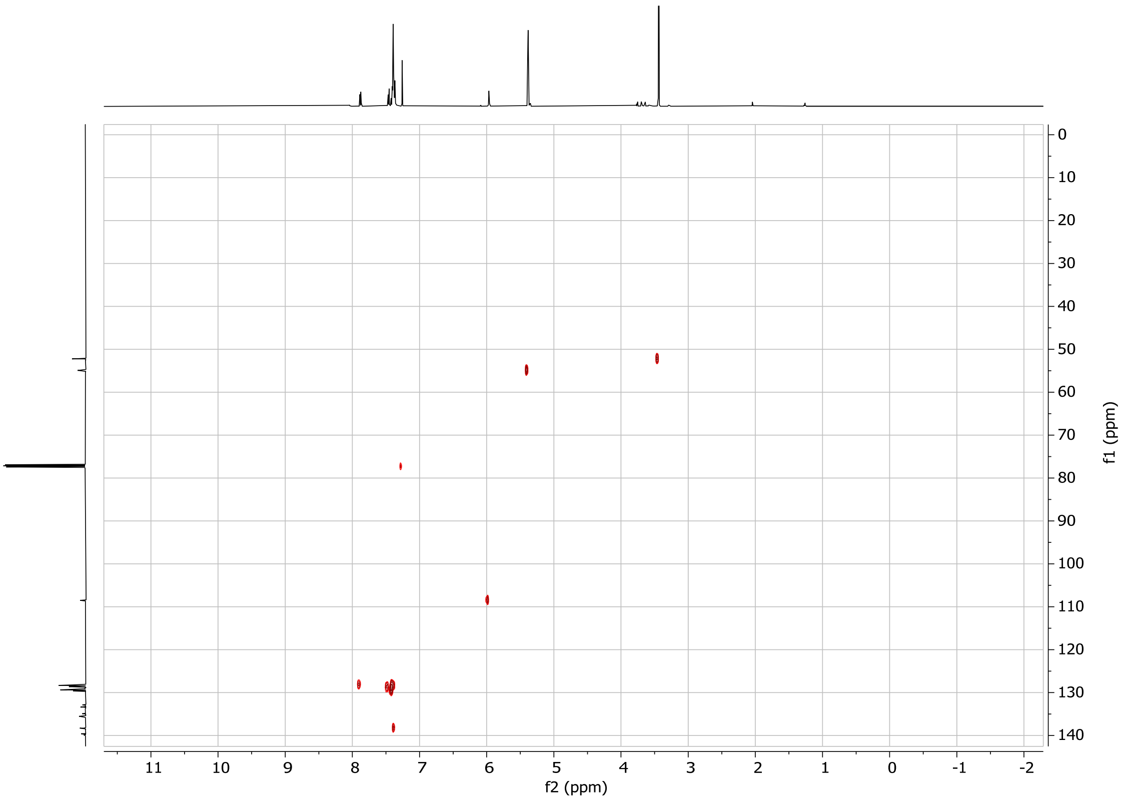

^{1}H, ^{13}C-HMBC

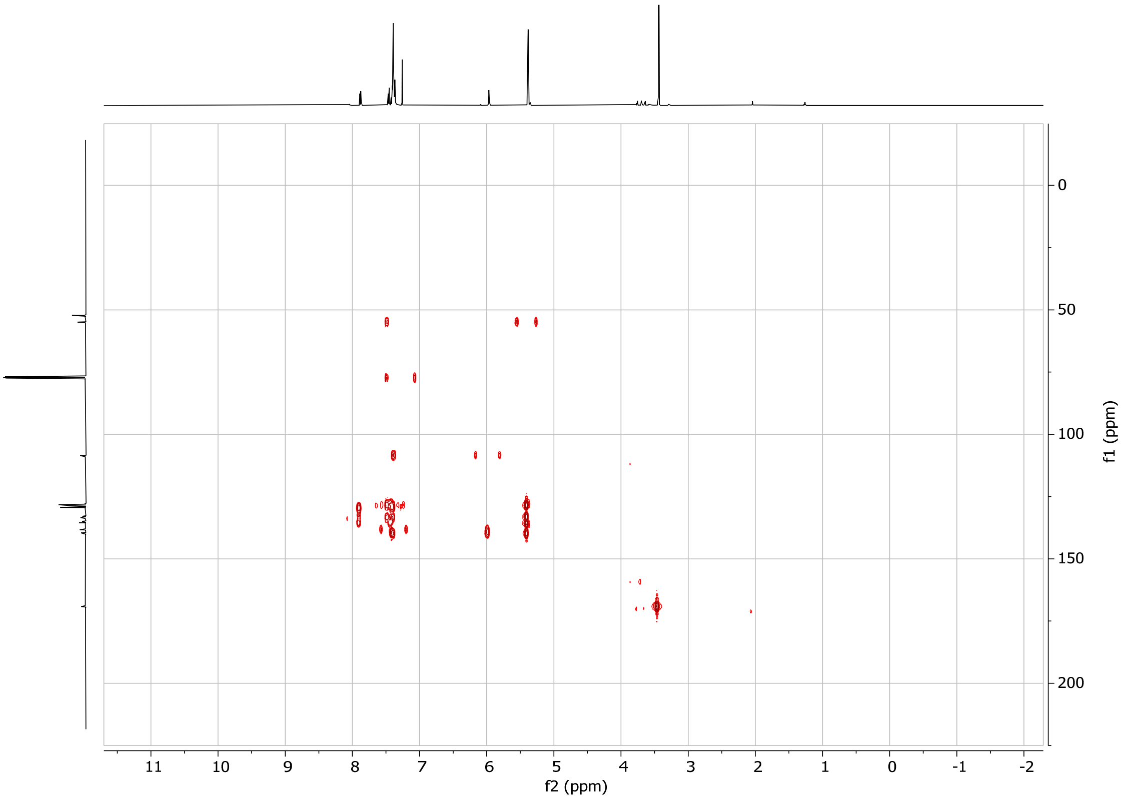

^{1}H-^{1}H NOESY

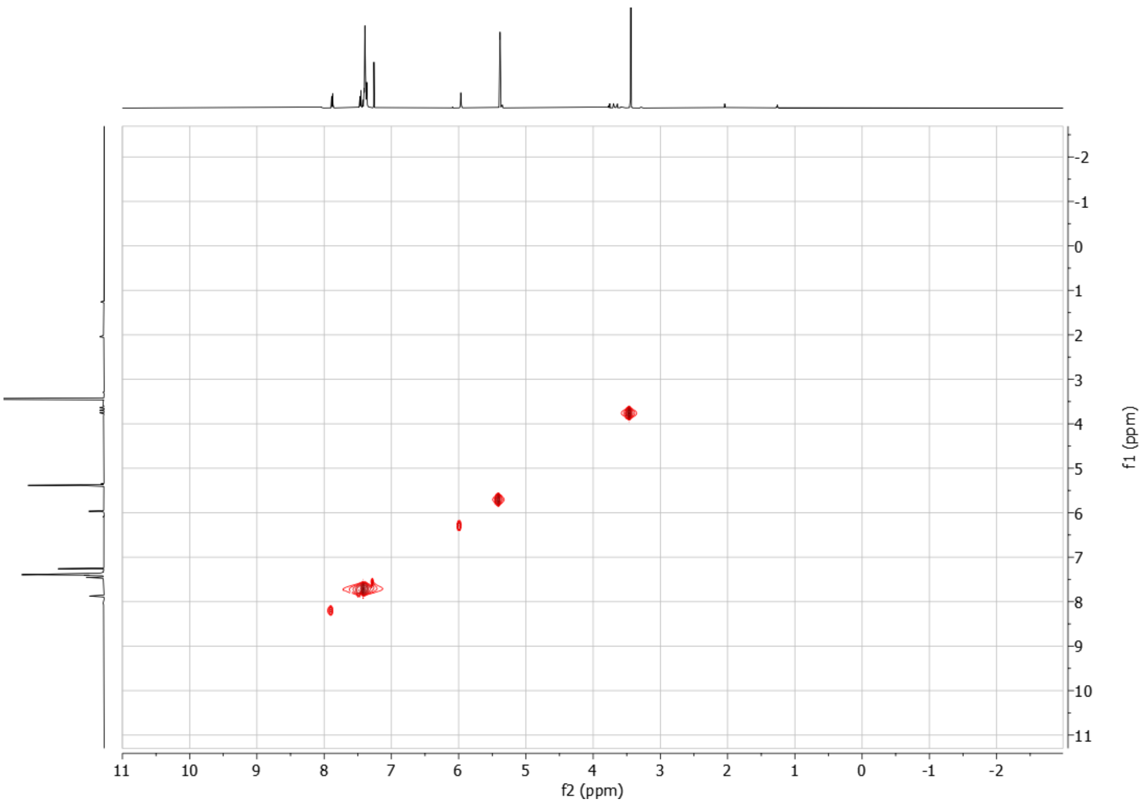

^{1}H NMR (500 MHz, $CDCl_3$)

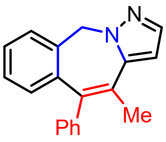

3ao

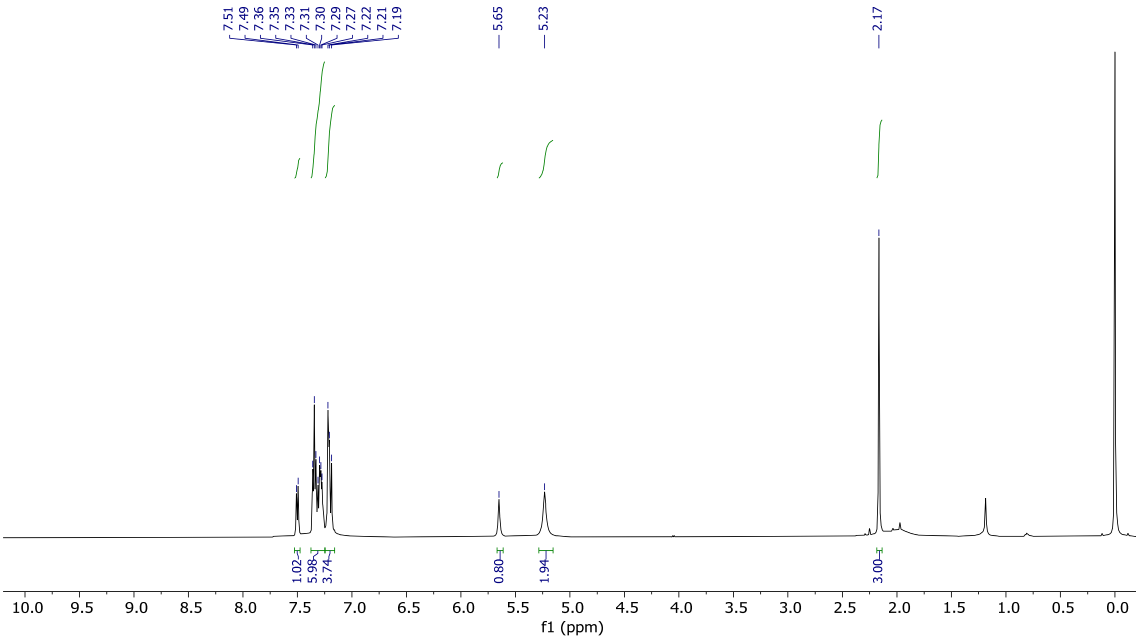

^{13}C NMR (126 MHz, $CDCl_3$)

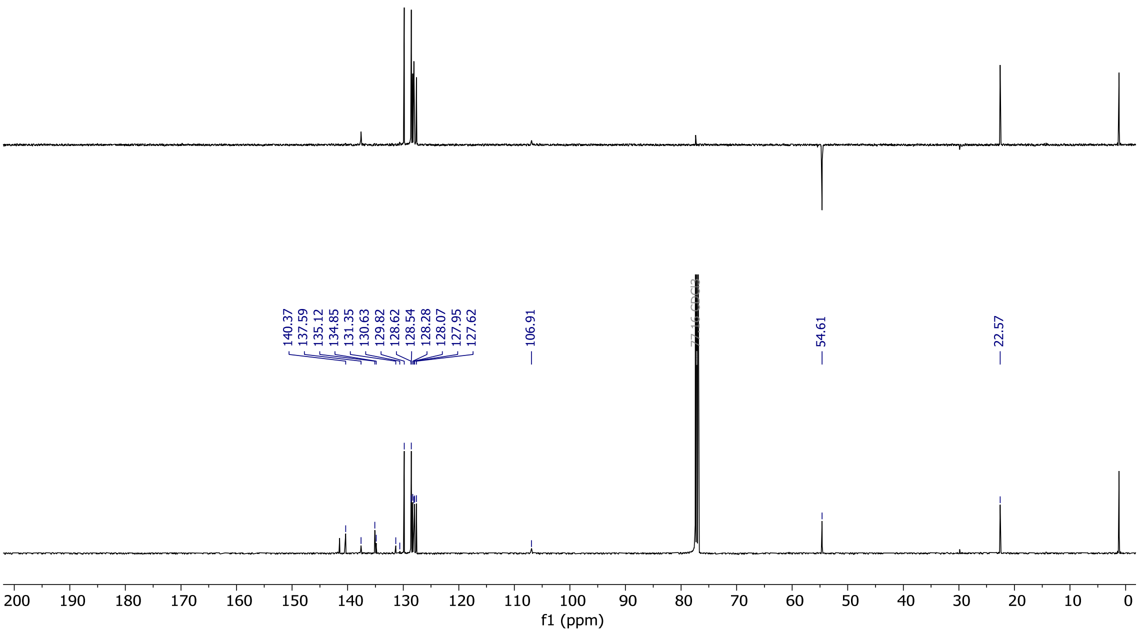

HSQC

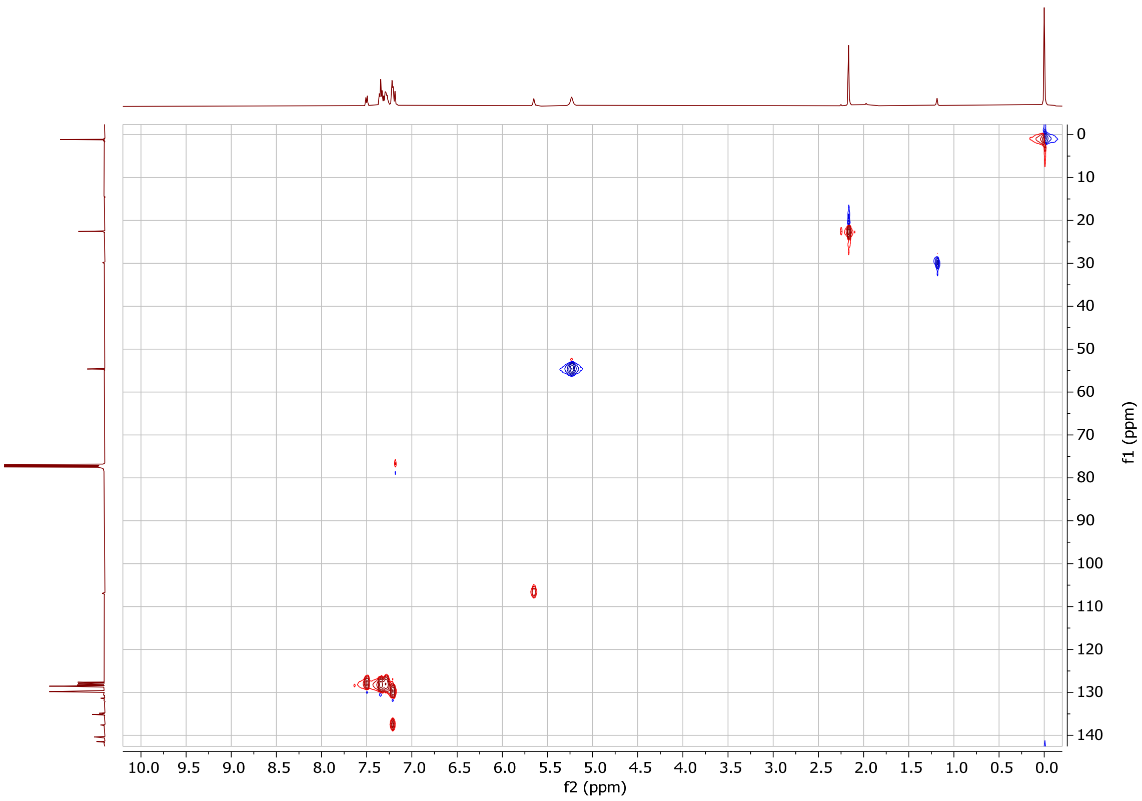

HMBC

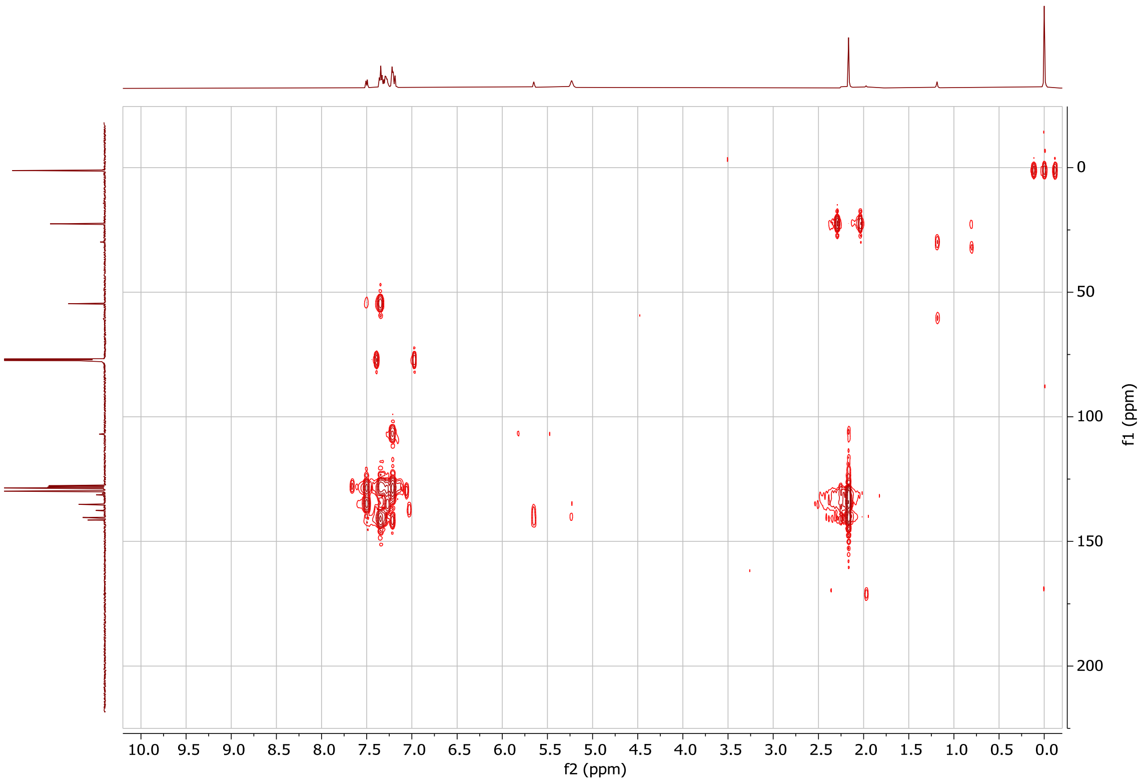

NOESY

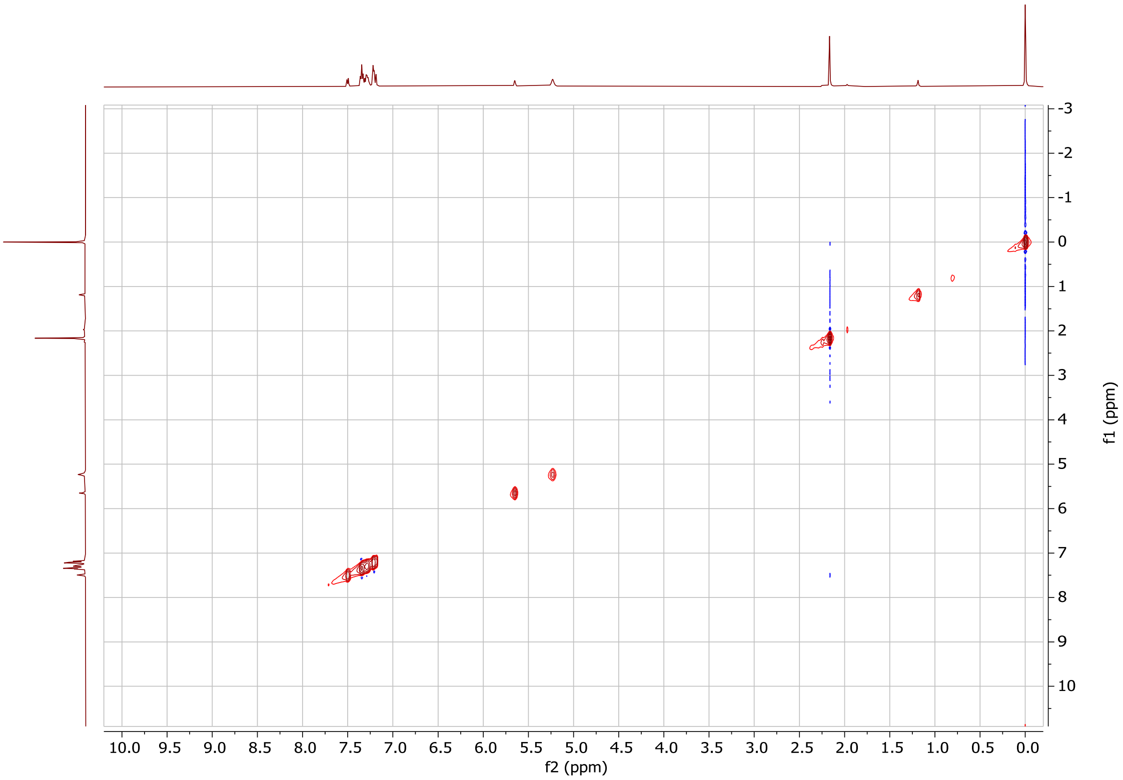

^{1}H NMR (500 MHz, $CDCl_3$)

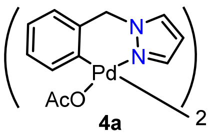

4a

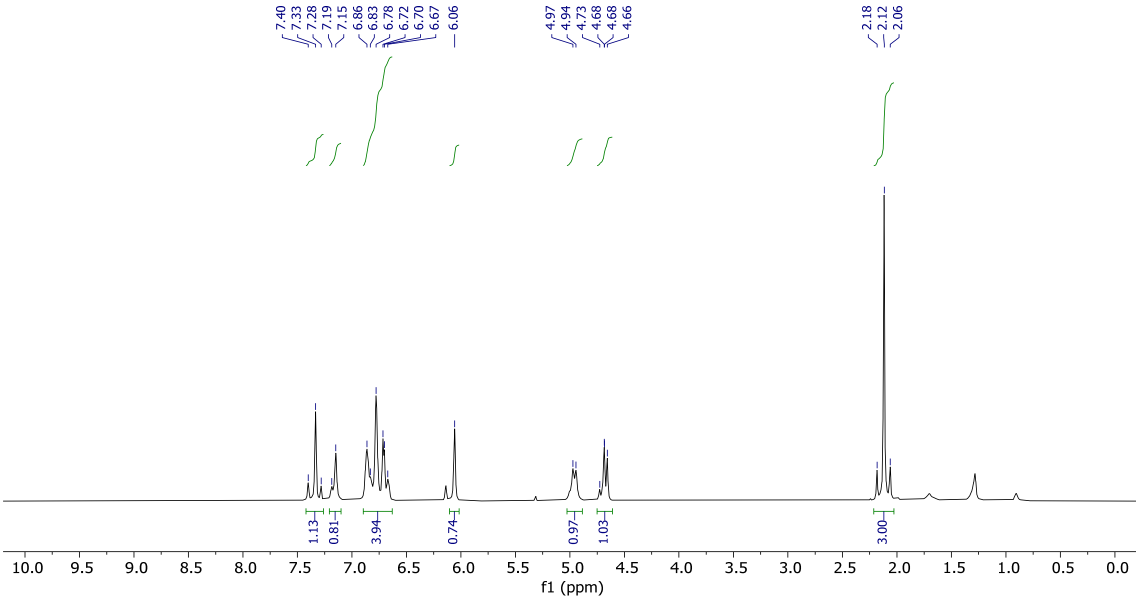

^{13}C NMR (126 MHz, $CDCl_3$)

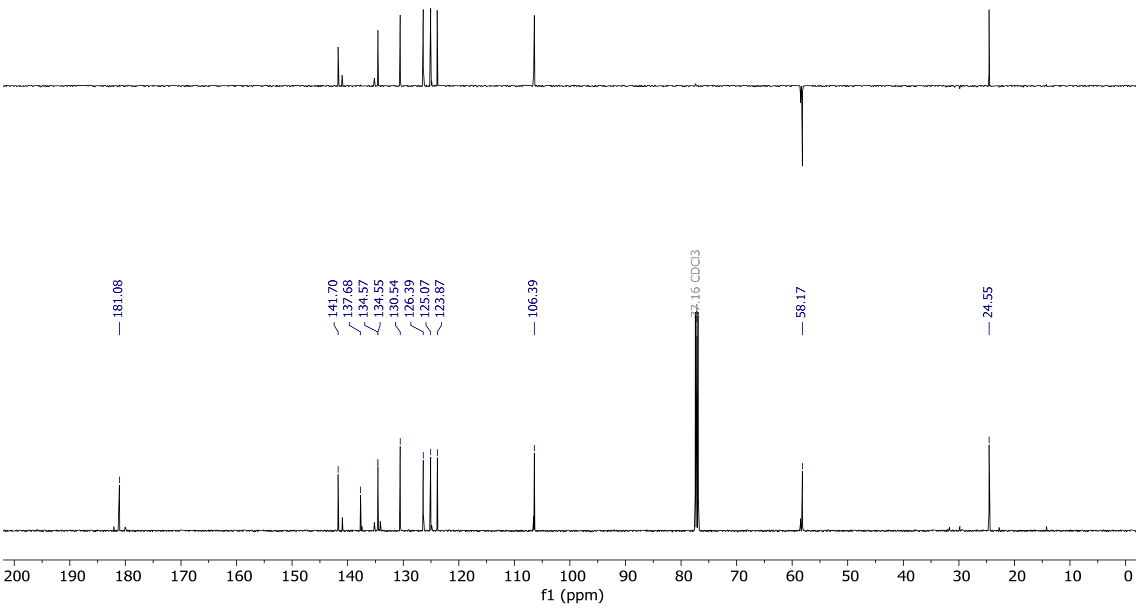

^1H NMR (500 MHz, $CDCl_3$)

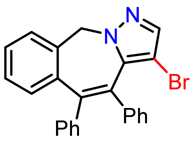

5

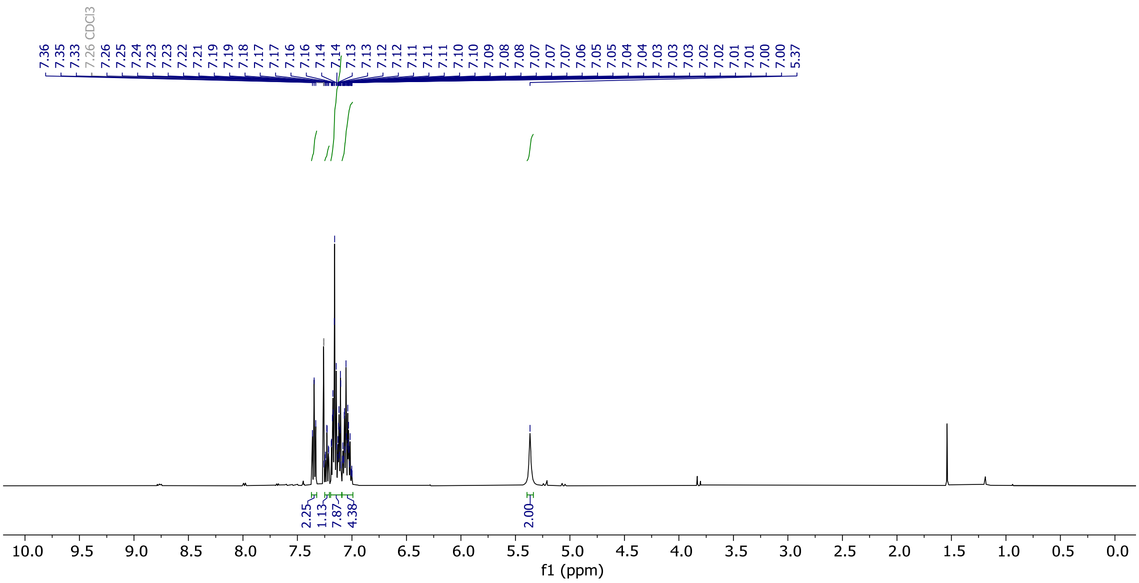

^{13}C NMR (126 MHz, $CDCl_3$)

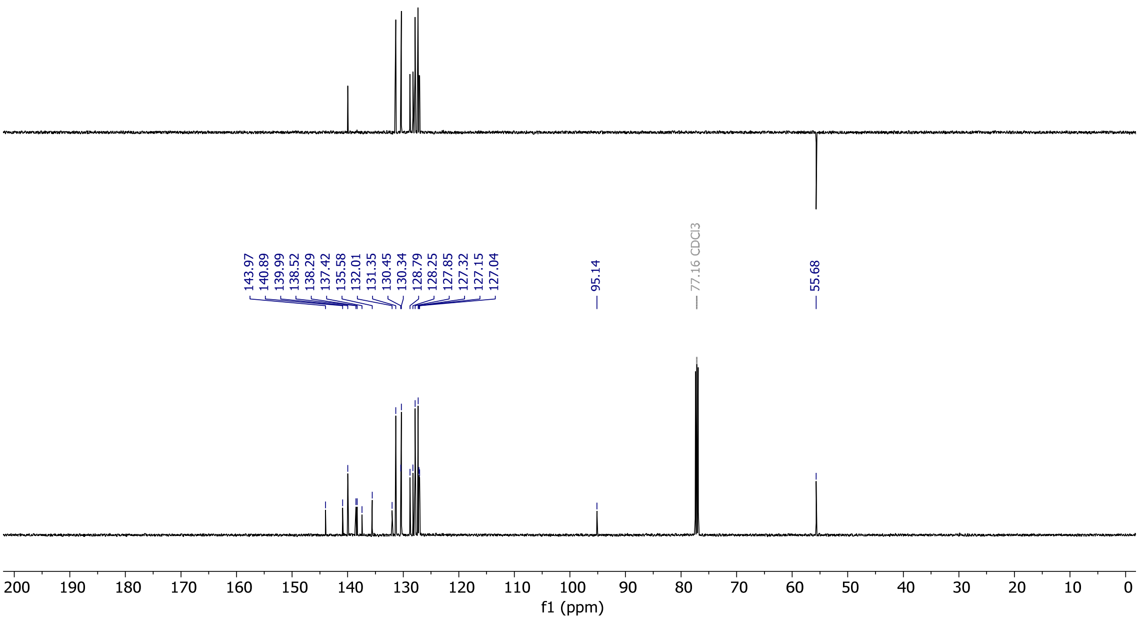

^{1}H NMR (500 MHz, CDCl$_3$)

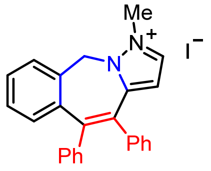

6

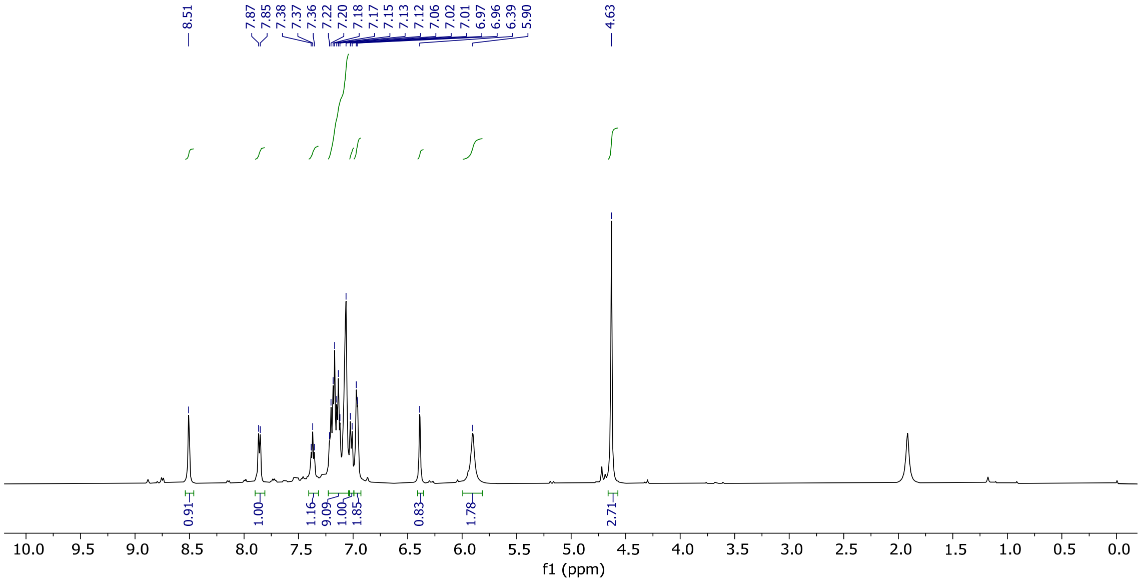

^{13}C NMR (126 MHz, CDCl$_3$)

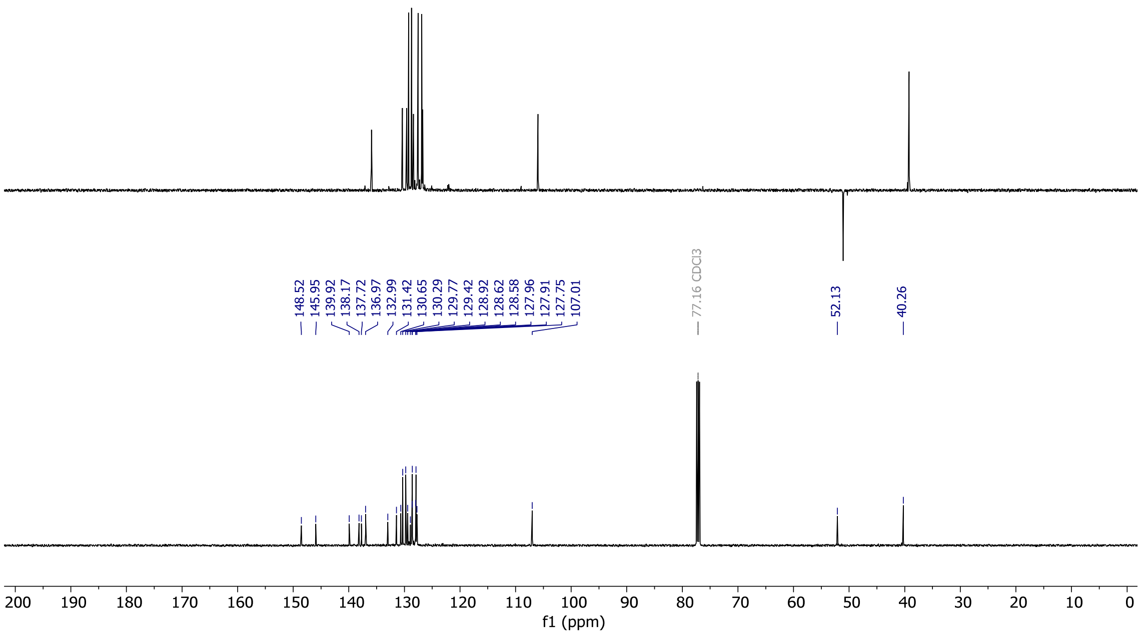

^{1}H NMR (500 MHz, CDCl$_3$)

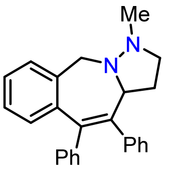

7

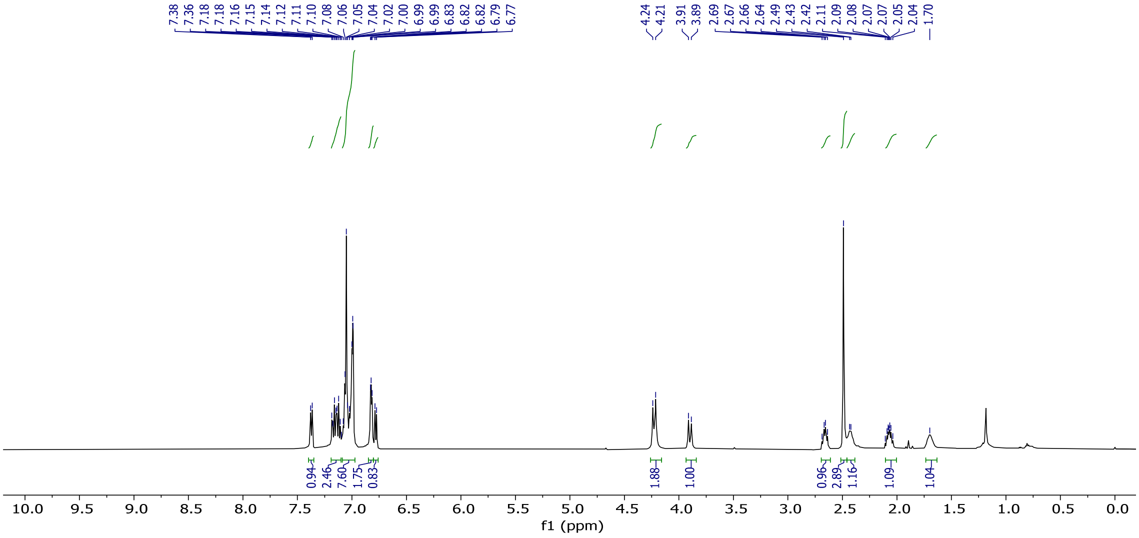

^{13}C NMR (126 MHz, CDCl$_3$)

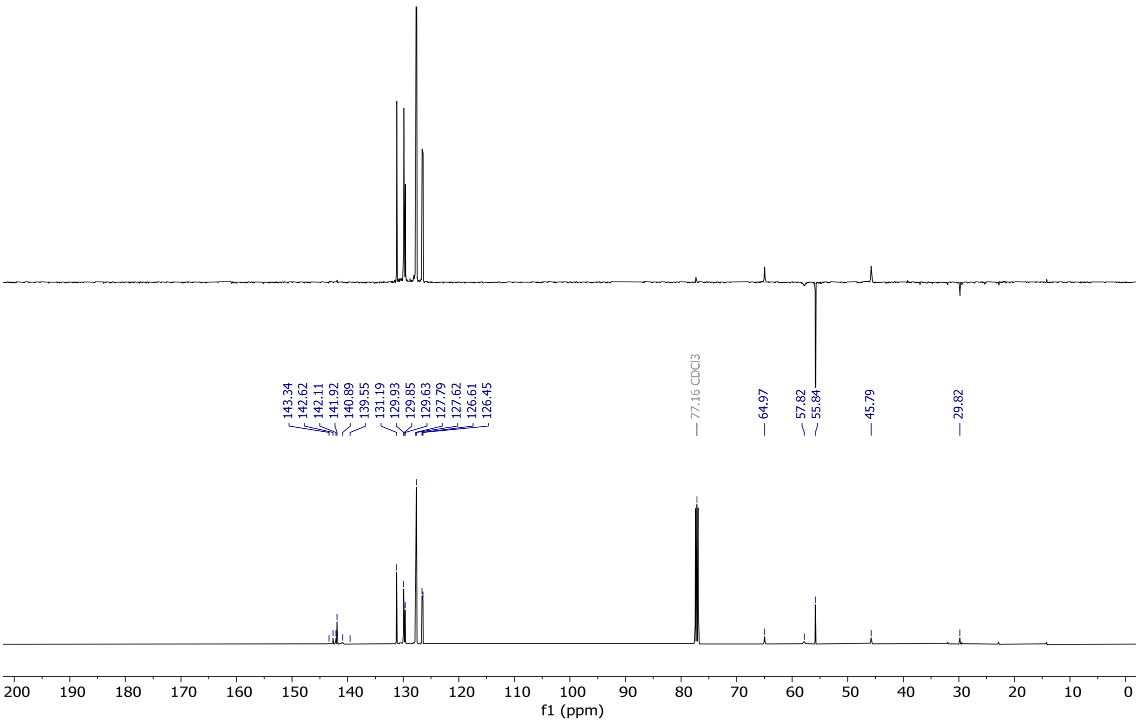

10. Computational details

All electronic structure calculations were performed using the Gaussian 16 software package[16] at the CESGA facilities. The geometries of all minima and transition states involved were optimized at the B3LYP[17] level with the addition of Grimme´s D3 dispersion corrections[18] (B3LYP-D3) within the self-consistent reaction field (SCRF) using the SMD model (*N*,*N*-dimethylformamide)[19] with a small basis set (BS1) featuring the double-ζ LANL2DZ[20] basis set and the LANLD2Z pseudopotential[21] to describe the electrons of the palladium and silver atoms and the 6-31G(d,p) basis set[22] for all other atoms. Frequency calculations were performed at the same level to evaluate the zero-point vibrational energy and thermal corrections at 298 K and to confirm the nature of the stationary points, yielding one imaginary frequency for the transition states and none for the minima. Each transition state was further confirmed by following the steepest descent to both sides and identifying the minima present in the reaction energy profile. Single-point energies were calculated using B3LYP with the addition of Grimme´s D3 dispersion corrections (B3LYP-D3) within the self-consistent reaction field (SCRF) using the SMD model (*N*,*N*-dimethylformamide) and a larger basis set (BS2) featuring the cc-pVTZ-pp basis set and pseudopotential[23] for the palladium and silver atoms and 6-311++G(d,p)[22e,24] for all other atoms. The resulting energies were used to correct the gas-phase energies obtained from B3LYP calculations. The reaction profiles were built up in terms of ΔG_{sol}.

11. DFT study for the [5+2] rollover annulation of 1-benzylpyrazole (1a) with 1,2-diphenylacetylene (2a) catalyzed by Pd^{II} monometallic species

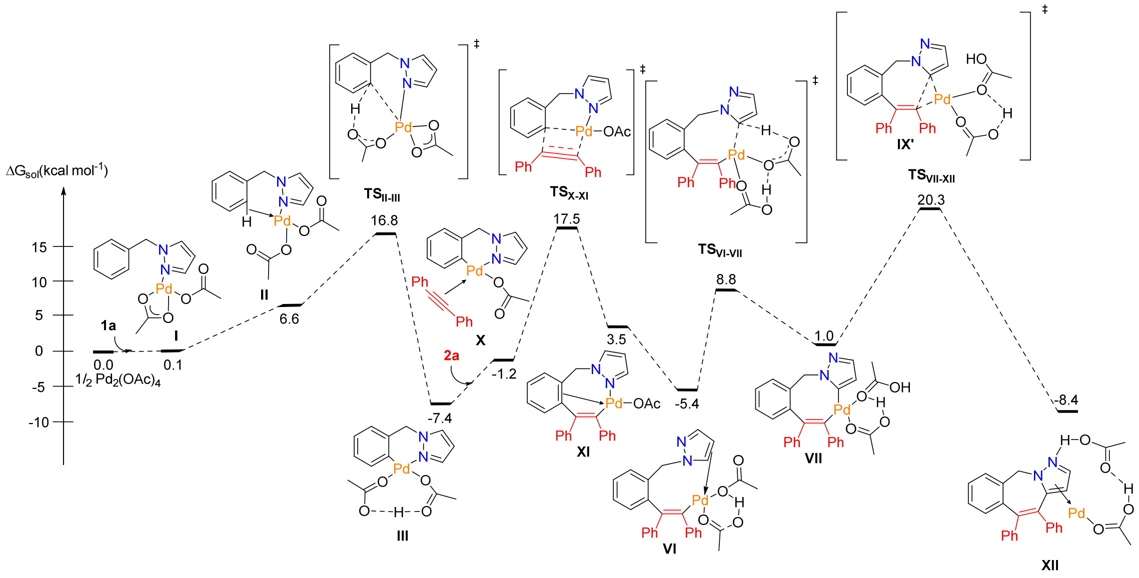

Figure S3. Free energy profile for the [5+2] rollover annulation of 1-benzylpyrazole (**1a**) with 1,2-diphenylacetylene (**2a**) catalyzed by Pd^{II} complexes. Computational studies were performed at B3LYP-D3/6-311++G(d,p)-cc-pVTZ-pp$_{DMF(SMD)}$//B3LYP-D3/6-

31G(d,p)-LANL2DZ$_{DMF(SMD)}$. Energies are relative to $\frac{1}{2}$Pd$_2$(OAc)$_4$ combined with those of the relevant substrates.

12. DFT study for the [5+2] rollover annulation of 1-benzylpyrazole (1a) with 1,2-diphenylacetylene (2a) catalyzed by Pd^{II}-Ag^{I} bimetallic species

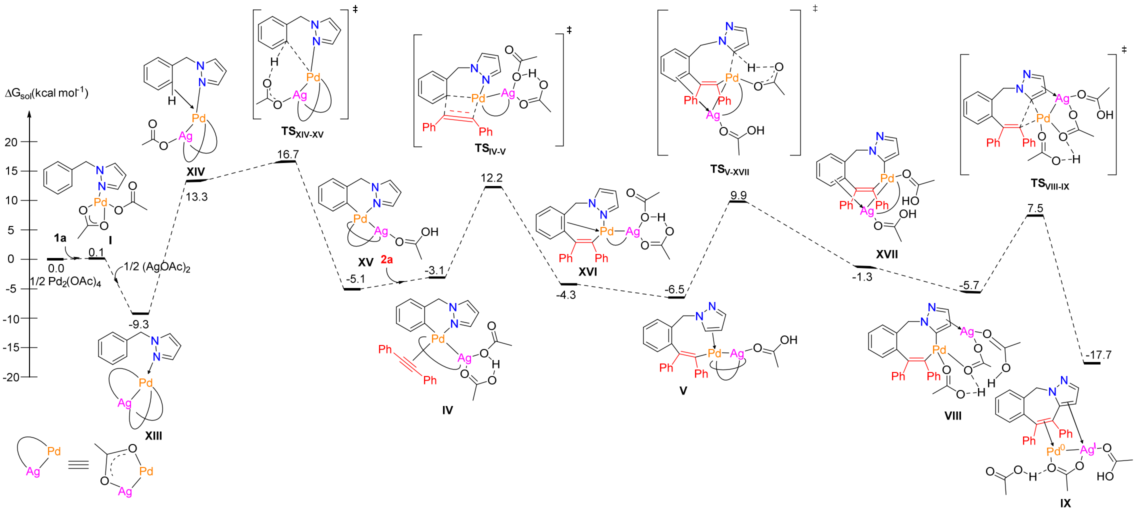

Figure S4. Free energy profile for the [5+2] rollover annulation of 1-benzylpyrazole (**1a**) with 1,2-diphenylacetylene (**2a**) catalyzed by Pd^{II}-Ag^{I} bimetallic species. Computational studies were performed at B3LYP-D3/6-311++G(d,p)-cc-pVTZ-pp$_{DMF(SMD)}$//B3LYP-D3/6-31G(d,p)-LANL2DZ$_{DMF(SMD)}$. Energies are relative to $\frac{1}{2}$Pd$_2$(OAc)$_4$ combined with those of the relevant substrates.

13.DFT study for the initial C-H activation in the pyrazole moiety catalyzed by Pd^II monometallic species

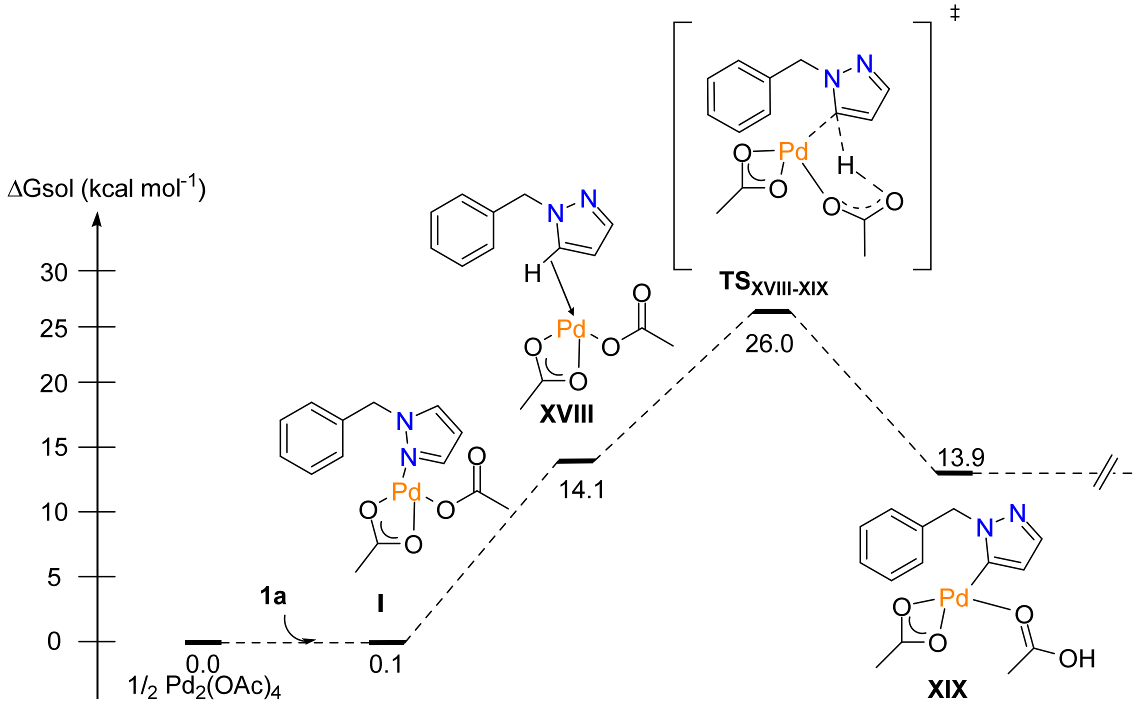

Figure S5. Free energy profile for the initial C-H activation in the pyrazole moiety catalyzed by Pd^{II} monometallic species. Computational studies were performed at B3LYP-D3/6-311++G(d,p)-cc-pVTZ-pp$_{DMF(SMD)}$//B3LYP-D3/6-31G(d,p)-LANL2DZ$_{DMF(SMD)}$. Energies are relative to $\frac{1}{2}$ $Pd_2(OAc)_4$ combined with those of the relevant substrates.

14. DFT study for the initial C-H activation in the pyrazole moiety catalyzed by Pd^{II}-Ag^{I} bimetallic species

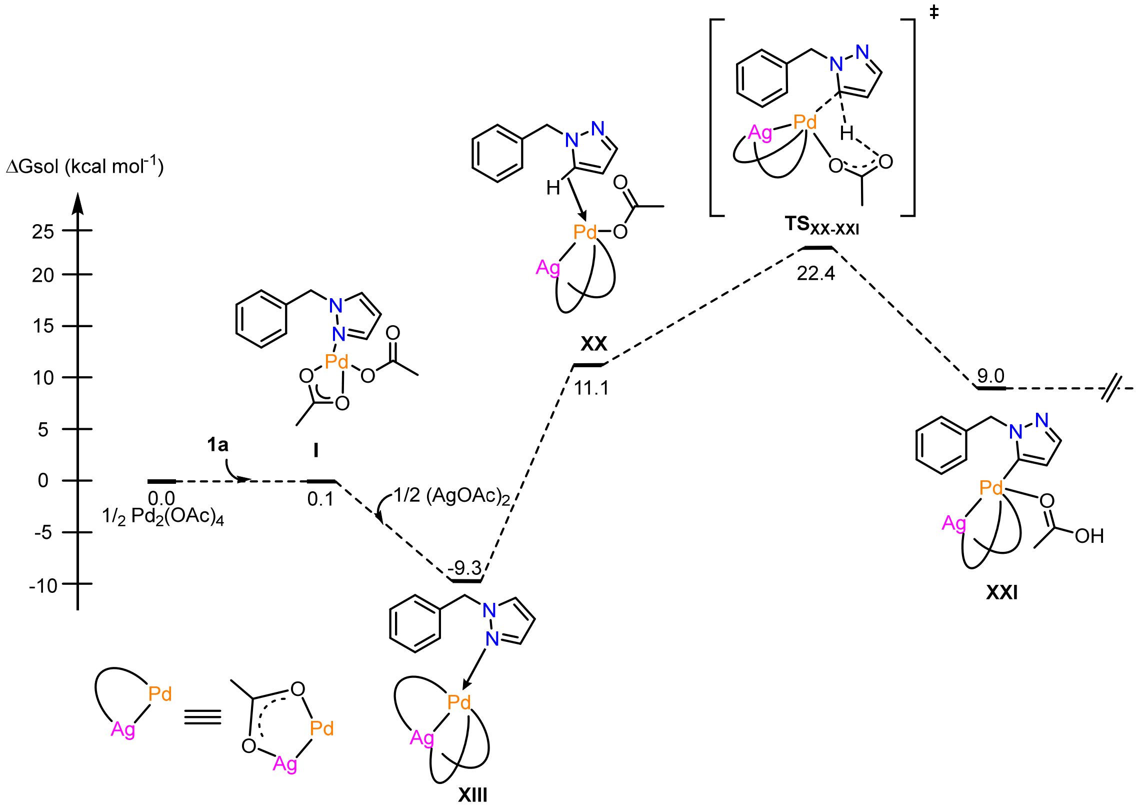

Figure S6. Free energy profile for the initial C-H activation in the pyrazole moiety catalyzed by Pd^{II}-Ag^{I} bimetallic species. Computational studies were performed at B3LYP-D3/6-311++G(d,p)-cc-pVTZ-pp$_{DMF(SMD)}$//B3LYP-D3/6-31G(d,p)-LANL2DZ$_{DMF(SMD)}$. Energies are relative to $\frac{1}{2}$ $Pd_2(OAc)_4$ combined with those of the relevant substrates.

15.DFT study for the 1,2-migratory insertion of 1,2-diphenylacetylene (2a) into C-Pd bond of dinuclear complex 4a

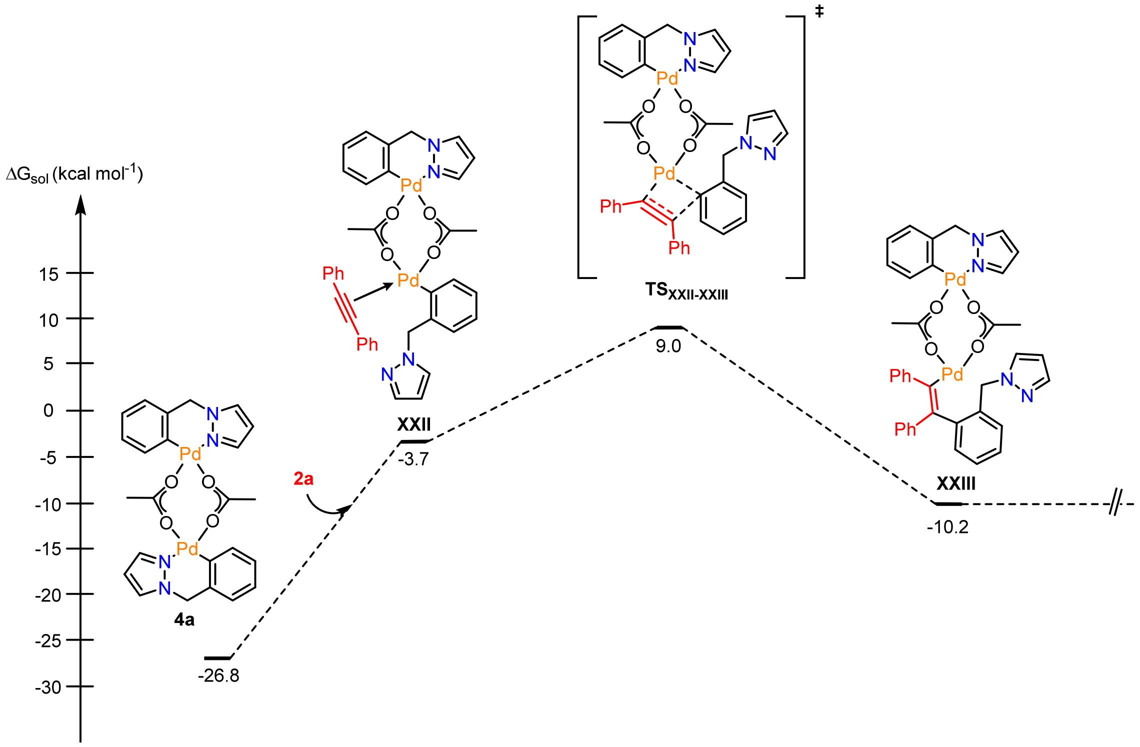

Figure S7. Free energy profile for the 1,2-migratory insertion of 1,2-diphenylacetylene (**2a**) into C-Pd bond of dinuclear complex **4a**. Computational studies were performed at B3LYP-D3/6-311++G(d,p)-cc-pVTZ-pp$_{DMF(SMD)}$//B3LYP-D3/6-31G(d,p)-LANL2DZ$_{DMF(SMD)}$. Energies are relative to $\frac{1}{2}$ $Pd_2(OAc)_4$ combined with those of the relevant substrates.

16.References

(1) Velasco-Rubio, Á.; Varela, J. A.; Saá, C. Palladium-Catalyzed [5 + 2] Heteroannulation of Phenethylamides with 1,3-Dienes to Dopaminergic 3-Benzazepines. *Org. Lett.* **2020**, *22*, 3591-3595.

(2) Zhang, P.; Huang, D.; Newhouse, T. R. Aryl-Nickel-Catalyzed Benzylic Dehydrogenation of Electron-Deficient Heteroarenes. *J. Am. Chem. Soc.* **2020**, *142*, 1757-1762.

(3) Zheng, H.-X.; Shan, X.-H.; Qu, J.-P.; Kang, Y.-B. Transition-Metal-Free Hydrogenation of Aryl Halides: From Alcohol to Aldehyde. *Org. Lett.* **2017**, *19*, 5114-5117.

(4) Desai, L. V.; Stowers, K. J.; Sanford, M. S. Insights into Directing Group Ability in Palladium-Catalyzed C−H Bond Functionalization. *J. Am. Chem. Soc.* **2008**, *130*, 13285-13293.

(5) Molander, G. A.; Ryu, D.; Hosseini-Sarvari, M.; Devulapally, R.; Seapy, D. G. Suzuki–Miyaura Cross-Coupling of Potassium Trifluoro(N-methylheteroaryl)borates with Aryl and Heteroaryl Halides. *J. Org. Chem.* **2013**, *78*, 6648-6656.

(6) Shi, J.; Yuan, T.; Wang, R.; Zheng, M.; Wang, X. Boron carbonitride photocatalysts for direct decarboxylation: the construction of C(sp3)–N or C(sp3)–C(sp2) bonds with visible light. *Green Chem.* **2021**, *23*, 3945-3949.

(7) Hamze, A.; Tréguier, B.; Brion, J.-D.; Alami, M. Copper-catalyzed reductive coupling of tosylhydrazones with amines: A convenient route to α-branched amines. *Org. Biomol. Chem.* **2011**, *9*, 6200-6204.

(8) Mio, M. J.; Kopel, L. C.; Braun, J. B.; Gadzikwa, T. L.; Hull, K. L.; Brisbois, R. G.; Markworth, C. J.; Grieco, P. A. One-Pot Synthesis of Symmetrical and Unsymmetrical Bisarylethynes by a Modification of the Sonogashira Coupling Reaction. *Org. Lett.* **2002**, *4*, 3199-3202.

(9) Kim, H. T.; Kang, E.; Kim, M.; Joo, J. M. Synthesis of Bidentate Nitrogen Ligands by Rh-Catalyzed C–H Annulation and Their Application to Pd-Catalyzed Aerobic C–H Alkenylation. *Org. Lett.* **2021**, *23*, 3657-3662.

(10) Li, J.-R.; Zhou, H.-C. Bridging-ligand-substitution strategy for the preparation of metal–organic polyhedra. *Nat. Chem.* **2010**, *2*, 893-898.

(11) Jing, T.; Liu, N.; Xu, C.; Bu, Q. Zinc Chloride-Promoted Coupling Reaction between Calcium Carbide and Aryl Chlorides. *Eur. J. Org. Chem.* **2022**, *2022*, e202200178.

(12) Bello-García, J.; Padín, D.; Varela, J. A.; Saá, C. Nonplanar Tub-Shaped Benzocyclooctatetraenes via Halogen-Radical Ring Opening of Dihydrobiphenylenes. *Org. Lett.* **2021**, *23*, 5539-5544.

(13) Peng, S.; Sun, Z.; Zhu, H.; Chen, N.; Sun, X.; Gong, X.; Wang, J.; Wang, L. Pd-Catalyzed Oxidative Annulation of Aryl Ethers with Alkynes: Synthesis of Functionalized Spirocycles and Naphthalenes. *Org. Lett.* **2020**, *22*, 3200-3204.

(14) Yamakawa, T.; Yoshikai, N. Annulation of α,β-Unsaturated Imines and Alkynes via Cobalt-Catalyzed Olefinic C–H Activation. *Org. Lett.* **2013**, *15*, 196-199.

(15) Schwärzer, K.; Rout, S. K.; Bessinger, D.; Lima, F.; Brocklehurst, C. E.; Karaghiosoff, K.; Bein, T.; Knochel, P. Selective functionalization of the 1H-imidazo[1,2-b]pyrazole scaffold. A new potential non-classical isostere of indole and a precursor of push–pull dyes. *Chem. Sci.* **2021**, *12*, 12993-13000.

(16) Frisch, M. J.; Trucks, G. W.; Schlegel, H. B.; Scuseria, G. E.; Robb, M. A.; Cheeseman, J. R.; Scalmani, G.; Barone, V.; Petersson, G. A.; Nakatsuji, H.; Li, X.;

Caricato, M.; Marenich, A. V.; Bloino, J.; Janesko, B. G.; Gomperts, R.; Mennucci, B.; Hratchian, H. P.; Ortiz, J. V.; Izmaylov, A. F.; Sonnenberg, J. L.; Williams-Young, D.; Ding, F.; Lipparini, F.; Egidi, F.; Goings, J.; Peng, B.; Petrone, A.; Henderson, T.; Ranasinghe, D.; Zakrzewski, V. G.; Gao, J.; Rega, N.; Zheng, G.; Liang, W.; Hada, M.; Ehara, M.; Toyota, K.; Fukuda, R.; Hasegawa, J.; Ishida, M.; Nakajima, T.; Honda, Y.; Kitao, O.; Nakai, H.; Vreven, T.; Throssell, K.; J. A. Montgomery, J.; Peralta, J. E.; Ogliaro, F.; Bearpark, M. J.; Heyd, J. J.; Brothers, E. N.; Kudin, K. N.; Staroverov, V. N.; Keith, T. A.; Kobayashi, R.; Normand, J.; Raghavachari, K.; Rendell, A. P.; Burant, J. C.; Iyengar, S. S.; Tomasi, J.; Cossi, M.; Millam, J. M.; Klene, M.; Adamo, C.; Cammi, R.; Ochterski, J. W.; Martin, R. L.; Morokuma, K.; Farkas, O.; Foresman, J. B.; Fox, D. J. *Gaussian 16, Revision B.01*, Gaussian, Inc., Wallingford CT, 2016.

(17) (a) Parr, R. G.; Wang, Y. Density Functional Theory of Atoms and Molecules. Oxford University Press: New York, 1989. (b) Miehlich, B.; Savin, A.; Stoll, H.; Preuss, H. Results obtained with the correlation energy density functionals of Becke and Lee, Yang and Parr. *Chem. Phys. Lett.* **1989**, *157*, 200-206. (c) Lee, C.; Yang, W.; Parr, R. G. Development of the Colle-Salvetti correlation-energy formula into a functional of the electron density. *Phys. Rev. B* **1988**, *37*, 785-789. (d) Becke, A. D. Density-functional thermochemistry. III. The role of exact exchange. *J. Chem. Phys.* **1993**, *98*, 5648-5652.

(18) Grimme, S.; Antony, J.; Ehrlich, S.; Krieg, H. A consistent and accurate ab initio parametrization of density functional dispersion correction (DFT-D) for the 94 elements H-Pu. *J. Chem. Phys.* **2010**, *132*, 154104.

(19) Marenich, A. V.; Cramer, C. J.; Truhlar, D. G. Universal Solvation Model Based on Solute Electron Density and on a Continuum Model of the Solvent Defined by the Bulk Dielectric Constant and Atomic Surface Tensions. *J. Phys. Chem. B* **2009**, *113*, 6378-6396.

(20) Hay, P. J.; Wadt, W. R. Ab initio effective core potentials for molecular calculations. Potentials for potassium to gold including the outermost core orbitals. *J. Chem. Phys.* **1985**, *82*, 299-310.

(21) Hay, P. J.; Wadt, W. R. Ab initio effective core potentials for molecular calculations. Potentials for the transition metal atoms scandium to mercury. *J. Chem. Phys.* **1985**, *82*, 270-283.

(22) (a) Hariharan, P. C.; Pople, J. A. Influence of polarization functions on MO hydrogenation energies. *Theor. Chim. Acta* **1973**, *28*, 213-222. (b) Ditchfield, R.; Hehre, W. J.; Pople, J. A. Self-consistent molecular-orbital methods. IX. Extended Gaussian-type basis for molecular-orbital studies of organic molecules. *J. Chem. Phys.* **1971**, *54*, 724-728. (c) Hehre, W. J.; Ditchfield, R.; Pople, J. A. Self-consistent molecular orbital methods. XII. Further extensions of Gaussian-type basis sets for use in molecular orbital studies of organic molecules. *J. Chem. Phys.* **1972**, *56*, 2257-2261. (d) Gordon, M. S.; Binkley, J. S.; Pople, J. A.; Pietro, W. J.; Hehre, W. J. Self-consistent molecular-orbital methods. 22. Small split-valence basis sets for second-row elements. *J. Am. Chem. Soc.* **1982**, *104*, 2797-2803. (e) Francl, M. M.; Pietro, W. J.; Hehre, W. J.; Binkley, J. S.; Gordon, M. S.; DeFrees, D. J.; Pople, J. A. Self-consistent molecular orbital methods. XXIII. A polarization-type basis set for second-row elements. *J. Chem. Phys.* **1982**, *77*, 3654-3665.

(23) Peterson, K. A.; Figgen, D.; Dolg, M.; Stoll, H. Energy-consistent relativistic pseudopotentials and correlation consistent basis sets for the 4d elements Y-Pd. *J. Chem. Phys.* **2007**, *126*, 124101.

(24) (a) Krishnan, R.; Binkley, J. S.; Seeger, R.; Pople, J. A. Self-consistent molecular orbital methods. XX. A basis set for correlated wave functions. *J. Chem. Phys.* **1980**, *72*, 650-654. (b) McLean, A. D.; Chandler, G. S. Contracted Gaussian basis sets for molecular

calculations. I. Second row atoms, Z = 11-18. *J. Chem. Phys.* **1980**, *72*, 5639-5648. (c) Clark, T.; Chandrasekhar, J.; Spitznagel, G. W.; Schleyer, P. v. R. Efficient diffuse function-augmented basis sets for anion calculations. III. The 3-21 + G basis set for first-row elements, lithium to fluorine. *J. Comput. Chem.* **1983**, *4*, 294-301. (d) Spitznagel, G. W.; Clark, T.; Schleyer, P.; Hehre, W. J. Efficient diffuse function-augmented basis sets for anion calculation. Part IV. An evaluation of the performance of diffuse function-augmented basis sets for second row elements, Na-Cl. *J. Comput. Chem.* **1987**, *8*, 1109-1116.

17. Cartesian coordinates in Å, energy values in Hartrees and imaginary frequencies in cm^{-1} for transition states (TS) of species involved throughout DFT study

17.1. Cartesian coordinates in Å, energy values in Hartrees and imaginary frequencies in cm^{-1} for transition states (TS) of common structures

1a

Electronic Energy BS1 = -496.611160456 Hartree

Electronic Energy BS2 = -496.726803309 Hartree

Zero-point Energy Correction = 0.180933 Hartree

Thermal Correction to Enthalpy = 0.191305 Hartree

Thermal Correction to Free Energy = 0.143766 Hartree

Chemical symbol X, Y, Z

C	0.384270	0.862937	1.121509
H	0.501112	0.515557	2.151212
H	0.401490	1.955077	1.126523
N	2.168785	-0.760692	0.747243
N	1.563568	0.399118	0.394119
C	2.100076	0.937736	-0.730125
H	1.718464	1.859983	-1.144284
C	3.123326	0.095842	-1.131070
H	3.780669	0.221157	-1.979226
C	3.118310	-0.942599	-0.176208
H	3.769443	-1.805495	-0.115087
C	-0.911488	0.352284	0.519911
C	-1.889045	1.247275	0.073078
C	-1.142043	-1.025898	0.403598
C	-3.083980	0.775452	-0.478227
H	-1.714759	2.317063	0.155896
C	-2.331406	-1.497985	-0.150898
H	-0.380768	-1.723059	0.742230
C	-3.306907	-0.597702	-0.592377
H	-3.835368	1.480836	-0.821906
H	-2.499765	-2.567853	-0.237152
H	-4.233658	-0.966306	-1.022885

2a

Electronic Energy BS1 = -539.506234338 Hartree

Electronic Energy BS2 = -539.627891799 Hartree

Zero-point Energy Correction = 0.191599 Hartree

Thermal Correction to Enthalpy = 0.203621 Hartree

Thermal Correction to Free Energy = 0.152525 Hartree

Chemical symbol X, Y, Z			
C	-0.608525	-0.000014	-0.000011
C	0.608526	0.000009	-0.000005
C	2.034029	0.000005	-0.000004
C	2.748443	-1.216090	-0.000113
C	2.748452	1.216095	0.000112
C	4.141229	-1.210023	-0.000110
H	2.202363	-2.154236	-0.000202
C	4.141237	1.210018	0.000116
H	2.202378	2.154244	0.000198
C	4.841781	-0.000005	0.000006
H	4.681937	-2.151924	-0.000198
H	4.681952	2.151916	0.000207
H	5.927814	-0.000009	0.000009
C	-2.034030	-0.000007	-0.000004
C	-2.748454	-1.216096	0.000110
C	-2.748441	1.216089	-0.000115
C	-4.141240	-1.210016	0.000117
H	-2.202382	-2.154246	0.000195
C	-4.141227	1.210025	-0.000110
H	-2.202358	2.154233	-0.000204
C	-4.841781	0.000008	0.000007
H	-4.681957	-2.151912	0.000208
H	-4.681933	2.151927	-0.000196
H	-5.927814	0.000014	0.000012

Pd_2OAc_4

Electronic Energy BS1 = -1167.58541374 Hartree

Electronic Energy BS2 = -1169.46429935 Hartree

Zero-point Energy Correction = 0.208410 Hartree

Thermal Correction to Enthalpy = 0.231755 Hartree

Thermal Correction to Free Energy = 0.152827 Hartree

Chemical symbol X, Y, Z			
Pd	-0.000004	-0.000030	1.308479
C	-1.858684	1.851630	0.000022
O	-1.456716	1.456653	1.144161
O	-1.456699	1.456725	-1.144139
C	-2.962417	2.879838	0.000043
H	-2.908514	3.498530	-0.897284
H	-3.921416	2.349165	-0.000289
H	-2.908884	3.498121	0.897672
C	1.858681	-1.851629	-0.000005
O	1.456712	-1.456698	1.144153
O	1.456695	-1.456678	-1.144147
C	2.962437	-2.879812	-0.000035
H	2.908633	-3.498371	-0.897460
H	3.921417	-2.349103	-0.000214
H	2.908855	-3.498223	0.897503
Pd	-0.000001	0.000029	-1.308480

C	-1.858693	-1.851621	-0.000029
O	-1.456712	-1.456713	1.144132
O	-1.456727	-1.456639	-1.144168
C	-2.962421	-2.879835	-0.000042
H	-3.921423	-2.349168	0.000559
H	-2.909042	-3.497969	-0.897781
H	-2.908355	-3.498674	0.897175
C	1.858694	1.851614	0.000019
O	1.456709	1.456656	1.144159
O	1.456725	1.456692	-1.144142
C	2.962434	2.879816	0.000033
H	3.921429	2.349137	-0.000405
H	2.908470	3.498564	-0.897252
H	2.908968	3.498043	0.897704

$(AgOAc)_2$

Electronic Energy BS1 = -748.644806337 Hartree

Electronic Energy BS2 = -751.224073979 Hartree

Zero-point Energy Correction = 0.102536 Hartree

Thermal Correction to Enthalpy = 0.117115 Hartree

Thermal Correction to Free Energy = 0.057899 Hartree

Chemical symbol X, Y, Z

C	-2.730850	0.002555	0.000019
O	-2.169123	1.139997	0.000036
O	-2.164863	-1.134426	0.000001
C	-4.250118	-0.017900	-0.000108
H	-4.603439	-0.560425	-0.882719
H	-4.603673	-0.562283	0.881249
H	-4.664029	0.991149	0.000845
Ag	-0.000000	-1.414882	0.000023
Ag	0.000000	1.422502	0.000012
O	2.164863	-1.134427	0.000008
C	2.730850	0.002555	0.000031
O	2.169123	1.139996	0.000046
C	4.250118	-0.017900	-0.000116
H	4.603693	-0.562455	0.881127
H	4.603421	-0.560251	-0.882842
H	4.664028	0.991149	0.001017

AcOH

Electronic Energy BS1 = -229.103532941 Hartree

Electronic Energy BS2 = -229.178130547 Hartree

Zero-point Energy Correction = 0.061726 Hartree

Thermal Correction to Enthalpy = 0.067194 Hartree

Thermal Correction to Free Energy = 0.034697 Hartree

Chemical symbol X, Y, Z

H	1.724922	-0.816116	0.000062
O	0.644214	1.203846	-0.000000
C	0.090990	0.122564	-0.000013
O	0.776778	-1.043772	-0.000007
C	-1.394091	-0.110259	0.000002
H	-1.918067	0.845175	-0.000052
H	-1.678122	-0.691816	-0.882689
H	-1.678058	-0.691671	0.882807

4a

Electronic Energy BS1 = -1702.63872071 Hartree

Electronic Energy BS2 = -1704.61476817 Hartree

Zero-point Energy Correction = 0.448141 Hartree

Thermal Correction to Enthalpy = 0.481780 Hartree

Thermal Correction to Free Energy = 0.381420 Hartree

Chemical symbol X, Y, Z

Pd	0.942386	-1.065240	0.969457
Pd	-0.550783	-0.509232	-1.511461
N	-0.486934	-0.514366	2.324013
N	-0.818269	0.769334	2.574555
C	-1.814873	0.832055	3.484077
H	-2.219701	1.784452	3.792718
C	-2.133981	-0.470133	3.846290
H	-2.880173	-0.785592	4.559916
C	-1.274064	-1.280863	3.091412
H	-1.180209	-2.355585	3.046358
C	-0.218142	1.854828	1.796586
H	-0.559632	2.786090	2.249787
H	-0.629969	1.795037	0.783692
C	1.292759	1.783604	1.758939
C	1.960980	0.595488	1.405911
C	3.360646	0.605640	1.325561
H	3.888237	-0.297456	1.035316
C	4.086334	1.776688	1.570802
H	5.171070	1.764424	1.495591
C	3.419044	2.956881	1.904002
H	3.975186	3.870928	2.092729
C	2.026376	2.953107	2.001368
H	1.498491	3.868218	2.260462
N	0.382343	1.275899	-1.920333
N	-0.276086	2.405046	-2.260807
C	0.571257	3.455080	-2.310868
H	0.214620	4.438368	-2.580638
C	1.836654	2.985250	-1.983332
H	2.749874	3.556712	-1.914698
C	1.668806	1.613572	-1.747174
H	2.383795	0.871394	-1.430171
C	-1.702624	2.345570	-2.579830
H	-1.811193	1.741739	-3.487869

H	-2.015952	3.365313	-2.805535
C	-2.503161	1.757692	-1.437335
C	-2.169215	0.499266	-0.898540
C	-2.948725	-0.005980	0.152303
H	-2.707499	-0.966807	0.590820
C	-4.029794	0.720848	0.663253
H	-4.610142	0.311008	1.485897
C	-4.352472	1.968625	0.127449
H	-5.187460	2.540335	0.522800
C	-3.586670	2.481385	-0.921190
H	-3.828088	3.453433	-1.345616
O	-0.167380	-2.936716	0.599304
C	-1.091625	-3.130283	-0.235938
O	-1.496984	-2.321416	-1.136646
C	-1.841353	-4.445666	-0.144704
H	-1.144729	-5.262431	0.059825
H	-2.404256	-4.649160	-1.057088
H	-2.542386	-4.385746	0.696046
O	1.224834	-1.601189	-2.230451
C	2.254436	-1.896991	-1.568696
O	2.451355	-1.683188	-0.324276
C	3.383796	-2.598946	-2.297473
H	3.400806	-2.307400	-3.349608
H	3.212084	-3.680481	-2.244023
H	4.345201	-2.382681	-1.827203

I

Electronic Energy BS1 = -1080.40368600 Hartree

Electronic Energy BS2 = -1081.46950771 Hartree

Zero-point Energy Correction = 0.285965 Hartree

Thermal Correction to Enthalpy = 0.308716 Hartree

Thermal Correction to Free Energy = 0.230938 Hartree

Chemical symbol X, Y, Z

Pd	0.389573	0.834637	-0.184014
N	0.979497	-0.942502	-1.016121
N	0.117342	-1.861552	-1.521367
C	0.797858	-2.937561	-1.974582
H	0.270906	-3.772841	-2.412134
C	2.148533	-2.707427	-1.760214
H	2.970682	-3.361112	-2.009467
C	2.213983	-1.444877	-1.155916
H	3.061872	-0.866747	-0.819426
C	-1.332528	-1.649622	-1.511257
H	-1.758059	-2.504412	-2.043141
H	-1.554285	-0.747684	-2.085936
C	-1.895030	-1.541592	-0.107538
C	-1.501075	-2.440394	0.892051
C	-2.021655	-2.330399	2.182066
H	-1.706158	-3.028733	2.952190

C	-2.946377	-1.325146	2.483417
H	-3.349368	-1.239348	3.488596
C	-3.346569	-0.430417	1.488285
H	-4.060447	0.356317	1.715687
C	-2.819107	-0.535457	0.198631
H	-3.109285	0.176573	-0.568528
O	1.458975	0.407096	1.470015
C	2.742764	0.653543	1.428898
O	3.354451	1.100940	0.455068
C	3.451176	0.284970	2.722725
H	4.425554	0.775212	2.768294
H	3.600846	-0.800676	2.745165
O	-0.952901	1.649751	-1.589523
C	-1.150116	2.655126	-0.816933
O	-0.494327	2.692109	0.280727
C	-2.087557	3.751768	-1.200645
H	-2.856141	3.373231	-1.877551
H	-2.542125	4.189069	-0.308981
H	-1.520238	4.534146	-1.718366
H	2.850470	0.556387	3.594409
H	-0.777859	-3.218570	0.662006

17.2. Cartesian coordinates in Å, energy values in Hartrees and imaginary frequencies in cm^{-1} for transition states (TS) of species in Figure S3

II

Electronic Energy BS1 = -1080.39268381 Hartree

Electronic Energy BS2 = -1081.45929736 Hartree

Zero-point Energy Correction = 0.285724 Hartree

Thermal Correction to Enthalpy = 0.308558 Hartree

Thermal Correction to Free Energy = 0.231052 Hartree

Chemical symbol X, Y, Z			
Pd	-0.375370	-0.026473	-0.188121
N	1.169436	-1.403029	-0.020397
N	2.288915	-1.227463	0.724954
C	3.100379	-2.302056	0.627036
H	4.042106	-2.329747	1.155346
C	2.482401	-3.215693	-0.214728
H	2.854978	-4.183668	-0.514357
C	1.277883	-2.608265	-0.596017
H	0.488008	-2.961493	-1.241198
C	2.479416	0.013902	1.476594
H	3.520031	0.026636	1.804582
H	1.827352	0.000440	2.354212

C	2.159500	1.188118	0.580669
C	0.929797	1.864246	0.724112
C	0.603488	2.913241	-0.161564
H	-0.333036	3.442539	-0.028893
C	1.482539	3.270654	-1.178197
H	1.231877	4.079189	-1.857935
C	2.700160	2.590620	-1.315432
H	3.388581	2.871566	-2.107261
C	3.035119	1.551332	-0.448258
H	3.973375	1.017045	-0.568365
O	-2.083245	-2.162461	1.117752
C	-2.127688	-2.278726	-0.104758
O	-1.498320	-1.528456	-0.976630
C	-2.930332	-3.382768	-0.783157
H	-2.241587	-4.131241	-1.190512
H	-3.515062	-2.983682	-1.616405
H	-3.590729	-3.862224	-0.058270
H	0.291507	1.682394	1.587919
O	-1.965385	1.175057	-0.501070
C	-2.479248	1.817264	0.513232
O	-2.032989	1.833873	1.663526
C	-3.732780	2.592261	0.130226
H	-4.500063	1.901866	-0.234899
H	-4.114764	3.141651	0.992533
H	-3.508295	3.291057	-0.682023

TS_{II-III}

Imaginary Freq = -1204.7951 (cm^{-1})

Electronic Energy BS1 = -1080.37468532 Hartree

Electronic Energy BS2 = -1081.44090235 Hartree

Zero-point Energy Correction = 0.280991 Hartree

Thermal Correction to Enthalpy = 0.302935 Hartree

Thermal Correction to Free Energy = 0.228846 Hartree

Chemical symbol X, Y, Z

Pd	-0.432527	0.301399	-0.314892
N	-0.302066	-1.731472	-0.115612
N	0.559572	-2.342982	0.730313
C	0.382862	-3.681248	0.697088
H	0.989262	-4.330901	1.311052
C	-0.639532	-3.946864	-0.203785
H	-1.037426	-4.913722	-0.472948
C	-1.039268	-2.693033	-0.686819
H	-1.812294	-2.420820	-1.389531
C	1.509148	-1.549565	1.519997
H	2.131719	-2.259447	2.065431
H	0.936939	-0.950618	2.235855
C	2.320666	-0.669225	0.598162
C	1.703644	0.462983	0.017364
C	2.431066	1.201603	-0.940640

H	1.977180	2.083377	-1.385703
C	3.731222	0.843280	-1.294623
H	4.278820	1.426904	-2.029106
C	4.329489	-0.266838	-0.691852
H	5.344711	-0.548399	-0.956936
C	3.624363	-1.023334	0.249524
H	4.087469	-1.895647	0.703037
O	-3.035788	0.018691	1.220823
C	-3.263352	-0.068562	0.011446
O	-2.379927	0.033659	-0.943439
C	-4.667243	-0.351463	-0.512543
H	-4.716247	-1.388162	-0.865229
H	-4.905245	0.295265	-1.361823
H	-5.404193	-0.212040	0.280956
H	1.072781	1.340863	0.809442
O	-0.671859	2.355450	-0.407167
C	-0.106894	3.013973	0.525822
O	0.713999	2.505675	1.354937
C	-0.400577	4.488432	0.624735
H	-1.285157	4.752134	0.044510
H	-0.533140	4.770286	1.672025
H	0.463572	5.039103	0.236598

III

Electronic Energy BS1 = -1080.42133889 Hartree

Electronic Energy BS2 = -1081.48347447 Hartree

Zero-point Energy Correction = 0.285861 Hartree

Thermal Correction to Enthalpy = 0.308113 Hartree

Thermal Correction to Free Energy = 0.232876 Hartree

Chemical symbol X, Y, Z

Pd	-0.247291	0.201681	-0.420834
N	0.114010	-1.815772	-0.273598
N	1.100820	-2.340689	0.483349
C	1.131122	-3.685056	0.359100
H	1.853993	-4.272680	0.905848
C	0.122819	-4.046324	-0.524584
H	-0.129005	-5.042304	-0.856614
C	-0.487953	-2.840250	-0.895567
H	-1.316359	-2.649466	-1.561199
C	1.881465	-1.475566	1.370754
H	2.649536	-2.103106	1.824317
H	1.208966	-1.128266	2.163613
C	2.490840	-0.301953	0.633871
C	1.695625	0.557957	-0.146836
C	2.295681	1.655004	-0.778487
H	1.689182	2.336039	-1.366500

C	3.667388	1.896684	-0.639613
H	4.115856	2.752856	-1.137583
C	4.454520	1.043817	0.135452
H	5.519813	1.225799	0.246478
C	3.863279	-0.051519	0.767796
H	4.469408	-0.720283	1.374601
O	-3.118205	0.100547	1.265875
C	-3.163808	-0.564443	0.142650
O	-2.390426	-0.370431	-0.812882
C	-4.206852	-1.643263	0.086128
H	-3.786644	-2.542210	0.553443
H	-4.450301	-1.876445	-0.951112
H	-5.102735	-1.357448	0.640568
H	-2.482245	0.920875	1.218314
O	-0.591894	2.230171	-0.610157
C	-1.270493	2.829708	0.296865
O	-1.858647	2.279139	1.261304
C	-1.377258	4.333721	0.143353
H	-0.516218	4.739839	-0.390974
H	-2.280944	4.561577	-0.434385
H	-1.475596	4.806630	1.122887

X

Electronic Energy BS1 = -1390.79961465 Hartree

Electronic Energy BS2 = -1391.92374778 Hartree

Zero-point Energy Correction = 0.415374 Hartree

Thermal Correction to Enthalpy = 0.445082 Hartree

Thermal Correction to Free Energy = 0.351104 Hartree

Chemical symbol X, Y, Z

C	0.019919	1.497006	-2.235196
C	0.823700	0.639804	-1.470958
C	2.207104	0.601561	-1.732082
C	2.759419	1.424990	-2.722162
C	1.951077	2.287743	-3.465016
C	0.577031	2.316134	-3.224446
H	-1.051794	1.539840	-2.065861
H	3.830037	1.387039	-2.910444
H	2.390705	2.922972	-4.228889
H	-0.066652	2.974118	-3.803152
C	3.118224	-0.317259	-0.944883
H	3.152749	-0.024575	0.110957
H	4.135067	-0.305851	-1.338116
N	1.408070	-2.018154	-0.609931
Pd	0.024300	-0.573441	-0.067343
C	-0.790505	1.263433	0.940006
C	-1.755375	0.741456	0.363162
N	2.662143	-1.709491	-0.998947
C	3.357921	-2.830563	-1.286684
H	4.389644	-2.773982	-1.601596
C	2.510254	-3.912216	-1.082575

H	2.739874	-4.957360	-1.226779
C	1.297707	-3.351664	-0.654734
H	0.365738	-3.816593	-0.369078
C	-3.026441	0.403351	-0.210424
C	-3.554615	-0.898168	-0.100941
C	-3.749288	1.399581	-0.896810
C	-4.791716	-1.188699	-0.671266
H	-2.979710	-1.653364	0.425625
C	-4.986255	1.093341	-1.461192
H	-3.335435	2.399688	-0.979579
C	-5.509238	-0.198600	-1.351487
H	-5.198537	-2.192241	-0.585591
H	-5.540772	1.863963	-1.988667
H	-6.472512	-0.433465	-1.795091
C	0.128894	2.124603	1.626748
C	1.080418	1.602058	2.524274
C	0.081749	3.509659	1.374692
C	1.967841	2.466426	3.162110
H	1.103502	0.528983	2.699294
C	0.975614	4.360576	2.021619
H	-0.647629	3.901785	0.672724
C	1.919788	3.842451	2.913808
H	2.702100	2.065234	3.855004
H	0.938461	5.428158	1.825342
H	2.617476	4.509417	3.412165
O	-0.886444	-2.025443	1.237982
C	-0.302353	-2.250606	2.369952
C	-0.942841	-3.377000	3.177601
H	-2.008765	-3.173790	3.325395
H	-0.867582	-4.317614	2.620678
H	-0.453499	-3.490658	4.147198
O	0.691450	-1.650346	2.809476

$TS_{X\text{-}XI}$

Imaginary Freq = -229.4658 (cm^{-1})

Electronic Energy BS1 = -1390.77442133 Hartree

Electronic Energy BS2 = -1391.89641977 Hartree

Zero-point Energy Correction = 0.414959 Hartree

Thermal Correction to Enthalpy = 0.443575 Hartree

Thermal Correction to Free Energy = 0.353587 Hartree

Chemical symbol X, Y, Z

C	-1.076674	1.143652	2.353319
C	-1.291694	0.631956	1.064029
C	-2.580457	0.703548	0.490319
C	-3.618699	1.295533	1.212161
C	-3.396070	1.815539	2.491643
C	-2.128480	1.724620	3.068486
H	-0.079197	1.121748	2.779574
H	-4.612514	1.338606	0.773822

H	-4.211504	2.285702	3.033321
H	-1.947939	2.123198	4.062726
C	-2.858776	0.047047	-0.842092
H	-2.078774	0.292953	-1.570116
H	-3.826382	0.348323	-1.244191
N	-1.849585	-2.062979	-0.137976
Pd	-0.090665	-0.920259	0.379555
C	0.392357	1.267740	0.046297
C	1.371657	0.493557	0.377368
N	-2.898658	-1.414969	-0.691547
C	-3.856725	-2.287692	-1.077035
H	-4.768069	-1.939756	-1.541133
C	-3.405756	-3.562121	-0.761416
H	-3.921682	-4.496275	-0.927101
C	-2.142431	-3.366459	-0.177753
H	-1.428578	-4.081823	0.204795
C	2.809794	0.488159	0.545935
C	3.512277	-0.699845	0.820293
C	3.525854	1.699283	0.429461
C	4.899506	-0.676267	0.963516
H	2.956663	-1.627478	0.906602
C	4.910213	1.712523	0.574909
H	2.986769	2.618389	0.219352
C	5.602716	0.525428	0.841884
H	5.432842	-1.599584	1.172441
H	5.451575	2.649837	0.481033
H	6.682972	0.539784	0.955689
C	0.009595	2.418031	-0.757362
C	0.643170	2.577092	-2.006689
C	-0.908565	3.390866	-0.323208
C	0.366158	3.690285	-2.798256
H	1.346628	1.821870	-2.343296
C	-1.180753	4.499619	-1.122116
H	-1.394767	3.282497	0.639304
C	-0.548312	4.652430	-2.360022
H	0.859018	3.801903	-3.759721
H	-1.887354	5.248334	-0.775877
H	-0.768492	5.516578	-2.980228
O	1.063656	-2.697657	0.206716
C	1.452652	-2.997509	-0.995621
C	2.283187	-4.277333	-1.063863
H	3.293183	-4.066359	-0.692096
H	1.856124	-5.057686	-0.427530
H	2.358529	-4.634683	-2.093295
O	1.235691	-2.335670	-2.018225

XI

Electronic Energy BS1 = -1390.80305243 Hartree

Electronic Energy BS2 = -1391.91947821 Hartree

Zero-point Energy Correction = 0.416616 Hartree

Thermal Correction to Enthalpy = 0.445526 Hartree

Thermal Correction to Free Energy = 0.354295 Hartree

Chemical symbol X, Y, Z			
C	-0.086006	1.624741	2.380627
C	-0.207316	1.516216	0.967649
C	-1.348737	2.108123	0.350048
C	-2.265550	2.817390	1.130253
C	-2.100947	2.940139	2.509744
C	-1.021776	2.316625	3.141888
H	0.790043	1.191266	2.851630
H	-3.140487	3.246889	0.649534
H	-2.824484	3.503211	3.091860
H	-0.897260	2.389801	4.217889
C	-1.726731	1.817536	-1.087508
H	-0.890958	1.394744	-1.650332
H	-2.077938	2.715929	-1.598728
N	-2.840558	-0.226895	-0.283059
Pd	-0.807613	-0.668062	0.584881
C	1.020700	0.943583	0.254035
C	1.164305	-0.382661	0.496879
N	-2.850037	0.862684	-1.096907
C	-3.954460	0.877722	-1.878296
H	-4.125425	1.681255	-2.579996
C	-4.697852	-0.250279	-1.565034
H	-5.644473	-0.547750	-1.991514
C	-3.955512	-0.905492	-0.566335
H	-4.164722	-1.829936	-0.045838
C	2.314766	-1.269151	0.501651
C	2.184781	-2.662732	0.333719
C	3.607643	-0.739959	0.730748
C	3.308415	-3.488171	0.345378
H	1.193594	-3.083448	0.226084
C	4.724374	-1.569058	0.739803
H	3.724157	0.326045	0.895635
C	4.582278	-2.947647	0.539728
H	3.187130	-4.559383	0.209084
H	5.708704	-1.141887	0.911026
H	5.455715	-3.593515	0.550277
C	1.845227	1.853883	-0.566215
C	2.610302	1.365808	-1.643878
C	1.852022	3.237037	-0.309332
C	3.374320	2.232035	-2.421519
H	2.592445	0.304692	-1.870738
C	2.618052	4.102458	-1.091568
H	1.263314	3.633995	0.512404
C	3.383600	3.604957	-2.148825
H	3.954941	1.837804	-3.251028
H	2.616113	5.166865	-0.873365
H	3.976095	4.279517	-2.760322
O	-1.024160	-2.744380	0.473208
C	-1.165488	-3.218457	-0.733296
C	-1.500817	-4.708174	-0.745160
H	-0.611396	-5.281037	-0.458085
H	-2.286022	-4.936493	-0.018735
H	-1.814592	-5.020420	-1.743388

O	-1.020551	-2.592700	-1.786861

VI

Electronic Energy BS1 = -1619.95034019 Hartree

Electronic Energy BS2 = -1621.13344293 Hartree

Zero-point Energy Correction = 0.479652 Hartree

Thermal Correction to Enthalpy = 0.513816 Hartree

Thermal Correction to Free Energy = 0.410687 Hartree

Chemical symbol X, Y, Z

C	0.171393	-1.710598	-2.197367
C	0.753224	-1.725692	-0.922538
C	0.702413	-2.914368	-0.163278
C	0.090550	-4.051408	-0.695943
C	-0.465885	-4.031538	-1.978307
C	-0.433177	-2.854913	-2.726615
H	0.197800	-0.790401	-2.772918
H	0.037495	-4.955876	-0.095495
H	-0.935073	-4.925118	-2.379705
H	-0.875255	-2.822863	-3.718460
C	1.206609	-2.917568	1.262510
H	2.265943	-2.657458	1.322904
H	1.063115	-3.898581	1.724187
C	1.439822	-0.487277	-0.419273
C	0.722998	0.557158	0.049453
C	1.170499	1.950818	0.200937
C	0.860286	2.733224	1.331372
C	1.869988	2.565456	-0.860624
C	1.263371	4.066524	1.410665
H	0.303876	2.290779	2.148972
C	2.269626	3.896889	-0.779506
H	2.097712	1.981375	-1.746632
C	1.970820	4.654113	0.358613
H	1.021610	4.647797	2.296303
H	2.809992	4.347769	-1.607467
H	2.280258	5.693612	0.420435
C	2.930441	-0.499789	-0.485037
C	3.705643	0.056500	0.549873
C	3.597253	-1.106578	-1.564986
C	5.098261	0.034509	0.490350
H	3.205402	0.490738	1.408760
C	4.991657	-1.126552	-1.623905
H	3.019039	-1.555504	-2.366914
C	5.749000	-0.553523	-0.598982
H	5.677012	0.467019	1.302155
H	5.486229	-1.591849	-2.472390
H	6.834355	-0.573095	-0.643231
O	-1.559186	1.428896	-1.473050
C	-2.593002	2.071683	-1.749794
Pd	-1.219532	0.329707	0.388786
C	-0.810243	-1.557135	1.920470

H	-1.540517	-2.174492	1.414087
C	0.302133	-0.130539	3.233021
O	-3.574424	-1.994287	0.605639
C	-4.075151	-0.874616	0.503593
O	-3.412605	0.251577	0.662692
C	-5.546735	-0.684724	0.177631
H	-5.643115	-0.163558	-0.781648
H	-6.022500	-0.056595	0.937491
H	-6.057458	-1.647120	0.118880
N	0.496576	-1.897510	2.044647
N	1.184944	-1.030338	2.831697
C	-0.988483	-0.376058	2.668973
H	0.600034	0.667754	3.899100
H	-1.928650	0.065103	2.968522
O	-3.697150	2.033555	-1.056810
H	-3.646520	1.320794	-0.291674
C	-2.625630	2.978980	-2.944541
H	-3.469737	2.711305	-3.586904
H	-2.781553	4.008393	-2.604754
H	-1.690837	2.912715	-3.500497

$TS_{VI\text{-}VII}$

Imaginary Freq = -1380.2299 (cm^{-1})

Electronic Energy BS1 = -1619.92316122 Hartree

Electronic Energy BS2 = -1621.10628725 Hartree

Zero-point Energy Correction = 0.475235 Hartree

Thermal Correction to Enthalpy = 0.509147 Hartree

Thermal Correction to Free Energy = 0.406139 Hartree

Chemical symbol X, Y, Z

C	-0.234577	-1.753725	-2.241617
C	0.306398	-1.724427	-0.949093
C	-0.175854	-2.628551	0.019160
C	-1.193277	-3.525858	-0.319384
C	-1.726170	-3.549475	-1.610389
C	-1.242338	-2.662746	-2.574280
H	0.127011	-1.045616	-2.981600
H	-1.570494	-4.210381	0.435925
H	-2.513907	-4.254483	-1.859864
H	-1.650673	-2.672249	-3.581113
C	0.395738	-2.619881	1.414060
H	1.487383	-2.554754	1.381307
H	0.129805	-3.544270	1.934279
C	1.337800	-0.701546	-0.562078
C	0.894193	0.431498	0.018094
C	1.669554	1.629023	0.379466
C	1.739060	2.084683	1.710089
C	2.338879	2.361284	-0.619943
C	2.475976	3.223730	2.032111
H	1.219384	1.531152	2.485973
C	3.068517	3.504853	-0.294855

H	2.285067	2.019654	-1.649010
C	3.142285	3.939865	1.031887
H	2.529633	3.554776	3.065824
H	3.580650	4.057060	-1.078354
H	3.710899	4.830542	1.284191
C	2.771515	-1.048430	-0.780578
C	3.755538	-0.707898	0.167965
C	3.173679	-1.768417	-1.920841
C	5.093044	-1.047034	-0.032594
H	3.464312	-0.185378	1.072675
C	4.513304	-2.105246	-2.121730
H	2.433664	-2.063228	-2.658047
C	5.481019	-1.743099	-1.181453
H	5.832371	-0.776152	0.716469
H	4.799638	-2.653947	-3.015088
H	6.523120	-2.008290	-1.336413
O	-1.062846	1.946591	-1.565966
C	-2.016072	2.344379	-2.253094
Pd	-1.073304	0.649958	0.220874
C	-1.011726	-0.518938	1.990822
H	-2.231634	-0.937336	1.509041
C	-0.188160	-0.401359	4.085209
O	-3.422323	-1.236829	1.231002
C	-3.950171	-0.294774	0.557619
O	-3.315738	0.763003	0.235212
C	-5.377679	-0.440451	0.107534
H	-5.382561	-0.582566	-0.979260
H	-5.928326	0.478727	0.324774
H	-5.858484	-1.294182	0.585469
N	-0.067494	-1.499772	2.256198
N	0.422095	-1.447145	3.496151
C	-1.085736	0.204666	3.204029
H	0.050689	-0.148300	5.110390
H	-1.734114	1.049201	3.392806
O	-3.280824	2.114889	-1.974547
H	-3.383205	1.588190	-1.123121
C	-1.801608	3.151013	-3.498164
H	-2.264954	2.639041	-4.347314
H	-2.293889	4.122682	-3.389492
H	-0.736549	3.290409	-3.680418

VII

Electronic Energy BS1 = -1619.94217802 Hartree

Electronic Energy BS2 = -1621.12550781 Hartree

Zero-point Energy Correction = 0.481093 Hartree

Thermal Correction to Enthalpy = 0.515285 Hartree

Thermal Correction to Free Energy = 0.412937 Hartree

Chemical symbol X, Y, Z

C	-0.309642	-2.687044	0.194818
C	0.140148	-1.555117	0.891582

C	-0.599782	-1.091063	1.998776	C	4.926730	-0.708362	1.659155
C	-1.784080	-1.742926	2.360928	H	3.434473	0.770301	1.211010
C	-2.233655	-2.858014	1.650231	C	4.267260	-3.007540	1.348136
C	-1.488837	-3.334382	0.567746	H	2.261771	-3.331141	0.653359
H	0.248383	-3.031997	-0.670678	C	5.236596	-2.070689	1.714252
H	-2.359542	-1.367154	3.203435	H	5.664550	0.031131	1.958949
H	-3.154429	-3.354590	1.943038	H	4.491255	-4.070039	1.393991
H	-1.830392	-4.200082	0.007104	H	6.217678	-2.397383	2.047521
C	-0.154173	0.140629	2.751640	O	-0.712955	-0.633734	-2.541582
H	0.936308	0.225498	2.737286	C	-1.483669	-1.539509	-2.880089
H	-0.476171	0.084900	3.792814	Pd	-0.854126	0.673110	-0.719918
C	1.330331	-0.770833	0.410156	C	-0.935853	1.784217	0.939171
C	1.072336	0.333830	-0.324214	H	-3.123427	1.899186	0.934147
C	2.047738	1.206726	-1.000197	C	-1.471484	3.386592	2.425239
C	2.089971	2.592004	-0.751489	O	-3.939933	1.335076	0.997778
C	2.952732	0.664425	-1.933339	C	-4.009894	0.506497	-0.017422
C	3.026930	3.401314	-1.393063	O	-3.196933	0.533670	-0.962058
H	1.385240	3.022592	-0.046424	C	-5.112865	-0.498387	0.056381
C	3.880445	1.478991	-2.582958	H	-4.672156	-1.437426	0.414197
H	2.924670	-0.402000	-2.136522	H	-5.526564	-0.671929	-0.939002
C	3.925390	2.849878	-2.312801	H	-5.893896	-0.186721	0.750121
H	3.053519	4.466387	-1.178408	N	-0.728581	1.392346	2.231343
H	4.570090	1.042226	-3.300475	N	-1.041950	2.354680	3.148665
H	4.648956	3.483639	-2.817850	C	-1.435733	3.095106	1.041947
C	2.685128	-1.220410	0.842777	H	-1.798551	4.293710	2.919605
C	3.667225	-0.288856	1.233136	H	-1.703409	3.744782	0.219492
C	3.004703	-2.588401	0.924978	O	-2.750654	-1.600006	-2.511330

H	-2.983252	-0.813883	-1.946834
C	-1.056141	-2.675780	-3.757187
H	-1.208635	-3.617772	-3.220289
H	-1.679462	-2.703763	-4.656235
H	-0.006795	-2.568115	-4.029404

$TS_{VII\text{-}XII}$

Imaginary Freq = -335.9126 (cm^{-1})

Electronic Energy BS1 = -1619.91535348 Hartree

Electronic Energy BS2 = -1621.09366461 Hartree

Zero-point Energy Correction = 0.479773 Hartree

Thermal Correction to Enthalpy = 0.513718 Hartree

Thermal Correction to Free Energy = 0.411856 Hartree

Chemical symbol X, Y, Z

C	-0.561684	2.536293	-0.443826
C	-0.039326	1.393072	-1.072075
C	-0.752194	0.825848	-2.147542
C	-1.968859	1.382194	-2.555073
C	-2.478471	2.517841	-1.922145
C	-1.768441	3.095655	-0.866048
H	-0.042931	2.958182	0.411237
H	-2.514655	0.922554	-3.375229
H	-3.421358	2.946591	-2.249314
H	-2.161214	3.972600	-0.359371
C	-0.203592	-0.414286	-2.802124
H	0.872221	-0.290982	-2.985002
H	-0.695745	-0.612858	-3.755162
C	1.209568	0.715597	-0.576031
C	1.046592	-0.463709	0.112880
C	2.122012	-1.107281	0.914086
C	2.693795	-2.351927	0.599651
C	2.597020	-0.423482	2.050463
C	3.704301	-2.893984	1.396498
H	2.359788	-2.885108	-0.282873
C	3.604846	-0.966828	2.845891
H	2.165848	0.541410	2.300833
C	4.162834	-2.207999	2.523877
H	4.138734	-3.853720	1.129378
H	3.955336	-0.419991	3.717216
H	4.947831	-2.633307	3.142879
C	2.522571	1.342655	-0.882005
C	3.653407	0.550911	-1.178399
C	2.679430	2.742498	-0.944332
C	4.883807	1.129197	-1.482928
H	3.560898	-0.528456	-1.188218
C	3.912934	3.320459	-1.248138
H	1.832608	3.390657	-0.751776
C	5.025924	2.519850	-1.513321
H	5.732158	0.489051	-1.710694

H	4.000712	4.403360	-1.278542
H	5.985185	2.970749	-1.751372
O	-1.241009	0.965454	2.560546
C	-2.113494	1.836589	2.574740
Pd	-0.865810	-0.584864	0.878601
C	-0.182983	-1.756363	-0.628184
H	-2.516914	-2.424331	-0.516471
C	-0.454541	-3.730508	-1.672815
O	-3.368735	-2.118386	-0.901760
C	-3.842867	-1.108226	-0.194486
O	-3.294081	-0.721885	0.847736
C	-5.053665	-0.454312	-0.775675
H	-4.704900	0.376985	-1.400910
H	-5.676273	-0.047563	0.022671
H	-5.621846	-1.146208	-1.398646
N	-0.384950	-1.617545	-1.986990
N	-0.528869	-2.801477	-2.631359
C	-0.233144	-3.151533	-0.410179
H	-0.570047	-4.777095	-1.927673
H	-0.103645	-3.653298	0.537251
O	-3.293091	1.706884	1.984569
H	-3.359325	0.818603	1.545485
C	-1.939020	3.155654	3.267343
H	-2.020757	3.961083	2.530048
H	-2.737029	3.299699	4.002078
H	-0.965903	3.198528	3.755652

XII

Electronic Energy BS1 = -1619.96878429 Hartree

Electronic Energy BS2 = -1621.14078690 Hartree

Zero-point Energy Correction = 0.481783 Hartree

Thermal Correction to Enthalpy = 0.515833 Hartree

Thermal Correction to Free Energy = 0.413229 Hartree

Chemical symbol X, Y, Z

C	-0.182126	2.674073	-0.159449
C	0.063848	1.489484	-0.882417
C	-0.919681	1.048706	-1.794319
C	-2.098796	1.780412	-1.963970
C	-2.331527	2.943225	-1.231189
C	-1.368452	3.385765	-0.320174
H	0.563610	3.027849	0.545326
H	-2.838546	1.430974	-2.678632
H	-3.253873	3.499295	-1.371166
H	-1.538008	4.287797	0.260772
C	-0.689953	-0.223773	-2.569903
H	0.264237	-0.180678	-3.109447
H	-1.485042	-0.409386	-3.291839
C	1.331914	0.751287	-0.632303
C	1.396294	-0.604221	-0.466493
C	2.681489	-1.308784	-0.163080

C	3.678587	-1.395167	-1.146151
C	2.907871	-1.902227	1.088622
C	4.878710	-2.058002	-0.882523
H	3.511235	-0.933512	-2.114929
C	4.107713	-2.566262	1.350420
H	2.143426	-1.825825	1.857237
C	5.096688	-2.646535	0.366048
H	5.642712	-2.114410	-1.653122
H	4.272230	-3.014223	2.326624
H	6.031136	-3.161214	0.571616
C	2.541357	1.607644	-0.445720
C	2.931356	2.489581	-1.467656
C	3.283693	1.588419	0.746333
C	4.052994	3.305390	-1.315722
H	2.353398	2.525627	-2.387049
C	4.399747	2.411867	0.902446
H	2.980150	0.927517	1.551453
C	4.792390	3.268238	-0.129823
H	4.348402	3.971156	-2.122048
H	4.960384	2.386456	1.832880
H	5.663449	3.906203	-0.009058
O	-1.532357	0.331481	2.740199
C	-2.376461	1.223646	2.595157
Pd	-0.841977	-1.233212	1.355664
C	0.220735	-1.489910	-0.602157
H	-3.086349	-1.832967	-2.026344
C	-1.174721	-3.250397	-0.789691

O	-3.939573	-1.304419	-2.170778
C	-4.223370	-0.616120	-1.078880
O	-3.582804	-0.745678	-0.033141
C	-5.372562	0.341104	-1.222389
H	-4.965353	1.356754	-1.272704
H	-6.008489	0.280792	-0.335943
H	-5.954716	0.145150	-2.123493
N	-0.648404	-1.359952	-1.661399
N	-1.514451	-2.403433	-1.756689
C	-0.079731	-2.765766	-0.015048
H	-1.714005	-4.181200	-0.673591
H	0.583059	-3.374199	0.586633
O	-3.355634	1.202678	1.715442
H	-3.355925	0.399848	1.112773
C	-2.362048	2.470916	3.432029
H	-2.058448	3.310578	2.796783
H	-3.364153	2.688004	3.811422
H	-1.656438	2.366977	4.256164

17.3. Cartesian coordinates in Å, energy values in Hartrees and imaginary frequencies in cm^{-1} for transition states (TS) of species in Figure S4

XIII

Electronic Energy BS1 = -1454.76099906 Hartree

Electronic Energy BS2 = -1457.10892745 Hartree

Zero-point Energy Correction = 0.338090 Hartree

Thermal Correction to Enthalpy = 0.368441 Hartree

Thermal Correction to Free Energy = 0.272287 Hartree

Chemical symbol X, Y, Z

N	-0.906918	-1.418413	0.586466
N	-1.993695	-2.029327	0.052657
C	-2.258068	-3.178293	0.714062
H	-3.101279	-3.791108	0.430869
C	-1.302771	-3.324946	1.708301
H	-1.216915	-4.132139	2.420025
C	-0.479593	-2.196668	1.590916
H	0.385431	-1.900715	2.165999
C	-2.770067	-1.425535	-1.034306
H	-3.583851	-2.126098	-1.235879
H	-2.133499	-1.364443	-1.917708
C	-3.126951	1.021186	-1.539018
C	-3.301492	-0.056471	-0.663527
C	-3.960742	0.147410	0.555936
C	-4.434871	1.414677	0.895813
C	-4.260135	2.489349	0.016568
C	-3.609019	2.289395	-1.202691
Pd	-0.052395	0.334036	-0.104783
O	0.701509	2.096563	-0.791072
C	1.959219	2.323019	-0.970324
C	2.243817	3.673920	-1.608047
O	-0.133876	1.122030	1.788910
C	0.767710	0.810023	2.653862
C	0.521282	1.366376	4.044181
Ag	2.574358	-0.609738	0.315845
O	2.907751	1.560493	-0.700533
O	1.769340	0.080214	2.464819
H	-2.597230	0.868213	-2.474955
H	-3.467406	3.119425	-1.889283
H	-4.630418	3.475565	0.282077
H	-4.942827	1.564657	1.844464
H	-4.092172	-0.685276	1.242481
H	-0.045324	2.298751	4.005576
H	-0.064845	0.631854	4.609054
H	1.469816	1.518831	4.563111
H	1.600248	4.447565	-1.181776
H	3.293411	3.943591	-1.479373
H	2.023290	3.610145	-2.679753
O	1.793732	-1.803125	-1.581190

C	0.859461	-1.383063	-2.296369
O	0.020311	-0.445266	-2.005118
C	0.610771	-2.028067	-3.647743
H	0.265734	-1.290129	-4.375522
H	1.514308	-2.523047	-4.007974
H	-0.178287	-2.780556	-3.532231

XIV

Electronic Energy BS1 = -1454.72895137 Hartree

Electronic Energy BS2 = -1457.07623342 Hartree

Zero-point Energy Correction = 0.338058 Hartree

Thermal Correction to Enthalpy = 0.367973 Hartree

Thermal Correction to Free Energy = 0.275555 Hartree

Chemical symbol X, Y, Z

N	-1.680335	1.283725	0.151545
N	-2.109308	0.999606	1.410055
C	-2.691787	2.082825	1.963506
H	-3.113200	2.032231	2.956818
C	-2.627617	3.115862	1.037558
H	-3.000857	4.122107	1.154175
C	-1.986190	2.567666	-0.080294
H	-1.722710	3.021232	-1.023758
C	-2.002714	-0.369058	1.937798
H	-2.677575	-0.425875	2.793339
H	-0.972833	-0.557116	2.259517
C	-1.387657	-1.930006	0.057193
C	-2.403779	-1.313223	0.829056
C	-3.741505	-1.450108	0.461666
C	-4.082537	-2.202152	-0.665989
C	-3.091452	-2.800368	-1.453899
C	-1.754250	-2.672019	-1.091519
Pd	-0.486159	-0.004044	-0.943386
O	0.736209	-1.281701	-1.920730
C	1.995688	-1.320469	-1.583403
C	2.702994	-2.572050	-2.068724
O	0.287655	1.618851	-1.968573
C	1.005445	2.544334	-1.440711
C	1.336768	3.694176	-2.373876
Ag	1.466376	0.855996	1.097399
O	2.598724	-0.482361	-0.901666
O	1.412471	2.611467	-0.257732
O	1.357930	-0.896551	2.382665
C	1.923749	-1.936082	1.847194
C	3.447662	-2.002556	1.922635
O	1.313231	-2.855566	1.274144
H	-0.382782	-2.060124	0.484608
H	-0.972419	-3.138693	-1.679594
H	-3.367760	-3.370335	-2.335646
H	-5.127834	-2.308079	-0.942196
H	-4.514634	-0.957774	1.044907

H	3.838768	-1.523269	1.018320
H	3.833319	-1.468244	2.795138
H	3.789488	-3.040969	1.934564
H	1.178876	3.424863	-3.418934
H	0.684671	4.538286	-2.122434
H	2.371054	4.008480	-2.214788
H	2.248037	-2.980186	-2.973512
H	3.763160	-2.367681	-2.232600
H	2.609715	-3.311729	-1.265121

TS_{XIV-XV}

Imaginary Freq = -789.0905 (cm^{-1})

Electronic Energy BS1 = -1454.72031950 Hartree

Electronic Energy BS2 = -1457.06589893 Hartree

Zero-point Energy Correction = 0.332856 Hartree

Thermal Correction to Enthalpy = 0.362343 Hartree

Thermal Correction to Free Energy = 0.270661 Hartree

Chemical symbol X, Y, Z

N	-0.843934	1.950392	-0.006134
N	-1.628391	1.969742	1.099014
C	-1.806296	3.235697	1.530084
H	-2.415492	3.440145	2.398372
C	-1.103197	4.074032	0.674775
H	-1.031293	5.149935	0.727326
C	-0.518225	3.223930	-0.271996
H	0.111475	3.450864	-1.118527
C	-2.196217	0.726443	1.636479
H	-2.904772	1.019369	2.411755
H	-1.385198	0.137535	2.080539
C	-2.023563	-0.773922	-0.367562
C	-2.857683	-0.048544	0.522592
C	-4.237577	-0.029607	0.343297
C	-4.808808	-0.713147	-0.737929
C	-4.005784	-1.418939	-1.639765
C	-2.624712	-1.446979	-1.455905
Pd	-0.199647	0.223499	-0.935132
O	0.404709	-1.478353	-1.864293
C	1.406248	-2.152066	-1.386961
C	1.599532	-3.498266	-2.063630
O	1.395038	1.321144	-1.778965
C	2.385564	1.831906	-1.151208
C	3.272281	2.748275	-1.975638
Ag	1.685867	0.089187	1.261550
O	2.151048	-1.811504	-0.454276
O	2.671795	1.686701	0.065428
O	0.388333	-1.316429	2.423080
C	0.220037	-2.421513	1.816258
C	1.104714	-3.597532	2.180253
O	-0.627820	-2.620929	0.891343

H	-1.179387	-1.496630	0.243748
H	-1.989351	-2.009387	-2.133337
H	-4.459487	-1.944645	-2.475058
H	-5.886193	-0.690479	-0.876327
H	-4.869348	0.519988	1.035967
H	1.950228	-3.581576	1.483321
H	1.484429	-3.505259	3.200023
H	0.574823	-4.544351	2.052665
H	3.178770	2.539841	-3.042707
H	2.959864	3.783472	-1.794378
H	4.313076	2.650169	-1.658837
H	1.328171	-3.460073	-3.121066
H	2.631473	-3.835607	-1.948556
H	0.939825	-4.220619	-1.569276

XV

Electronic Energy BS1 = -1454.75641729 Hartree

Electronic Energy BS2 = -1457.10232574 Hartree

Zero-point Energy Correction = 0.338060 Hartree

Thermal Correction to Enthalpy = 0.368547 Hartree

Thermal Correction to Free Energy = 0.272333 Hartree

Chemical symbol X, Y, Z

N	-1.685712	1.658503	0.013308
N	-1.215948	2.426786	-0.992124
C	-2.192476	3.225198	-1.475156
H	-1.995585	3.902231	-2.293401
C	-3.344278	2.966151	-0.743966
H	-4.310704	3.433241	-0.860165
C	-2.977287	1.976528	0.178889
H	-3.557123	1.475355	0.939380
C	0.152711	2.251327	-1.479580
H	0.321112	3.031879	-2.222022
H	0.212427	1.278928	-1.980162
C	1.048895	1.518437	0.787899
C	1.173768	2.325151	-0.363947
C	2.283746	3.172410	-0.512443
C	3.270180	3.233158	0.472756
C	3.146836	2.444474	1.618481
C	2.047149	1.593932	1.772290
Pd	-0.526683	0.309689	1.032950
O	0.666657	-0.987089	2.115933
C	1.345710	-1.936601	1.581591
C	2.398304	-2.549682	2.487796
O	-2.357084	-0.868613	1.347580
C	-3.048545	-1.402584	0.424141
C	-4.510554	-1.673610	0.741964
Ag	-0.431896	-1.589150	-1.105905
O	1.243053	-2.364504	0.408026
O	-2.652004	-1.701386	-0.740796
O	1.575297	-0.777262	-2.308445

C	2.651967	-1.019903	-1.763197
C	3.462112	-2.256852	-1.999344
O	3.202862	-0.200225	-0.869890
H	2.567277	0.516066	-0.665336
H	1.979421	0.961683	2.651470
H	3.910345	2.481844	2.391433
H	4.125642	3.890112	0.345239
H	2.372266	3.779833	-1.409858
H	3.245630	-2.937546	-1.168909
H	3.166160	-2.726718	-2.937708
H	4.532159	-2.037533	-1.996372
H	-4.668145	-1.777836	1.817349
H	-5.101850	-0.819699	0.389599
H	-4.863053	-2.565478	0.218831
H	2.095630	-2.505798	3.536113
H	2.607174	-3.580361	2.194449
H	3.319676	-1.965449	2.375337

IV

Electronic Energy BS1 = -1994.27391104 Hartree

Electronic Energy BS2 = -1996.75052566 Hartree

Zero-point Energy Correction = 0.530366 Hartree

Thermal Correction to Enthalpy = 0.573627 Hartree

Thermal Correction to Free Energy = 0.448334 Hartree

Chemical symbol X, Y, Z

C	2.084239	-1.320212	2.694137
C	1.562656	-1.750791	1.466986
C	1.226084	-3.107057	1.299729
C	1.386566	-3.994041	2.373425
C	1.884461	-3.555661	3.601370
C	2.244089	-2.217244	3.756518
H	2.361439	-0.281617	2.839662
H	1.120582	-5.039962	2.238689
H	2.000951	-4.257151	4.422549
H	2.646820	-1.862077	4.701584
C	0.682780	-3.636306	-0.009124
H	-0.305991	-3.227429	-0.240949
H	0.613054	-4.724699	0.008652
N	1.903500	-2.009637	-1.352826
Pd	1.409280	-0.456002	-0.063048
C	0.174926	0.810070	1.309617
C	1.245773	1.420580	1.141391
N	1.554744	-3.293446	-1.136299
C	1.999141	-4.079635	-2.139048
H	1.794991	-5.140419	-2.138893
C	2.678356	-3.267124	-3.038995
H	3.171340	-3.571269	-3.950225
C	2.590774	-1.974912	-2.502796
H	2.966729	-1.030457	-2.866621
C	2.278385	2.407276	1.038498

C	3.637360	2.046918	0.970890
C	1.910781	3.766421	0.979482
C	4.611171	3.034585	0.845884
H	3.912145	0.997618	1.009829
C	2.894512	4.745311	0.859272
H	0.860674	4.037334	1.017975
C	4.243467	4.383315	0.788558
H	5.658448	2.752651	0.791203
H	2.607094	5.791414	0.810965
H	5.006272	5.149729	0.686941
C	-1.094440	0.430565	1.863555
C	-1.686676	1.285945	2.814456
C	-1.756161	-0.748115	1.479472
C	-2.918503	0.954433	3.372053
H	-1.176051	2.199133	3.104343
C	-2.986623	-1.070671	2.046404
H	-1.308036	-1.389363	0.730957
C	-3.569875	-0.224480	2.993128
H	-3.371445	1.616685	4.103975
H	-3.492264	-1.981515	1.741593
H	-4.531173	-0.477711	3.430491
O	1.760455	0.881191	-1.791783
C	1.298756	2.033038	-2.067337
C	2.303598	3.056596	-2.566278
H	3.085073	3.191552	-1.812598
H	2.784428	2.675631	-3.473463
H	1.827958	4.014746	-2.780627

O	0.089098	2.386731	-1.973667
Ag	-1.457528	0.857160	-1.641817
O	-3.146461	-0.578111	-1.712001
C	-2.789517	-1.819893	-1.559373
O	-1.622120	-2.221149	-1.664645
C	-3.929657	-2.777538	-1.250666
H	-4.595969	-2.844369	-2.117835
H	-4.522753	-2.395947	-0.414173
H	-3.549395	-3.772157	-1.011444
O	-4.913609	0.557583	-0.228490
C	-4.430453	1.714381	0.202031
O	-3.364886	2.200711	-0.166677
C	-5.356563	2.408770	1.170303
H	-6.219138	2.800930	0.619424
H	-4.838679	3.234733	1.658543
H	-5.731034	1.703476	1.915831
H	-4.243089	0.098853	-0.840063

$TS_{IV\text{-}V}$

Imaginary Freq = -199.9073 (cm^{-1})

Electronic Energy BS1 = -1994.25421169 Hartree

Electronic Energy BS2 = -1996.72833072 Hartree

Zero-point Energy Correction = 0.530361 Hartree

Thermal Correction to Enthalpy = 0.572559 Hartree

Thermal Correction to Free Energy = 0.450615 Hartree

Chemical symbol X, Y, Z

C	-2.311859	-0.492348	3.066035
C	-2.098837	-0.076792	1.744040
C	-3.008078	0.800966	1.119052
C	-4.118161	1.248642	1.838446
C	-4.328629	0.840519	3.159956
C	-3.432728	-0.039036	3.769289
H	-1.587655	-1.135755	3.554060
H	-4.827080	1.916616	1.356095
H	-5.189545	1.210694	3.708737
H	-3.587445	-0.357297	4.796234
C	-2.821847	1.189511	-0.326631
H	-1.789970	1.479824	-0.534267
H	-3.474586	2.016141	-0.606110
N	-2.591837	-1.143454	-1.020919
Pd	-1.042831	-1.325365	0.480059
C	-0.032515	0.297483	1.720848
C	0.558390	-0.846101	1.652506
N	-3.157764	0.068944	-1.214478
C	-3.927040	0.070375	-2.323133
H	-4.451280	0.962929	-2.631016
C	-3.862334	-1.202554	-2.875565
H	-4.367891	-1.556569	-3.761706
C	-3.009992	-1.924254	-2.024340
H	-2.675961	-2.950490	-2.076309
C	1.793368	-1.513898	2.009021
C	2.038690	-2.853397	1.658049
C	2.787652	-0.789803	2.701914
C	3.255421	-3.451754	1.985666
H	1.275088	-3.407743	1.124927
C	3.999256	-1.395042	3.022446
H	2.602984	0.246192	2.969702
C	4.238410	-2.727368	2.665130
H	3.435568	-4.486463	1.707994
H	4.760096	-0.827418	3.550772
H	5.185636	-3.196139	2.916062
C	0.141921	1.737604	1.760968
C	1.242071	2.262231	1.055800
C	-0.690406	2.605454	2.490409
C	1.505869	3.629922	1.081537
H	1.871268	1.585385	0.493274
C	-0.420920	3.972269	2.508903
H	-1.533390	2.209673	3.044713
C	0.672293	4.488563	1.803970
H	2.349630	4.016041	0.518979
H	-1.065225	4.637394	3.076400
H	0.871609	5.556299	1.817427
O	-0.397069	-3.151898	-0.451725
C	0.315361	-3.278167	-1.499860
C	0.623555	-4.702996	-1.923604
H	1.526287	-5.029431	-1.393328

H	-0.191578	-5.376761	-1.651546
H	0.818479	-4.755834	-2.996471
O	0.813332	-2.346089	-2.185726
Ag	0.680746	-0.172925	-1.820663
O	0.944577	1.991527	-2.084211
C	-0.106996	2.674859	-2.439198
O	-1.183297	2.159694	-2.759456
C	0.077168	4.183288	-2.399241
H	0.988718	4.474367	-2.930255
H	0.193024	4.496696	-1.355549
H	-0.784096	4.692294	-2.835318
O	3.332450	2.644677	-1.490093
C	3.967079	1.540744	-1.115054
O	3.458980	0.422647	-1.108681
C	5.382670	1.807276	-0.668386
H	5.926207	2.357692	-1.442255
H	5.892450	0.869435	-0.446377
H	5.363385	2.435522	0.228798
H	2.362868	2.430921	-1.742133

XVI

Electronic Energy BS1 = -1994.28658781 Hartree

Electronic Energy BS2 = -1996.75410501 Hartree

Zero-point Energy Correction = 0.531542 Hartree

Thermal Correction to Enthalpy = 0.574288 Hartree

Thermal Correction to Free Energy = 0.450082 Hartree

Chemical symbol X, Y, Z

C	2.194115	1.849605	-2.636860
C	1.727915	1.625615	-1.312104
C	2.685366	1.614003	-0.256526
C	4.029942	1.867334	-0.544494
C	4.455977	2.116399	-1.847866
C	3.537803	2.083958	-2.902653
H	1.463106	1.875945	-3.438409
H	4.752846	1.835801	0.266204
H	5.504774	2.318974	-2.043836
H	3.865631	2.261565	-3.922275
C	2.327939	1.155770	1.139461
H	1.248498	1.121653	1.289682
H	2.765143	1.805403	1.899400
N	2.834011	-1.138860	0.384579
Pd	1.278792	-0.634587	-1.205621
C	0.205758	1.606111	-1.148864
C	-0.328919	0.458261	-1.619795
N	2.888136	-0.188487	1.361321
C	3.408462	-0.690675	2.500923
H	3.508018	-0.072770	3.379879
C	3.718376	-2.022540	2.265258
H	4.168451	-2.720665	2.955642
C	3.335459	-2.251298	0.931991

H	3.390694	-3.157083	0.344160
C	-1.648956	0.086479	-2.090676
C	-2.238058	-1.156744	-1.787001
C	-2.375144	1.004782	-2.883327
C	-3.521349	-1.459460	-2.236890
H	-1.693199	-1.870574	-1.184201
C	-3.655568	0.694960	-3.331806
H	-1.923909	1.960740	-3.130341
C	-4.234724	-0.538249	-3.009982
H	-3.966929	-2.415524	-1.978892
H	-4.202703	1.413103	-3.936008
H	-5.233573	-0.779641	-3.361964
C	-0.459044	2.758324	-0.510737
C	-1.741823	2.631416	0.054839
C	0.200194	3.997265	-0.414871
C	-2.350162	3.715180	0.681256
H	-2.248907	1.676592	0.028652
C	-0.413296	5.082145	0.213627
H	1.193703	4.114651	-0.837143
C	-1.690480	4.947434	0.763549
H	-3.334883	3.590763	1.122758
H	0.109405	6.032697	0.274160
H	-2.164778	5.790061	1.258601
O	0.712493	-2.591484	-1.768371
C	0.386736	-3.503549	-0.937432
C	0.296749	-4.906482	-1.510693
H	-0.707726	-5.041795	-1.928803

H	1.021881	-5.048661	-2.314533
H	0.446190	-5.651704	-0.727160
O	0.095021	-3.349226	0.278479
Ag	-0.342310	-1.438260	1.301414
O	-1.074826	0.354283	2.318680
C	-0.391637	0.832788	3.321740
O	0.664597	0.342445	3.732232
C	-0.988015	2.089613	3.935735
H	-2.032817	1.916091	4.213147
H	-0.981683	2.891703	3.189648
H	-0.420981	2.402707	4.814149
O	-3.550693	0.429027	1.676643
C	-3.927864	-0.787111	1.292693
O	-3.215939	-1.783371	1.360314
C	-5.335687	-0.805140	0.751820
H	-6.022709	-0.352261	1.473111
H	-5.643593	-1.827396	0.531389
H	-5.374587	-0.206983	-0.164267
H	-2.581910	0.426676	1.992310

V

Electronic Energy BS1 = -1994.29895041 Hartree

Electronic Energy BS2 = -1996.76211653 Hartree

Zero-point Energy Correction = 0.532581 Hartree

Thermal Correction to Enthalpy = 0.574490 Hartree

Thermal Correction to Free Energy = 0.454500 Hartree

Chemical symbol X, Y, Z			
O	-1.104302	1.845768	-0.710714
C	0.800637	-1.859633	-1.925786
C	1.387312	-1.750022	-0.654856
C	1.378430	-2.876008	0.192998
C	0.764902	-4.060531	-0.227906
C	0.177779	-4.158055	-1.491189
C	0.200343	-3.054454	-2.346815
H	0.820806	-1.000838	-2.586643
H	0.745367	-4.913824	0.444970
H	-0.289924	-5.086897	-1.804085
H	-0.238137	-3.116150	-3.338927
C	1.984865	-2.794441	1.574687
H	3.048077	-2.546172	1.530344
H	1.873594	-3.745427	2.103399
C	2.047652	-0.452664	-0.276614
C	1.355129	0.606833	0.201041
C	1.831499	2.001690	0.232043
C	1.749217	2.811617	1.380101
C	2.321437	2.579419	-0.957750
C	2.167812	4.142201	1.346379
H	1.359360	2.391659	2.300179
C	2.735284	3.909731	-0.990794
H	2.370253	1.970135	-1.854956
C	2.662580	4.697629	0.162875
H	2.106980	4.746917	2.247239
H	3.111207	4.333558	-1.918114
H	2.983586	5.735138	0.137696
C	3.529298	-0.416115	-0.481907
C	4.373019	0.177586	0.475537
C	4.120045	-1.010482	-1.611725
C	5.755166	0.203855	0.293737
H	3.935239	0.605881	1.370676
C	5.503587	-0.981231	-1.794060
H	3.492401	-1.489580	-2.356807
C	6.327829	-0.371636	-0.844617
H	6.386927	0.665347	1.048046
H	5.937443	-1.436913	-2.680152
H	7.404915	-0.353017	-0.985495
Pd	-0.531868	0.424484	0.815959
C	0.038422	-1.388585	2.356577
H	-0.716512	-2.048312	1.950891
C	1.272976	0.047108	3.543161
O	-2.900923	-1.861816	0.655428
C	-3.231365	-0.924836	1.446059
O	-2.596391	0.161767	1.619510
C	-4.510512	-1.116459	2.242053
H	-4.591283	-0.392570	3.054803
H	-4.557255	-2.134485	2.638681
H	-5.361710	-0.987811	1.564170
N	1.356720	-1.726527	2.354520

N	2.114335	-0.856087	3.057637
C	-0.062891	-0.211145	3.124590
H	1.636140	0.854987	4.163622
H	-0.969871	0.253271	3.481103
C	-1.017689	1.685934	-1.994448
C	-0.912163	2.974810	-2.780905
Ag	-1.894926	-1.113447	-1.233297
O	-1.031758	0.588773	-2.587324
O	-4.183487	0.614962	-1.202968
C	-4.487050	1.545427	-0.467130
C	-5.876762	1.770427	0.069515
O	-3.630062	2.475530	-0.049501
H	-2.705282	2.217946	-0.308724
H	-6.579415	1.083059	-0.402027
H	-5.870795	1.602147	1.151635
H	-6.188571	2.805082	-0.100951
H	-1.766418	3.616784	-2.542271
H	-0.006411	3.506513	-2.473375
H	-0.885995	2.779941	-3.853726

TS_{V-XVII}

Imaginary Freq = -1325.2430 (cm^{-1})

Electronic Energy BS1 = -1994.26676254 Hartree

Electronic Energy BS2 = -1996.73041175 Hartree

Zero-point Energy Correction = 0.527031 Hartree

Thermal Correction to Enthalpy = 0.568571 Hartree

Thermal Correction to Free Energy = 0.448910 Hartree

Chemical symbol X, Y, Z

O	-1.054894	2.167708	-0.299663
C	0.324157	-1.791967	-2.146552
C	1.009352	-1.735880	-0.914429
C	0.830779	-2.778875	0.010554
C	-0.052948	-3.827080	-0.280420
C	-0.750782	-3.868413	-1.486013
C	-0.552486	-2.856214	-2.433513
H	0.523187	-1.033444	-2.897987
H	-0.190029	-4.620106	0.449770
H	-1.429462	-4.688821	-1.697902
H	-1.044745	-2.901972	-3.400945
C	1.619411	-2.795183	1.295308
H	2.668265	-2.555387	1.097458
H	1.582158	-3.793576	1.739650
C	1.887315	-0.570172	-0.570179
C	1.392634	0.439225	0.176595
C	2.110581	1.685013	0.509452
C	2.344151	2.060070	1.846284
C	2.544577	2.545521	-0.516394
C	3.008588	3.249822	2.144250
H	2.011248	1.404083	2.645667

C	3.201453	3.739016	-0.215105
H	2.358109	2.268778	-1.549547
C	3.437365	4.095966	1.116010
H	3.192860	3.517511	3.181355
H	3.528580	4.391030	-1.020654
H	3.949368	5.025038	1.350407
C	3.314442	-0.660681	-1.007765
C	4.356761	-0.406529	-0.096372
C	3.656451	-1.048907	-2.315316
C	5.691867	-0.509532	-0.486379
H	4.111472	-0.134532	0.925027
C	4.992973	-1.149312	-2.706047
H	2.873636	-1.266026	-3.035101
C	6.017129	-0.877718	-1.795151
H	6.478918	-0.311386	0.236325
H	5.233292	-1.441300	-3.724892
H	7.056903	-0.959477	-2.099259
Pd	-0.479670	0.448994	0.883579
C	0.027576	-1.039054	2.327889
H	-1.163433	-1.513373	1.874701
C	1.182149	-0.960302	4.260638
O	-2.393028	-1.845204	1.741634
C	-3.074835	-0.755787	1.775553
O	-2.572152	0.395225	1.654418
C	-4.568387	-0.893486	1.947829
H	-5.023348	0.073656	2.165171
H	-4.792515	-1.608163	2.743836

H	-4.989464	-1.284027	1.015831
N	1.151656	-1.844938	2.315799
N	1.842389	-1.815873	3.459319
C	0.048103	-0.458184	3.617671
H	1.556810	-0.759267	5.256286
H	-0.685759	0.232772	4.008448
C	-0.727920	2.368058	-1.546416
C	-0.465134	3.815432	-1.904995
Ag	-1.767540	-0.544330	-1.507338
O	-0.672723	1.478563	-2.412629
O	-3.979852	0.168940	-1.169040
C	-4.450512	1.230344	-0.746887
C	-5.931328	1.469796	-0.663838
O	-3.739341	2.259466	-0.333280
H	-2.759477	2.072911	-0.284890
H	-6.476132	0.620137	-1.074102
H	-6.213002	1.622093	0.382922
H	-6.189056	2.382247	-1.210232
H	-1.384842	4.391603	-1.754760
H	0.294202	4.226574	-1.234616
H	-0.141865	3.910342	-2.942603

XVII

Electronic Energy BS1 = -1994.28951088 Hartree

Electronic Energy BS2 = -1996.75472592 Hartree

Zero-point Energy Correction = 0.532976 Hartree

Thermal Correction to Enthalpy = 0.574678 Hartree

Thermal Correction to Free Energy = 0.455494 Hartree

Chemical symbol X, Y, Z

O	-1.012493	2.176116	0.332245
C	0.460177	-1.052165	-2.598228
C	1.063058	-1.391783	-1.366531
C	0.767700	-2.637494	-0.788674
C	-0.159634	-3.493050	-1.402439
C	-0.783458	-3.140874	-2.596730
C	-0.460318	-1.923037	-3.210735
H	0.750839	-0.133682	-3.100460
H	-0.388767	-4.445834	-0.931821
H	-1.498058	-3.813116	-3.061617
H	-0.892010	-1.659368	-4.172275
C	1.434169	-3.051146	0.501192
H	2.421625	-2.589340	0.585474
H	1.563713	-4.134423	0.524257
C	1.960181	-0.432029	-0.644534
C	1.477785	0.252813	0.417045
C	2.230937	1.284562	1.162583
C	2.440037	1.179197	2.550840
C	2.728324	2.418662	0.493662
C	3.145312	2.164773	3.241187
H	2.051202	0.313012	3.078864
C	3.423654	3.409165	1.188365
H	2.559857	2.515579	-0.574392
C	3.638199	3.285540	2.564149
H	3.309417	2.059343	4.310360
H	3.796814	4.278993	0.654283
H	4.180334	4.056273	3.104787
C	3.390334	-0.387439	-1.084367
C	4.426597	-0.487436	-0.136124
C	3.745813	-0.301993	-2.442398
C	5.764878	-0.474329	-0.527790
H	4.175041	-0.581232	0.915034
C	5.085828	-0.285497	-2.834416
H	2.974018	-0.239724	-3.202033
C	6.102535	-0.368787	-1.880125
H	6.544674	-0.555747	0.224792
H	5.333738	-0.208394	-3.889730
H	7.144758	-0.359302	-2.186581
Pd	-0.422197	0.137369	1.045615
C	-0.022158	-1.577146	2.018294
H	-1.843007	-1.919958	0.662120
C	-0.069544	-3.092578	3.672786
O	-2.820235	-2.028400	0.709237
C	-3.328675	-0.965663	1.326595
O	-2.646473	-0.000428	1.678247
C	-4.802714	-1.060962	1.566256
H	-5.185639	-0.104240	1.919676
H	-4.989109	-1.833058	2.320613

H	-5.312969	-1.361405	0.647717
N	0.649735	-2.726167	1.695259
N	0.634950	-3.658668	2.689810
C	-0.506048	-1.803711	3.315520
H	-0.242535	-3.638986	4.592808
H	-1.110252	-1.123607	3.899386
C	-0.520074	2.817980	-0.688024
C	-0.113577	4.250604	-0.416818
Ag	-1.561260	0.174016	-1.756536
O	-0.424447	2.339390	-1.833569
O	-3.818055	0.654767	-1.237952
C	-4.314447	1.577816	-0.580456
C	-5.792038	1.851305	-0.580775
O	-3.631146	2.416273	0.168682
H	-2.648766	2.202842	0.216012
H	-6.323074	1.059415	-1.108113
H	-6.157468	1.936946	0.446076
H	-5.975846	2.810206	-1.077729
H	-1.011309	4.827850	-0.167603
H	0.552850	4.287989	0.448441
H	0.373202	4.695571	-1.285930

VIII

Electronic Energy BS1 = -1994.29330748 Hartree

Electronic Energy BS2 = -1996.75775040 Hartree

Zero-point Energy Correction = 0.532300 Hartree

Thermal Correction to Enthalpy = 0.574363 Hartree

Thermal Correction to Free Energy = 0.451480 Hartree

Chemical symbol X, Y, Z

C	-2.259589	-2.592691	1.229035
C	-2.049840	-1.821634	0.076556
C	-1.276334	-2.355622	-0.975737
C	-0.678187	-3.612520	-0.822284
C	-0.858939	-4.354407	0.345819
C	-1.664469	-3.847095	1.369422
H	-2.878263	-2.190359	2.026601
H	-0.078283	-4.016284	-1.634205
H	-0.387136	-5.327143	0.450608
H	-1.824970	-4.424198	2.275904
C	-1.149282	-1.617625	-2.286765
H	-2.075685	-1.073558	-2.496888
H	-0.988139	-2.329855	-3.097912
C	-2.615598	-0.434280	-0.019363
C	-1.757945	0.609409	0.033169
C	-2.070506	2.029907	-0.206311
C	-2.575362	2.437739	-1.455284
C	-1.837769	3.008871	0.778775
C	-2.849818	3.782376	-1.707186
H	-2.750638	1.689898	-2.223306
C	-2.122900	4.350798	0.526931

H	-1.440613	2.702227	1.742137
C	-2.628543	4.744098	-0.716851
H	-3.238054	4.079609	-2.677843
H	-1.948839	5.092310	1.302223
H	-2.844308	5.790626	-0.912987
C	-4.094928	-0.321166	-0.154502
C	-4.801395	-1.277098	-0.909198
C	-4.829561	0.705703	0.467947
C	-6.185808	-1.192562	-1.061670
H	-4.257197	-2.088154	-1.384262
C	-6.213776	0.786859	0.318867
H	-4.312144	1.437701	1.078620
C	-6.899425	-0.158260	-0.450416
H	-6.707150	-1.937325	-1.657193
H	-6.759593	1.586378	0.812811
H	-7.977952	-0.094002	-0.564393
C	0.504180	0.139851	-1.418669
C	1.330935	0.554185	-3.488483
N	-0.036457	-0.659494	-2.376330
N	0.448210	-0.425765	-3.637207
C	1.433475	0.953000	-2.124123
H	1.864516	0.944359	-4.346624
H	1.921480	1.838096	-1.733913
Pd	0.126195	0.194671	0.545543
O	2.223869	-0.540254	1.346186
C	2.436014	-1.814108	1.102019
C	1.918784	-2.783358	2.138282
O	-0.450177	0.477889	2.696910
C	0.238388	0.428339	3.727607
C	-0.326720	0.761072	5.075879
Ag	3.258189	-0.465909	-1.486283
O	3.020673	-2.220543	0.085292
O	1.505187	0.080775	3.741629
O	5.017524	0.959591	-0.515562
C	4.743521	1.776673	0.364813
C	5.528340	3.036993	0.592048
O	3.727622	1.641583	1.205786
H	3.250685	0.772028	1.087044
H	4.869843	3.901959	0.460901
H	5.900593	3.060351	1.620782
H	6.360065	3.094817	-0.109512
H	-1.353209	1.114241	4.981405
H	-0.297952	-0.132903	5.707764
H	0.292915	1.524411	5.556102
H	2.375011	-2.567085	3.109609
H	0.836911	-2.655212	2.242592
H	2.137514	-3.811468	1.850376
H	1.809244	-0.174816	2.804733

$TS_{VIII\text{-}IX}$

Imaginary Freq = -314.7637 (cm^{-1})

Electronic Energy BS1 = -1994.27411890 Hartree

Electronic Energy BS2 = -1996.73492073 Hartree

Zero-point Energy Correction = 0.530229 Hartree

Thermal Correction to Enthalpy = 0.572121 Hartree

Thermal Correction to Free Energy = 0.449674 Hartree

Chemical symbol X, Y, Z

C	2.057139	2.753231	0.058506
C	1.883937	1.605335	-0.732839
C	1.230086	1.728217	-1.978663
C	0.710102	2.966899	-2.368048
C	0.854837	4.093308	-1.555354
C	1.541074	3.984707	-0.342605
H	2.576218	2.664922	1.008237
H	0.203329	3.049991	-3.326074
H	0.448548	5.048875	-1.873612
H	1.671196	4.856889	0.291959
C	1.165347	0.548594	-2.914325
H	2.159403	0.084105	-2.977335
H	0.874122	0.865140	-3.916961
C	2.374330	0.280401	-0.228751
C	1.474152	-0.729159	-0.004810
C	1.837852	-2.098037	0.435251
C	2.792213	-2.844046	-0.279888
C	1.200698	-2.699179	1.532717
C	3.116959	-4.142965	0.108213
H	3.277068	-2.394440	-1.141202
C	1.530270	-3.998304	1.924710
H	0.442521	-2.140048	2.075012
C	2.490260	-4.724995	1.215120
H	3.856821	-4.704090	-0.456364
H	1.032789	-4.444130	2.781818
H	2.742047	-5.738131	1.515794
C	3.820126	0.213918	0.119980
C	4.771934	0.772291	-0.754371
C	4.274898	-0.355427	1.323669
C	6.132978	0.731602	-0.452695
H	4.437766	1.232854	-1.679833
C	5.635597	-0.388525	1.628446
H	3.555382	-0.767063	2.023498
C	6.571576	0.148688	0.739818
H	6.851202	1.156842	-1.148520
H	5.964612	-0.828761	2.565868
H	7.631145	0.121334	0.978193
C	-0.020010	-0.952386	-1.246123
C	-0.719675	-2.395705	-2.845144
N	0.226017	-0.501728	-2.516753
N	-0.172487	-1.372056	-3.490681
C	-0.670806	-2.211668	-1.436636
H	-1.131386	-3.230574	-3.398750
H	-0.816737	-2.961528	-0.670241
Pd	-0.355021	0.144655	0.412791
O	-2.377213	1.518910	0.381807
C	-2.862190	1.897390	-0.771592

C	-2.857370	3.386763	-1.031288
O	-0.040745	1.265775	2.429626
C	-0.260338	2.468092	2.619282
C	0.529081	3.287352	3.600139
Ag	-2.766165	-1.155382	-1.008191
O	-3.312828	1.104782	-1.616737
O	-1.196637	3.149597	1.990726
O	-4.370151	-1.515529	0.754628
C	-4.346101	-1.008486	1.878606
C	-5.077317	-1.597163	3.051992
O	-3.682350	0.093444	2.183501
H	-3.226598	0.510262	1.393806
H	-4.354227	-1.872649	3.826893
H	-5.751016	-0.849494	3.481382
H	-5.640541	-2.477358	2.743059
H	1.256001	2.662965	4.119284
H	1.046902	4.087700	3.061408
H	-0.145995	3.758995	4.320689
H	-3.506668	3.883299	-0.302059
H	-1.844677	3.776047	-0.892913
H	-3.204479	3.608851	-2.040920
H	-1.689166	2.544209	1.338199

IX

Electronic Energy BS1 = -1994.32081441 Hartree

Electronic Energy BS2 = -1996.77837100 Hartree

Zero-point Energy Correction = 0.533361 Hartree

Thermal Correction to Enthalpy = 0.575563 Hartree

Thermal Correction to Free Energy = 0.452965 Hartree

Chemical symbol X, Y, Z

C	-1.712401	1.364726	-2.529956
C	-1.409130	1.834160	-1.233667
C	-1.053097	3.190896	-1.086931
C	-0.938406	4.013251	-2.215179
C	-1.205229	3.522839	-3.491954
C	-1.606596	2.190984	-3.644006
H	-2.019166	0.330618	-2.653421
H	-0.655058	5.053902	-2.079728
H	-1.117138	4.174171	-4.356525
H	-1.835082	1.796435	-4.630111
C	-0.833444	3.765727	0.288300
H	-1.710740	3.594363	0.922024
H	-0.635455	4.836483	0.250203
C	-1.525795	0.866562	-0.088528
C	-0.658043	0.842083	1.046615
C	-1.071904	0.150285	2.324574
C	-1.972563	0.805361	3.178992
C	-0.557492	-1.094334	2.709256
C	-2.360121	0.220744	4.385816
H	-2.374931	1.772169	2.888317

C	-0.939862	-1.677503	3.920412	C	3.008941	-2.300553	-3.377296
H	0.129018	-1.611240	2.046640	O	-1.071838	-3.849330	-0.848581
C	-1.843456	-1.023079	4.761164	C	-1.373934	-3.456867	-1.963059
H	-3.065454	0.735981	5.032189	C	-2.731271	-3.635494	-2.591416
H	-0.535469	-2.646529	4.200064	Ag	2.717662	0.854991	-0.161803
H	-2.145056	-1.478585	5.700245	O	3.251692	-0.297213	-2.089113
C	-2.857503	0.165634	-0.008738	O	-0.532214	-2.783872	-2.760890
C	-4.028858	0.935626	-0.111891	O	3.524183	-1.093747	1.434550
C	-2.983617	-1.218217	0.187367	C	2.909610	-2.097434	1.779644
C	-5.287465	0.341771	-0.007678	C	2.920702	-2.636876	3.182403
H	-3.951240	2.007726	-0.269356	O	2.133119	-2.807429	0.961973
C	-4.242486	-1.814115	0.284677	H	2.055406	-2.376112	0.072027
H	-2.090233	-1.829726	0.246735	H	2.020138	-2.273678	3.691927
C	-5.399814	-1.037260	0.190567	H	2.893278	-3.728762	3.187373
H	-6.180006	0.957050	-0.082741	H	3.798314	-2.271034	3.716205
H	-4.314275	-2.889122	0.425219	H	-3.297002	-4.392570	-2.047655
H	-6.379265	-1.501505	0.264935	H	-3.268475	-2.682181	-2.535059
C	0.412066	1.842143	1.283041	H	-2.642262	-3.909242	-3.645885
C	2.235197	3.006355	1.861278	H	3.188629	-3.300195	-2.969672
N	0.315415	3.149093	0.944983	H	2.174022	-2.380036	-4.081946
N	1.415825	3.876374	1.266215	H	3.895800	-1.945845	-3.903040
C	1.671864	1.706001	1.909095	H	0.308029	-2.586683	-2.267199
H	3.193462	3.334072	2.242891				
H	2.034396	0.835455	2.437962				
Pd	0.176220	-0.408127	-0.504662				
O	1.624453	-1.754571	-1.519276				
C	2.623397	-1.360926	-2.258081				

17.4. Cartesian coordinates in Å, energy values in Hartrees and imaginary frequencies in cm^{-1} for transition states (TS) of species in Figure S5

XVIII

Electronic Energy BS1 = -1080.38309287 Hartree

Electronic Energy BS2 = -1081.44658733 Hartree

Zero-point Energy Correction = 0.285016 Hartree

Thermal Correction to Enthalpy = 0.307802 Hartree

Thermal Correction to Free Energy = 0.230245 Hartree

Chemical symbol X, Y, Z

Pd	-1.241223	-0.512117	-0.074831
O	-2.511178	0.991208	-0.496151
C	-2.001412	2.143497	-0.860492
O	-0.799614	2.411311	-0.903492
C	-3.073104	3.153032	-1.238639
H	-3.773388	3.286304	-0.408161
H	-3.645807	2.782662	-2.095109
H	-2.613316	4.109738	-1.491997
C	-0.174889	0.830505	1.752709
C	-1.386169	0.150535	2.055501
C	-0.945663	-0.963272	2.853729
H	-2.378308	0.581178	2.040419
H	-1.554668	-1.730070	3.313743
H	0.000243	1.751342	1.214540
N	0.820915	0.114034	2.294103
N	0.365668	-0.993635	2.965203
C	2.265446	0.357605	2.153427
H	2.537145	1.209275	2.782954
H	2.752171	-0.535573	2.549369
C	2.637186	0.608115	0.708976
C	3.042679	1.877600	0.286183
C	2.521945	-0.426860	-0.229827
C	3.328037	2.114029	-1.062278
H	3.128020	2.683379	1.010817
C	2.796292	-0.189667	-1.576110
H	2.187796	-1.406557	0.095380
C	3.200732	1.083073	-1.995264
H	3.641951	3.103674	-1.382173
H	2.695610	-0.995469	-2.298133
H	3.415369	1.267962	-3.044123
O	-0.099431	-2.250952	-0.018984
C	-0.216071	-2.426013	-1.289253
O	-0.937703	-1.601206	-1.936743
C	0.513301	-3.540837	-1.964386
H	0.527847	-4.425086	-1.322578
H	0.053182	-3.772786	-2.926653
H	1.549853	-3.226330	-2.133900

$TSX_{VIII-XIX}$

Imaginary Freq = -866.3815 (cm^{-1})

Electronic Energy BS1 = -1080.35758032 Hartree

Electronic Energy BS2 = -1081.42045965 Hartree

Zero-point Energy Correction = 0.279919 Hartree

Thermal Correction to Enthalpy = 0.302317 Hartree

Thermal Correction to Free Energy = 0.223141 Hartree

Chemical symbol X, Y, Z

Pd	-1.747648	0.274746	-0.007880
O	-1.330074	2.271162	-0.193054
C	-0.280671	2.848432	0.239334
O	0.634662	2.275065	0.906738
C	-0.108131	4.306849	-0.099948
H	-1.065243	4.764466	-0.352068
H	0.558600	4.376235	-0.967085
H	0.363490	4.831357	0.733433
C	-0.071354	-0.230423	1.138387
C	-0.241171	-0.719655	2.456261
C	0.557224	-1.856951	2.551158
H	-0.858433	-0.272278	3.222576
H	0.713073	-2.519258	3.392560
H	0.301004	1.006489	1.025526
N	0.809619	-1.138128	0.556788
N	1.199196	-2.094358	1.385668
C	1.381377	-1.063600	-0.794613
H	1.212440	-2.028601	-1.278709
H	0.802905	-0.306056	-1.327620
C	2.853927	-0.715597	-0.769339
C	3.821984	-1.715660	-0.915998
C	3.261076	0.610946	-0.570733
C	5.181354	-1.395972	-0.868467
H	3.509487	-2.745408	-1.068345
C	4.618815	0.930452	-0.519489
H	2.513591	1.389730	-0.445916
C	5.581918	-0.072384	-0.669693
H	5.925086	-2.179133	-0.986920
H	4.925443	1.961537	-0.366213
H	6.638682	0.177417	-0.633326
O	-2.561673	-1.624678	-0.131656
C	-3.565423	-1.209441	-0.824491
O	-3.631267	0.031311	-1.097771
C	-4.591257	-2.183447	-1.308492
H	-4.753629	-2.964293	-0.561735
H	-5.527120	-1.668664	-1.534042
H	-4.220475	-2.658648	-2.224136

XIX

Electronic Energy BS1 = -1080.38570589 Hartree

Electronic Energy BS2 = -1081.44841903 Hartree

Zero-point Energy Correction = 0.285722 Hartree

Thermal Correction to Enthalpy = 0.308287 Hartree

Thermal Correction to Free Energy = 0.231801 Hartree

Chemical symbol X, Y, Z

Pd	-1.153946	0.069104	0.487279
O	0.486375	0.862982	1.529012
C	1.558813	0.419502	1.973743
O	1.905215	-0.847078	1.948692
C	2.546276	1.320764	2.642612
H	2.345505	2.358153	2.378238
H	3.563338	1.040099	2.363026
H	2.444014	1.195919	3.727011
C	-0.275827	-1.648030	0.067530
C	-0.162373	-2.823896	0.818218
C	0.600578	-3.700957	0.011352
H	-0.591855	-3.015751	1.791680
H	0.909198	-4.716824	0.225521
H	1.211538	-1.397573	1.503494
N	0.394727	-1.886127	-1.095553
N	0.941012	-3.133125	-1.145276
C	0.626032	-0.947486	-2.193646
H	1.103774	-1.529601	-2.985303
H	-0.339367	-0.592616	-2.563876
C	1.493004	0.219575	-1.767217
C	2.744899	-0.014477	-1.182625
C	1.041859	1.535332	-1.914093
C	3.531624	1.053016	-0.749560
H	3.091281	-1.036743	-1.054661
C	1.830626	2.606874	-1.484478
H	0.066393	1.720056	-2.356959
C	3.074814	2.367763	-0.898108
H	4.499300	0.861308	-0.294380
H	1.468204	3.624577	-1.600506
H	3.686686	3.198143	-0.557087
O	-2.945961	-0.275256	-0.476167
C	-3.441535	0.879066	-0.166893
O	-2.754314	1.676890	0.532186
C	-4.807687	1.228729	-0.676958
H	-5.489009	0.385182	-0.536596
H	-5.190194	2.113745	-0.165919
H	-4.744592	1.432940	-1.751741

17.5. Cartesian coordinates in Å, energy values in Hartrees and imaginary frequencies in cm^{-1} for transition states (TS) of species in Figure S6

XX

Electronic Energy BS1 = -1454.72905834 Hartree

Electronic Energy BS2 = -1457.07671800 Hartree

Zero-point Energy Correction = 0.337354 Hartree

Thermal Correction to Enthalpy = 0.367577 Hartree

Thermal Correction to Free Energy = 0.272473 Hartree

Chemical symbol X, Y, Z

Pd	0.316012	-0.287555	0.239123
O	1.100525	-1.402522	-1.356392
C	2.283153	-1.893160	-1.307814
C	2.477785	-3.175918	-2.091636
O	0.028046	1.408490	-0.902436
C	0.751541	2.468925	-0.889236
C	0.108357	3.685529	-1.518787
Ag	3.035703	0.698698	-0.005751
O	3.265371	-1.425919	-0.672575
O	1.896268	2.587906	-0.382856
H	-0.603600	3.398125	-2.294739
H	-0.438305	4.212660	-0.729056
H	0.868641	4.354907	-1.925809
H	1.897747	-3.156079	-3.017026
H	3.533961	-3.344539	-2.307555
H	2.103219	-4.004996	-1.479700
O	0.562338	-1.956902	1.348705
C	-0.438545	-2.800053	1.374256
O	-1.554333	-2.603796	0.886985
C	-0.081006	-4.103571	2.070004
H	0.456256	-3.911845	3.002993
H	0.582382	-4.684533	1.419450
H	-0.983840	-4.683659	2.269777
C	-1.659655	0.470992	1.594898
C	-0.388956	0.676460	2.190186
C	-0.218730	2.099789	2.130459
H	0.124063	-0.008338	2.850405
H	0.613965	2.685590	2.494809
H	-2.236813	-0.434179	1.475067
N	-2.097503	1.675037	1.192807
N	-1.228728	2.681671	1.515042
C	-3.309237	1.989764	0.419937
H	-4.086261	2.323808	1.113640
H	-3.042317	2.829183	-0.226014
C	-3.775949	0.803180	-0.389849
C	-4.928596	0.100420	-0.024236
C	-3.030150	0.376050	-1.498929
C	-5.338840	-1.015210	-0.760800
H	-5.504816	0.425278	0.838291

C	-3.437646	-0.738465	-2.231564
H	-2.120093	0.905862	-1.763042
C	-4.593481	-1.436698	-1.863882
H	-6.236505	-1.553893	-0.470532
H	-2.854403	-1.063376	-3.088973
H	-4.910250	-2.304473	-2.435785

TS_{XX-XXI}

Imaginary Freq = -813.2746 (cm^{-1})

Electronic Energy BS1 = -1454.70932870 Hartree

Electronic Energy BS2 = -1457.05503755 Hartree

Zero-point Energy Correction = 0.332287 Hartree

Thermal Correction to Enthalpy = 0.361854 Hartree

Thermal Correction to Free Energy = 0.268886 Hartree

Chemical symbol X, Y, Z

Pd	0.163387	-0.274646	-0.199653
O	1.106101	-1.787228	-1.346611
C	2.021599	-2.540879	-0.871945
C	2.150684	-3.902827	-1.527773
O	0.876642	1.265720	-1.376891
C	1.758752	2.131083	-1.023333
C	1.688932	3.441750	-1.779053
Ag	2.938444	-0.100920	0.608995
O	2.800261	-2.270018	0.084659
O	2.624243	1.994880	-0.123108
H	1.437560	3.270672	-2.828221
H	0.884570	4.039147	-1.334485
H	2.628500	3.990023	-1.696518
H	1.926103	-3.841900	-2.595003
H	3.148422	-4.317032	-1.372186
H	1.416106	-4.574293	-1.067706
O	-0.676019	-1.863383	0.800792
C	-1.683981	-1.758981	1.574595
O	-2.196405	-0.666705	1.957226
C	-2.320845	-3.045991	2.038343
H	-1.614461	-3.875074	1.980080
H	-3.168184	-3.255289	1.375722
H	-2.701998	-2.934478	3.055344
C	-0.783238	1.256497	0.902239
C	-0.060375	1.982674	1.878267
C	-0.335413	3.328218	1.646787
H	0.549779	1.550846	2.658939
H	0.008882	4.204618	2.179876
H	-1.526922	0.314433	1.379250
N	-1.429679	2.242949	0.165461
N	-1.170649	3.471826	0.593740
C	-2.220690	2.057645	-1.058820
H	-2.786580	2.982294	-1.190855
H	-1.519081	1.952101	-1.891060

C	-3.132251	0.856718	-0.963637
C	-4.230573	0.874997	-0.094206
C	-2.869498	-0.298244	-1.709057
C	-5.048920	-0.246411	0.033702
H	-4.434423	1.769628	0.489229
C	-3.684978	-1.426042	-1.578783
H	-2.021193	-0.315749	-2.388137
C	-4.774076	-1.402497	-0.704772
H	-5.898420	-0.222264	0.710629
H	-3.467927	-2.319160	-2.158275
H	-5.410463	-2.277356	-0.603584

XXI

Electronic Energy BS1 = -1454.72959152 Hartree

Electronic Energy BS2 = -1457.07761014 Hartree

Zero-point Energy Correction = 0.337195 Hartree

Thermal Correction to Enthalpy = 0.367698 Hartree

Thermal Correction to Free Energy = 0.270065 Hartree

Chemical symbol X, Y, Z

Pd	0.164278	-0.093043	-0.222094
O	0.953214	-1.841301	-1.235015
C	1.754757	-2.653836	-0.673603
C	1.688049	-4.095105	-1.145541
O	1.012090	1.286244	-1.500703
C	1.991669	2.053650	-1.178479
C	2.129607	3.290334	-2.042003
Ag	2.927603	-0.206097	0.585652
O	2.581019	-2.380925	0.248820
O	2.799511	1.882045	-0.231852
H	1.892173	3.062850	-3.083641
H	1.405456	4.033492	-1.688149
H	3.133951	3.708801	-1.962799
H	1.509416	-4.138373	-2.222694
H	2.600147	-4.637715	-0.890786
H	0.839808	-4.580087	-0.647983
O	-0.879085	-1.542119	0.918071
C	-1.714841	-1.497336	1.834877
O	-2.013395	-0.414789	2.518274
C	-2.463735	-2.718519	2.263620
H	-2.160151	-3.577262	1.666359
H	-3.535757	-2.536650	2.142082
H	-2.274144	-2.907639	3.324757
C	-0.571590	1.485176	0.722839
C	-0.262168	2.032036	1.973404
C	-1.102371	3.163659	2.100087
H	0.482031	1.671305	2.669917
H	-1.173669	3.862967	2.924190
H	-1.495099	0.364504	2.190027
N	-1.533436	2.295754	0.198465
N	-1.875494	3.322380	1.025426

C	-2.211588	2.129763	-1.086062
H	-2.895207	2.977471	-1.176342
H	-1.466568	2.197067	-1.883315
C	-2.953305	0.809782	-1.163518
C	-3.835732	0.437077	-0.140112
C	-2.738276	-0.067525	-2.232280
C	-4.485144	-0.796759	-0.181808
H	-3.997727	1.110730	0.697130
C	-3.393557	-1.302218	-2.279422
H	-2.046919	0.211719	-3.023327
C	-4.263111	-1.672028	-1.251306
H	-5.164344	-1.076614	0.618648
H	-3.216243	-1.975482	-3.113644
H	-4.767615	-2.633665	-1.281884

17.6. Cartesian coordinates in Å, energy values in Hartrees and imaginary frequencies in cm^{-1} for transition states (TS) of species in Figure S7

XXII

Electronic Energy BS1 = -2242.12591302 Hartree

Electronic Energy BS2 = -2244.22852889 Hartree

Zero-point Energy Correction = 0.640339 Hartree

Thermal Correction to Enthalpy = 0.687055 Hartree

Thermal Correction to Free Energy = 0.556542 Hartree

Chemical symbol X, Y, Z			
Pd	-1.423814	0.050128	-0.511147
Pd	1.478161	-1.671129	-1.118861
N	1.824434	3.510924	-1.521599
N	1.676496	3.439785	-0.178299
C	2.523383	4.273048	0.476636
H	2.527214	4.326565	1.555924
C	3.274739	4.925189	-0.488316
H	4.050454	5.660235	-0.329649
C	2.796937	4.408794	-1.711588
H	3.116624	4.649608	-2.717683
C	0.677685	2.553214	0.404045
H	0.832752	2.580309	1.485034
H	0.874668	1.533281	0.073444
C	-0.738599	2.959616	0.051390
C	-1.709371	2.020485	-0.318417
C	-3.013845	2.439389	-0.612405
H	-3.773047	1.711738	-0.887266
C	-3.356657	3.793082	-0.552211
H	-4.371195	4.103805	-0.788425
C	-2.393400	4.736096	-0.184044
H	-2.649166	5.790621	-0.129721
C	-1.099401	4.316228	0.115713
H	-0.345348	5.047380	0.397814

N	1.409535	-3.042816	0.425620	H	0.885145	2.261539	-3.916125
N	2.478009	-3.389317	1.175534	O	-0.056267	-2.771724	-2.254449
C	2.099671	-4.134778	2.234920	C	-1.261706	-2.797440	-1.882903
H	2.832645	-4.511188	2.933387	O	-1.743706	-2.134344	-0.909763
C	0.719344	-4.272733	2.174672	C	-2.221964	-3.708660	-2.624440
H	0.084578	-4.802152	2.869282	H	-3.052313	-3.113393	-3.018179
C	0.328848	-3.570298	1.026718	H	-2.644988	-4.434187	-1.921587
H	-0.654108	-3.375633	0.624880	H	-1.727107	-4.237463	-3.440641
C	3.821122	-3.003956	0.752184	C	-2.730856	-0.295395	1.253404
H	4.031327	-3.527171	-0.187779	C	-1.593116	-0.054274	1.695576
H	4.508216	-3.378393	1.511683	C	-4.137124	-0.513959	1.072278
C	3.956663	-1.506999	0.571180	C	-0.501863	0.330805	2.547697
C	3.070888	-0.787973	-0.257474	C	-4.623000	-1.576690	0.286473
C	3.325454	0.572839	-0.476660	C	-5.040601	0.378126	1.684487
H	2.696973	1.136654	-1.153661	C	0.779361	-0.227111	2.399020
C	4.393864	1.223316	0.151981	C	-0.713607	1.348771	3.498437
H	4.554821	2.282297	-0.030231	C	-5.996754	-1.744946	0.129945
C	5.239743	0.515090	1.005541	H	-3.910276	-2.234395	-0.198816
H	6.066133	1.012930	1.505205	C	-6.412109	0.196212	1.519665
C	5.023066	-0.850433	1.201796	H	-4.658603	1.203293	2.277148
H	5.692016	-1.418915	1.844147	C	1.836433	0.232935	3.179135
O	-0.709086	0.435122	-2.481483	H	0.942539	-0.988987	1.650710
C	0.436178	0.395869	-3.020963	C	0.349959	1.799625	4.278187
O	1.464051	-0.244443	-2.634193	H	-1.700955	1.787574	3.602034
C	0.621523	1.253377	-4.256609	C	-6.892913	-0.863331	0.743946
H	-0.308907	1.315463	-4.824906	H	-6.370513	-2.565559	-0.475710
H	1.429104	0.873723	-4.885082	H	-7.105728	0.884600	1.993498

C	1.626336	1.249015	4.116755
H	2.825804	-0.191713	3.038758
H	0.184926	2.589303	5.005245
H	-7.962876	-0.999694	0.615681
H	2.454121	1.614222	4.717744

$TS_{XXII\text{-}XXIII}$

Imaginary Freq = -219.9886 (cm^{-1})

Electronic Energy BS1 = -2242.10933079 Hartree

Electronic Energy BS2 = -2244.21001631 Hartree

Zero-point Energy Correction = 0.640133 Hartree

Thermal Correction to Enthalpy = 0.685929 Hartree

Thermal Correction to Free Energy = 0.558271 Hartree

Chemical symbol X, Y, Z

Pd	0.784171	-1.296689	-0.954874
Pd	0.180362	1.873889	-1.246006
N	-4.388767	-0.810860	-1.196252
N	-3.825341	-0.788665	0.034770
C	-4.748708	-0.668751	1.022223
H	-4.448937	-0.633816	2.059969
C	-5.988139	-0.601925	0.407459
H	-6.951732	-0.494958	0.884175
C	-5.701134	-0.692849	-0.972130
H	-6.389595	-0.673231	-1.807638
C	-2.390322	-0.922352	0.185995
H	-2.125889	-0.467040	1.141892
H	-1.908519	-0.327415	-0.584113
C	-1.883387	-2.345019	0.126177
C	-0.497901	-2.593428	0.038030
C	-0.013711	-3.910884	0.071307
H	1.056381	-4.092041	0.075458
C	-0.900721	-4.988958	0.120930
H	-0.516411	-6.004908	0.132899
C	-2.273793	-4.747951	0.181810
H	-2.972895	-5.577427	0.237549
C	-2.755040	-3.435644	0.193258
H	-3.824400	-3.257514	0.251410
N	1.666510	2.367607	0.085901
N	1.532190	3.174936	1.159378
C	2.613042	3.080598	1.966365
H	2.681556	3.676328	2.864764
C	3.478815	2.156763	1.397735
H	4.427725	1.819128	1.785969
C	2.844439	1.738527	0.219857
H	3.149796	1.004051	-0.506529
C	0.423954	4.120132	1.227572
H	0.588010	4.870654	0.444711
H	0.502864	4.619798	2.193599
C	-0.939167	3.483436	1.055113

C	-1.237462	2.596794	-0.002197
C	-2.578921	2.225661	-0.185694
H	-2.852460	1.594057	-1.021292
C	-3.585252	2.650817	0.688221
H	-4.608328	2.324053	0.522923
C	-3.270100	3.474633	1.767852
H	-4.039200	3.797877	2.463719
C	-1.950864	3.896824	1.934978
H	-1.698839	4.568427	2.752620
O	-0.643093	-1.004274	-2.620165
C	-1.293031	-0.010270	-3.043571
O	-1.246601	1.193735	-2.614820
C	-2.308230	-0.266340	-4.140333
H	-1.981826	-1.087691	-4.781686
H	-2.486896	0.633035	-4.732851
H	-3.246125	-0.557241	-3.653286
O	1.712859	1.427257	-2.788092
C	2.395083	0.387986	-2.990102
O	2.350277	-0.687563	-2.310605
C	3.378391	0.395063	-4.146734
H	3.010661	-0.278949	-4.928406
H	4.347811	0.013994	-3.812837
H	3.493971	1.397056	-4.562887
C	1.911904	-1.466290	0.732437
C	0.761156	-1.471561	1.305917
C	3.329446	-1.480792	1.027726
C	-0.045353	-1.026634	2.425586
C	4.307418	-1.444095	0.017937
C	3.734722	-1.498072	2.379849
C	-0.026933	0.341291	2.757730
C	-0.857801	-1.911107	3.155915
C	5.660397	-1.409669	0.356077
H	3.992751	-1.416897	-1.018959
C	5.087019	-1.461297	2.706738
H	2.980991	-1.528035	3.161026
C	-0.818598	0.815932	3.801922
H	0.587084	1.017999	2.176664
C	-1.639561	-1.429518	4.203589
H	-0.881380	-2.961523	2.885307
C	6.055342	-1.414711	1.696457
H	6.408207	-1.375411	-0.431243
H	5.387865	-1.468433	3.750536
C	-1.628260	-0.066484	4.522888
H	-0.814801	1.875465	4.038454
H	-2.265725	-2.114971	4.767031
H	7.109965	-1.384910	1.954820
H	-2.252308	0.305795	5.330221

XXIII

Electronic Energy BS1 = -2242.14803576 Hartree

Electronic Energy BS2 = -2244.24300176 Hartree

Zero-point Energy Correction = 0.642897 Hartree

Thermal Correction to Enthalpy = 0.688480 Hartree

Thermal Correction to Free Energy = 0.560743 Hartree

Chemical symbol X, Y, Z

Pd	-0.447335	1.643264	0.858054
Pd	-0.517941	0.497606	-2.102532
N	4.261663	0.730727	-0.277837
N	4.049952	-0.358803	0.498025
C	5.169858	-1.114642	0.635954
H	5.161903	-2.019912	1.225985
C	6.170008	-0.491860	-0.090893
H	7.193929	-0.815809	-0.208164
C	5.545889	0.650500	-0.637744
H	5.973425	1.414520	-1.274766
C	2.740802	-0.625309	1.070939
H	2.798552	-1.615873	1.527862
H	2.010421	-0.691198	0.261798
C	2.295846	0.407461	2.084343
C	0.924797	0.476910	2.480131
C	0.551092	1.474453	3.427997
H	-0.460753	1.465507	3.819588
C	1.476728	2.392955	3.926339
H	1.158103	3.143786	4.643020
C	2.808644	2.300719	3.535197
H	3.547994	2.987521	3.936771
C	3.206523	1.308322	2.631102
H	4.247907	1.254220	2.333783
N	-2.101472	-0.815668	-1.972419
N	-2.046206	-2.150954	-2.182919
C	-3.192424	-2.744212	-1.777704
H	-3.330911	-3.808730	-1.898101
C	-4.018359	-1.756276	-1.265207
H	-4.995933	-1.878190	-0.824842
C	-3.294638	-0.564977	-1.413647
H	-3.556269	0.440504	-1.135613
C	-1.014511	-2.718751	-3.043495
H	-1.224136	-2.380828	-4.066455
H	-1.152608	-3.800355	-3.015212
C	0.397550	-2.343776	-2.658298
C	0.786243	-1.020521	-2.360891
C	2.155875	-0.761749	-2.198797
H	2.492851	0.243247	-1.987900
C	3.112060	-1.780436	-2.275712
H	4.160760	-1.537866	-2.126635
C	2.713911	-3.093262	-2.520047
H	3.443127	-3.897452	-2.564709
C	1.359624	-3.363682	-2.716141
H	1.038449	-4.380414	-2.930944
O	0.927381	2.963051	-0.283245
C	1.371162	2.791329	-1.448413
O	1.012052	1.909179	-2.301360
C	2.502781	3.697453	-1.898217
H	2.387070	4.695786	-1.470212
H	2.560005	3.754593	-2.986778

H	3.439702	3.271526	-1.520969
O	-1.977676	2.187106	-2.018314
C	-2.397907	2.913683	-1.080465
O	-2.069759	2.848300	0.150925
C	-3.457967	3.950218	-1.410616
H	-3.295430	4.864202	-0.834578
H	-4.436813	3.545161	-1.127798
H	-3.466839	4.172495	-2.478979
C	-1.324729	0.102997	1.738957
C	-0.156012	-0.511968	2.086181
C	-2.723184	-0.274638	1.881332
C	0.123001	-1.963873	2.058528
C	-3.756273	0.615998	1.519195
C	-3.092300	-1.514184	2.458875
C	-0.321343	-2.738163	0.971115
C	0.876280	-2.580096	3.071610
C	-5.095963	0.268035	1.685051
H	-3.492806	1.580910	1.104917
C	-4.430887	-1.861963	2.605406
H	-2.326199	-2.207422	2.783551
C	-0.025460	-4.097430	0.902987
H	-0.879157	-2.257637	0.175169
C	1.168066	-3.943365	3.002479
H	1.234449	-1.986846	3.908520
C	-5.442988	-0.976579	2.215013
H	-5.870480	0.972252	1.394015
H	-4.687360	-2.826012	3.035999
C	0.720293	-4.705246	1.918799
H	-0.364558	-4.678724	0.050134
H	1.747863	-4.410416	3.793671
H	-6.486957	-1.251348	2.335619
H	0.957300	-5.763872	1.862786
